# Prenatal exposure to maternal disadvantage-related inflammatory biomarkers: associations with neonatal white matter microstructure

**DOI:** 10.1038/s41398-024-02782-6

**Published:** 2024-02-02

**Authors:** Ashley F. P. Sanders, Brian Tirado, Nicole A. Seider, Regina L. Triplett, Rachel E. Lean, Jeffrey J. Neil, J. Philip Miller, Rebecca Tillman, Tara A. Smyser, Deanna M. Barch, Joan L. Luby, Cynthia E. Rogers, Christopher D. Smyser, Barbara B. Warner, Edith Chen, Gregory E. Miller

**Affiliations:** 1 Department of Psychiatry, Washington University School of Medicine, St. Louis, MO 63110 USA; 2 Department of Neurology, Washington University School of Medicine, St. Louis, MO 63110 USA; 3 Division of Biostatistics, Institute for Informatics, Washington University School of Medicine, St. Louis, MO 63110 USA; 4 Department of Psychological and Brain Sciences, Washington University School of Medicine, St. Louis, MO 63130 USA; 5 Department of Pediatrics, Washington University School of Medicine, St. Louis, MO 63110 USA; 6 Department of Radiology, Washington University School of Medicine, St. Louis, MO 63110 USA; 7 Newborn Medicine, Washington University School of Medicine, St. Louis, MO 63110 USA; 8 Institute for Policy Research, Northwestern University, Evanston, IL 60208 USA; 9 Department of Psychology, Northwestern University, Evanston, IL 60208 USA

**Keywords:** Molecular neuroscience, Human behaviour

## Abstract

Prenatal exposure to heightened maternal inflammation has been associated with adverse neurodevelopmental outcomes, including atypical brain maturation and psychiatric illness. In mothers experiencing socioeconomic disadvantage, immune activation can be a product of the chronic stress inherent to such environmental hardship. While growing preclinical and clinical evidence has shown links between altered neonatal brain development and increased inflammatory states in utero, the potential mechanism by which socioeconomic disadvantage differentially impacts neural-immune crosstalk remains unclear. In the current study, we investigated associations between socioeconomic disadvantage, gestational inflammation, and neonatal white matter microstructure in 320 mother-infant dyads over-sampled for poverty. We analyzed maternal serum levels of four cytokines (IL-6, IL-8, IL-10, TNF-α) over the course of pregnancy in relation to offspring white matter microstructure and socioeconomic disadvantage. Higher average maternal IL-6 was associated with very low socioeconomic status (SES; INR < 200% poverty line) and lower neonatal corticospinal fractional anisotropy (FA) and lower uncinate axial diffusivity (AD). No other cytokine was associated with SES. Higher average maternal IL-10 was associated with lower FA and higher radial diffusivity (RD) in corpus callosum and corticospinal tracts, higher optic radiation RD, lower uncinate AD, and lower FA in inferior fronto-occipital fasciculus and anterior limb of internal capsule tracts. SES moderated the relationship between average maternal TNF-α levels during gestation and neonatal white matter diffusivity. When these interactions were decomposed, the patterns indicated that this association was significant and positive among very low SES neonates, whereby TNF-α was inversely and significantly associated with inferior cingulum AD. By contrast, among the more advantaged neonates (lower-to-higher SES [INR ≥ 200% poverty line]), TNF-α was positively and significantly associated with superior cingulum AD. Taken together, these findings suggest that the relationship between prenatal cytokine exposure and white matter microstructure differs as a function of SES. These patterns are consistent with a scenario where gestational inflammation’s effects on white matter development diverge depending on the availability of foundational resources in utero.

## Introduction

During intrauterine life, there are sensitive periods of development when exposures and experiences can have especially large influences that contribute to fetal programming (i.e., the formation of tissues and organs) [1]. Indeed, the plastic fetal brain is highly sensitive to its environment, with studies indicating that exposure to a variety of external factors (e.g., prenatal stress, maternal diet and nutrition, and environmental toxins) can affect brain development [2]. The concept of fetal programming suggests that such exposures shape brain development in a durable manner, to prepare calibrating the offspring’s behavioral phenotype in a manner that is suited to meet the demands of its likely postnatal environment [3–5]. This environmental sensitivity, part of a “predictive adaptive response”, is generally advantageous in that prenatal environmental cues prepare the fetus for the postnatal environment [6, 7]. Specifically, transmission of maternal biological signals across the placenta during gestation is thought to cue the developing fetus about aspects of extrauterine life that reflect the environment into which they will be born [8–10]. In addition to glucocorticoid hormones, circulating metabolites, and epigenetic processes within the placenta, these signals might include inflammatory cytokines, which can serve as mediators of normative neural development [11–14]. Some cytokines have been implicated in neuronal and glial cell survival and growth, the modulation of synaptic plasticity and axon pathfinding, and neuronal specification and differentiation [15, 16]. Furthermore, cytokines coordinate immune responses to wounding and injury [11–14]. Assuming the precipitating adverse stimulus (e.g., infection, trauma, disease) is eliminated, inflammatory responses are typically acute and controlled by regulatory signals. However, stressors like maternal social disadvantage and racial discrimination can interfere with these regulatory processes, leading to excess inflammatory activity in the placenta’s chorionic villous layer, which functions as the maternal-fetal interface [17–20]. This “non-resolving” inflammation is hypothesized to affect the structural and functional development of multiple fetal organ systems, including the brain [21–23]. Maternal inflammation during pregnancy has, therefore, garnered substantial attention in the investigation of fetal neurodevelopment.

The maternal inflammatory response plays multiple and shifting roles over the course of pregnancy, which include protecting against infection, shaping the intrauterine environment, promoting fetal development, and facilitating childbirth. The formation of neural tissue heavily relies on the fine-tuned cellular signaling of each gestational stage. Consequently, aberrations in these rhythms brought on by adverse maternal environmental exposures and resulting dysregulation of inflammatory cytokine profiles release can alter the neural developmental pathways and ultimately result in subtle, but impactful, structural differences in the fetal brain [24, 25]. The mechanism by which maternal cytokines reach the developing fetus remains a subject of debate; however, the prevailing consensus supports the notion that the maternal immune response influences the fetus through placental tissue [26–28]. Ex-vivo investigations of term placenta suggest that direct placental transfer of maternal cytokines does not occur in most circumstances, though it is possible. A more likely scenario is that maternal inflammation stimulates placental expression of cytokines – or other mediators – that ultimately reach fetal circulation and affect tissue development [29]. Consistent with that scenario, chronic maternal infections (e.g., HIV, hepatitis B) have been associated with elevated cytokine levels in cord blood and modified fetal immune responses, suggesting that maternal immune responses may affect the fetus through production of cytokines by the placenta and/or neonate [30, 31]. Thus, while maternal cytokines could plausibly cross the placenta, they also could activate signaling cascades that result in the release of cytokines or other mediators on the fetal side.

Both preclinical and clinical research has shown that excessive maternal immune activation during pregnancy alters the development of white matter microstructure in offspring [11, 32, 33]. Furthermore, multiple studies have revealed an association between elevated maternal cytokine concentrations and subsequent brain conditions in childhood and beyond, including cerebral palsy, autism, and schizophrenia [34–36]. There is also emerging evidence for increased risk of future depression and cognitive impairment in offspring [37, 38]. Related to these findings, high levels of maternal pro-inflammatory cytokines, specifically interleukin (IL)-6, IL-8, and tumor necrosis factor (TNF)-ɑ, have been shown to induce downstream neuromodulatory effects consistent with these neuropsychiatric conditions [39–41]. In contrast, increased expression of anti-inflammatory cytokines by macrophages has been shown to be neuroprotective and neuromodulatory (e.g., influencing receptor behaviors and neuron activity) in the absence of a counteractive inflammatory response.

Maternal stress and social disadvantage are increasingly recognized as risk factors for aberrant fetal neurodevelopment, including white matter development. Neonates exposed to prenatal stress have been found to have increased mean diffusivity (MD; apparent water diffusion rate), axial diffusivity (AD; i.e., apparent water diffusion parallel to axons), and radial diffusivity (RD; i.e., apparent water diffusion perpendicular to axons) in the uncinate fasciculus [42] and decreased fractional anisotropy (FA; directional heterogeneity of water diffusion) in white matter tracts including the angular gyrus, uncinate, and posterior cingulate [43]. Both decreased FA and increased MD have also been found in amygdala-frontal white matter connections and the cingulum [44]. Other work has shown increased MD, RD, and AD in right frontal areas [45] in neonates prenatally exposed to maternal stress. Taken together, these findings may reflect alterations in diffusivity influenced by membrane permeability, brain water content, oligodendrocyte proliferation, myelination, or the density of axonal packing in neonates born to mothers experiencing heightened stress during pregnancy [46–48]. However, in a sample of healthy term-born neonates from the same study sample reported here, Lean et al. [49] found that social disadvantage, a latent construct composed of income-to-needs ratio (INR), area deprivation, insurance status, parental education, and an index of healthy eating, was linked with lower MD in the inferior cingulum, uncinate, and fornix, as well as lower MD and higher FA in the dorsal cingulum. During infancy, increased FA and lower MD suggests greater white matter maturation [50]. In accordance with the Stress Acceleration Hypothesis, early life adversity may prematurely quicken the maturation of neural circuits towards a more adult-like functioning in environments where long-term survival is not guaranteed [51]. In neonates exposed to social disadvantage in utero, these findings may reflect an accelerated developmental trajectory, whereby an adverse extrauterine environment promotes a reprioritization of maturation over protracted growth [49, 51–53].

While growing evidence suggests that early exposure to chronic stress alters neurodevelopment, the mediating pathway by which this association occurs remains poorly understood. Compared to higher SES environments, residing in socioeconomically disadvantaged settings is associated with a different set of stressors, including an increased likelihood of exposure to unpredictable cues including violence, conflict, family instability, nutrient deficiencies, and caregiver distress [2]. These environmental features are hypothesized to heighten individuals’ perception of danger and uncertainty; whereby otherwise ambiguous social situations are interpreted as threatening [17]. Yet, how these environment-specific stressors modulate neural circuitry has not been clarified. To elucidate this mechanism, Nusslock and Miller [54] proposed a neuroimmune network hypothesis. This hypothesis suggests that severe chronic stress in childhood leads to excessive immune-brain crosstalk, involving elevated inflammatory activity and altered neural circuits involved in threat and reward processing. Indeed, several recent studies have observed strong relationships between inflammatory biomarkers and neural reactivity to threats and rewards among children facing chronic stressors relative to unexposed youth [17, 55, 56]. However, these existing studies have focused on children and adolescents. The question of whether these associations operate even earlier in life, for example, during highly plastic prenatal development when stress exposure might affect neural-immune communication, remains unknown.

The current study aims to fill this gap in the literature. In a sample of 320 mother-infant dyads over-sampled for exposure to poverty, we consider the relationships among socioeconomic disadvantage, gestational inflammation, and neonatal white matter microstructure. Our first hypothesis was that socioeconomic disadvantage would be associated with higher concentrations of inflammatory cytokines across pregnancy. Second, we hypothesized that disadvantage would be associated with variations in newborn white matter microstructure, as reflected in lower tract MD (suggesting lower brain water content, and in turn, maturation) and higher FA values (suggesting tighter packing of parallel fibers in unmyelinated tracts and greater or more mature myelination in myelinated tracts). Third, we hypothesized that mothers with higher cytokine concentrations during pregnancy would have newborns with significantly different white matter tract microstructure compared to those born to mothers with lower cytokine levels. Specifically, we expected higher maternal cytokine concentrations to be associated with higher MD and lower FA across white matter tracts, reflecting aberrant microstructural development (e.g., reduced axonal integrity, greater brain water content). Lastly, we hypothesized that family SES would moderate the association between maternal cytokine levels and neonatal white matter microstructure. Namely, there would be a stronger relationship between maternal cytokine concentrations and white matter microstructure in neonates from very low SES relative to lower-to-higher SES families, reflecting the excessive brain-immune crosstalk implied by the neuroimmune network hypothesis.

## Materials and methods

### Sample

The current study included 320 mother-infant dyads who participated in the Early Life Adversity, Biological Embedding, and Risk for Developmental Precursors of Mental Disorders (eLABE) study. Pregnant women were recruited from the March of Dimes Prematurity Research Center at Washington University in St. Louis from 2017-2020. Women facing social disadvantage were over-sampled by increased recruitment from a clinic serving low-income women. All study procedures were approved by the Washington University School of Medicine Institutional Review Board. Written informed consent was obtained from all mothers. eLABE exclusion criteria spanned multiple gestations, infections known to cause congenital disease (e.g., syphilis), and maternal alcohol or drug use other than tobacco and cannabis. A total of 395 pregnant women and their 399 singleton offspring were recruited for participation in eLABE (*n* = 4 mothers with 2 singleton births during recruitment). During each trimester and after birth, mothers completed detailed surveys and provided blood samples. Neonatal brain imaging was performed within the first month of life on 385 non-sedated neonates. Of these, diffusion MRI (dMRI) data was deemed unusable for *n* = 20 (no dMRI collected *n* = 3, required frames not collected *n* = 4, sequence collected in one direction *n* = 8, artifact *n* = 5). Seventeen neonates were excluded for the presence of brain injury (e.g., cerebellar, frontal, or parietal hemorrhage, multifocal periventricular leukomalacia, asymmetric mild ventriculomegaly). An additional *n* = 28 neonates were removed from analyses because of maternal conditions and/or treatments that may affect immune or inflammatory activity (i.e., diagnosis of hepatitis C, human immunodeficiency virus, syphilis, lupus, or currently taking oral or intravenous steroids). Of the 320 neonates retained for final analyses, infants born preterm (<37 weeks’ gestation; *n* = 45) or admitted to the Neonatal Intensive Care Unit >7 days (*n* = 28) were included, but these indications were controlled for as outlined in the “Statistical Analysis” section. Further, sensitivity analyses excluding neonates born <34 weeks’ gestation and born weighing <2000×*g* are included in the Supplemental Information section (Tables S8–S10).

### Measures

#### Income-to-Needs Ratio

Income-to-Needs Ratio (INR) was collected from mothers at each trimester. INR uses self-reported family income and household size compared to federal poverty thresholds, with a ratio of 1.0 being at the poverty line. The percent change in INR from the first through third trimesters was relatively small (i.e., 1.8%), and therefore, INR at the first trimester was used in the current analyses. INR was used to dichotomize the sample into very low and lower-to-higher family SES groups (defined as mean INR below or at/above 200% of the national poverty line threshold, respectively). This categorization is justified by its implementation in previous large-scale, prospective studies such as Fragile Families and the Child Wellbeing Study [57–59]. Analyses examining continuous INR are included in the Supplemental Information (Tables S6 and S7).

#### Cytokines

At each trimester, maternal antecubital blood samples were obtained during routine clinical lab visits. Samples were refrigerated at 4 °C and centrifuged for 5 minutes at 1620 x g within 12 hours of collection. Aliquots of serum and plasma (1 mL) were stored at −80 °C [60]. We measured serum levels of four inflammatory biomarkers: IL-6, IL-8, IL-10, and TNF-α. The cytokines were measured in triplicate using a multiplex immunoassay protocol on an automated microfluidic platform (Simple Plex, Protein Simple) [61]. Lower limits of detection range from 0.08 pg/mL (IL-8) to 0.28 pg/mL (TNF-α). Across runs, the average intra-assay coefficients of variation for triplicate samples were 3.6% (IL-6), 2.1% (IL-8), 2.4% (IL-10), and 3.8% (TNF-α). The corresponding inter-assay coefficients of variation were 3.6%, 3.2%, 4.5%, and 1.3%.

#### White matter microstructure

Non-sedated neonates (mean postmenstrual age [PMA] = 41 weeks, range = 37-45 weeks) underwent MRI scans on a Siemens Prisma 3 T scanner (Siemens Healthineers Erlangen, Germany) using a 64-channel head coil. Neonates were fed, swaddled, and noise protection gear was applied. They were then positioned in a stabilizing vacuum fix wrap and placed in the head coil on foam padding to decrease motion. Neonatal dMRI scans were acquired as two 5-minute runs (multiband factor=MB4, TR/TE = 2500/79.4 ms, 1.75-mm isotropic voxels) with whole brain coverage (80 slices), 108 b values sampled on 3 shells b = 500-2500 s/mm^2^ and 7 b = 0 images interspersed throughout each run with phase encoding directional reversal (anterior → posterior and posterior → anterior) for susceptibility- and eddy-current distortion correction [62]. dMRI parameters: FA, MD, AD, and RD, were extracted for the corpus callosum (CC) and eight bilateral (left and right) white matter tracts: superior cingulum bundle (CB), corticospinal tract (CST), optic radiation (OR), uncinate (UNCXL), inferior fronto-occipital fasciculus (IFOF), anterior limb of internal capsule (ALIC), inferior cingulum bundle (CBIF), and fornix (FX). These nine tracts represent a set of white matter fibers that can be reliably detected with DTI at term equivalent age due to timing and myelination, and thus, are commonly studied in neonates [63–65]. They are also known to be involved in socioemotional and neurodevelopmental functioning [48, 66, 67]. Furthermore, prior work in human children and adolescents has mostly focused on examining inflammation in relation to a narrow set of white matter tracts (e.g., CC, UNCXL) [68, 69] thus, associations between inflammation and other white matter tracts remains unclear, particularly in human neonates. Additionally, prior work in the same sample examined here found relationships between neonatal white matter tracts and disadvantage [49], and, therefore, a similar set of tracts was selected for the current study to be consistent with prior work and to extend previous findings by examining the mechanistic role of inflammation for variability in microstructure in these key tracts. We, therefore, included these tracts to determine if inflammatory associations are seen across a broader range of white matter tracts than previously noted in older youth, and to determine if these associations are related to family socioeconomic disadvantage. White matter tracts were defined using FA and FSL’s RGB V1 (primary vector) images. Referencing the FA and V1 images, seeds were placed at start-, way-, and end-points of each tract using standard anatomical landmarks in subject native space by two highly trained raters (inter-rater coefficients: 0.80–0.98 for MD and 0.73–0.92 for FA). Depending on length, size, visibility, and shape of the tract, each tract was constructed with a standard set of seeds and exclusion masks placed if necessary.

Probabilistic tractography was then completed in FSL Version 5.0.9 [70]. The diffusion tensor model was completed using FSL’s dtifit and the tensors were fitted using FSL’s bedpostx which allows for the modeling of two crossing fibers. Curvature thresholds were determined according to the shape, length, and proximity of the tract to other white matter pathways, with curvature thresholds ranging from 0.20 to 0.94 across tracts. If more than one waypoint mask was required, we forced waypoint crossing in listed order. Probtackx output files were then thresholded to retain streamlines with highest probability values indicating greater certainty of white matter. After probabilistic tractography was completed and dMRI parameters obtained, stringent quality control checks were performed by identifying any dMRI value > 2 SD above/below the mean of the distribution, and visually inspecting each Probtrakx output file. Manual intervention (e.g., seed placement, altering curvature threshold) was undertaken if the tract output image was not found to be representative of the FA and tensors on the V1 image. If the tract output image was found to be representative of the FA and tensors on the V1 image, manual intervention was not deemed necessary. As such, none of the 320 participants with dMRI data failed probabilistic tractography.

### Data analysis

All analyses were conducted in R version 4.2.1 [71]. Data were examined for distributions and outliers, and extreme dMRI and cytokine outliers (>3 SD from the mean) were removed from analysis. This included two FA values (1 CC, 1 CB), five MD values (3 CC, 1 CST, 1 FX), six AD values (4 CC, 1 OR, 1 ALIC), five RD values (2 CC, 2 CST, 1 FX), two IL-6 values, three IL-10 values, and three TNF-α values. Maternal cytokine data were normalized with log_10_ transformations to account for skewed and kurtotic distributions and were labeled based on the estimated weeks of gestation at the time of blood draw (trimester 1: <14 weeks; trimester 2: ≥14 and <27 weeks; trimester 3: ≥27 and ≤40 weeks). For each cytokine, we calculated the average concentration across gestational blood draws. For 32.8% of cases, values were available from blood draws in all three trimesters, and for the remaining 84.4% of the sample values were available from two trimesters. Trimester-specific effects were not analyzed as a primary aim of the study, given the variability in both blood collection by gestational age and sample size (trimester 1: 72% of sample, *n* = 231, SD = 2.98; trimester 2: 85% of sample, *n* = 272, SD = 4.03; trimester 3: 87% of sample, *n* = 279, SD = 3.13). However, these analyses are included in the Supplementary Information (Tables S4 and S5).

The relationships between family SES group (very low, lower-to-higher), maternal cytokine concentrations during pregnancy, and neonatal dMRI metrics (FA, MD, AD, and RD) were examined separately with multiple linear regression using the “lm” function in the R package “stats” [71]. Covariates for the SES group and maternal cytokine analysis included maternal age and maternal pre-pregnancy body mass index (BMI). Covariates for the SES group, neonatal dMRI, and maternal cytokine analyses included infant sex, NICU stay >7 days, gestational age at delivery, and infant PMA at scan. Next, moderation analyses were conducted to investigate the interaction between maternal cytokine concentration and family SES group on neonatal white matter microstructure. Linear models were fit using the “lm” function in the R package “stats” [71]. Significant interactions were probed by calculating the estimated marginal means of dMRI metric at different levels of maternal cytokine concentration (-1 SD, mean, +1 SD) by family SES group (very low, lower-to-higher) with simple slope analysis using the R function “emtrends” in the “emmeans” package [72]. Interactions were visualized with the “probe_interaction” function in the R package “interactions” [73]. Benjamini-Hochberg False Discovery Rate (FDR) procedures were used to correct for multiple comparisons by cytokine of interest and all white matter tracts (36 corrections) for each dMRI metric. In total, we ran 36 linear regression models to investigate the relationship between family SES group and neonatal white matter microstructure, 144 linear regression models to investigate the relationship between maternal cytokine concentration and neonatal white matter microstructure (four diffusion measures across nine tracts for four cytokines), and 144 moderation models to investigate the interacting effect between maternal cytokine concentration and family SES group on neonatal white matter microstructure (four diffusion measures across nine tracts for four cytokines). Standardized coefficients (β), standard errors, uncorrected *p*-values, and FDR-corrected significance values (*q*) are reported.

Supplementary Information includes analyses using continuous INR, sensitivity analyses excluding neonates born <34 weeks’ gestation and <2000 grams, and estimates of within-group variance for all maternal cytokine levels and neonatal dMRI parameters.

## Results

Sample characteristics are presented in Table 1.

**Table 1:** Sample characteristics.

	*N*	Mean	SD	Range
Infant characteristics				
Gestational age, weeks^a^	320	37.99	1.86	28–41
Birthweight, grams	320	3165.85	572.16	1310–4627
Sex assigned at birth, % (*n*)	320			
Female	–	44 (140)	–	–
Male	–	56 (180)	–	–
Race, % (*n*)^b^	320			
Black/African American	–	61.6 (197)	–	–
White/Caucasian	–	23.9 (118)	–	–
Asian	–	1.9 (6)	–	–
Native Hawaiian/Pacific Islander	–	0.3 (1)	–	–
Other (not defined)	–	0.6 (2)	–	–
Ethnicity, % (*n*)	320			
Hispanic or Latino/a	–	2.5 (8)	–	–
Not Hispanic or Latino/a	–	96.9 (310)	–	–
Unspecified	–	0.6 (2)	–	–
Postmenstrual age at MRI scan, weeks	320	41.2	1.47	37-45

Maternal characteristics				
Age at delivery, years	320	29.27	5.29	19–42
Tobacco use during pregnancy, % (*n*)	320	11.6 (37)	–	–
Cannabis use during pregnancy and/or positive urine drug screen, % (*n*)	320	10.9 (35)	–	–
Income-to-needs ratio	311	2.80	2.97	0.32–12.48
Family SES group	311			
Lower, % (*n*)	–	63 (196)	–	–
Higher, % (*n*)	–	37 (115)	–	–

All trimester-specific effects are included in the Supplementary Information material (Tables S4 and S5).

^a^Premature neonates included: *n* = 45 (3 very preterm [<32 weeks’ GA]; 4 moderate preterm [32–33 weeks’ GA]; 38 late preterm [34–36 weeks’ GA]).

^b^More than one race reported for four infants (African American-Caucasian = 2; Caucasian-Asian = 2).

### Socioeconomic status

**Fig. 1:**
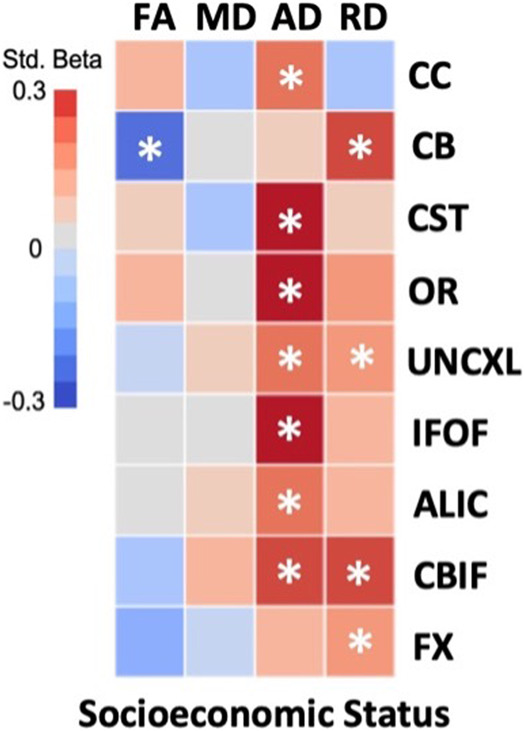
Multiple linear regression heatmap of relationship between family socioeconomic status and neonatal white matter tract dMRI parameters. Colors represent standardized beta values. Red = positive relationship. Blue = negative relationship. Covariates in models are child sex, gestational age at delivery, and infant postmenstrual age at scan. FA fractional anisotropy; MD mean diffusivity; AD axial diffusivity; RD radial diffusivity; CC corpus callosum; CB superior cingulum bundle; CST corticospinal tract; OR optic radiation; UNCXL uncinate fasciculus; IFOF inferior fronto-occipital fasciculus; ALIC anterior limb of internal capsule; CBIF inferior longitudinal fasciculus; FX fornix; SES socioeconomic status; *, significant after FDR correction for multiple comparisons.

Mothers in the very low SES group had significantly higher average IL-6 than mothers in the lower-to-higher SES group (*β* = 0.24; *q* < 0.001). There were no significant associations between family SES group and average maternal IL-8 (*β* = −0.02; *q* = 0.90), IL-10 (*β* = 0.06; *q* = 0.63), or TNF-α (*β* = −0.01; *q* = 0.90) concentrations during pregnancy. In relation to white matter microstructure, neonates in the very low SES group had significantly lower (1) CC AD; (2) CB RD; (3) CST AD; (4) OR AD; (5) UNCXL AD and RD; (6) IFOF AD; (7) ALIC AD; (8) CBIF AD and RD; and (9) FX RD. Neonates in the very low SES group displayed significantly higher CB FA (Fig. 1 and Table 2).

**Table 2:** Associations between family socioeconomic status group and neonatal dMRI parameters.

	FA				MD				AD				RD			
	*β*	SE	*p*	*q*	*β*	SE	*p*	*q*	*β*	SE	*p*	*q*	*β*	SE	*p*	*q*
Corpus callosum																
SES group	0.096	0.003	0.05	0.14	−0.048	0.004	0.42	0.59	0.146	0.003	0.01	**0.03**	−0.050	0.004	0.29	0.45
Sex	−0.102	0.003	0.04	0.20	−0.058	0.004	0.32	0.64	−0.087	0.003	0.12	0.30	0.049	0.004	0.29	0.62
NICU stay	−0.106	0.007	0.08	0.70	−0.065	0.008	0.36	0.70	−0.007	0.007	0.92	0.96	0.033	0.010	0.58	0.83
GA at delivery	0.001	0.001	0.99	0.99	−0.018	0.001	0.81	0.99	0.105	0.001	0.17	0.74	−0.010	0.002	0.88	0.99
PMA at scan	0.462	0.001	<0.001	**<0.001**	−0.131	0.001	0.04	**0.04**	−0.229	0.001	<0.001	**<0.001**	−0.566	0.002	<0.001	**<0.001**
Superior cingulum bundle																
SES group	−0.147	0.003	0.01	0.02	0.004	0.005	0.94	0.94	0.047	0.005	0.38	0.55	0.170	0.005	0.001	**<0.01**
Sex	−0.104	0.003	0.05	0.22	0.014	0.005	0.815	0.95	−0.119	0.005	0.02	0.20	0.018	0.005	0.72	0.93
NICU stay	−0.057	0.006	0.39	0.71	−0.148	0.011	0.04	0.44	0.037	0.011	0.57	0.83	0.079	0.011	0.20	0.70
GA at delivery	0.045	0.001	0.53	0.99	−0.037	0.002	0.63	0.99	0.089	0.002	0.22	0.74	0.029	0.002	0.66	0.99
PMA at scan	0.354	0.001	<0.001	**<0.001**	−0.127	0.002	0.04	**0.04**	−0.417	0.002	<0.001	**<0.001**	−0.520	0.002	<0.001	**<0.001**
Corticospinal tract																
SES group	0.056	0.003	0.25	0.40	−0.053	0.006	0.37	0.55	0.222	0.003	<0.001	**<0.01**	0.055	0.005	0.20	0.36
Sex	0.003	0.003	0.96	0.98	0.021	0.005	0.72	0.93	−0.071	0.003	0.10	0.29	−0.033	0.005	0.44	0.79
NICU stay	−0.031	0.006	0.61	0.83	−0.076	0.012	0.28	0.70	0.027	0.007	0.62	0.83	0.020	0.011	0.70	0.87
GA at delivery	0.020	0.001	0.76	0.99	0.002	0.002	0.98	0.99	0.022	0.001	0.71	0.99	0.006	0.002	0.91	0.99
PMA at scan	0.544	0.001	<0.001	**<0.001**	−0.182	0.002	<0.001	**<0.001**	−0.646	0.001	<0.001	**<0.001**	−0.688	0.002	<0.001	**<0.001**
Optic radiation																
SES group	0.080	0.003	0.09	0.18	0.026	0.006	<0.001	0.66	0.291	0.005	<0.001	**<0.01**	0.102	0.006	0.02	0.05
Sex	−0.013	0.003	0.78	0.93	−0.016	0.006	0.00	0.778	−0.204	0.005	<0.001	**<0.01**	−0.088	0.005	0.04	0.20
NICU stay	−0.161	0.007	0.01	0.21	−0.077	0.014	0.09	0.27	−0.003	0.010	0.96	0.96	0.122	0.012	0.02	0.43
GA at delivery	−0.025	0.001	0.69	0.99	−0.013	0.002	0.27	0.87	−0.091	0.002	0.19	0.74	−0.046	0.002	0.43	0.96
PMA at scan	0.544	0.001	<0.001	**<0.001**	−0.153	0.002	<0.001	**0.013**	−0.324	0.002	<0.001	**<0.001**	−0.618	0.002	<0.001	**<0.001**
Uncinate fasciculus																
SES group	−0.017	0.003	0.72	0.79	0.035	0.005	0.56	0.70	0.144	0.005	0.01	**0.03**	0.116	0.004	0.01	**0.03**
Sex	−0.083	0.002	0.08	0.26	0.007	0.005	0.90	0.98	−0.120	0.005	0.03	0.20	−0.014	0.004	0.76	0.93
NICU stay	−0.057	0.005	0.33	0.70	0.021	0.010	0.77	0.92	−0.087	0.011	0.21	0.70	−0.004	0.009	0.94	0.96
GA at delivery	0.092	0.001	0.15	0.74	0.092	0.002	0.23	0.74	0.125	0.002	0.09	0.74	0.005	0.002	0.93	0.99
PMA at scan	0.511	0.001	<0.001	**<0.001**	−0.094	0.002	0.13	0.14	−0.294	0.002	<0.001	**<0.001**	−0.632	0.002	<0.001	**<0.001**
Inferior fronto-occipital fasciculus																
SES group	0.016	0.003	0.70	0.79	0.005	0.006	0.94	0.94	0.229	0.004	<0.001	**<0.01**	0.077	0.006	0.07	0.16
Sex	0.001	0.003	0.98	0.98	−0.010	0.006	0.870	0.98	−0.167	0.003	0.001	**0.02**	−0.070	0.005	0.09	0.26
NICU stay	−0.072	0.006	0.18	0.70	−0.064	0.014	0.37	0.70	0.026	0.008	0.68	0.87	0.059	0.012	0.25	0.70
GA at delivery	0.085	0.001	0.14	0.74	−0.046	0.002	0.54	0.99	0.075	0.001	0.27	0.74	−0.054	0.002	0.34	0.87
PMA at scan	0.617	0.001	<0.001	**<0.001**	−0.100	0.002	0.11	0.12	−0.469	0.001	<0.001	**<0.001**	−0.668	0.002	<0.001	**<0.001**
Anterior limb of internal capsule																
SES group	0.022	0.003	0.61	0.73	0.035	0.007	0.55	0.70	0.154	0.005	0.004	**0.02**	0.075	0.006	0.09	0.19
Sex	−0.031	0.002	0.47	0.80	−0.030	0.007	0.61	0.93	−0.055	0.005	0.28	0.62	−0.021	0.006	0.63	0.93
NICU stay	−0.056	0.005	0.29	0.70	−0.081	0.015	0.25	0.70	0.032	0.010	0.62	0.83	0.059	0.014	0.29	0.70
GA at delivery	0.121	0.001	0.04	0.74	−0.064	0.003	0.40	0.96	0.083	0.002	0.24	0.74	−0.044	0.002	0.46	0.98
PMA at scan	0.597	0.001	<0.001	**<0.001**	−0.078	0.003	0.21	0.21	−0.460	0.002	<0.001	**<0.001**	−0.621	0.002	<0.001	**<0.001**
Inferior cingulum bundle																
SES group	−0.033	0.002	0.53	0.70	0.071	0.003	0.22	0.38	0.196	0.003	<0.001	**<0.01**	0.171	0.003	<0.001	**<0.01**
Sex	−0.025	0.002	0.63	0.93	0.024	0.003	0.68	0.93	−0.121	0.003	0.02	0.20	−0.086	0.003	0.05	0.22
NICU stay	−0.104	0.005	0.12	0.70	−0.010	0.007	0.88	0.96	−0.036	0.007	0.59	0.83	0.076	0.006	0.17	0.70
GA at delivery	−0.115	0.001	0.11	0.74	0.085	0.001	0.26	0.74	−0.020	0.001	0.78	0.99	0.110	0.001	0.07	0.74
PMA at scan	0.441	0.001	<0.001	**<0.001**	−0.224	0.001	<0.001	**<0.001**	−0.332	0.001	<0.001	**<0.001**	−0.658	0.001	<0.001	**<0.001**
Fornix																
SES group	−0.096	0.002	0.07	0.17	−0.019	0.004	0.75	0.79	0.076	0.004	0.14	0.26	0.124	0.003	0.01	**0.02**
Sex	−0.091	0.002	0.09	0.26	0.047	0.004	0.42	0.79	−0.078	0.004	0.12	0.30	0.003	0.003	0.94	0.98
NICU stay	−0.016	0.004	0.81	0.94	−0.063	0.008	0.37	0.70	0.011	0.008	0.87	0.96	0.029	0.006	0.60	0.83
GA at delivery	0.031	0.001	0.67	0.99	−0.018	0.001	0.82	0.99	0.041	0.001	0.55	0.99	0.008	0.001	0.90	0.99
PMA at scan	0.363	0.001	<0.001	**<0.001**	−0.169	0.001	<0.01	**0.01**	−0.503	0.001	<0.001	**<0.001**	−0.653	0.001	<0.001	**<0.001**

Multiple linear regression results of the association between family socioeconomic status group and neonatal dMRI parameters. Covariates in models are child sex, NICU stay > 7 days, gestational age at delivery, and infant postmenstrual age at scan. Bolded values represent statistical significance after FDR correction for multiple comparisons.

*β* standardized beta coefficient, *SE* standard error, *q* FDR-corrected p-value, *FA* fractional anisotropy, *MD* mean diffusivity, *AD* axial diffusivity; *RD* radial diffusivity, *GA* gestational age, *PMA* infant postmenstrual age, *Sex* child sex.

### Maternal cytokine concentration and neonatal dMRI parameters

**Fig. 2:**
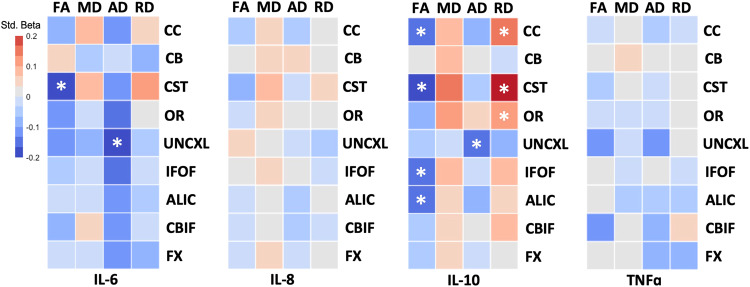
Multiple linear regression heatmap of relationship between average maternal cytokine concentration and neonatal white matter tract dMRI parameters. Colors represent standardized beta values. Covariates in models are child sex, NICU stay, gestational age at delivery, infant postmenstrual age at scan, and maternal BMI pre-pregnancy. FA fractional anisotropy; MD mean diffusivity; AD axial diffusivity; RD radial diffusivity; CC corpus callosum; CB superior cingulum bundle; CST corticospinal tract; OR optic radiation; UNCXL uncinate fasciculus; IFOF inferior fronto-occipital fasciculus; ALIC anterior limb of internal capsule; CBIF inferior longitudinal fasciculus; FX fornix; IL interleukin; *, significant after FDR correction for multiple comparisons.

Average maternal IL-6 concentration was negatively associated with CST FA and UNCXL. Average maternal IL-10 concentration was 1) negatively associated with CC, CST, IFOF, and ALIC FA and UNCXL AD and 2) positively associated with CC, CST, and OR RD. There were no significant associations between average maternal IL-8 or TNF-α concentrations and neonatal dMRI measures (Fig. 2 and Table 3).

**Table 3:** Associations between maternal cytokine concentrations and neonatal dMRI parameters.

IL-6																
	FA				MD				AD				RD			
	*β*	SE	*p*	*q*	*β*	SE	*p*	*q*	*β*	SE	*p*	*q*	*β*	SE	*p*	*q*
Corpus callosum																
IL-6	−0.083	0.002	0.10	0.23	0.092	0.002	0.12	0.25	−0.108	0.002	0.06	0.18	0.044	0.002	0.36	0.51
Sex	−0.084	0.003	0.09	0.32	−0.061	0.004	0.29	0.65	−0.074	0.003	0.19	0.46	0.036	0.004	0.44	0.88
NICU stay	−0.099	0.007	0.12	0.63	−0.069	0.008	0.34	0.63	0.021	0.008	0.77	1.00	0.030	0.010	0.63	0.90
GA at delivery	0.014	0.001	0.84	0.89	−0.004	0.001	0.95	0.95	0.119	0.001	0.13	0.73	−0.026	0.002	0.70	0.89
PMA at scan	0.450	0.001	<0.001	**<0.001**	−0.155	0.001	0.01	**0.01**	−0.227	0.001	<0.001	**<0.001**	−0.555	0.002	<0.001	**<0.001**
Maternal BMI	0.054	0.000	0.28	0.56	0.086	0.000	0.14	0.45	0.065	0.000	0.26	0.56	−0.035	0.000	0.47	0.66
Superior cingulum bundle																
IL-6	0.062	0.002	0.25	0.38	−0.049	0.003	0.40	0.55	−0.033	0.003	0.54	0.72	−0.082	0.003	0.10	0.23
Sex	−0.099	0.003	0.06	0.32	0.011	0.005	0.85	0.98	−0.117	0.005	0.03	0.24	0.014	0.005	0.77	0.98
NICU stay	−0.052	0.006	0.45	0.71	−0.161	0.011	0.03	0.32	0.066	0.011	0.33	0.63	0.097	0.011	0.13	0.63
GA at delivery	0.030	0.001	0.68	0.89	−0.050	0.002	0.51	0.89	0.087	0.002	0.24	0.87	0.042	0.002	0.54	0.89
PMA at scan	0.346	0.001	<0.001	**<0.001**	−0.127	0.002	0.04	**0.04**	−0.414	0.002	**<0.001**	**<0.001**	−0.511	0.002	**<0.001**	**<0.001**
Maternal BMI	−0.050	0.000	0.35	0.63	0.048	0.000	0.41	0.64	−0.024	0.000	0.65	0.78	0.021	0.000	0.67	0.78
Corticospinal tract																
IL-6	−0.212	0.002	<0.01	**<0.01**	0.089	0.003	0.12	0.25	−0.110	0.002	0.01	0.07	0.105	0.003	0.01	0.07
Sex	−0.005	0.003	0.91	0.98	0.005	0.005	0.92	0.98	−0.074	0.003	0.10	0.32	−0.025	0.005	0.55	0.91
NICU stay	0.016	0.006	0.79	1.00	−0.076	0.012	0.29	0.63	0.059	0.008	0.31	0.63	−0.004	0.011	0.94	1.00
GA at delivery	0.028	0.001	0.66	0.89	0.018	0.002	0.81	0.89	0.051	0.001	0.41	0.89	0.009	0.002	0.87	0.90
PMA at scan	0.521	0.001	<0.001	**<0.001**	−0.212	0.002	<0.001	**<0.001**	−0.646	0.001	<0.001	**<0.001**	−0.668	0.002	<0.001	**<0.001**
Maternal BMI	0.032	0.000	0.50	0.66	0.064	0.000	0.27	0.56	0.031	0.000	0.49	0.66	−0.008	0.000	0.85	0.85
Optic radiation																
IL-6	−0.114	0.002	0.02	0.07	−0.011	0.003	0.85	0.92	−0.139	0.003	0.01	0.07	0.004	0.003	0.93	0.94
Sex	−0.022	0.003	0.63	0.91	−0.010	0.006	0.86	0.98	−0.185	0.005	<0.01	**0.02**	−0.073	0.005	0.09	0.32
NICU stay	−0.152	0.007	0.01	0.32	−0.068	0.014	0.34	0.63	0.019	0.011	0.78	1.00	0.127	0.012	0.02	0.32
GA at delivery	−0.029	0.001	0.65	0.89	0.019	0.002	0.80	0.89	−0.033	0.002	0.65	0.89	−0.013	0.002	0.82	0.89
PMA at scan	0.542	0.001	<0.001	**<0.001**	−0.175	0.002	<0.01	**0.01**	−0.327	0.002	<0.001	**<0.001**	−0.621	0.002	<0.001	**<0.001**
Maternal BMI	0.013	0.000	0.78	0.80	0.142	0.000	0.02	0.14	0.164	0.000	<0.01	0.08	0.083	0.000	0.06	0.27
Uncinate fasciculus																
IL-6	−0.122	0.001	0.01	0.07	−0.072	0.003	0.22	0.36	−0.217	0.003	<0.001	**<0.001**	−0.065	0.002	0.16	0.29
Sex	−0.073	0.002	0.12	0.34	0.003	0.005	0.96	0.98	−0.123	0.005	0.02	0.24	−0.026	0.004	0.56	0.91
NICU stay	−0.055	0.005	0.37	0.63	−0.002	0.011	0.97	1.00	−0.082	0.011	0.24	0.63	−0.004	0.010	0.95	1.00
GA at delivery	0.070	0.001	0.28	0.89	0.053	0.002	0.49	0.89	0.122	0.002	0.10	0.73	0.024	0.002	0.70	0.89
PMA at scan	0.501	0.001	<0.001	**<0.001**	−0.097	0.002	0.12	0.12	−0.303	0.002	<0.001	**<0.001**	−0.627	0.002	<0.001	**<0.001**
Maternal BMI	0.039	0.000	0.41	0.64	0.084	0.000	0.15	0.45	0.093	0.000	0.09	0.31	0.048	0.000	0.30	0.56
Inferior fronto-occipital fasciculus																
IL-6	−0.053	0.002	0.21	0.36	−0.015	0.003	0.80	0.92	−0.143	0.002	0.01	0.06	−0.021	0.003	0.62	0.77
Sex	−0.006	0.003	0.89	0.98	−0.015	0.006	0.80	0.98	−0.167	0.003	<0.01	**0.02**	−0.067	0.005	0.11	0.32
NICU stay	−0.075	0.006	0.17	0.63	−0.067	0.014	0.36	0.63	0.044	0.008	0.50	0.76	0.069	0.012	0.19	0.63
GA at delivery	0.069	0.001	0.24	0.87	−0.026	0.002	0.73	0.89	0.098	0.001	0.16	0.73	−0.030	0.002	0.60	0.89
PMA at scan	0.620	0.001	<0.001	**<0.001**	−0.131	0.002	0.04	**0.04**	−0.460	0.001	<0.001	**<0.001**	−0.672	0.002	<0.001	**<0.001**
Maternal BMI	0.017	0.000	0.69	0.78	0.126	0.000	0.03	0.20	0.121	0.000	0.02	0.14	0.038	0.000	0.37	0.63
Anterior limb of internal capsule																
IL-6	−0.009	0.001	0.83	0.92	−0.004	0.004	0.94	0.94	−0.120	0.003	0.02	0.09	−0.062	0.003	0.16	0.30
Sex	−0.025	0.002	0.56	0.91	−0.028	0.007	0.63	0.91	−0.053	0.005	0.31	0.66	−0.023	0.006	0.59	0.91
NICU stay	−0.045	0.005	0.42	0.68	−0.080	0.016	0.27	0.63	0.076	0.011	0.26	0.63	0.074	0.014	0.19	0.63
GA at delivery	0.124	0.001	0.04	0.69	−0.034	0.003	0.66	0.89	0.103	0.002	0.15	0.73	−0.030	0.002	0.62	0.89
PMA at scan	0.595	0.001	<0.001	**<0.001**	−0.100	0.003	0.11	0.11	−0.455	0.002	<0.001	**<0.001**	−0.620	0.002	<0.001	**<0.001**
Maternal BMI	0.019	0.000	0.67	0.78	0.137	0.000	0.02	0.14	0.021	0.000	0.69	0.78	0.016	0.000	0.73	0.78
Inferior cingulum bundle																
IL-6	−0.094	0.001	0.07	0.19	0.062	0.002	0.28	0.42	−0.122	0.002	0.02	0.09	−0.017	0.002	0.71	0.85
Sex	0.001	0.002	0.98	0.98	0.019	0.003	0.73	0.98	−0.100	0.003	0.07	0.32	−0.093	0.003	0.04	0.31
NICU stay	−0.097	0.005	0.15	0.63	0.000	0.007	1.00	1.00	−0.011	0.008	0.87	1.00	0.087	0.006	0.14	0.63
GA at delivery	−0.116	0.001	0.11	0.73	0.118	0.001	0.12	0.73	0.024	0.001	0.75	0.89	0.145	0.001	0.02	0.69
PMA at scan	0.429	0.001	<0.001	**<0.001**	−0.233	0.001	<0.001	**<0.001**	−0.321	0.001	<0.001	**<0.001**	−0.644	0.001	<0.001	**<0.001**
Maternal BMI	0.071	0.000	0.18	0.47	0.112	0.000	0.05	0.27	0.131	0.000	0.02	0.14	0.052	0.000	0.26	0.56
Fornix																
IL-6	−0.006	0.001	0.92	0.94	−0.030	0.002	0.61	0.77	−0.101	0.002	0.05	0.15	−0.082	0.002	0.06	0.18
Sex	−0.086	0.002	0.11	0.32	0.032	0.003	0.58	0.91	−0.073	0.004	0.15	0.37	0.003	0.003	0.95	0.98
NICU stay	0.003	0.004	0.96	1.00	−0.078	0.008	0.28	0.63	0.006	0.008	0.93	1.00	0.014	0.006	0.81	1.00
GA at delivery	0.029	0.001	0.69	0.89	−0.023	0.001	0.77	0.89	0.067	0.001	0.34	0.89	0.028	0.001	0.65	0.89
PMA at scan	0.354	0.001	<0.001	**<0.001**	−0.191	0.001	<0.001	**<0.001**	−0.514	0.001	<0.001	**<0.001**	−0.649	0.001	<0.001	**<0.001**
Maternal BMI	−0.041	0.000	0.45	0.66	0.020	0.000	0.74	0.78	0.071	0.000	0.16	0.45	0.079	0.000	0.08	0.31

IL-8																
Corpus callosum																
IL-8	−0.039	0.002	0.44	0.97	0.038	0.002	0.51	0.97	−0.055	0.002	0.33	0.97	0.019	0.003	0.69	0.97
Sex	−0.084	0.003	0.09	0.27	−0.056	0.004	0.34	0.71	−0.086	0.003	0.13	0.34	0.031	0.004	0.52	0.89
NICU stay	−0.098	0.007	0.12	0.64	−0.071	0.008	0.33	0.64	0.023	0.008	0.75	0.96	0.031	0.010	0.62	0.89
GA at delivery	0.018	0.001	0.79	0.93	−0.020	0.001	0.80	0.93	0.142	0.001	0.07	0.53	−0.022	0.002	0.74	0.93
PMA at scan	0.450	0.001	<0.001	**<0.001**	−0.158	0.001	0.01	**0.01**	−0.224	0.001	<0.001	**<0.001**	−0.554	0.002	<0.001	**<0.001**
Maternal BMI	0.053	0.000	0.30	0.54	0.085	0.000	0.15	0.44	0.074	0.000	0.19	0.47	−0.029	0.000	0.55	0.75
Superior cingulum bundle																
IL-8	0.001	0.002	0.99	0.99	0.064	0.003	0.27	0.97	0.036	0.003	0.49	0.97	0.029	0.003	0.56	0.97
Sex	−0.097	0.003	0.07	0.24	0.013	0.005	0.82	0.97	−0.123	0.005	0.02	0.18	0.008	0.005	0.87	0.97
NICU stay	−0.055	0.006	0.43	0.67	−0.159	0.011	0.03	0.32	0.066	0.011	0.33	0.64	0.100	0.011	0.12	0.64
GA at delivery	0.019	0.001	0.80	0.93	−0.036	0.002	0.64	0.93	0.103	0.002	0.16	0.64	0.064	0.002	0.35	0.91
PMA at scan	0.342	0.001	<0.001	**<0.001**	−0.125	0.002	0.04	0.05	−0.411	0.002	<0.001	**<0.001**	−0.504	0.002	<0.001	**<0.001**
Maternal BMI	−0.050	0.000	0.36	0.62	0.043	0.000	0.46	0.71	−0.017	0.000	0.75	0.85	0.025	0.000	0.63	0.76
Corticospinal tract																
IL-8	−0.071	0.002	0.14	0.97	0.076	0.003	0.19	0.97	−0.006	0.002	0.89	0.99	0.062	0.003	0.15	0.97
Sex	−0.005	0.003	0.91	0.97	0.010	0.005	0.87	0.97	−0.085	0.003	0.06	0.24	−0.029	0.005	0.49	0.89
NICU stay	0.020	0.007	0.74	0.96	−0.078	0.012	0.28	0.64	0.062	0.008	0.29	0.64	−0.006	0.011	0.91	0.99
GA at delivery	0.048	0.001	0.47	0.93	0.009	0.002	0.91	0.96	0.078	0.001	0.22	0.64	0.010	0.002	0.87	0.96
PMA at scan	0.524	0.001	<0.001	**<0.001**	−0.212	0.002	<0.001	**<0.001**	−0.636	0.001	<0.001	**<0.001**	−0.666	0.002	<0.001	**<0.001**
Maternal BMI	0.027	0.000	0.57	0.75	0.064	0.000	0.27	0.54	0.039	0.000	0.39	0.64	−0.001	0.000	0.98	0.98
Optic radiation																
IL-8	−0.021	0.002	0.66	0.97	0.043	0.004	0.45	0.97	0.004	0.003	0.94	0.99	0.030	0.003	0.49	0.97
Sex	−0.020	0.003	0.66	0.95	−0.007	0.006	0.91	0.97	−0.192	0.005	<0.01	**0.01**	−0.079	0.005	0.07	0.24
NICU stay	−0.150	0.007	0.01	0.32	−0.068	0.014	0.34	0.64	0.024	0.011	0.73	0.96	0.127	0.012	0.02	0.32
GA at delivery	−0.019	0.001	0.77	0.93	0.023	0.002	0.76	0.93	−0.004	0.002	0.96	0.96	−0.004	0.002	0.95	0.96
PMA at scan	0.544	0.001	<0.001	**<0.001**	−0.174	0.002	<0.001	**<0.001**	−0.318	0.002	<0.001	**<0.001**	−0.618	0.002	<0.001	**<0.001**
Maternal BMI	0.008	0.000	0.86	0.89	0.137	0.000	0.02	0.16	0.166	0.000	<0.01	0.07	0.088	0.000	0.05	0.24
Uncinate fasciculus																
IL-8	0.058	0.001	0.22	0.97	0.017	0.003	0.77	0.99	−0.028	0.003	0.61	0.97	−0.066	0.003	0.15	0.97
Sex	−0.068	0.002	0.15	0.36	0.006	0.005	0.92	0.97	−0.129	0.005	0.02	0.18	−0.034	0.004	0.46	0.89
NICU stay	−0.054	0.005	0.38	0.65	−0.001	0.011	0.99	0.99	−0.076	0.011	0.28	0.64	0.000	0.010	0.99	0.99
GA at delivery	0.088	0.001	0.18	0.64	0.064	0.002	0.41	0.93	0.154	0.002	0.04	0.52	0.034	0.002	0.59	0.93
PMA at scan	0.503	0.001	<0.001	**<0.001**	−0.095	0.002	0.13	0.13	−0.298	0.002	<0.001	**<0.001**	−0.625	0.002	<0.001	**<0.001**
Maternal BMI	0.034	0.000	0.49	0.73	0.078	0.000	0.19	0.47	0.093	0.000	0.10	0.36	0.053	0.000	0.26	0.54
Inferior fronto-occipital fasciculus																
IL-8	0.011	0.002	0.80	0.99	0.049	0.004	0.40	0.97	0.000	0.002	0.99	0.99	−0.010	0.003	0.81	0.99
Sex	0.000	0.003	1.00	1.00	−0.011	0.006	0.85	0.97	−0.173	0.004	<0.01	**0.01**	−0.074	0.005	0.07	0.24
NICU stay	−0.075	0.006	0.18	0.64	−0.067	0.014	0.35	0.64	0.047	0.008	0.48	0.72	0.070	0.012	0.19	0.64
GA at delivery	0.071	0.001	0.23	0.64	−0.022	0.002	0.78	0.93	0.125	0.001	0.08	0.53	−0.020	0.002	0.72	0.93
PMA at scan	0.622	0.001	<0.001	**<0.001**	−0.130	0.002	0.04	**0.04**	−0.455	0.001	<0.001	**<0.001**	−0.671	0.002	<0.001	**<0.001**
Maternal BMI	0.010	0.000	0.81	0.86	0.121	0.000	0.04	0.23	0.124	0.000	0.02	0.16	0.045	0.000	0.29	0.54
Anterior limb of internal capsule																
IL-8	−0.031	0.001	0.47	0.97	0.031	0.004	0.59	0.97	−0.056	0.003	0.28	0.97	0.005	0.004	0.91	0.99
Sex	−0.026	0.002	0.54	0.89	−0.024	0.007	0.68	0.95	−0.063	0.005	0.23	0.51	−0.028	0.006	0.53	0.89
NICU stay	−0.046	0.005	0.41	0.67	−0.080	0.016	0.26	0.64	0.079	0.011	0.25	0.64	0.076	0.014	0.19	0.64
GA at delivery	0.123	0.001	0.04	0.52	−0.034	0.003	0.65	0.93	0.124	0.002	0.09	0.53	−0.016	0.002	0.79	0.93
PMA at scan	0.594	0.001	<0.001	**<0.001**	−0.102	0.003	0.10	0.10	−0.453	0.002	<0.001	**<0.001**	−0.618	0.002	<0.001	**<0.001**
Maternal BMI	0.021	0.000	0.63	0.76	0.134	0.000	0.02	0.16	0.029	0.000	0.58	0.75	0.018	0.000	0.68	0.79
Inferior cingulum bundle																
IL-8	−0.031	0.001	0.55	0.97	0.022	0.002	0.70	0.97	−0.048	0.002	0.38	0.97	−0.005	0.002	0.90	0.99
Sex	−0.001	0.002	0.98	1.00	0.023	0.003	0.69	0.95	−0.108	0.003	0.05	0.24	−0.097	0.003	0.03	0.24
NICU stay	−0.095	0.005	0.16	0.64	−0.001	0.007	0.99	0.99	−0.008	0.008	0.91	0.99	0.087	0.006	0.14	0.64
GA at delivery	−0.105	0.001	0.15	0.64	0.108	0.001	0.15	0.64	0.045	0.001	0.55	0.93	0.151	0.001	0.02	0.52
PMA at scan	0.430	0.001	<0.001	**<0.001**	−0.231	0.001	<0.001	**<0.001**	−0.319	0.001	<0.001	**<0.001**	−0.645	0.001	<0.001	**<0.001**
Maternal BMI	0.071	0.000	0.18	0.47	0.111	0.000	0.06	0.25	0.137	0.000	0.01	0.16	0.056	0.000	0.23	0.51
Fornix																
IL-8	−0.023	0.001	0.66	0.97	0.053	0.002	0.36	0.97	−0.007	0.002	0.89	0.99	0.023	0.002	0.60	0.97
Sex	−0.096	0.002	0.07	0.24	0.032	0.003	0.58	0.90	−0.081	0.004	0.11	0.30	0.006	0.003	0.89	0.97
NICU stay	0.004	0.004	0.95	0.99	−0.077	0.008	0.28	0.64	0.008	0.008	0.90	0.99	0.015	0.006	0.80	0.99
GA at delivery	0.039	0.001	0.60	0.93	−0.010	0.001	0.89	0.96	0.088	0.001	0.21	0.64	0.038	0.001	0.54	0.93
PMA at scan	0.347	0.001	<0.001	**<0.001**	−0.188	0.001	<0.001	**<0.001**	−0.510	0.001	<0.001	**<0.001**	−0.647	0.001	<0.001	**<0.001**
Maternal BMI	−0.029	0.000	0.59	0.75	0.017	0.000	0.78	0.85	0.078	0.000	0.13	0.42	0.074	0.000	0.10	0.36

IL-10																
Corpus callosum																
IL-10	−0.146	0.002	<0.02	**0.02**	0.073	0.002	0.21	0.39	−0.087	0.002	0.13	0.30	0.149	0.003	0.00	0.01
Sex	−0.079	0.003	0.11	0.33	−0.052	0.004	0.37	0.77	−0.080	0.003	0.17	0.37	0.030	0.004	0.52	0.85
NICU stay	−0.134	0.007	0.03	0.32	−0.059	0.009	0.42	0.65	0.001	0.008	0.99	0.99	0.059	0.010	0.32	0.65
GA at delivery	0.026	0.001	0.70	0.98	−0.021	0.001	0.78	0.98	0.131	0.001	0.09	0.52	−0.028	0.002	0.66	0.98
PMA at scan	0.461	0.001	<0.001	**<0.001**	−0.155	0.001	0.01	**0.01**	−0.214	0.001	<0.001	**<0.001**	−0.555	0.002	<0.001	**<0.001**
Maternal BMI	0.056	0.000	0.26	0.53	0.088	0.000	0.13	0.40	0.071	0.000	0.22	0.50	−0.033	0.000	0.49	0.70
Superior cingulum bundle																
IL-10	0.001	0.002	0.99	0.99	0.088	0.003	0.13	0.30	0.005	0.003	0.93	0.96	−0.009	0.003	0.86	0.93
Sex	−0.085	0.003	0.12	0.34	0.012	0.005	0.84	0.96	−0.149	0.005	0.01	0.06	−0.022	0.005	0.66	0.96
NICU stay	−0.047	0.006	0.51	0.65	−0.145	0.011	0.05	0.32	0.082	0.011	0.23	0.64	0.099	0.011	0.13	0.49
GA at delivery	0.006	0.001	0.94	0.98	−0.036	0.002	0.63	0.98	0.110	0.002	0.13	0.52	0.082	0.002	0.23	0.70
PMA at scan	0.342	0.001	<0.001	**<0.001**	−0.121	0.002	0.05	0.05	−0.416	0.002	<0.001	**<0.001**	−0.512	0.002	<0.001	**<0.001**
Maternal BMI	−0.045	0.000	0.41	0.70	0.054	0.000	0.36	0.64	0.004	0.000	0.94	0.95	0.037	0.000	0.47	0.70
Corticospinal tract																
IL-10	−0.301	0.002	0.00	0.00	0.140	0.003	0.01	0.06	−0.038	0.002	0.42	0.60	0.217	0.003	<0.001	**<0.001**
Sex	−0.011	0.003	0.80	0.96	0.009	0.005	0.87	0.96	−0.092	0.003	0.05	0.27	−0.029	0.005	0.49	0.83
NICU stay	−0.048	0.006	0.40	0.65	−0.057	0.012	0.42	0.65	0.054	0.008	0.36	0.65	0.036	0.011	0.49	0.65
GA at delivery	0.052	0.001	0.40	0.89	0.005	0.002	0.95	0.98	0.085	0.001	0.18	0.58	0.001	0.002	0.98	0.98
PMA at scan	0.525	0.001	<0.001	**<0.001**	−0.203	0.002	<0.001	**<0.001**	−0.630	0.001	<0.001	**<0.001**	−0.658	0.002	<0.001	**<0.001**
Maternal BMI	0.015	0.000	0.74	0.82	0.079	0.000	0.17	0.44	0.026	0.000	0.58	0.72	0.003	0.000	0.95	0.95
Optic radiation																
IL-10	−0.098	0.002	0.04	0.13	0.109	0.004	0.06	0.17	0.065	0.003	0.23	0.39	0.114	0.003	0.01	0.05
Sex	−0.013	0.003	0.79	0.96	−0.006	0.006	0.92	0.98	−0.177	0.005	<0.01	**0.02**	−0.077	0.005	0.08	0.27
NICU stay	−0.178	0.007	<0.01	0.07	−0.053	0.014	0.46	0.65	0.046	0.011	0.50	0.65	0.160	0.012	<0.01	0.07
GA at delivery	−0.024	0.001	0.70	0.98	0.021	0.002	0.78	0.98	−0.012	0.002	0.87	0.98	−0.003	0.002	0.96	0.98
PMA at scan	0.547	0.001	<0.001	**<0.001**	−0.170	0.002	0.01	**0.01**	−0.327	0.002	<0.001	**<0.001**	−0.619	0.002	<0.001	**<0.001**
Maternal BMI	0.009	0.000	0.85	0.90	0.145	0.000	0.01	0.17	0.148	0.000	0.01	0.17	0.078	0.000	0.08	0.34
Uncinate fasciculus																
IL-10	−0.037	0.001	0.46	0.61	−0.023	0.003	0.69	0.78	−0.142	0.003	0.01	0.05	−0.089	0.003	0.06	0.17
Sex	−0.071	0.002	0.15	0.35	0.003	0.005	0.96	0.98	−0.138	0.005	0.01	0.11	−0.041	0.004	0.39	0.77
NICU stay	−0.063	0.006	0.32	0.65	−0.002	0.011	0.98	0.99	−0.091	0.012	0.20	0.59	−0.010	0.010	0.87	0.99
GA at delivery	0.076	0.001	0.25	0.70	0.063	0.002	0.42	0.89	0.159	0.002	0.03	0.45	0.049	0.002	0.44	0.89
PMA at scan	0.492	0.001	<0.001	**<0.001**	−0.090	0.002	0.15	0.15	−0.297	0.002	<0.001	**<0.001**	−0.618	0.002	<0.001	**<0.001**
Maternal BMI	0.035	0.000	0.48	0.70	0.085	0.000	0.15	0.42	0.087	0.000	0.12	0.40	0.048	0.000	0.31	0.58
Inferior fronto-occipital fasciculus																
IL-10	−0.139	0.002	<0.01	**0.01**	0.084	0.004	0.15	0.31	−0.030	0.002	0.57	0.67	0.098	0.003	0.02	0.07
Sex	−0.001	0.003	0.98	0.98	−0.009	0.006	0.87	0.96	−0.181	0.004	<0.01	**0.02**	−0.077	0.005	0.06	0.27
NICU stay	−0.112	0.006	0.04	0.32	−0.054	0.014	0.46	0.65	0.052	0.008	0.43	0.65	0.103	0.012	0.05	0.32
GA at delivery	0.060	0.001	0.29	0.75	−0.025	0.002	0.74	0.98	0.125	0.001	0.08	0.52	−0.011	0.002	0.85	0.98
PMA at scan	0.627	0.001	<0.001	**<0.001**	−0.124	0.002	0.05	0.05	−0.451	0.001	<0.001	**<0.001**	−0.674	0.002	<0.001	**<0.001**
Maternal BMI	0.025	0.000	0.56	0.72	0.129	0.000	0.03	0.17	0.120	0.000	0.02	0.17	0.033	0.000	0.43	0.70
Anterior limb of internal capsule																
IL-10	−0.148	0.001	<0.01	**0.01**	0.066	0.004	0.26	0.42	−0.070	0.003	0.19	0.38	0.054	0.004	0.23	0.39
Sex	−0.033	0.002	0.44	0.83	−0.023	0.007	0.69	0.96	−0.080	0.005	0.13	0.34	−0.033	0.006	0.46	0.83
NICU stay	−0.073	0.005	0.18	0.59	−0.070	0.016	0.33	0.65	0.075	0.011	0.27	0.65	0.092	0.014	0.11	0.49
GA at delivery	0.121	0.001	0.04	0.45	−0.036	0.003	0.64	0.98	0.129	0.002	0.07	0.52	−0.013	0.002	0.83	0.98
PMA at scan	0.590	0.001	<0.001	**<0.001**	−0.096	0.003	0.12	0.13	−0.453	0.002	<0.001	**<0.001**	−0.616	0.002	<0.001	**<0.001**
Maternal BMI	0.024	0.000	0.58	0.72	0.142	0.000	0.02	0.17	0.025	0.000	0.64	0.77	0.014	0.000	0.75	0.82
Inferior cingulum bundle																
IL-10	−0.059	0.001	0.27	0.42	0.062	0.002	0.28	0.42	0.031	0.002	0.58	0.67	0.079	0.002	0.09	0.23
Sex	0.008	0.002	0.88	0.96	0.022	0.003	0.70	0.96	−0.101	0.003	0.07	0.27	−0.104	0.003	0.03	0.18
NICU stay	−0.102	0.005	0.14	0.49	0.009	0.007	0.90	0.99	−0.001	0.008	0.99	0.99	0.101	0.007	0.09	0.45
GA at delivery	−0.112	0.001	0.12	0.52	0.111	0.001	0.14	0.52	0.052	0.001	0.48	0.92	0.169	0.001	0.01	0.27
PMA at scan	0.420	0.001	<0.001	**<0.001**	−0.225	0.001	<0.001	**<0.001**	−0.310	0.001	<0.001	**<0.001**	−0.636	0.001	<0.001	**<0.001**
Maternal BMI	0.062	0.000	0.25	0.53	0.117	0.000	0.04	0.22	0.127	0.000	0.02	0.17	0.058	0.000	0.22	0.50
Fornix																
IL-10	−0.041	0.001	0.45	0.61	0.036	0.002	0.54	0.67	−0.004	0.002	0.93	0.96	0.028	0.002	0.54	0.67
Sex	−0.094	0.002	0.09	0.28	0.030	0.004	0.60	0.94	−0.096	0.004	0.06	0.27	−0.007	0.003	0.88	0.96
NICU stay	−0.003	0.004	0.97	0.99	−0.072	0.008	0.32	0.65	0.003	0.009	0.97	0.99	0.017	0.007	0.77	0.96
GA at delivery	0.040	0.001	0.59	0.98	−0.015	0.001	0.85	0.98	0.103	0.001	0.14	0.52	0.047	0.001	0.44	0.89
PMA at scan	0.341	0.001	<0.001	**<0.001**	−0.187	0.001	<0.001	**<0.001**	−0.506	0.001	<0.001	**<0.001**	−0.641	0.001	<0.001	**<0.001**
Maternal BMI	−0.033	0.000	0.55	0.72	0.022	0.000	0.70	0.82	0.079	0.000	0.13	0.40	0.079	0.000	0.09	0.35

TNF-ɑ																
Corpus callosum																
TNF-ɑ	−0.002	0.002	0.97	0.97	0.020	0.002	0.73	0.97	−0.062	0.002	0.27	0.97	−0.022	0.003	0.64	0.97
Sex	−0.088	0.003	0.08	0.25	−0.058	0.004	0.32	0.67	−0.091	0.003	0.11	0.26	0.032	0.004	0.50	0.88
NICU stay	−0.100	0.007	0.12	0.67	−0.070	0.008	0.34	0.67	0.019	0.008	0.79	0.97	0.030	0.010	0.63	0.91
GA at delivery	0.022	0.001	0.75	0.93	−0.017	0.001	0.82	0.93	0.135	0.001	0.09	0.52	−0.030	0.002	0.66	0.93
PMA at scan	0.453	0.001	<0.001	**<0.001**	−0.157	0.001	0.01	**0.01**	−0.223	0.001	<0.001	**<0.001**	−0.554	0.002	<0.001	**<0.001**
Maternal BMI	0.054	0.000	0.29	0.56	0.088	0.000	0.14	0.41	0.076	0.000	0.18	0.47	−0.030	0.000	0.54	0.78
Superior cingulum bundle																
TNF-ɑ	0.003	0.002	0.95	0.97	0.036	0.003	0.54	0.97	0.029	0.003	0.59	0.97	0.011	0.003	0.82	0.97
Sex	−0.100	0.003	0.06	0.22	0.013	0.005	0.82	0.93	−0.120	0.005	0.02	0.21	0.012	0.005	0.80	0.93
NICU stay	−0.057	0.006	0.41	0.68	−0.158	0.011	0.03	0.36	0.067	0.011	0.33	0.67	0.102	0.011	0.12	0.67
GA at delivery	0.019	0.001	0.80	0.93	−0.034	0.002	0.65	0.93	0.102	0.002	0.17	0.67	0.063	0.002	0.37	0.93
PMA at scan	0.341	0.001	<0.001	**<0.001**	−0.127	0.002	0.04	**0.04**	−0.413	0.002	<0.001	**<0.001**	−0.506	0.002	<0.001	**<0.001**
Maternal BMI	−0.047	0.000	0.38	0.63	0.046	0.000	0.44	0.66	−0.018	0.000	0.74	0.83	0.022	0.000	0.66	0.80
Corticospinal tract																
TNF-ɑ	−0.038	0.002	0.44	0.97	0.019	0.003	0.75	0.97	−0.020	0.002	0.67	0.97	0.022	0.003	0.61	0.97
Sex	−0.007	0.003	0.88	0.93	0.006	0.005	0.92	0.93	−0.086	0.003	0.06	0.22	−0.029	0.005	0.50	0.88
NICU stay	0.017	0.007	0.79	0.97	−0.076	0.012	0.29	0.67	0.058	0.008	0.32	0.67	−0.005	0.011	0.93	0.97
GA at delivery	0.046	0.001	0.49	0.93	0.009	0.002	0.90	0.93	0.071	0.001	0.26	0.85	0.009	0.002	0.88	0.93
PMA at scan	0.528	0.001	<0.001	**<0.001**	−0.212	0.002	<0.001	**<0.001**	−0.636	0.001	<0.001	**<0.001**	−0.670	0.002	<0.001	**<0.001**
Maternal BMI	0.026	0.000	0.59	0.80	0.068	0.000	0.24	0.52	0.040	0.000	0.38	0.63	0.000	0.000	0.99	0.99
Optic radiation																
TNF-ɑ	−0.031	0.002	0.51	0.97	−0.016	0.004	0.78	0.97	−0.002	0.003	0.97	0.97	0.020	0.003	0.64	0.97
Sex	−0.024	0.003	0.62	0.91	−0.007	0.006	0.90	0.93	−0.202	0.005	<0.01	**0.01**	−0.082	0.005	0.06	0.22
NICU stay	−0.153	0.007	0.01	0.36	−0.062	0.014	0.39	0.67	0.014	0.011	0.84	0.97	0.123	0.012	0.03	0.36
GA at delivery	−0.022	0.001	0.74	0.93	0.026	0.002	0.74	0.93	−0.012	0.002	0.87	0.93	−0.007	0.002	0.91	0.93
PMA at scan	0.545	0.001	<0.001	**<0.001**	−0.169	0.002	0.01	**0.01**	−0.323	0.002	<0.001	**<0.001**	−0.621	0.002	<0.001	**<0.001**
Maternal BMI	0.009	0.000	0.84	0.87	0.139	0.000	0.02	0.15	0.173	0.000	<0.01	0.05	0.091	0.000	0.04	0.20
Uncinate fasciculus																
TNF-ɑ	−0.127	0.001	0.01	0.27	−0.016	0.003	0.78	0.97	−0.125	0.003	0.02	0.27	0.016	0.003	0.72	0.97
Sex	−0.078	0.002	0.10	0.26	0.008	0.005	0.89	0.93	−0.131	0.005	0.02	0.19	−0.028	0.004	0.54	0.88
NICU stay	−0.057	0.005	0.36	0.67	0.003	0.011	0.97	0.97	−0.075	0.011	0.29	0.67	0.002	0.010	0.97	0.97
GA at delivery	0.066	0.001	0.32	0.88	0.064	0.002	0.41	0.93	0.140	0.002	0.06	0.52	0.043	0.002	0.50	0.93
PMA at scan	0.501	0.001	<0.001	**<0.001**	−0.093	0.002	0.14	0.14	−0.293	0.002	<0.001	**<0.001**	−0.621	0.002	<0.001	**<0.001**
Maternal BMI	0.040	0.000	0.41	0.64	0.076	0.000	0.20	0.48	0.093	0.000	0.09	0.38	0.048	0.000	0.31	0.56
Inferior fronto-occipital fasciculus																
TNF-ɑ	0.006	0.002	0.88	0.97	−0.021	0.004	0.72	0.97	0.011	0.002	0.83	0.97	−0.012	0.003	0.77	0.97
Sex	−0.004	0.003	0.93	0.93	−0.012	0.006	0.84	0.93	−0.176	0.004	<0.01	**0.01**	−0.072	0.005	0.08	0.25
NICU stay	−0.078	0.006	0.16	0.67	−0.063	0.014	0.39	0.67	0.042	0.008	0.53	0.80	0.071	0.012	0.19	0.67
GA at delivery	0.069	0.001	0.25	0.85	−0.023	0.002	0.76	0.93	0.121	0.001	0.09	0.52	−0.022	0.002	0.71	0.93
PMA at scan	0.619	0.001	<0.001	**<0.001**	−0.127	0.002	0.04	**0.04**	−0.457	0.001	<0.001	**<0.001**	−0.669	0.002	<0.001	**<0.001**
Maternal BMI	0.013	0.000	0.77	0.84	0.123	0.000	0.04	0.20	0.126	0.000	0.02	0.15	0.043	0.000	0.31	0.56
Anterior limb of internal capsule																
TNF-ɑ	0.019	0.001	0.66	0.97	−0.055	0.004	0.35	0.97	−0.058	0.003	0.27	0.97	−0.050	0.004	0.26	0.97
Sex	−0.022	0.002	0.61	0.91	−0.026	0.007	0.65	0.91	−0.060	0.005	0.25	0.57	−0.029	0.006	0.51	0.88
NICU stay	−0.044	0.005	0.44	0.68	−0.078	0.016	0.28	0.67	0.079	0.011	0.25	0.67	0.075	0.014	0.19	0.67
GA at delivery	0.129	0.001	0.03	0.52	−0.040	0.003	0.60	0.93	0.118	0.002	0.11	0.52	−0.025	0.002	0.68	0.93
PMA at scan	0.594	0.001	<0.001	**<0.001**	−0.098	0.003	0.11	0.12	−0.450	0.002	<0.001	**<0.001**	−0.617	0.002	<0.001	**<0.001**
Maternal BMI	0.018	0.000	0.69	0.80	0.136	0.000	0.02	0.15	0.026	0.000	0.63	0.80	0.019	0.000	0.68	0.80
Inferior cingulum bundle																
TNF-ɑ	−0.126	0.001	0.02	0.27	0.032	0.002	0.57	0.97	−0.073	0.002	0.18	0.97	0.056	0.002	0.22	0.97
Sex	−0.009	0.002	0.85	0.93	0.026	0.003	0.66	0.91	−0.114	0.003	0.04	0.22	−0.094	0.003	0.04	0.22
NICU stay	−0.100	0.005	0.14	0.67	0.006	0.007	0.94	0.97	−0.014	0.008	0.84	0.97	0.087	0.006	0.14	0.67
GA at delivery	−0.124	0.001	0.09	0.52	0.119	0.001	0.12	0.52	0.033	0.001	0.66	0.93	0.158	0.001	0.01	0.48
PMA at scan	0.433	0.001	<0.001	**<0.001**	−0.227	0.001	<0.001	**<0.001**	−0.319	0.001	<0.001	**<0.001**	−0.645	0.001	<0.001	**<0.001**
Maternal BMI	0.074	0.000	0.16	0.44	0.110	0.000	0.06	0.26	0.139	0.000	0.01	0.15	0.054	0.000	0.24	0.52
Fornix																
TNF-ɑ	0.003	0.001	0.96	0.97	0.023	0.002	0.69	0.97	−0.071	0.002	0.16	0.97	−0.069	0.002	0.12	0.97
Sex	−0.104	0.002	0.05	0.22	0.039	0.003	0.50	0.88	−0.082	0.004	0.11	0.26	0.010	0.003	0.83	0.93
NICU stay	−0.005	0.004	0.94	0.97	−0.066	0.008	0.36	0.67	0.006	0.008	0.93	0.97	0.020	0.006	0.72	0.97
GA at delivery	0.034	0.001	0.64	0.93	0.001	0.001	0.99	0.99	0.075	0.001	0.29	0.86	0.029	0.001	0.63	0.93
PMA at scan	0.345	0.001	<0.001	**<0.001**	−0.183	0.001	<0.001	**<0.001**	−0.509	0.001	<0.001	**<0.001**	−0.647	0.001	<0.001	**<0.001**
Maternal BMI	−0.026	0.000	0.63	0.80	0.013	0.000	0.82	0.87	0.078	0.000	0.13	0.41	0.072	0.000	0.11	0.39

Multiple linear regression results of the association between maternal cytokine concentrations and neonatal dMRI parameters. Covariates in models are child sex, NICU stay > 7 days, gestational age at delivery, infant postmenstrual age at scan, and maternal BMI pre-pregnancy. Bolded values indicate significant association after FDR correction for multiple comparisons.

*β* standardized beta coefficient, *SE* standard error, *q* FDR-corrected p-value; *IL* interleukin, *TNF-α* tumor necrosis factor alpha, *FA* fractional anisotropy, *MD* mean diffusivity; *AD* axial diffusivity, *RD* radial diffusivity, *Sex* child sex, *GA,* gestational age, *PMA* infant postmenstrual age.

### Moderating effect of socioeconomic status in the relationship between maternal inflammation and neonatal white matter microstructure

**Fig. 3:**
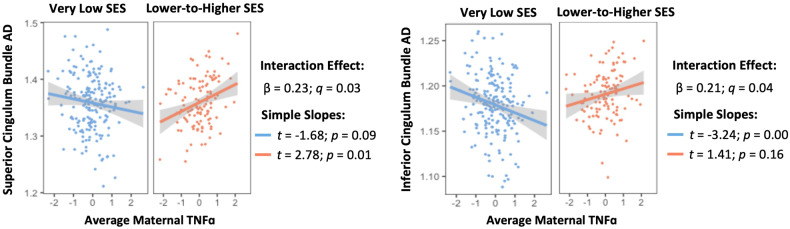
Moderating effect of family socioeconomic status group in the relationship between neonatal white matter dMRI parameters and average maternal cytokine concentration. Covariates in models are child sex, NICU stay > 7 days, gestational age at delivery, infant postmenstrual age at scan, and maternal BMI pre-pregnancy. MD mean diffusivity, AD axial diffusivity, TNF-α tumor necrosis factor alpha, SES socioeconomic status, *β* standardized beta, *q* FDR-corrected *p*-value.

There were significant interactions between family SES group and average maternal TNF-α concentration during pregnancy on neonatal CB AD (*β* = 0.23; *q* = 0.03) and CBIF AD (*β* = 0.21; *q* = 0.04). As Fig. 3 shows, simple slopes analyses indicated that TNF-α and CB AD values were positively and significantly associated among the lower-to-higher SES group neonates (*t* = 2.78; *p* = 0.01; SE = 0.00). However, these associations were not significant among the very low SES group neonates. By contrast, simple slopes analyses of CBIF AD indicated that its association with TNF-α was significant and negative in the very low SES group (*t* = −3.24; *p* < 0.01; SE = 0.00) but positive and not significance in the lower-to-higher SES group (*t* = 1.41; *p* = 0.16; SE = 0.00). Family SES group did not interact with average maternal IL-6, IL-8, or IL-10 concentration for any neonatal white matter tract measures (see Supplementary Table 3).

## Discussion

In this study, we capitalized on a cohort of mother-infant dyads oversampled for poverty to investigate early white matter development as a function of disadvantage-related prenatal inflammation. Consistent with our hypotheses, we found that family SES was associated with maternal cytokine concentrations during pregnancy and neonatal white matter microstructure. Very low SES was associated with higher average maternal IL-6 and lower diffusivity across multiple tracts in neonates. Additionally, higher maternal cytokine concentrations were associated with alterations in neonatal white matter microstructure. Furthermore, these associations varied across cytokines. Higher average maternal IL-6 was associated with lower tract FA and AD, whereas higher average maternal IL-10 was associated with lower tract FA and AD but higher RD. An intriguing finding was that family SES moderated the relationship between average maternal TNF-α levels during gestation and neonatal white matter diffusivity, such that the association was significant and positive in the lower-to-higher SES neonates for superior cingulum AD, but significant and negative in the very low SES neonates for inferior cingulum AD. This suggests differential relationships of the effects of cytokines on white matter development depending upon SES context and raises questions about the divergence of biological mechanisms depending upon foundational resources/SES in utero.

The developing fetus’s expected extrauterine environment is communicated via maternal biological mediators that cross the placental barrier. Less favorable socioeconomic conditions impart unique mental, physical, and social challenges that, once born, a neonate must successfully interact with to facilitate survival. Fetal developmental trajectories are, therefore, malleable to environmental conditions. In the current study, we found that family SES was inversely related to maternal IL-6 levels during pregnancy. Chronic stress associated with economic disadvantage is hypothesized to result in a low-grade inflammatory phenotype, which is ultimately resistant to the dampening effects of glucocorticoids [22, 23]. Regarding the specificity of IL-6 as the only cytokine associated with SES, similar patterns have been reported in other pregnancy cohorts. For example, Miller et al. [74] found that maternal childhood disadvantage was associated solely with higher circulating levels of IL-6, but not other inflammatory cytokines. Moreover, in that study, IL-6 also mediated the relationship between disadvantage and adverse pregnancy outcomes (i.e., preterm birth, shorter gestation length). They suggested that these results could reflect IL-6’s role in the progression from acute to chronic inflammation, ultimately explaining many long-term health consequences in individuals from lower SES environments.

Socioeconomic disadvantage has also been linked with aberrant neonatal white matter microstructure, whereby exposure to greater familial disadvantage early in life is associated with lower MD and RD in many major tracts [49]. Similarly, we found a positive relationship between family SES and neonatal white matter diffusivity in all tracts except the corpus callosum. The differential timing of white matter tract development in the human brain is characterized by two categories: “early developing” and “late developing” [75]. Early developing tracts include projections connecting sensorimotor regions, whereas later developing tracts project to and from association systems. Myelination starts in utero and, for later developing tracts, is not complete until adolescence. Yet, exposure to stressors in utero has the potential to disrupt synaptic plasticity and myelination, ultimately affecting the course of neurodevelopment in profound ways, such as promoting accelerated brain maturation and reduced plasticity [2, 76]. Within the range of PMA studied herein, some tracts are myelinated while others are not. Our results suggest that economic disadvantage may modify white matter microstructure independent of tract myelination timing. Consistent with findings from Lean et al. [49] in this sample, this indicates that chronic exposure to maternally mediated stressors in utero may alter white matter maturation, particularly in the context of low SES (see Supplementary Information for a more in-depth discussion).

Maternal inflammatory biomarkers during pregnancy were also associated with variations in white matter microstructure. Higher maternal average IL-6 levels during pregnancy were related to lower uncinate AD, as well as lower corticospinal tract FA. Chronically elevated IL-6 may have deleterious effects on cell survival, synaptogenesis, and axonal growth [16]. Our results support the notion that heightened inflammatory cytokine exposure may alter the course of axonal maturation, as indicated by AD. In terms of tract FA, a study by Rasmussen et al. [69] similarly found that elevated maternal IL-6 was associated with lower uncinate FA in newborns. The uncinate plays a key role in communication between regions involved in emotion regulation and higher-order cognition, and its integrity is associated with socioemotional development. Our results in conjunction with those of Rasmussen et al. suggest that the uncinate in a developing fetus may be uniquely sensitive to maternal inflammation during pregnancy. In addition, we found that higher average maternal IL-10 concentration was associated higher corpus callosum, corticospinal, and optic radiation RD, lower uncinate AD, and lower corpus callosum, corticospinal, inferior fronto-occipital fasciculus, and anterior limb of internal capsule FA. IL-10, a key anti-inflammatory cytokine, can suppress inflammatory cytokine production. However, chronically elevated IL-10 concentrations have been implicated in behavioral abnormalities [77] and demyelination of white matter [78] in animal models. As discussed in the previous paragraph, neonatal white matter tract development is spatiotemporally distinct. Processes such as myelination and axonal growth and packing occur at varying maturational periods that also differ by tract [79–81]. Our results suggest that maternal levels of both IL-6 and IL-10 were associated with neonatal white matter tract differences regardless of myelination timing. Yet, maternal IL-10 was associated with a wider range of tracts than was maternal IL-6. This raises the question of why neonatal white matter tracts seem to be differentially sensitive. Depending on exactly *how* these inflammatory signals reach the fetal interface, exposure to elevated pro-inflammatory cytokines like IL-6 in utero may impede the expression of proteins responsible for axonal and oligodendrocyte differentiation, while heightened exposure to anti-inflammatory cytokines like IL-10 may stimulate the activity of macrophage phagocytosis and microglia [82, 83]. Whether the mechanism by which these signals reach the fetus, or if the timing of specific cytokine exposure plays a role in differential white matter sensitivity remains a topic of ongoing investigation. Nonetheless, our results suggest that elevated maternal cytokine concentrations during gestation may have implications for the maturational timing of major projection, limbic, and association fibers.

Notably, the relationship between maternal TNF-α during pregnancy and neonatal white matter diffusivity was moderated by the SES context of the dyad. Specifically, higher concentrations of maternal TNF-α interacted with family SES to predict superior and inferior cingulum diffusivity. This association suggested that higher prenatal TNF-α exposure related to reduced axonal integrity in the inferior cingulum tract specifically in neonates from very low SES families. Interestingly, the opposite pattern was observed in lower-to-higher SES neonates, with neonates born to mothers experiencing less disadvantage showing higher superior cingulum AD in relation to elevated TNF-α exposure in utero. These findings are consistent with a critical and central facet of the neuroimmune network hypothesis: the early environmental context differentially impacts crosstalk between neural circuitry and the immune system [54]. The question of why elevated TNF-α affects the developing fetal brain differentially in low vs. high SES conditions is unclear. However, we offer two speculative scenarios by which this may occur. First, low SES could involve inflammation relative to environmental conditions. Low SES conditions may start a positive feedback circuit, involving peripheral cytokines and threat circuitry [17]. The latter develops early enough that it is plausible here and can, via the sympathetic nervous system, affect peripheral immune activity. Second, the stressors that low SES mothers face may disrupt regulatory pathways that usually counter inflammation (e.g., induces resistance to glucocorticoid inhibition of cytokine release) [54, 84–86]. Low SES environments may amplify brain-immune bidirectional communication whereby the chronicity of stress-induced inflammation promotes a positive feedback circuit between peripheral cytokines and brain systems [21]. While we cannot make specific inferences about the neurobiological source of alterations to diffusion parameters, this pattern suggests that sustained exposure to elevated TNF-α during gestation may alter fronto-limbic axonal development via aberrant pruning or reduced fiber branching among neonates from very low SES environments. Enhanced brain-immune crosstalk characterized by stress-induced inflammatory phenotypes may also function to prematurely prompt fetal brain development in preparation for a harsh extrauterine environment. While potentially advantageous for immediate survival, atypical maturation could hinder experience-dependent synaptic plasticity that prolonged, more typical maturation affords. Given that the white matter projections implicated here are critical for social and emotional development and typically continue to myelinate into the postnatal period, their aberrant maturation may have detrimental consequences for healthy socioemotional development. These findings raise important questions regarding how prenatal disadvantage may alter the course of brain development.

This study had several limitations. First, the current analyses are cross-sectional in design, precluding from the ability to make specific mechanistic inferences regarding how maternal inflammatory biomarkers during pregnancy and their association with economic disadvantage might impact brain maturation. However, the eLABE study is longitudinal in nature, and we will be able to investigate how these factors relate to trajectories of brain development into the second year of life and beyond. Second, our measure of brain-immune interaction is indirect, as methodology to better assess this is still being explored [17, 87]. Lastly, there was some variability in blood collection time by trimester as well as unequal sample sizes by trimester. Therefore, we averaged maternal cytokine concentrations over the course of pregnancy for the main analyses but include results by trimester in the Supplemental Information. It should be noted that there remains no clear explanation as to why cytokines were differentially associated with varying white matter tracts. While we outlined the hypothesized role of each cytokine in neuronal development, further studies are necessary to uncover the exact mechanisms by which maternal cytokines influence fetal development and, more specifically, white matter microstructural changes.

In summary, our results provide unique information about the relationships between SES and maternal inflammatory biomarkers, and in turn, how these factors interact in unique ways that are context dependent, to impact the developing fetus. We found that while average pregnancy levels of maternal IL-6 and IL-10 were associated with variations in neonatal white matter microstructure, average pregnancy levels of maternal TNF-α uniquely interacted with family SES to predict fronto-limbic white matter tract diffusivity. These findings suggest that neonates from very low SES families experience altered white matter tract maturation under high inflammatory conditions. Future studies that further interrogate these unique context-dependent trajectories in utero are warranted.

### Supplementary information

Supplemental Material

## Data Availability

Data is currently not publicly available; however, these data are intended to be published in a public repository at a future date.

## Code availability

Code for all statistical analyses is available upon request by emailing AFPS.
