# Supplementary material for: Prenatal exposure to maternal disadvantage-related inflammatory biomarkers: associations with neonatal white matter microstructure
Source: Transl Psychiatry. 2024 Feb 2;14:72. doi: 10.1038/s41398-024-02782-6 (PMC10837200; doi:10.1038/s41398-024-02782-6)
Supplement: Supplementary file 1 — Supplemental Material [file 41398_2024_2782_MOESM1_ESM.pdf]

Supplementary Information

Table S1. Within SES group means and standard deviations of maternal cytokine levels and neonatal white matter dMRI parameters.

		Very Low SES		Lower-to-Higher SES	
		M	*SD*	*M*	*SD*
**Maternal Cytokine Levels**	***IL-6***	0.13	0.86	-0.19	0.83
	IL-8	-0.06	0.80	-0.01	0.86
	IL-10	0.01	0.79	-0.05	0.86
	TNF-α	-0.06	0.78	0.01	0.83
**Corpus Callosum**	***FA***	0.34	0.03	0.35	0.03
	MD	1.01	0.03	1.00	0.03
	AD	1.40	0.03	1.40	0.03
	RD	0.81	0.04	0.80	0.04
**Superior Cingulum Bundle**	***FA***	0.23	0.03	0.22	0.02
	MD	1.10	0.04	1.11	0.04
	AD	1.36	0.05	1.36	0.04
	RD	0.97	0.05	0.98	0.05
**Corticospinal Tract**	***FA***	0.27	0.03	0.27	0.03
	MD	0.98	0.05	0.98	0.04
	AD	1.26	0.04	1.27	0.03
	RD	0.84	0.06	0.83	0.05
**Optic Radiation**	***FA***	0.21	0.03	0.22	0.03
	MD	1.07	0.05	1.08	0.05
	AD	1.31	0.05	1.33	0.04
	RD	0.95	0.06	0.95	0.06
**Uncinate Fasciculus**	***FA***	0.23	0.03	0.23	0.03
	MD	1.05	0.04	1.05	0.04
	AD	1.31	0.05	1.32	0.04
	RD	0.91	0.05	0.92	0.04
**Inferior Fronto-Occipital Fasciculus**	***FA***	0.23	0.03	0.24	0.03
	MD	1.09	0.06	1.09	0.05
	AD	1.35	0.04	1.37	0.03
	RD	0.96	0.07	0.96	0.06
**Anterior Limb of Internal Capsule**	***FA***	0.17	0.03	0.18	0.02
	MD	1.12	0.06	1.13	0.06
	AD	1.32	0.05	1.33	0.04
	RD	1.02	0.07	1.02	0.07
**Inferior Cingulum Bundle**	***FA***	0.21	0.02	0.21	0.02
	MD	0.96	0.03	0.97	0.02
	AD	1.18	0.03	1.19	0.03
	RD	0.86	0.03	0.86	0.03
**Fornix**	***FA***	0.23	0.02	0.23	0.01
	MD	0.95	0.03	0.95	0.03
	AD	1.19	0.04	1.19	0.04
	RD	0.83	0.03	0.83	0.03

Cytokine levels ae log_{10} transformed values; *M*, mean; *SD*, standard deviation; FA, fractional anisotropy; MD, mean diffusivity; AD, axial diffusivity; RD, radial diffusivity; SES, socioeconomic status.

Table S2. Pearson correlation matrix of average maternal cytokine levels during pregnancy.

	Maternal Avg. IL-6	Maternal Avg. IL-8	Maternal Avg. IL-10	Maternal Avg. TNF-α
**Maternal Avg. IL-6**	1.00			
**Maternal Avg. IL-8**	0.18	1.00		
**Maternal Avg. IL-10**	0.37	0.25	1.00	
**Maternal Avg. TNF-α**	0.39	0.33	0.36	1.00

Values represent Pearson correlation coefficients. All p values < 0.001. IL, interleukin; TNF-α, tumor necrosis factor alpha; Avg., average.

Table S3. Multiple linear regression results of the moderating role of family socioeconomic status group in the relationship between average maternal cytokine concentration and neonatal dMRI parameters.

	FA				MD				AD				RD			
	β	SE	*p*	*q*	β	SE	*p*	*q*	β	SE	*p*	*q*	β	SE	*p*	*q*
**IL-6**																
**Corpus Callosum**																
IL-6	-0.008	0.002	0.89	0.92	-0.016	0.003	0.83	0.92	-0.097	0.002	0.19	0.39	-0.032	0.003	0.59	0.73
SES group	0.073	0.003	0.16	0.33	-0.027	0.004	0.65	0.79	0.116	0.004	0.05	0.18	-0.042	0.005	0.39	0.59
Sex	-0.092	0.003	0.07	0.27	-0.072	0.004	0.21	0.48	-0.078	0.003	0.17	0.41	0.041	0.004	0.39	0.77
NICU stay	-0.107	0.007	0.09	0.73	-0.054	0.009	0.46	0.47	0.009	0.008	0.90	0.73	0.030	0.010	0.62	0.73
GA at delivery	0.006	0.001	0.93	0.95	0.023	0.001	0.77	0.92	0.115	0.001	0.15	0.82	-0.022	0.002	0.74	0.92
PMA at scan	0.451	0.001	<0.001	**<0.001**	-0.153	0.001	0.02	**0.02**	-0.244	0.001	<0.001	**<0.001**	-0.559	0.002	<0.001	**<0.001**
Maternal BMI	0.053	0.000	0.30	0.64	0.080	0.000	0.18	0.63	0.034	0.000	0.56	0.83	-0.044	0.000	0.37	0.64
IL-6:SES group	-0.091	0.004	0.15	0.46	0.182	0.005	0.01	0.35	0.014	0.004	0.85	0.88	0.099	0.005	0.10	0.35
**Superior Cingulum Bundle**																
IL-6	0.004	0.002	0.96	0.96	-0.133	0.004	0.07	0.26	-0.099	0.004	0.14	0.36	-0.077	0.004	0.22	0.41
SES group	-0.122	0.003	0.03	0.14	-0.003	0.006	0.96	0.96	0.054	0.005	0.33	0.55	0.153	0.005	<0.001	**0.03**
Sex	-0.106	0.003	0.05	0.27	0.008	0.005	0.89	0.98	-0.119	0.005	0.03	0.27	0.017	0.005	0.73	0.98
NICU stay	-0.051	0.006	0.47	0.73	-0.150	0.012	0.04	0.73	0.052	0.011	0.45	0.73	0.086	0.011	0.18	0.73
GA at delivery	0.039	0.001	0.61	0.92	-0.031	0.002	0.69	0.92	0.072	0.002	0.33	0.92	0.025	0.002	0.72	0.92
PMA at scan	0.356	0.001	<0.001	**<0.001**	-0.131	0.002	0.04	**0.04**	-0.418	0.002	<0.001	**<0.001**	-0.523	0.002	<0.001	**<0.001**
Maternal BMI	-0.037	0.000	0.51	0.80	0.052	0.000	0.39	0.64	-0.048	0.000	0.38	0.64	-0.006	0.000	0.90	0.92
IL-6:SES group	0.060	0.003	0.38	0.73	0.150	0.006	0.04	0.35	0.129	0.006	0.05	0.35	0.042	0.006	0.50	0.73
**Corticospinal Tract**																
IL-6	-0.186	0.002	<0.01	0.07	0.033	0.004	0.64	0.77	-0.094	0.002	0.09	0.27	0.082	0.003	0.13	0.36
SES group	0.027	0.003	0.59	0.75	-0.033	0.006	0.58	0.75	0.202	0.004	<0.001	**<0.001**	0.066	0.005	0.14	0.31
Sex	-0.002	0.003	0.96	0.98	0.007	0.005	0.90	0.98	-0.071	0.003	0.10	0.29	-0.028	0.005	0.51	0.93
NICU stay	0.005	0.006	0.93	0.96	-0.061	0.012	0.40	0.73	0.043	0.007	0.45	0.96	-0.003	0.011	0.96	0.96
GA at delivery	0.049	0.001	0.46	0.92	0.041	0.002	0.60	0.92	0.018	0.001	0.76	0.92	-0.019	0.002	0.74	0.92
PMA at scan	0.515	0.001	<0.001	**<0.001**	-0.210	0.002	<0.001	**<0.001**	-0.662	0.001	<0.001	**<0.001**	-0.671	0.002	<0.001	**<0.001**
Maternal BMI	0.053	0.000	0.28	0.64	0.053	0.000	0.38	0.64	-0.015	0.000	0.73	0.85	-0.044	0.000	0.32	0.64
IL-6:SES group	-0.033	0.003	0.57	0.76	0.099	0.007	0.16	0.46	0.014	0.004	0.80	0.88	0.045	0.006	0.40	0.73
**Optic Radiation**																
IL-6	-0.127	0.002	0.03	0.21	-0.047	0.004	0.52	0.72	-0.111	0.003	0.09	0.27	0.031	0.004	0.58	0.73
SES group	0.074	0.003	0.14	0.31	0.014	0.007	0.82	0.90	0.237	0.005	<0.001	**<0.001**	0.075	0.006	0.10	0.28
Sex	-0.023	0.003	0.63	0.94	-0.024	0.006	0.69	0.98	-0.200	0.005	<0.001	**<0.001**	-0.078	0.005	0.08	0.27
NICU stay	-0.160	0.007	0.01	0.32	-0.072	0.014	0.32	0.96	-0.007	0.010	0.91	0.73	0.119	0.012	0.04	0.47
GA at delivery	-0.034	0.001	0.61	0.92	0.026	0.002	0.74	0.92	-0.074	0.002	0.30	0.90	-0.031	0.002	0.61	0.92
PMA at scan	0.532	0.001	<0.001	**<0.001**	-0.176	0.002	0.01	**0.01**	-0.349	0.002	<0.001	**<0.001**	-0.624	0.002	<0.001	**<0.001**
Maternal BMI	0.012	0.000	0.80	0.85	0.132	0.000	0.03	0.40	0.138	0.000	0.01	0.34	0.068	0.000	0.13	0.53
IL-6:SES group	0.044	0.004	0.45	0.73	0.078	0.008	0.28	0.71	0.000	0.006	1.00	1.00	-0.034	0.007	0.54	0.74
**Uncinate Fasciculus**																
IL-6	-0.123	0.002	0.04	0.21	-0.104	0.003	0.15	0.37	-0.160	0.003	0.02	0.18	-0.023	0.003	0.69	0.80

SES group	-0.041	0.003	0.41	0.59	0.019	0.005	0.76	0.86	0.088	0.005	0.12	0.30	0.093	0.005	0.05	0.18
Sex	-0.086	0.002	0.07	0.27	0.003	0.005	0.96	0.98	-0.116	0.005	0.03	0.27	-0.009	0.004	0.85	0.98
NICU stay	-0.059	0.006	0.34	0.73	-0.004	0.011	0.96	0.73	-0.072	0.011	0.30	0.87	0.007	0.010	0.91	0.96
GA at delivery	0.080	0.001	0.23	0.82	0.084	0.002	0.29	0.90	0.127	0.002	0.09	0.82	0.020	0.002	0.75	0.92
PMA at scan	0.504	0.001	<0.001	**<0.001**	-0.102	0.002	0.11	0.11	-0.318	0.002	<0.001	**<0.001**	-0.641	0.002	<0.001	**<0.001**
Maternal BMI	0.047	0.000	0.34	0.64	0.093	0.000	0.13	0.53	0.059	0.000	0.29	0.64	0.019	0.000	0.68	0.85
IL-6:SES group	-0.015	0.003	0.80	0.88	0.075	0.006	0.30	0.72	-0.093	0.006	0.17	0.46	-0.051	0.005	0.37	0.73
**Inferior Fronto-Occipital Fasciculus**																
IL-6	-0.038	0.002	0.48	0.70	-0.078	0.004	0.28	0.48	-0.148	0.002	0.02	0.18	-0.032	0.004	0.55	0.73
SES group	0.014	0.003	0.76	0.86	-0.003	0.007	0.96	0.96	0.194	0.004	<0.001	**<0.001**	0.064	0.006	0.15	0.31
Sex	0.001	0.003	0.98	0.98	-0.020	0.006	0.74	0.98	-0.165	0.003	<0.001	**0.02**	-0.070	0.006	0.10	0.29
NICU stay	-0.078	0.007	0.16	0.73	-0.056	0.014	0.44	0.73	0.036	0.008	0.58	0.73	0.069	0.013	0.21	0.73
GA at delivery	0.077	0.001	0.20	0.82	-0.011	0.002	0.89	0.95	0.090	0.001	0.20	0.82	-0.040	0.002	0.49	0.92
PMA at scan	0.614	0.001	<0.001	**<0.001**	-0.125	0.002	0.05	0.05	-0.485	0.001	<0.001	**<0.001**	-0.675	0.002	<0.001	**<0.001**
Maternal BMI	0.022	0.000	0.62	0.83	0.119	0.000	0.05	0.45	0.078	0.000	0.13	0.53	0.016	0.000	0.71	0.85
IL-6:SES group	-0.026	0.004	0.64	0.82	0.119	0.008	0.10	0.35	0.051	0.004	0.42	0.73	0.036	0.007	0.49	0.73
**Anterior Limb of Internal Capsule**																
IL-6	-0.008	0.002	0.88	0.92	-0.070	0.005	0.33	0.52	-0.124	0.003	0.06	0.26	-0.062	0.004	0.28	0.48
SES group	0.030	0.003	0.51	0.70	0.029	0.008	0.64	0.79	0.139	0.005	0.01	0.06	0.059	0.007	0.21	0.39
Sex	-0.022	0.002	0.61	0.94	-0.038	0.007	0.52	0.93	-0.054	0.005	0.31	0.65	-0.025	0.006	0.58	0.94
NICU stay	-0.043	0.006	0.44	0.73	-0.073	0.016	0.32	0.96	0.061	0.011	0.37	0.88	0.064	0.014	0.27	0.73
GA at delivery	0.130	0.001	0.03	0.82	-0.020	0.003	0.79	0.92	0.098	0.002	0.18	0.82	-0.038	0.002	0.54	0.92
PMA at scan	0.593	0.001	<0.001	**<0.001**	-0.101	0.003	0.11	0.11	-0.472	0.002	<0.001	**<0.001**	-0.625	0.002	<0.001	**<0.001**
Maternal BMI	0.021	0.000	0.64	0.83	0.129	0.000	0.03	0.40	-0.013	0.000	0.81	0.85	-0.004	0.000	0.92	0.92
IL-6:SES group	0.018	0.003	0.75	0.87	0.122	0.009	0.09	0.35	0.046	0.006	0.48	0.73	0.012	0.008	0.84	0.88
**Inferior Cingulum Bundle**																
IL-6	-0.139	0.002	0.04	0.21	0.010	0.002	0.89	0.92	-0.121	0.002	0.08	0.26	0.042	0.002	0.46	0.69
SES group	-0.061	0.002	0.27	0.46	0.075	0.003	0.22	0.39	0.156	0.004	0.01	**0.04**	0.158	0.003	<0.001	**0.01**
Sex	-0.013	0.002	0.80	0.98	0.011	0.003	0.85	0.98	-0.103	0.003	0.06	0.27	-0.082	0.003	0.07	0.27
NICU stay	-0.100	0.005	0.14	0.96	-0.015	0.007	0.84	0.73	-0.033	0.008	0.64	0.96	0.075	0.006	0.20	0.73
GA at delivery	-0.103	0.001	0.16	0.82	0.121	0.001	0.12	0.82	-0.004	0.001	0.95	0.95	0.117	0.001	0.07	0.82
PMA at scan	0.434	0.001	<0.001	**<0.001**	-0.239	0.001	<0.001	**<0.001**	-0.335	0.001	<0.001	**<0.001**	-0.658	0.001	<0.001	**<0.001**
Maternal BMI	0.070	0.000	0.20	0.63	0.104	0.000	0.08	0.53	0.090	0.000	0.11	0.53	0.022	0.000	0.64	0.83
IL-6:SES group	0.049	0.003	0.46	0.73	0.119	0.004	0.09	0.35	0.028	0.004	0.69	0.85	-0.056	0.003	0.33	0.73
**Fornix**																
IL-6	-0.094	0.001	0.17	0.38	-0.090	0.003	0.21	0.41	-0.163	0.003	0.01	0.18	-0.059	0.002	0.30	0.49
SES group	-0.095	0.002	0.09	0.28	-0.010	0.004	0.88	0.93	0.045	0.004	0.40	0.59	0.099	0.003	0.04	0.15
Sex	-0.088	0.002	0.10	0.29	0.034	0.004	0.56	0.94	-0.074	0.004	0.15	0.38	0.003	0.003	0.94	0.98
NICU stay	0.010	0.004	0.89	0.73	-0.058	0.008	0.43	0.96	0.009	0.008	0.90	0.88	0.011	0.006	0.85	0.96
GA at delivery	0.037	0.001	0.63	0.92	-0.010	0.001	0.90	0.95	0.042	0.001	0.56	0.92	0.006	0.001	0.92	0.95
PMA at scan	0.362	0.001	<0.001	**<0.001**	-0.186	0.001	<0.001	**<0.001**	-0.507	0.001	<0.001	**<0.001**	-0.653	0.001	<0.001	**<0.001**
Maternal BMI	-0.030	0.000	0.59	0.83	0.018	0.000	0.77	0.85	0.053	0.000	0.31	0.64	0.058	0.000	0.21	0.63
IL-6:SES group	0.127	0.002	0.06	0.35	0.144	0.004	0.04	0.35	0.114	0.004	0.08	0.35	-0.019	0.003	0.74	0.87
**IL-8**																

	FA				MD				AD				RD			
	β	SE	*p*	*q*	β	SE	*p*	*q*	β	SE	*p*	*q*	β	SE	*p*	*q*
**Corpus Callosum**																
IL-8	0.015	0.002	0.81	0.94	0.086	0.003	0.26	0.94	0.018	0.003	0.81	0.94	0.008	0.003	0.90	0.94
SES group	0.090	0.003	0.08	0.20	-0.059	0.004	0.33	0.48	0.144	0.004	0.01	0.05	-0.048	0.005	0.33	0.48
Sex	-0.094	0.003	0.06	0.24	-0.056	0.004	0.34	0.73	-0.088	0.003	0.13	0.32	0.039	0.004	0.42	0.82
NICU stay	-0.108	0.007	0.09	0.76	-0.064	0.009	0.38	0.76	0.006	0.008	0.94	0.94	0.029	0.010	0.64	0.92
GA at delivery	0.003	0.001	0.96	0.96	0.008	0.001	0.92	0.96	0.127	0.001	0.11	0.69	-0.015	0.002	0.82	0.96
PMA at scan	0.456	0.001	<0.001	**<0.001**	-0.151	0.001	0.02	**0.02**	-0.239	0.001	<0.001	**<0.001**	-0.560	0.002	<0.001	**<0.001**
Maternal BMI	0.045	0.000	0.39	0.78	0.081	0.000	0.18	0.60	0.034	0.000	0.56	0.84	-0.038	0.000	0.45	0.78
IL-8:SES group	-0.032	0.004	0.62	0.95	-0.061	0.005	0.42	0.95	-0.065	0.004	0.38	0.95	-0.020	0.005	0.75	0.95
**Superior Cingulum Bundle**																
IL-8	-0.044	0.002	0.53	0.94	0.142	0.004	0.06	0.94	0.047	0.004	0.50	0.94	0.083	0.004	0.20	0.94
SES group	-0.135	0.003	0.02	0.05	-0.009	0.005	0.88	0.90	0.056	0.005	0.31	0.48	0.165	0.005	<0.001	**0.01**
Sex	-0.103	0.003	0.06	0.24	0.023	0.005	0.69	0.91	-0.119	0.005	0.03	0.24	0.014	0.005	0.77	0.91
NICU stay	-0.049	0.006	0.48	0.76	-0.160	0.012	0.03	0.43	0.050	0.011	0.47	0.76	0.083	0.011	0.20	0.76
GA at delivery	0.041	0.001	0.59	0.96	-0.023	0.002	0.77	0.96	0.090	0.002	0.23	0.77	0.035	0.002	0.61	0.96
PMA at scan	0.348	0.001	<0.001	**<0.001**	-0.124	0.002	0.05	0.05	-0.418	0.002	<0.001	**<0.001**	-0.516	0.002	<0.001	**<0.001**
Maternal BMI	-0.027	0.000	0.63	0.84	0.043	0.000	0.47	0.78	-0.041	0.000	0.46	0.78	-0.011	0.000	0.82	0.89
IL-8:SES group	0.080	0.003	0.25	0.95	-0.082	0.006	0.28	0.95	-0.005	0.006	0.95	0.96	-0.083	0.006	0.20	0.95
**Corticospinal Tract**																
IL-8	-0.014	0.002	0.83	0.94	0.095	0.004	0.21	0.94	0.032	0.003	0.58	0.94	0.035	0.004	0.53	0.94
SES group	0.066	0.003	0.19	0.36	-0.059	0.006	0.33	0.48	0.228	0.004	<0.001	**<0.001**	0.050	0.005	0.27	0.46
Sex	-0.003	0.003	0.95	0.95	0.018	0.005	0.76	0.91	-0.080	0.003	0.07	0.24	-0.031	0.005	0.48	0.86
NICU stay	0.007	0.007	0.92	0.94	-0.066	0.012	0.36	0.76	0.041	0.008	0.48	0.76	-0.005	0.011	0.93	0.94
GA at delivery	0.058	0.001	0.39	0.96	0.036	0.002	0.65	0.96	0.032	0.001	0.61	0.96	-0.016	0.002	0.79	0.96
PMA at scan	0.521	0.001	<0.001	**<0.001**	-0.208	0.002	<0.001	**<0.001**	-0.651	0.001	<0.001	**<0.001**	-0.671	0.002	<0.001	**<0.001**
Maternal BMI	0.036	0.000	0.48	0.78	0.056	0.000	0.35	0.78	-0.017	0.000	0.71	0.89	-0.032	0.000	0.47	0.78
IL-8:SES group	-0.048	0.004	0.44	0.95	-0.021	0.007	0.78	0.95	-0.063	0.004	0.28	0.95	0.008	0.006	0.88	0.96
**Optic Radiation**																
IL-8	0.008	0.002	0.90	0.94	0.000	0.005	1.00	1.00	0.014	0.004	0.84	0.94	0.019	0.004	0.73	0.94
SES group	0.084	0.003	0.09	0.20	0.004	0.007	0.95	0.95	0.266	0.005	<0.001	**<0.001**	0.084	0.006	0.06	0.17
Sex	-0.018	0.003	0.71	0.91	-0.017	0.006	0.77	0.91	-0.205	0.005	<0.001	**<0.001**	-0.085	0.005	0.05	0.24
NICU stay	-0.160	0.007	0.01	0.34	-0.068	0.014	0.35	0.76	-0.005	0.011	0.94	0.94	0.119	0.012	0.04	0.43
GA at delivery	-0.028	0.001	0.67	0.96	0.036	0.002	0.65	0.96	-0.057	0.002	0.43	0.96	-0.026	0.002	0.68	0.96
PMA at scan	0.535	0.001	<0.001	**<0.001**	-0.179	0.002	<0.001	**0.01**	-0.340	0.002	<0.001	**<0.001**	-0.621	0.002	<0.001	**<0.001**
Maternal BMI	0.003	0.000	0.95	0.95	0.136	0.000	0.02	0.44	0.133	0.000	0.01	0.44	0.072	0.000	0.11	0.58
IL-8:SES group	-0.031	0.004	0.62	0.95	0.077	0.008	0.30	0.95	-0.018	0.006	0.79	0.95	0.003	0.007	0.96	0.96
**Uncinate Fasciculus**																
IL-8	0.032	0.002	0.61	0.94	0.087	0.004	0.26	0.94	0.014	0.004	0.85	0.94	-0.018	0.003	0.76	0.94
SES group	-0.019	0.003	0.70	0.83	0.016	0.005	0.80	0.86	0.142	0.005	0.01	0.05	0.115	0.005	0.02	0.05
Sex	-0.083	0.002	0.08	0.24	0.014	0.005	0.81	0.91	-0.125	0.005	0.02	0.24	-0.018	0.004	0.70	0.91
NICU stay	-0.058	0.006	0.35	0.76	-0.008	0.011	0.91	0.94	-0.071	0.011	0.32	0.76	0.006	0.010	0.91	0.94
GA at delivery	0.096	0.001	0.15	0.69	0.091	0.002	0.25	0.77	0.140	0.002	0.07	0.69	0.018	0.002	0.78	0.96

	FA β	FA SE	FA p	FA q	MD β	MD SE	MD p	MD q	AD β	AD SE	AD p	AD q	RD β	RD SE	RD p	RD q
PMA at scan	0.505	0.001	<0.001	**<0.001**	-0.096	0.002	0.13	0.13	-0.309	0.002	<0.001	**<0.001**	-0.636	0.002	<0.001	**<0.001**
Maternal BMI	0.041	0.000	0.42	0.78	0.082	0.000	0.18	0.60	0.043	0.000	0.45	0.78	0.013	0.000	0.78	0.89
IL-8:SES group	0.033	0.003	0.60	0.95	-0.048	0.006	0.53	0.95	-0.070	0.006	0.33	0.95	-0.072	0.005	0.23	0.95
**Inferior Fronto-Occipital Fasciculus**																
IL-8	0.016	0.002	0.78	0.94	0.072	0.005	0.34	0.94	0.015	0.003	0.82	0.94	-0.009	0.004	0.86	0.94
SES group	0.018	0.003	0.69	0.83	-0.014	0.007	0.82	0.86	0.219	0.004	<0.001	**<0.001**	0.072	0.006	0.10	0.22
Sex	0.006	0.003	0.88	0.93	-0.007	0.006	0.90	0.93	-0.168	0.003	<0.001	**0.02**	-0.076	0.006	0.07	0.24
NICU stay	-0.078	0.007	0.17	0.76	-0.061	0.014	0.40	0.76	0.036	0.008	0.59	0.88	0.068	0.013	0.21	0.76
GA at delivery	0.078	0.001	0.20	0.77	-0.008	0.002	0.92	0.96	0.107	0.001	0.13	0.69	-0.034	0.002	0.56	0.96
PMA at scan	0.617	0.001	<0.001	**<0.001**	-0.123	0.002	0.05	0.06	-0.481	0.001	<0.001	**<0.001**	-0.675	0.002	<0.001	**<0.001**
Maternal BMI	0.015	0.000	0.74	0.89	0.116	0.000	0.06	0.50	0.076	0.000	0.15	0.60	0.021	0.000	0.63	0.84
IL-8:SES group	0.010	0.004	0.86	0.96	-0.020	0.008	0.79	0.95	-0.024	0.004	0.71	0.95	-0.018	0.007	0.75	0.95
**Anterior Limb of Internal Capsule**																
IL-8	-0.024	0.002	0.67	0.94	0.098	0.006	0.20	0.94	-0.030	0.004	0.66	0.94	0.020	0.005	0.73	0.94
SES group	0.029	0.003	0.51	0.66	0.016	0.007	0.79	0.86	0.165	0.005	<0.001	**0.01**	0.073	0.007	0.11	0.24
Sex	-0.022	0.002	0.61	0.91	-0.023	0.007	0.69	0.91	-0.062	0.005	0.24	0.54	-0.029	0.006	0.52	0.90
NICU stay	-0.043	0.006	0.44	0.76	-0.083	0.016	0.25	0.76	0.061	0.011	0.37	0.76	0.064	0.014	0.27	0.76
GA at delivery	0.130	0.001	0.03	0.69	-0.028	0.003	0.73	0.96	0.111	0.002	0.13	0.69	-0.030	0.002	0.63	0.96
PMA at scan	0.591	0.001	<0.001	**<0.001**	-0.099	0.003	0.12	0.12	-0.471	0.002	<0.001	**<0.001**	-0.624	0.002	<0.001	**<0.001**
Maternal BMI	0.024	0.000	0.59	0.84	0.123	0.000	0.04	0.50	-0.011	0.000	0.84	0.89	-0.006	0.000	0.89	0.91
IL-8:SES group	0.017	0.003	0.76	0.95	-0.086	0.009	0.26	0.95	-0.018	0.006	0.79	0.95	-0.030	0.008	0.61	0.95
**Inferior Cingulum Bundle**																
IL-8	-0.077	0.002	0.27	0.94	0.023	0.002	0.76	0.94	-0.098	0.003	0.17	0.94	-0.007	0.002	0.91	0.94
SES group	-0.040	0.002	0.46	0.61	0.052	0.003	0.39	0.54	0.185	0.004	<0.001	**0.01**	0.166	0.003	<0.001	**<0.001**
Sex	-0.015	0.002	0.78	0.91	0.022	0.003	0.70	0.91	-0.112	0.003	0.04	0.24	-0.088	0.003	0.05	0.24
NICU stay	-0.093	0.005	0.17	0.76	-0.017	0.007	0.81	0.94	-0.026	0.008	0.71	0.94	0.075	0.006	0.20	0.76
GA at delivery	-0.084	0.001	0.26	0.77	0.114	0.001	0.14	0.69	0.016	0.001	0.83	0.96	0.114	0.001	0.07	0.69
PMA at scan	0.431	0.001	<0.001	**<0.001**	-0.239	0.001	<0.001	**<0.001**	-0.339	0.001	<0.001	**<0.001**	-0.659	0.001	<0.001	**<0.001**
Maternal BMI	0.074	0.000	0.18	0.60	0.108	0.000	0.07	0.50	0.097	0.000	0.08	0.50	0.024	0.000	0.62	0.84
IL-8:SES group	0.101	0.003	0.14	0.95	0.020	0.004	0.79	0.95	0.102	0.004	0.15	0.95	-0.010	0.003	0.86	0.96
**Fornix**																
IL-8	-0.041	0.001	0.56	0.94	0.092	0.003	0.23	0.94	-0.029	0.003	0.66	0.94	0.026	0.002	0.65	0.94
SES group	-0.086	0.002	0.12	0.24	-0.017	0.004	0.78	0.86	0.064	0.004	0.22	0.40	0.109	0.003	0.02	0.06
Sex	-0.093	0.002	0.08	0.24	0.046	0.004	0.43	0.82	-0.077	0.004	0.13	0.32	0.007	0.003	0.88	0.93
NICU stay	0.011	0.004	0.87	0.94	-0.065	0.008	0.38	0.76	0.011	0.009	0.87	0.94	0.011	0.006	0.85	0.94
GA at delivery	0.053	0.001	0.49	0.96	-0.005	0.001	0.95	0.96	0.065	0.001	0.37	0.96	0.012	0.001	0.85	0.96
PMA at scan	0.350	0.001	<0.001	**<0.001**	-0.182	0.001	<0.001	**0.01**	-0.508	0.001	<0.001	**<0.001**	-0.650	0.001	<0.001	**<0.001**
Maternal BMI	-0.017	0.000	0.76	0.89	0.015	0.000	0.80	0.89	0.061	0.000	0.25	0.74	0.052	0.000	0.27	0.74
IL-8:SES group	0.027	0.002	0.70	0.95	-0.049	0.004	0.52	0.95	0.043	0.005	0.52	0.95	0.003	0.003	0.96	0.96

IL-10

	**FA**				**MD**				**AD**				**RD**			
	**β**	**SE**	***p***	***q***	**β**	**SE**	***p***	***q***	**β**	**SE**	***p***	***q***	**β**	**SE**	***p***	***q***
**Corpus Callosum**																
IL-10	-0.077	0.002	0.22	0.46	0.019	0.003	0.80	0.92	-0.059	0.003	0.42	0.63	0.106	0.003	0.07	0.24

SES group	0.093	0.003	0.07	0.18	-0.045	0.004	0.46	0.64	0.142	0.004	0.02	0.08	-0.049	0.004	0.31	0.51
Sex	-0.098	0.003	0.05	0.23	-0.048	0.004	0.41	0.78	-0.083	0.003	0.16	0.36	0.045	0.004	0.34	0.73
NICU stay	-0.146	0.007	0.02	0.23	-0.044	0.009	0.55	0.77	-0.010	0.008	0.89	0.99	0.063	0.010	0.29	0.71
GA at delivery	0.005	0.001	0.94	0.96	0.011	0.001	0.89	0.94	0.118	0.001	0.14	0.72	-0.015	0.002	0.82	0.94
PMA at scan	0.462	0.001	<0.001	**<0.001**	-0.150	0.001	0.02	**0.02**	-0.230	0.001	<0.001	**<0.001**	-0.560	0.002	<0.001	**<0.001**
Maternal BMI	0.052	0.000	0.31	0.73	0.088	0.000	0.15	0.62	0.038	0.000	0.52	0.77	-0.041	0.000	0.39	0.77
IL-10:SES group	-0.102	0.004	0.10	0.86	0.099	0.005	0.18	0.86	-0.011	0.004	0.88	0.98	0.070	0.005	0.24	0.86
**Superior Cingulum Bundle**																
IL-10	-0.057	0.002	0.41	0.63	0.068	0.004	0.36	0.62	0.011	0.004	0.87	0.92	0.037	0.004	0.56	0.74
SES group	-0.109	0.003	0.06	0.16	0.004	0.005	0.95	0.97	0.063	0.005	0.27	0.46	0.151	0.005	<0.001	**0.03**
Sex	-0.085	0.003	0.12	0.32	0.022	0.005	0.70	0.92	-0.144	0.005	0.01	0.10	-0.020	0.005	0.70	0.92
NICU stay	-0.043	0.006	0.55	0.77	-0.136	0.012	0.07	0.39	0.067	0.012	0.33	0.75	0.085	0.011	0.19	0.67
GA at delivery	0.023	0.001	0.76	0.94	-0.020	0.002	0.80	0.94	0.096	0.002	0.20	0.72	0.057	0.002	0.41	0.93
PMA at scan	0.351	0.001	<0.001	**<0.001**	-0.124	0.002	0.05	0.05	-0.423	0.002	<0.001	**<0.001**	-0.526	0.002	<0.001	**<0.001**
Maternal BMI	-0.033	0.000	0.56	0.78	0.059	0.000	0.33	0.73	-0.020	0.000	0.72	0.85	0.010	0.000	0.84	0.87
IL-10:SES group	0.091	0.004	0.19	0.86	0.050	0.006	0.50	0.97	0.001	0.006	0.98	0.98	-0.063	0.006	0.32	0.97
**Corticospinal Tract**																
IL-10	-0.278	0.002	<0.001	**<0.001**	0.105	0.004	0.15	0.38	-0.004	0.003	0.94	0.94	0.220	0.004	<0.001	**<0.001**
SES group	0.046	0.003	0.33	0.51	-0.038	0.006	0.52	0.70	0.225	0.004	<0.001	**<0.001**	0.064	0.005	0.14	0.26
Sex	-0.016	0.003	0.72	0.92	0.023	0.005	0.69	0.92	-0.087	0.003	0.06	0.23	-0.025	0.005	0.55	0.89
NICU stay	-0.062	0.006	0.28	0.71	-0.040	0.012	0.58	0.78	0.036	0.008	0.54	0.77	0.038	0.011	0.47	0.77
GA at delivery	0.066	0.001	0.28	0.72	0.031	0.002	0.69	0.94	0.039	0.001	0.53	0.94	-0.030	0.002	0.59	0.94
PMA at scan	0.522	0.001	<0.001	**<0.001**	-0.200	0.002	<0.001	**<0.001**	-0.647	0.001	<0.001	**<0.001**	-0.663	0.002	<0.001	**<0.001**
Maternal BMI	0.034	0.000	0.47	0.77	0.070	0.000	0.24	0.72	-0.024	0.000	0.60	0.80	-0.034	0.000	0.43	0.77
IL-10:SES group	-0.031	0.003	0.59	0.97	0.075	0.007	0.30	0.97	-0.038	0.004	0.51	0.97	-0.003	0.006	0.96	0.98
**Optic Radiation**																
IL-10	-0.096	0.002	0.11	0.31	0.120	0.005	0.10	0.30	0.160	0.004	0.01	0.08	0.159	0.004	<0.001	0.05
SES group	0.077	0.003	0.12	0.25	0.015	0.007	0.81	0.88	0.255	0.005	<0.001	**<0.001**	0.085	0.006	0.06	0.16
Sex	-0.010	0.003	0.83	0.94	-0.013	0.006	0.82	0.94	-0.194	0.005	<0.001	**0.01**	-0.084	0.005	0.05	0.23
NICU stay	-0.185	0.007	<0.001	0.10	-0.060	0.014	0.41	0.77	0.017	0.011	0.80	0.99	0.150	0.013	0.01	0.13
GA at delivery	-0.031	0.001	0.63	0.94	0.023	0.002	0.77	0.94	-0.070	0.002	0.32	0.77	-0.029	0.002	0.63	0.94
PMA at scan	0.538	0.001	<0.001	**<0.001**	-0.172	0.002	0.01	**0.01**	-0.350	0.002	<0.001	**<0.001**	-0.623	0.002	<0.001	**<0.001**
Maternal BMI	0.010	0.000	0.84	0.87	0.135	0.000	0.02	0.33	0.117	0.000	0.03	0.33	0.060	0.000	0.18	0.66
IL-10:SES group	0.010	0.004	0.87	0.98	-0.019	0.008	0.79	0.98	-0.131	0.006	0.04	0.80	-0.071	0.007	0.19	0.86
**Uncinate Fasciculus**																
IL-10	-0.054	0.002	0.39	0.63	-0.025	0.004	0.73	0.92	-0.125	0.004	0.07	0.24	-0.066	0.003	0.27	0.53
SES group	-0.024	0.003	0.65	0.75	0.025	0.005	0.68	0.77	0.128	0.005	0.03	0.08	0.109	0.005	0.03	0.08
Sex	-0.088	0.003	0.08	0.25	0.010	0.005	0.86	0.94	-0.136	0.005	0.01	0.13	-0.024	0.004	0.61	0.92
NICU stay	-0.069	0.006	0.27	0.71	-0.001	0.011	0.99	0.99	-0.083	0.012	0.24	0.71	0.002	0.010	0.97	0.99
GA at delivery	0.084	0.001	0.22	0.72	0.091	0.002	0.25	0.72	0.150	0.002	0.05	0.59	0.036	0.002	0.57	0.94
PMA at scan	0.495	0.001	<0.001	**<0.001**	-0.093	0.002	0.15	0.15	-0.310	0.002	<0.001	**<0.001**	-0.631	0.002	<0.001	**<0.001**
Maternal BMI	0.039	0.000	0.44	0.77	0.095	0.000	0.12	0.62	0.046	0.000	0.42	0.77	0.017	0.000	0.73	0.85
IL-10:SES group	0.003	0.003	0.96	0.98	0.037	0.006	0.62	0.97	-0.029	0.006	0.68	0.97	-0.017	0.006	0.77	0.98
**Inferior Fronto-Occipital Fasciculus**																

IL-10	-0.136	0.002	0.01	0.08	0.056	0.005	0.45	0.64	-0.014	0.003	0.82	0.92	0.097	0.004	0.07	0.24
SES group	0.020	0.003	0.64	0.75	0.002	0.007	0.98	0.98	0.209	0.004	<0.001	**<0.001**	0.068	0.006	0.12	0.25
Sex	0.002	0.003	0.95	0.98	-0.002	0.006	0.98	0.98	-0.175	0.004	<0.001	**0.01**	-0.076	0.006	0.07	0.25
NICU stay	-0.116	0.007	0.03	0.30	-0.043	0.014	0.56	0.77	0.042	0.008	0.52	0.77	0.102	0.013	0.06	0.39
GA at delivery	0.065	0.001	0.26	0.72	-0.012	0.002	0.88	0.94	0.106	0.001	0.13	0.72	-0.023	0.002	0.68	0.94
PMA at scan	0.623	0.001	<0.001	**<0.001**	-0.118	0.002	0.06	0.07	-0.476	0.001	<0.001	**<0.001**	-0.678	0.002	<0.001	**<0.001**
Maternal BMI	0.029	0.000	0.50	0.77	0.124	0.000	0.04	0.37	0.076	0.000	0.15	0.62	0.011	0.000	0.81	0.87
IL-10:SES group	-0.005	0.004	0.92	0.98	0.061	0.008	0.41	0.97	-0.019	0.005	0.76	0.98	0.005	0.007	0.93	0.98
**Anterior Limb of Internal Capsule**																
IL-10	-0.143	0.002	0.01	0.08	0.023	0.005	0.76	0.92	-0.090	0.004	0.18	0.42	0.041	0.005	0.47	0.66
SES group	0.025	0.003	0.58	0.74	0.031	0.008	0.61	0.75	0.157	0.005	<0.001	**0.03**	0.074	0.007	0.12	0.25
Sex	-0.031	0.002	0.48	0.86	-0.019	0.007	0.74	0.92	-0.075	0.005	0.16	0.36	-0.031	0.006	0.50	0.86
NICU stay	-0.070	0.006	0.20	0.67	-0.062	0.016	0.40	0.77	0.060	0.011	0.38	0.77	0.082	0.015	0.16	0.65
GA at delivery	0.127	0.001	0.03	0.59	-0.023	0.003	0.77	0.94	0.117	0.002	0.11	0.72	-0.026	0.002	0.68	0.94
PMA at scan	0.588	0.001	<0.001	**<0.001**	-0.096	0.003	0.13	0.13	-0.469	0.002	<0.001	**<0.001**	-0.621	0.002	<0.001	**<0.001**
Maternal BMI	0.029	0.000	0.52	0.77	0.137	0.000	0.02	0.33	-0.010	0.000	0.86	0.87	-0.008	0.000	0.87	0.87
IL-10:SES group	0.001	0.003	0.98	0.98	0.088	0.009	0.23	0.86	0.049	0.006	0.47	0.97	0.025	0.008	0.66	0.97
**Inferior Cingulum Bundle**																
IL-10	-0.019	0.002	0.78	0.92	0.092	0.002	0.21	0.46	0.143	0.003	0.04	0.18	0.131	0.002	0.02	0.13
SES group	-0.043	0.002	0.44	0.63	0.057	0.003	0.34	0.51	0.159	0.004	0.01	**0.03**	0.147	0.003	<0.001	**0.02**
Sex	-0.010	0.002	0.85	0.94	0.021	0.003	0.72	0.92	-0.110	0.003	0.05	0.23	-0.097	0.003	0.04	0.23
NICU stay	-0.108	0.005	0.12	0.61	-0.008	0.007	0.92	0.99	-0.027	0.008	0.70	0.90	0.088	0.007	0.14	0.61
GA at delivery	-0.098	0.001	0.18	0.72	0.111	0.001	0.15	0.72	0.013	0.001	0.87	0.94	0.130	0.001	0.04	0.59
PMA at scan	0.423	0.001	<0.001	**<0.001**	-0.233	0.001	<0.001	**<0.001**	-0.327	0.001	<0.001	**<0.001**	-0.649	0.001	<0.001	**<0.001**
Maternal BMI	0.057	0.000	0.31	0.73	0.113	0.000	0.06	0.43	0.083	0.000	0.15	0.62	0.029	0.000	0.54	0.77
IL-10:SES group	-0.063	0.003	0.36	0.97	-0.035	0.004	0.63	0.97	-0.164	0.004	0.02	0.67	-0.079	0.004	0.17	0.86
**Fornix**																
IL-10	-0.075	0.001	0.28	0.53	-0.009	0.003	0.90	0.93	-0.013	0.003	0.84	0.92	0.053	0.002	0.36	0.62
SES group	-0.088	0.002	0.13	0.25	-0.006	0.004	0.92	0.97	0.062	0.004	0.25	0.46	0.110	0.003	0.02	0.08
Sex	-0.088	0.002	0.11	0.31	0.048	0.004	0.41	0.78	-0.089	0.004	0.09	0.28	-0.007	0.003	0.89	0.94
NICU stay	0.005	0.004	0.95	0.99	-0.051	0.008	0.49	0.77	0.006	0.009	0.93	0.99	0.013	0.007	0.82	0.99
GA at delivery	0.056	0.001	0.46	0.94	-0.004	0.001	0.96	0.96	0.078	0.001	0.28	0.72	0.018	0.001	0.78	0.94
PMA at scan	0.346	0.001	<0.001	**<0.001**	-0.182	0.001	<0.001	**0.01**	-0.501	0.001	<0.001	**<0.001**	-0.645	0.001	<0.001	**<0.001**
Maternal BMI	-0.022	0.000	0.70	0.85	0.025	0.000	0.68	0.85	0.060	0.000	0.26	0.73	0.057	0.000	0.23	0.72
IL-10:SES group	0.054	0.002	0.43	0.97	0.092	0.004	0.21	0.86	0.029	0.005	0.65	0.97	-0.031	0.004	0.60	0.97

TNF-α

	**FA**				**MD**				**AD**				**RD**			
	**β**	**SE**	***p***	***q***	**β**	**SE**	***p***	***q***	**β**	**SE**	***p***	***q***	**β**	**SE**	***p***	***q***
**Corpus Callosum**																
TNF-α	0.058	0.003	0.37	0.76	0.039	0.003	0.59	0.82	-0.114	0.003	0.12	0.43	-0.075	0.003	0.22	0.59
SES group	0.096	0.003	0.06	0.17	-0.053	0.004	0.39	0.56	0.156	0.004	0.01	**0.03**	-0.049	0.005	0.32	0.50
Sex	-0.099	0.003	0.05	0.19	-0.062	0.004	0.30	0.63	-0.099	0.003	0.08	0.21	0.039	0.004	0.41	0.83
NICU stay	-0.112	0.007	0.08	0.71	-0.061	0.009	0.41	0.71	0.007	0.008	0.93	0.99	0.035	0.010	0.57	0.85
GA at delivery	0.006	0.001	0.93	0.96	0.011	0.001	0.89	0.96	0.121	0.001	0.13	0.71	-0.019	0.002	0.78	0.96

PMA at scan	0.451	0.001	<0.001	**<0.001**	-0.158	0.001	0.01	**0.02**	-0.240	0.001	<0.001	**<0.001**	-0.559	0.002	<0.001	**<0.001**
Maternal BMI	0.051	0.000	0.32	0.71	0.090	0.000	0.14	0.51	0.040	0.000	0.50	0.78	-0.038	0.000	0.44	0.71
TNFα:SES group	-0.070	0.004	0.27	0.62	-0.042	0.005	0.57	0.75	0.071	0.004	0.33	0.64	0.055	0.005	0.37	0.64
**Superior Cingulum Bundle**																
TNF-α	0.005	0.002	0.94	0.99	0.048	0.004	0.52	0.76	-0.122	0.004	0.07	0.33	-0.082	0.004	0.20	0.59
SES group	-0.134	0.003	0.02	0.05	-0.004	0.005	0.95	0.95	0.058	0.005	0.29	0.47	0.167	0.005	<0.01	**0.01**
Sex	-0.106	0.003	0.05	0.19	0.016	0.005	0.78	0.90	-0.126	0.005	0.02	0.17	0.012	0.005	0.81	0.90
NICU stay	-0.056	0.006	0.43	0.71	-0.154	0.012	0.04	0.48	0.056	0.011	0.41	0.71	0.093	0.011	0.15	0.71
GA at delivery	0.036	0.001	0.64	0.96	-0.021	0.002	0.79	0.96	0.087	0.002	0.24	0.84	0.038	0.002	0.59	0.96
PMA at scan	0.350	0.001	<0.001	**<0.001**	-0.134	0.002	0.04	**0.04**	-0.409	0.002	<0.001	**<0.001**	-0.513	0.002	<0.001	**<0.001**
Maternal BMI	-0.031	0.000	0.58	0.87	0.054	0.000	0.37	0.71	-0.043	0.000	0.43	0.71	-0.008	0.000	0.87	0.90
TNFα:SES group	0.013	0.004	0.85	0.92	-0.028	0.007	0.71	0.82	0.228	0.006	0.00	0.03	0.128	0.006	0.05	0.27
**Corticospinal Tract**																
TNF-α	-0.022	0.002	0.73	0.90	0.063	0.004	0.39	0.76	-0.069	0.003	0.23	0.59	-0.017	0.004	0.76	0.91
SES group	0.070	0.003	0.16	0.31	-0.048	0.006	0.43	0.59	0.229	0.004	<0.001	**<0.001**	0.048	0.005	0.28	0.47
Sex	-0.008	0.003	0.87	0.90	0.011	0.005	0.85	0.90	-0.083	0.003	0.06	0.19	-0.029	0.005	0.51	0.87
NICU stay	0.002	0.007	0.98	0.99	-0.068	0.012	0.35	0.71	0.044	0.008	0.45	0.71	0.001	0.011	0.98	0.99
GA at delivery	0.056	0.001	0.41	0.96	0.030	0.002	0.70	0.96	0.029	0.001	0.64	0.96	-0.015	0.002	0.80	0.96
PMA at scan	0.520	0.001	<0.001	**<0.001**	-0.217	0.002	<0.001	**<0.001**	-0.649	0.001	<0.001	**<0.001**	-0.670	0.002	<0.001	**<0.001**
Maternal BMI	0.042	0.000	0.40	0.71	0.064	0.000	0.29	0.71	-0.013	0.000	0.78	0.87	-0.035	0.000	0.43	0.71
TNFα:SES group	-0.023	0.004	0.71	0.82	-0.101	0.007	0.17	0.54	0.051	0.004	0.37	0.64	0.048	0.006	0.39	0.65
**Optic Radiation**																
TNF-α	-0.039	0.002	0.52	0.76	-0.001	0.005	0.99	0.99	0.001	0.004	0.99	0.99	0.022	0.004	0.70	0.90
SES group	0.090	0.003	0.07	0.17	0.016	0.007	0.80	0.90	0.266	0.005	<0.001	**<0.001**	0.081	0.006	0.07	0.18
Sex	-0.023	0.003	0.64	0.90	-0.016	0.006	0.79	0.90	-0.211	0.005	<0.001	**<0.001**	-0.085	0.005	0.05	0.19
NICU stay	-0.161	0.007	0.01	0.34	-0.070	0.014	0.34	0.71	-0.011	0.011	0.87	0.99	0.117	0.013	0.04	0.48
GA at delivery	-0.031	0.001	0.65	0.96	0.028	0.002	0.72	0.96	-0.060	0.002	0.40	0.96	-0.027	0.002	0.66	0.96
PMA at scan	0.534	0.001	<0.001	**<0.001**	-0.173	0.002	0.01	**0.01**	-0.343	0.002	<0.001	**<0.001**	-0.622	0.002	<0.001	**<0.001**
Maternal BMI	0.006	0.000	0.90	0.90	0.131	0.000	0.03	0.37	0.137	0.000	0.01	0.35	0.073	0.000	0.11	0.51
TNFα:SES group	0.010	0.004	0.87	0.92	-0.025	0.008	0.73	0.82	0.005	0.006	0.94	0.96	0.000	0.007	1.00	1.00
**Uncinate Fasciculus**																
TNF-α	-0.145	0.002	0.02	0.14	0.070	0.004	0.34	0.76	-0.118	0.004	0.09	0.38	0.039	0.003	0.51	0.76
SES group	-0.008	0.003	0.86	0.94	0.023	0.005	0.71	0.82	0.154	0.005	0.01	**0.03**	0.114	0.005	0.02	0.05
Sex	-0.089	0.002	0.06	0.19	0.012	0.005	0.84	0.90	-0.125	0.005	0.02	0.17	-0.013	0.004	0.77	0.90
NICU stay	-0.057	0.006	0.36	0.71	-0.008	0.011	0.91	0.99	-0.066	0.011	0.35	0.71	0.009	0.010	0.89	0.99
GA at delivery	0.074	0.001	0.27	0.84	0.085	0.002	0.28	0.84	0.130	0.002	0.09	0.71	0.029	0.002	0.65	0.96
PMA at scan	0.505	0.001	<0.001	**<0.001**	-0.107	0.002	0.09	0.09	-0.310	0.002	<0.001	**<0.001**	-0.636	0.002	<0.001	**<0.001**
Maternal BMI	0.038	0.000	0.44	0.71	0.090	0.000	0.14	0.51	0.047	0.000	0.40	0.71	0.018	0.000	0.71	0.87
TNFα:SES group	0.034	0.003	0.58	0.75	-0.153	0.006	0.04	0.27	-0.054	0.006	0.44	0.67	-0.068	0.005	0.25	0.60
**Inferior Fronto-Occipital Fasciculus**																
TNF-α	0.035	0.002	0.53	0.76	0.027	0.005	0.71	0.90	-0.075	0.003	0.25	0.60	-0.069	0.004	0.20	0.59
SES group	0.019	0.003	0.66	0.80	-0.004	0.007	0.95	0.95	0.222	0.004	<0.001	**<0.001**	0.073	0.006	0.09	0.20
Sex	0.003	0.003	0.94	0.94	-0.009	0.006	0.87	0.90	-0.175	0.003	<0.001	**0.01**	-0.076	0.006	0.07	0.20

NICU stay	-0.083	0.007	0.14	0.71	-0.059	0.014	0.42	0.71	0.035	0.008	0.59	0.85	0.072	0.013	0.19	0.71
GA at delivery	0.074	0.001	0.22	0.84	-0.014	0.002	0.86	0.96	0.102	0.001	0.15	0.71	-0.034	0.002	0.56	0.96
PMA at scan	0.612	0.001	<0.001	**<0.001**	-0.128	0.002	0.04	0.05	-0.478	0.001	<0.001	**<0.001**	-0.669	0.002	<0.001	**<0.001**
Maternal BMI	0.018	0.000	0.69	0.87	0.121	0.000	0.05	0.43	0.078	0.000	0.14	0.51	0.019	0.000	0.66	0.87
TNFα:SES group	-0.055	0.004	0.32	0.64	-0.091	0.008	0.22	0.56	0.089	0.004	0.17	0.54	0.079	0.007	0.15	0.54
**Anterior Limb of Internal Capsule**																
TNF-α	0.044	0.002	0.43	0.76	0.002	0.006	0.98	0.99	-0.164	0.004	0.01	0.13	-0.122	0.005	0.03	0.20
SES group	0.028	0.003	0.54	0.72	0.027	0.008	0.66	0.80	0.168	0.005	<0.001	**0.01**	0.077	0.007	0.09	0.20
Sex	-0.019	0.002	0.65	0.90	-0.027	0.007	0.64	0.90	-0.064	0.005	0.22	0.49	-0.032	0.006	0.47	0.85
NICU stay	-0.044	0.006	0.43	0.71	-0.077	0.016	0.29	0.71	0.066	0.011	0.33	0.71	0.068	0.014	0.24	0.71
GA at delivery	0.134	0.001	0.03	0.71	-0.031	0.003	0.69	0.96	0.103	0.002	0.16	0.71	-0.038	0.002	0.55	0.96
PMA at scan	0.589	0.001	<0.001	**<0.001**	-0.107	0.003	0.09	0.09	-0.462	0.002	<0.001	**<0.001**	-0.618	0.002	<0.001	**<0.001**
Maternal BMI	0.023	0.000	0.61	0.87	0.132	0.000	0.03	0.37	-0.014	0.000	0.80	0.87	-0.007	0.000	0.89	0.90
TNFα:SES group	-0.042	0.003	0.45	0.67	-0.102	0.009	0.16	0.54	0.137	0.006	0.04	0.27	0.100	0.008	0.08	0.42
**Inferior Cingulum Bundle**																
TNF-α	-0.180	0.002	0.01	0.10	0.051	0.002	0.48	0.76	-0.213	0.003	<0.01	0.07	0.010	0.002	0.87	0.98
SES group	-0.031	0.002	0.56	0.72	0.058	0.003	0.33	0.50	0.191	0.003	<0.01	**<0.001**	0.162	0.003	<0.001	**<0.001**
Sex	-0.024	0.002	0.65	0.90	0.024	0.003	0.68	0.90	-0.121	0.003	0.02	0.17	-0.087	0.003	0.06	0.19
NICU stay	-0.101	0.005	0.14	0.71	-0.014	0.007	0.85	0.99	-0.030	0.008	0.66	0.91	0.077	0.006	0.19	0.71
GA at delivery	-0.110	0.001	0.14	0.71	0.119	0.001	0.13	0.71	-0.003	0.001	0.96	0.96	0.121	0.001	0.06	0.71
PMA at scan	0.440	0.001	<0.001	**<0.001**	-0.236	0.001	<0.001	**<0.001**	-0.325	0.001	<0.01	**<0.001**	-0.654	0.001	<0.001	**<0.001**
Maternal BMI	0.067	0.000	0.22	0.71	0.108	0.000	0.07	0.51	0.087	0.000	0.11	0.51	0.023	0.000	0.63	0.87
TNFα:SES group	0.091	0.003	0.18	0.54	-0.041	0.004	0.58	0.75	0.207	0.004	<0.001	**0.04**	0.055	0.004	0.35	0.64
**Fornix**																
TNF-α	-0.016	0.001	0.81	0.95	0.047	0.003	0.53	0.76	-0.179	0.003	0.01	0.10	-0.118	0.002	0.04	0.22
SES group	-0.087	0.002	0.12	0.24	-0.006	0.004	0.92	0.95	0.067	0.004	0.20	0.36	0.112	0.003	0.02	0.05
Sex	-0.104	0.002	0.06	0.19	0.045	0.004	0.45	0.85	-0.082	0.004	0.11	0.26	0.011	0.003	0.81	0.90
NICU stay	-0.001	0.004	0.99	0.99	-0.055	0.008	0.45	0.71	0.013	0.009	0.85	0.99	0.022	0.006	0.71	0.94
GA at delivery	0.045	0.001	0.55	0.96	0.004	0.001	0.96	0.96	0.050	0.001	0.48	0.96	0.005	0.001	0.94	0.96
PMA at scan	0.350	0.001	<0.001	**<0.001**	-0.184	0.001	<0.001	**<0.001**	-0.498	0.001	<0.001	**<0.001**	-0.647	0.001	<0.001	**<0.001**
Maternal BMI	-0.015	0.000	0.79	0.87	0.020	0.000	0.75	0.87	0.054	0.000	0.30	0.71	0.046	0.000	0.32	0.71
TNFα:SES group	0.034	0.002	0.62	0.77	-0.048	0.005	0.51	0.73	0.171	0.005	0.01	0.11	0.074	0.004	0.20	0.55

β, standardized beta coefficient; SE, standard error; *q*, FDR-corrected p-value; IL, interleukin; TNF-α, tumor necrosis factor alpha; FA, fractional anisotropy; MD, mean diffusivity; AD, axial diffusivity; RD, radial diffusivity; GA, gestational age; PMA, infant postmenstrual age; SES, socioeconomic status.

The relationship between maternal cytokine concentrations by trimester and neonatal dMRI parameters were examined with multiple linear regressions. As shown in Table S2, there was a negative relationship between maternal IL-6 at T2 and FA in the CST and OR. At T3, there was a negative relationship between maternal IL-6 and 1) FA in the CST; 2) MD in the UF and FX; and 3) AD in the CST, UF, IFOF, ALIC, CBIF, and FX. For maternal IL-10 at T2, there was a 1) negative relationship with FA in the CC, CST, IFOF, ALIC, and CBIF; 2) a positive relationship with MD in the CST; 3) and a positive relationship with RD in the CC, CST, OR, and CBIF. For maternal IL-10 at T3, there was a negative relationship FA in the CST and AD in the UF, but a positive relationship with RD in the CST. For maternal TNF-α at T2, there was a negative relationship with FA in the CBIF.

Table S4. Multiple linear regression results of the association between maternal cytokine concentrations by trimester and neonatal dMRI parameters.

	**FA**				**MD**				**AD**				**RD**			
	**β**	**SE**	***p***	***q***	**β**	**SE**	***p***	***q***	**β**	**SE**	***p***	***q***	**β**	**SE**	***p***	***q***
**IL-6 Trimester 1**																
**Corpus Callosum**																
IL-6 T1	-0.070	0.002	0.27	0.79	0.065	0.002	0.29	0.66	-0.025	0.002	0.73	0.74	0.069	0.002	0.26	0.59
Sex	-0.129	0.004	0.04	0.29	0.058	0.004	0.34	0.63	-0.087	0.004	0.23	0.41	0.103	0.005	0.09	0.82
NICU stay	0.025	0.009	0.74	0.96	-0.049	0.009	0.49	0.90	0.096	0.010	0.24	0.59	-0.068	0.012	0.33	0.90
GA at delivery	-0.014	0.002	0.85	0.85	0.030	0.001	0.69	0.89	-0.017	0.002	0.85	0.95	0.016	0.002	0.83	0.83
GA at T1	-0.047	0.001	0.46	0.71	0.073	0.001	0.23	0.93	0.040	0.001	0.58	0.71	0.069	0.001	0.25	0.80
PMA at scan	0.458	0.002	<0.001	**<0.001**	-0.527	0.001	<0.001	**<0.001**	-0.087	0.002	0.28	0.28	-0.529	0.002	<0.001	**<0.001**
**Superior Cingulum Bundle**																
IL-6 T1	0.022	0.002	0.74	0.84	-0.004	0.003	0.95	0.96	0.041	0.003	0.55	0.74	-0.024	0.003	0.70	0.85
Sex	-0.103	0.003	0.13	0.39	-0.037	0.005	0.56	0.84	-0.126	0.006	0.06	0.14	0.005	0.006	0.94	0.98
NICU stay	0.028	0.008	0.72	0.96	0.061	0.012	0.41	0.90	0.077	0.014	0.33	0.59	0.039	0.014	0.60	0.90
GA at delivery	0.129	0.001	0.12	0.37	-0.009	0.002	0.90	0.94	0.082	0.002	0.33	0.93	-0.057	0.002	0.47	0.72
GA at T1	-0.030	0.001	0.66	0.84	-0.014	0.001	0.82	0.93	-0.048	0.001	0.48	0.71	-0.001	0.001	0.98	0.98
PMA at scan	0.282	0.001	<0.001	**<0.001**	-0.473	0.002	<0.001	**<0.001**	-0.377	0.002	<0.001	**<0.001**	-0.448	0.002	<0.001	**<0.001**
**Corticospinal Tract**																
IL-6 T1	-0.039	0.002	0.53	0.79	-0.023	0.002	0.67	0.96	-0.066	0.002	0.26	0.65	-0.007	0.003	0.90	0.90
Sex	-0.071	0.003	0.25	0.41	0.009	0.005	0.88	0.88	-0.043	0.004	0.47	0.54	0.027	0.006	0.64	0.98
NICU stay	0.113	0.008	0.12	0.96	0.010	0.011	0.87	0.98	0.106	0.010	0.13	0.59	-0.023	0.013	0.72	0.90
GA at delivery	-0.053	0.001	0.49	0.55	0.061	0.002	0.37	0.84	0.044	0.002	0.55	0.93	0.061	0.002	0.38	0.72
GA at T1	0.019	0.001	0.76	0.86	0.023	0.001	0.67	0.93	0.063	0.001	0.29	0.71	0.008	0.001	0.89	0.98
PMA at scan	0.524	0.001	<0.001	**<0.001**	-0.666	0.002	<0.001	**<0.001**	-0.550	0.002	<0.001	**<0.001**	-0.653	0.002	<0.001	**<0.001**
**Optic Radiation**																
IL-6 T1	0.002	0.002	0.98	0.98	-0.089	0.003	0.14	0.62	-0.093	0.003	0.17	0.65	-0.071	0.003	0.22	0.59
Sex	-0.080	0.003	0.18	0.41	-0.066	0.006	0.27	0.63	-0.166	0.006	0.02	0.05	-0.028	0.007	0.63	0.98
NICU stay	-0.078	0.008	0.27	0.96	0.056	0.014	0.42	0.90	0.032	0.014	0.68	0.77	0.064	0.015	0.35	0.90
GA at delivery	-0.100	0.001	0.18	0.38	0.006	0.002	0.94	0.94	-0.054	0.002	0.52	0.93	0.029	0.003	0.69	0.77

GA at T1	-0.010	0.001	0.87	0.87	0.015	0.001	0.81	0.93	0.038	0.001	0.58	0.71	0.018	0.002	0.76	0.98
PMA at scan	0.559	0.001	<0.001	**<0.001**	-0.545	0.002	<0.001	**<0.001**	-0.271	0.002	<0.001	**<0.001**	-0.592	0.003	<0.001	**<0.001**
**Uncinate Fasciculus**																
IL-6 T1	-0.090	0.001	0.14	0.79	-0.005	0.002	0.94	0.96	-0.073	0.003	0.29	0.65	0.025	0.003	0.67	0.85
Sex	-0.114	0.003	0.06	0.29	-0.074	0.005	0.23	0.63	-0.166	0.006	0.02	0.05	-0.025	0.005	0.67	0.98
NICU stay	0.009	0.007	0.90	0.96	0.002	0.011	0.98	0.98	-0.010	0.014	0.90	0.90	0.008	0.012	0.90	0.90
GA at delivery	0.094	0.001	0.21	0.38	0.108	0.002	0.16	0.71	0.186	0.002	0.03	0.27	0.052	0.002	0.48	0.72
GA at T1	-0.110	0.001	0.07	0.66	0.026	0.001	0.67	0.93	-0.041	0.001	0.55	0.71	0.045	0.001	0.44	0.80
PMA at scan	0.448	0.001	<0.001	**<0.001**	-0.556	0.002	<0.001	**<0.001**	-0.301	0.002	<0.001	**<0.001**	-0.596	0.002	<0.001	**<0.001**
**Inferior Fronto-Occipital Fasciculus**																
IL-6 T1	0.042	0.002	0.45	0.79	-0.044	0.003	0.42	0.76	-0.022	0.002	0.74	0.74	-0.044	0.003	0.41	0.74
Sex	-0.040	0.004	0.47	0.53	-0.088	0.005	0.11	0.63	-0.178	0.004	0.01	0.05	-0.049	0.007	0.36	0.98
NICU stay	-0.003	0.008	0.96	0.96	0.043	0.012	0.50	0.90	0.077	0.010	0.31	0.59	0.030	0.015	0.63	0.90
GA at delivery	0.054	0.001	0.43	0.55	-0.027	0.002	0.69	0.89	0.030	0.002	0.71	0.93	-0.038	0.003	0.57	0.73
GA at T1	-0.058	0.001	0.30	0.71	0.028	0.001	0.62	0.93	-0.013	0.001	0.84	0.84	0.046	0.002	0.39	0.80
PMA at scan	0.603	0.001	<0.001	**<0.001**	-0.634	0.002	<0.001	**<0.001**	-0.401	0.002	<0.001	**<0.001**	-0.644	0.003	<0.001	**<0.001**
**Anterior Limb of Internal Capsule**																
IL-6 T1	0.042	0.001	0.44	0.79	-0.062	0.003	0.29	0.96	-0.049	0.003	0.46	0.74	-0.063	0.004	0.26	0.59
Sex	-0.050	0.003	0.36	0.47	-0.011	0.007	0.85	0.88	-0.041	0.006	0.54	0.54	0.001	0.007	0.98	0.98
NICU stay	0.021	0.007	0.74	0.96	0.060	0.015	0.38	0.90	0.096	0.013	0.22	0.59	0.045	0.017	0.49	0.90
GA at delivery	0.116	0.001	0.09	0.37	-0.029	0.003	0.69	0.89	0.040	0.002	0.63	0.93	-0.050	0.003	0.47	0.72
GA at T1	-0.039	0.001	0.48	0.71	0.044	0.002	0.46	0.93	0.032	0.001	0.63	0.71	0.044	0.002	0.43	0.80
PMA at scan	0.584	0.001	<0.001	**<0.001**	-0.554	0.003	<0.001	**<0.001**	-0.376	0.002	<0.001	**<0.001**	-0.586	0.003	<0.001	**<0.001**
**Inferior Cingulum Bundle**																
IL-6 T1	-0.048	0.001	0.46	0.79	0.003	0.002	0.96	0.96	-0.023	0.002	0.74	0.74	0.019	0.002	0.75	0.85
Sex	0.003	0.002	0.96	0.96	-0.057	0.003	0.35	0.63	-0.050	0.004	0.47	0.54	-0.049	0.003	0.42	0.98
NICU stay	0.067	0.005	0.38	0.96	0.006	0.007	0.93	0.98	0.045	0.010	0.57	0.77	-0.016	0.008	0.82	0.90
GA at delivery	-0.171	0.001	0.04	0.34	0.118	0.001	0.12	0.71	-0.004	0.002	0.96	0.96	0.161	0.001	0.03	0.27
GA at T1	-0.071	0.001	0.28	0.71	0.005	0.001	0.94	0.94	-0.034	0.001	0.62	0.71	0.028	0.001	0.64	0.96
PMA at scan	0.411	0.001	<0.001	**<0.001**	-0.569	0.001	<0.001	**<0.001**	-0.298	0.002	<0.001	**<0.001**	-0.609	0.001	<0.001	**<0.001**
**Fornix**																
IL-6 T1	0.031	0.001	0.65	0.84	-0.088	0.002	0.13	0.62	-0.069	0.002	0.28	0.65	-0.089	0.002	0.12	0.59
Sex0	-0.075	0.002	0.27	0.41	-0.020	0.003	0.72	0.88	-0.045	0.005	0.48	0.54	0.002	0.003	0.97	0.98
NICU stay	0.019	0.005	0.81	0.96	0.028	0.008	0.67	0.98	0.034	0.010	0.64	0.77	0.022	0.008	0.73	0.90
GA at delivery	-0.076	0.001	0.36	0.54	0.066	0.001	0.35	0.84	0.028	0.002	0.73	0.93	0.085	0.001	0.23	0.72
GA at T1	-0.060	0.000	0.37	0.71	0.081	0.001	0.16	0.93	0.078	0.001	0.22	0.71	0.081	0.001	0.15	0.80
PMA at scan	0.346	0.001	<0.001	**<0.001**	-0.606	0.001	<0.001	**<0.001**	-0.462	0.002	<0.001	**<0.001**	-0.623	0.001	<0.001	**<0.001**

IL-6 Trimester 2

	FA				MD				AD				RD			

	β	SE	*p*	*q*	β	SE	*p*	*q*	β	SE	*p*	*q*	β	SE	*p*	*q*
**Corpus Callosum**																
IL-6 T2	-0.114	0.002	0.06	0.13	0.052	0.002	0.37	0.51	-0.101	0.002	0.15	0.24	0.084	0.002	0.14	0.42
Sex	-0.065	0.004	0.28	0.97	-0.035	0.004	0.54	0.61	-0.099	0.004	0.16	0.25	0.017	0.005	0.77	0.98
NICU stay	-0.059	0.009	0.42	0.82	-0.044	0.009	0.55	0.79	0.038	0.010	0.67	0.92	-0.026	0.013	0.72	0.82
GA at delivery	0.070	0.001	0.37	0.67	-0.059	0.001	0.46	0.85	0.153	0.001	0.11	0.43	-0.100	0.002	0.20	0.63
GA at T2	0.006	0.001	0.92	0.99	0.019	0.001	0.75	0.75	0.037	0.001	0.60	0.68	0.022	0.001	0.70	0.89
PMA at scan	0.463	0.001	<0.001	**<0.001**	-0.564	0.001	<0.001	**<0.001**	-0.208	0.002	0.01	**0.01**	-0.549	0.002	<0.001	**<0.001**
**Superior Cingulum Bundle**																
IL-6 T2	0.104	0.002	0.11	0.17	-0.104	0.003	0.09	0.41	-0.024	0.003	0.72	0.81	-0.124	0.003	0.04	0.20
Sex	-0.049	0.003	0.46	0.97	-0.031	0.005	0.61	0.61	-0.091	0.006	0.17	0.25	-0.002	0.006	0.98	0.98
NICU stay	-0.003	0.007	0.97	0.97	0.071	0.012	0.36	0.64	0.062	0.014	0.46	0.92	0.061	0.014	0.43	0.78
GA at delivery	0.022	0.001	0.81	0.81	0.117	0.002	0.16	0.85	0.131	0.002	0.14	0.43	0.090	0.002	0.28	0.63
GA at T2	-0.047	0.000	0.49	0.99	0.034	0.001	0.58	0.69	0.027	0.001	0.69	0.69	0.032	0.001	0.61	0.89
PMA at scan	0.380	0.001	<0.001	**<0.001**	-0.527	0.002	<0.001	**<0.001**	-0.393	0.002	<0.001	**<0.001**	-0.515	0.002	<0.001	**<0.001**
**Corticospinal Tract**																
IL-6 T2	-0.193	0.002	<0.01	**0.01**	0.110	0.002	0.03	0.27	-0.012	0.002	0.83	0.83	0.133	0.003	0.01	0.10
Sex	0.048	0.003	0.39	0.97	-0.062	0.005	0.22	0.49	-0.057	0.004	0.30	0.34	-0.058	0.006	0.26	0.60
NICU stay	-0.042	0.008	0.55	0.82	0.025	0.012	0.69	0.79	0.051	0.010	0.47	0.92	0.022	0.014	0.73	0.82
GA at delivery	0.040	0.001	0.59	0.74	-0.025	0.002	0.72	0.85	0.034	0.001	0.65	0.65	-0.015	0.002	0.82	0.93
GA at T2	0.018	0.000	0.76	0.99	0.027	0.001	0.61	0.69	0.049	0.001	0.39	0.58	0.011	0.001	0.84	0.89
PMA at scan	0.535	0.001	<0.001	**<0.001**	-0.677	0.002	<0.001	**<0.001**	-0.632	0.001	<0.001	**<0.001**	-0.659	0.002	<0.001	**<0.001**
**Optic Radiation**																
IL-6 T2	-0.152	0.002	0.01	**0.03**	0.026	0.003	0.62	0.70	-0.091	0.003	0.16	0.24	0.063	0.003	0.21	0.45
Sex	0.000	0.004	1.00	1.00	-0.147	0.006	0.01	**0.03**	-0.214	0.006	<0.01	**0.01**	-0.112	0.006	0.03	0.12
NICU stay	-0.097	0.008	0.16	0.73	0.102	0.013	0.12	0.64	0.062	0.014	0.45	0.92	0.108	0.014	0.09	0.50
GA at delivery	0.033	0.001	0.65	0.74	-0.075	0.002	0.29	0.85	-0.044	0.002	0.62	0.65	-0.073	0.002	0.28	0.63
GA at T2	0.040	0.001	0.48	0.99	0.051	0.001	0.34	0.69	0.081	0.001	0.23	0.41	0.032	0.001	0.53	0.89
PMA at scan	0.554	0.001	<0.001	**<0.001**	-0.601	0.002	<0.001	**<0.001**	-0.323	0.002	<0.001	**<0.001**	-0.633	0.002	<0.001	**<0.001**
**Uncinate Fasciculus**																
IL-6 T2	-0.124	0.001	0.03	0.09	-0.063	0.002	0.30	0.51	-0.157	0.003	0.02	0.19	-0.018	0.003	0.75	0.75
Sex	-0.035	0.003	0.54	0.97	-0.058	0.005	0.33	0.60	-0.098	0.006	0.15	0.25	-0.037	0.005	0.51	0.76
NICU stay	-0.025	0.007	0.73	0.89	0.029	0.011	0.70	0.79	0.008	0.013	0.92	0.96	0.036	0.012	0.62	0.82
GA at delivery	0.104	0.001	0.17	0.52	0.055	0.002	0.50	0.85	0.147	0.002	0.11	0.43	0.003	0.002	0.97	0.97
GA at T2	0.002	0.000	0.97	0.99	0.074	0.001	0.23	0.69	0.089	0.001	0.20	0.41	0.053	0.001	0.36	0.89
PMA at scan	0.524	0.001	<0.001	**<0.001**	-0.549	0.002	<0.001	**<0.001**	-0.246	0.002	<0.001	**<0.001**	-0.605	0.002	<0.001	**<0.001**
**Inferior Fronto-Occipital Fasciculus**																
IL-6 T2	-0.083	0.002	0.10	0.17	-0.014	0.003	0.80	0.80	-0.112	0.002	0.08	0.24	0.016	0.003	0.75	0.75
Sex	0.012	0.004	0.82	1.00	-0.109	0.006	0.04	0.12	-0.181	0.004	<0.01	**0.01**	-0.078	0.007	0.13	0.39

NICU stay	-0.046	0.008	0.47	0.82	0.070	0.013	0.29	0.64	0.061	0.010	0.44	0.92	0.067	0.016	0.30	0.67
GA at delivery	0.082	0.001	0.23	0.52	-0.014	0.002	0.85	0.85	0.092	0.002	0.29	0.57	-0.037	0.002	0.59	0.88
GA at T2	0.037	0.000	0.47	0.99	0.027	0.001	0.61	0.69	0.106	0.001	0.11	0.41	0.007	0.001	0.89	0.89
PMA at scan	0.635	0.001	<0.001	**<0.001**	-0.651	0.002	<0.001	**<0.001**	-0.427	0.002	<0.001	**<0.001**	-0.661	0.002	<0.001	**<0.001**
**Anterior Limb of Internal Capsule**																
IL-6 T2	-0.042	0.001	0.40	0.45	-0.047	0.003	0.40	0.51	-0.096	0.003	0.13	0.24	-0.030	0.004	0.58	0.75
Sex	0.003	0.003	0.95	1.00	-0.045	0.007	0.42	0.60	-0.059	0.006	0.36	0.36	-0.037	0.008	0.49	0.76
NICU stay	-0.062	0.007	0.32	0.82	0.068	0.016	0.33	0.64	0.050	0.013	0.53	0.92	0.071	0.017	0.29	0.67
GA at delivery	0.142	0.001	0.04	0.30	-0.021	0.002	0.78	0.85	0.076	0.002	0.38	0.57	-0.051	0.003	0.48	0.87
GA at T2	-0.015	0.000	0.76	0.99	0.066	0.001	0.25	0.69	0.099	0.001	0.13	0.41	0.053	0.001	0.34	0.89
PMA at scan	0.616	0.001	<0.001	**<0.001**	-0.596	0.002	<0.001	**<0.001**	-0.442	0.002	<0.001	**<0.001**	-0.621	0.003	<0.001	**<0.001**
**Inferior Cingulum Bundle**																
IL-6 T2	-0.062	0.001	0.33	0.43	-0.055	0.001	0.32	0.51	-0.097	0.002	0.14	0.24	-0.026	0.002	0.63	0.75
Sex	-0.021	0.003	0.75	1.00	-0.205	0.003	<0.001	**<0.01**	-0.211	0.004	<0.01	**0.01**	-0.172	0.003	<0.01	**0.02**
NICU stay	-0.114	0.006	0.16	0.73	0.068	0.007	0.33	0.64	-0.030	0.009	0.72	0.92	0.110	0.008	0.11	0.50
GA at delivery	-0.159	0.001	0.07	0.30	0.093	0.001	0.22	0.85	-0.045	0.001	0.62	0.65	0.148	0.001	0.05	0.41
GA at T2	0.001	0.000	0.99	0.99	0.076	0.000	0.18	0.69	0.081	0.001	0.23	0.41	0.073	0.000	0.20	0.89
PMA at scan	0.444	0.001	<0.001	**<0.001**	-0.622	0.001	<0.001	**<0.001**	-0.312	0.001	<0.001	**<0.001**	-0.658	0.001	<0.001	**<0.001**
**Fornix**																
IL-6 T2	0.029	0.001	0.65	0.65	-0.062	0.002	0.26	0.51	-0.045	0.002	0.47	0.61	-0.062	0.002	0.25	0.45
Sex	-0.090	0.002	0.17	0.97	-0.040	0.003	0.47	0.60	-0.078	0.004	0.21	0.27	-0.009	0.003	0.88	0.98
NICU stay	0.022	0.005	0.79	0.89	-0.012	0.008	0.86	0.86	-0.004	0.010	0.96	0.96	-0.014	0.008	0.83	0.83
GA at delivery	0.041	0.001	0.64	0.74	0.043	0.001	0.56	0.85	0.076	0.002	0.36	0.57	0.022	0.001	0.76	0.93
GA at T2	-0.007	0.000	0.91	0.99	-0.035	0.000	0.54	0.69	-0.045	0.001	0.48	0.62	-0.024	0.000	0.67	0.89
PMA at scan	0.376	0.001	<0.001	**<0.001**	-0.649	0.001	<0.001	**<0.001**	-0.500	0.002	<0.001	**<0.001**	-0.655	0.001	<0.001	**<0.001**

IL-6 Trimester 3

	FA				MD				AD				RD			
	**β**	**SE**	***p***	***q***	**β**	**SE**	***p***	***q***	**β**	**SE**	***p***	***q***	**β**	**SE**	***p***	***q***
**Corpus Callosum**																
IL-6 T3	-0.032	0.002	0.57	0.82	-0.068	0.002	0.20	0.25	-0.131	0.002	0.04	0.05	-0.026	0.003	0.62	0.73
Sex	-0.136	0.003	0.01	0.12	0.014	0.003	0.78	0.95	-0.085	0.004	0.17	0.30	0.076	0.005	0.14	0.41
NICU stay	-0.045	0.009	0.47	0.60	-0.026	0.009	0.67	0.91	0.007	0.009	0.92	0.92	-0.006	0.012	0.92	0.92
GA at delivery	-0.006	0.001	0.93	0.93	-0.001	0.001	0.99	0.99	0.065	0.002	0.42	0.72	-0.018	0.002	0.79	0.89
GA at T3	0.045	0.001	0.42	0.59	-0.024	0.001	0.65	0.86	0.025	0.001	0.70	0.93	-0.045	0.001	0.41	0.74
PMA at scan	0.460	0.001	<0.001	**<0.001**	-0.583	0.001	<0.001	**<0.001**	-0.210	0.001	<0.001	**<0.001**	-0.568	0.002	<0.001	**<0.001**
**Superior Cingulum Bundle**																
IL-6 T3	0.054	0.002	0.37	0.67	-0.084	0.003	0.13	0.20	-0.064	0.003	0.28	0.28	-0.079	0.003	0.16	0.26
Sex	-0.084	0.003	0.15	0.45	-0.018	0.005	0.73	0.95	-0.097	0.005	0.09	0.25	0.018	0.005	0.74	0.94
NICU stay	-0.063	0.008	0.36	0.60	0.009	0.012	0.89	0.91	-0.045	0.014	0.50	0.71	0.030	0.014	0.64	0.92

GA at delivery	0.043	0.001	0.58	0.87	0.057	0.002	0.43	0.99	0.079	0.002	0.30	0.72	0.034	0.002	0.63	0.82
GA at T3	-0.089	0.001	0.15	0.53	0.039	0.001	0.50	0.86	-0.024	0.001	0.69	0.93	0.060	0.001	0.30	0.74
PMA at scan	0.327	0.001	<0.001	**<0.001**	-0.535	0.002	<0.001	**<0.001**	-0.417	0.002	<0.001	**<0.001**	-0.509	0.002	<0.001	**<0.001**
**Corticospinal Tract**																
IL-6 T3	-0.166	0.002	<0.01	**0.01**	0.010	0.002	0.82	0.82	-0.153	0.002	<0.01	**0.01**	0.064	0.003	0.18	0.26
Sex	-0.002	0.003	0.97	0.97	-0.040	0.004	0.38	0.83	-0.063	0.004	0.21	0.32	-0.026	0.005	0.57	0.86
NICU stay	-0.071	0.008	0.22	0.60	0.028	0.011	0.59	0.91	-0.035	0.009	0.56	0.71	0.037	0.014	0.48	0.92
GA at delivery	-0.039	0.001	0.56	0.87	-0.002	0.002	0.97	0.99	-0.036	0.002	0.59	0.72	0.034	0.002	0.56	0.82
GA at T3	0.090	0.001	0.09	0.53	-0.032	0.001	0.50	0.86	0.042	0.001	0.43	0.93	-0.053	0.001	0.27	0.74
PMA at scan	0.536	0.001	<0.001	**<0.001**	-0.682	0.002	<0.001	**<0.001**	-0.594	0.001	<0.001	**<0.001**	-0.666	0.002	<0.001	**<0.001**
**Optic Radiation**																
IL-6 T3	-0.072	0.002	0.17	0.39	-0.038	0.003	0.46	0.52	-0.102	0.003	0.09	0.10	-0.006	0.003	0.90	0.90
Sex	0.003	0.003	0.95	0.97	-0.110	0.005	0.03	0.14	-0.167	0.005	<0.01	**0.04**	-0.082	0.006	0.09	0.41
NICU stay	-0.087	0.009	0.15	0.60	0.074	0.013	0.20	0.90	0.025	0.013	0.72	0.81	0.084	0.015	0.13	0.60
GA at delivery	-0.024	0.001	0.72	0.89	-0.060	0.002	0.36	0.99	-0.091	0.002	0.24	0.72	-0.037	0.002	0.56	0.82
GA at T3	0.028	0.001	0.61	0.61	0.038	0.001	0.47	0.86	0.087	0.001	0.16	0.93	0.017	0.001	0.73	0.77
PMA at scan	0.539	0.001	<0.001	**<0.001**	-0.572	0.002	<0.001	**<0.001**	-0.288	0.002	<0.001	**<0.001**	-0.609	0.002	<0.001	**<0.001**
**Uncinate Fasciculus**																
IL-6 T3	-0.104	0.002	0.05	0.15	-0.169	0.002	<0.01	**0.02**	-0.258	0.003	<0.001	**<0.001**	-0.105	0.003	0.04	0.17
Sex	-0.065	0.003	0.21	0.47	-0.039	0.004	0.46	0.83	-0.091	0.005	0.11	0.25	-0.011	0.005	0.83	0.94
NICU stay	-0.047	0.007	0.44	0.60	-0.136	0.011	0.03	0.25	-0.183	0.013	0.01	0.06	-0.090	0.012	0.13	0.60
GA at delivery	0.122	0.001	0.07	0.37	0.010	0.002	0.88	0.99	0.094	0.002	0.21	0.72	-0.036	0.002	0.59	0.82
GA at T3	-0.035	0.000	0.53	0.59	0.010	0.001	0.85	0.96	-0.005	0.001	0.93	0.93	0.017	0.001	0.75	0.77
PMA at scan	0.456	0.001	<0.001	**<0.001**	-0.544	0.002	<0.001	**<0.001**	-0.285	0.002	<0.001	**<0.001**	-0.588	0.002	<0.001	**<0.001**
**Inferior Fronto-Occipital Fasciculus**																
IL-6 T3	-0.009	0.002	0.85	0.85	-0.101	0.003	0.04	0.08	-0.167	0.002	<0.01	**0.01**	-0.071	0.003	0.13	0.26
Sex	-0.027	0.003	0.57	0.73	-0.071	0.005	0.13	0.40	-0.140	0.004	0.01	0.06	-0.039	0.006	0.39	0.86
NICU stay	-0.047	0.008	0.40	0.60	-0.011	0.012	0.84	0.91	-0.051	0.010	0.43	0.71	0.008	0.015	0.88	0.92
GA at delivery	0.079	0.001	0.20	0.59	-0.045	0.002	0.47	0.99	0.032	0.002	0.66	0.72	-0.057	0.002	0.35	0.82
GA at T3	0.032	0.001	0.52	0.59	-0.002	0.001	0.97	0.97	0.041	0.001	0.50	0.93	-0.014	0.001	0.77	0.77
PMA at scan	0.600	0.001	<0.001	**<0.001**	-0.646	0.002	<0.001	**<0.001**	-0.428	0.002	<0.001	**<0.001**	-0.654	0.002	<0.001	**<0.001**
**Anterior Limb of Internal Capsule**																
IL-6 T3	0.018	0.002	0.71	0.82	-0.111	0.003	0.03	0.08	-0.152	0.003	0.01	**0.01**	-0.094	0.004	0.06	0.17
Sex	-0.050	0.003	0.28	0.50	-0.010	0.006	0.84	0.95	-0.033	0.005	0.56	0.56	0.003	0.007	0.95	0.95
NICU stay	-0.027	0.007	0.61	0.61	-0.036	0.015	0.53	0.91	-0.081	0.013	0.21	0.71	-0.019	0.017	0.74	0.92
GA at delivery	0.107	0.001	0.08	0.37	-0.034	0.002	0.60	0.99	0.044	0.002	0.55	0.72	-0.054	0.003	0.39	0.82
GA at T3	0.052	0.000	0.29	0.53	-0.022	0.001	0.67	0.86	0.007	0.001	0.91	0.93	-0.030	0.001	0.55	0.77
PMA at scan	0.599	0.001	<0.001	**<0.001**	-0.613	0.002	<0.001	**<0.001**	-0.478	0.002	<0.001	**<0.001**	-0.631	0.003	<0.001	**<0.001**

**Inferior Cingulum Bundle**																
IL-6 T3	-0.146	0.001	0.01	0.06	-0.087	0.002	0.09	0.17	-0.188	0.002	<0.01	**0.01**	-0.023	0.002	0.65	0.73
Sex	0.051	0.002	0.37	0.56	-0.110	0.003	0.03	0.14	-0.057	0.004	0.32	0.42	-0.115	0.003	0.02	0.21
NICU stay	-0.128	0.006	0.06	0.51	0.007	0.007	0.91	0.91	-0.080	0.009	0.24	0.71	0.068	0.008	0.25	0.74
GA at delivery	-0.085	0.001	0.26	0.59	0.111	0.001	0.10	0.87	0.042	0.001	0.59	0.72	0.132	0.001	0.05	0.42
GA at T3	-0.065	0.000	0.28	0.53	0.024	0.001	0.66	0.86	-0.021	0.001	0.73	0.93	0.046	0.001	0.39	0.74
PMA at scan	0.371	0.001	<0.001	**<0.001**	-0.624	0.001	<0.001	**<0.001**	-0.361	0.001	<0.001	**<0.001**	-0.630	0.001	<0.001	**<0.001**
**Fornix**																
IL-6 T3	0.021	0.001	0.73	0.82	-0.135	0.002	0.01	**0.03**	-0.128	0.002	0.02	**0.04**	-0.119	0.002	0.02	0.16
Sex	-0.096	0.002	0.10	0.44	-0.002	0.003	0.97	0.97	-0.049	0.004	0.37	0.42	0.029	0.003	0.55	0.86
NICU stay	-0.041	0.005	0.54	0.61	-0.025	0.008	0.66	0.91	-0.052	0.010	0.42	0.71	-0.007	0.008	0.91	0.92
GA at delivery	0.021	0.001	0.79	0.89	0.007	0.001	0.91	0.99	0.026	0.002	0.72	0.72	0.000	0.001	1.00	1.00
GA at T3	-0.082	0.000	0.18	0.53	0.041	0.001	0.43	0.86	-0.011	0.001	0.84	0.93	0.063	0.001	0.22	0.74
PMA at scan	0.351	0.001	<0.001	**<0.001**	-0.635	0.001	<0.001	**<0.001**	-0.487	0.002	<0.001	**<0.001**	-0.642	0.001	<0.001	**<0.001**

IL-8 Trimester 1

	FA				MD				AD				RD			
	**β**	**SE**	***p***	***q***	**β**	**SE**	***p***	***q***	**β**	**SE**	***p***	***q***	**β**	**SE**	***p***	***q***
**Corpus Callosum**																
IL-8 T1	-0.019	0.002	0.76	0.91	0.076	0.002	0.22	0.67	0.024	0.003	0.74	0.97	0.068	0.003	0.27	0.77
Sex	-0.133	0.004	0.04	0.28	0.047	0.004	0.45	0.67	-0.104	0.004	0.15	0.27	0.097	0.005	0.11	0.99
NICU stay	0.034	0.009	0.64	0.85	-0.065	0.009	0.36	0.84	0.096	0.010	0.24	0.59	-0.085	0.012	0.23	0.88
GA at delivery	-0.008	0.002	0.92	0.92	0.023	0.002	0.77	0.87	-0.005	0.002	0.95	0.95	0.007	0.002	0.93	0.93
GA at T1	-0.046	0.001	0.47	0.70	0.079	0.001	0.20	0.82	0.047	0.001	0.51	0.68	0.074	0.001	0.23	0.59
PMA at scan	0.454	0.002	<0.001	**<0.001**	-0.511	0.002	<0.001	**<0.001**	-0.085	0.002	0.29	0.29	-0.516	0.002	<0.001	**<0.001**
**Superior Cingulum Bundle**																
IL-8 T1	-0.047	0.002	0.49	0.91	0.033	0.003	0.60	0.78	0.002	0.004	0.98	0.98	0.044	0.004	0.50	0.77
Sex	-0.113	0.003	0.10	0.28	-0.049	0.005	0.44	0.67	-0.147	0.006	0.03	0.07	-0.001	0.006	0.99	0.99
NICU stay	0.030	0.008	0.70	0.85	0.056	0.012	0.45	0.84	0.069	0.014	0.38	0.59	0.036	0.014	0.63	0.88
GA at delivery	0.132	0.001	0.12	0.35	-0.008	0.002	0.92	0.92	0.084	0.002	0.32	0.91	-0.056	0.002	0.48	0.65
GA at T1	-0.033	0.001	0.63	0.80	-0.006	0.001	0.92	0.92	-0.042	0.001	0.54	0.68	0.006	0.001	0.92	0.92
PMA at scan	0.275	0.001	<0.001	**<0.001**	-0.471	0.002	<0.001	**<0.001**	-0.382	0.002	<0.001	**<0.001**	-0.445	0.002	<0.001	**<0.001**
**Corticospinal Tract**																
IL-8 T1	-0.034	0.002	0.59	0.48	0.038	0.003	0.50	0.78	0.019	0.003	0.75	0.97	0.041	0.004	0.47	0.77
Sex	-0.067	0.003	0.28	0.44	-0.005	0.005	0.93	0.93	-0.057	0.004	0.34	0.45	0.015	0.006	0.79	0.99
NICU stay	0.126	0.008	0.08	0.74	0.004	0.011	0.95	0.97	0.109	0.010	0.12	0.59	-0.032	0.014	0.63	0.88
GA at delivery	-0.040	0.001	0.61	0.50	0.059	0.002	0.39	0.87	0.057	0.002	0.45	0.91	0.055	0.002	0.44	0.65
GA at T1	0.006	0.001	0.92	0.57	0.039	0.001	0.48	0.82	0.074	0.001	0.22	0.68	0.024	0.001	0.67	0.76
PMA at scan	0.522	0.001	<0.001	**<0.001**	-0.660	0.002	<0.001	**<0.001**	-0.548	0.002	<0.001	**<0.001**	-0.647	0.002	<0.001	**<0.001**
**Optic Radiation**																

IL-8 T1	-0.007	0.002	0.91	0.91	0.023	0.004	0.71	0.80	0.021	0.004	0.76	0.97	0.020	0.004	0.74	0.83
Sex	-0.074	0.003	0.22	0.26	-0.066	0.006	0.28	0.67	-0.164	0.006	0.02	0.05	-0.029	0.007	0.62	0.99
NICU stay	-0.071	0.008	0.31	0.85	0.068	0.014	0.34	0.84	0.051	0.014	0.52	0.59	0.071	0.016	0.30	0.88
GA at delivery	-0.094	0.001	0.21	0.38	0.022	0.002	0.77	0.87	-0.029	0.002	0.74	0.91	0.040	0.003	0.58	0.65
GA at T1	-0.016	0.001	0.80	0.90	0.028	0.001	0.64	0.82	0.053	0.001	0.44	0.68	0.030	0.002	0.61	0.76
PMA at scan	0.562	0.001	<0.001	**<0.001**	-0.543	0.002	<0.001	**<0.001**	-0.267	0.002	<0.001	**<0.001**	-0.591	0.003	<0.001	**<0.001**
**Uncinate Fasciculus**																
IL-8 T1	-0.020	0.002	0.75	0.91	0.037	0.003	0.55	0.78	0.029	0.004	0.68	0.97	0.039	0.003	0.51	0.77
Sex	-0.114	0.003	0.06	0.28	-0.077	0.005	0.21	0.67	-0.167	0.006	0.02	0.05	-0.028	0.005	0.64	0.99
NICU stay	0.022	0.007	0.75	0.85	-0.002	0.011	0.97	0.97	-0.005	0.014	0.95	0.95	0.001	0.012	0.99	0.99
GA at delivery	0.105	0.001	0.17	0.38	0.109	0.002	0.16	0.72	0.192	0.002	0.03	0.24	0.049	0.002	0.50	0.65
GA at T1	-0.124	0.001	0.04	0.39	0.036	0.001	0.56	0.82	-0.038	0.001	0.59	0.68	0.058	0.001	0.33	0.59
PMA at scan	0.447	0.001	<0.001	**<0.001**	-0.556	0.002	<0.001	**<0.001**	-0.305	0.002	<0.001	**<0.001**	-0.595	0.002	<0.001	**<0.001**
**Inferior Fronto-Occipital Fasciculus**																
IL-8 T1	-0.032	0.002	0.57	0.91	0.073	0.003	0.19	0.67	0.091	0.003	0.17	0.76	0.060	0.004	0.27	0.77
Sex	-0.044	0.003	0.42	0.48	-0.096	0.005	0.08	0.67	-0.197	0.005	<0.01	**0.03**	-0.053	0.007	0.33	0.99
NICU stay	-0.001	0.008	0.98	0.98	0.040	0.012	0.54	0.84	0.072	0.010	0.34	0.59	0.028	0.015	0.66	0.88
GA at delivery	0.056	0.001	0.42	0.63	-0.027	0.002	0.69	0.87	0.031	0.002	0.71	0.91	-0.039	0.003	0.57	0.65
GA at T1	-0.074	0.001	0.19	0.65	0.046	0.001	0.41	0.82	0.002	0.001	0.97	0.97	0.063	0.002	0.24	0.59
PMA at scan	0.604	0.001	<0.001	**<0.001**	-0.620	0.002	<0.001	**<0.001**	-0.389	0.002	<0.001	**<0.001**	-0.634	0.003	<0.001	**<0.001**
**Anterior Limb of Internal Capsule**																
IL-8 T1	-0.113	0.002	0.04	0.38	0.091	0.004	0.13	0.67	0.045	0.004	0.50	0.97	0.101	0.004	0.08	0.70
Sex	-0.065	0.003	0.23	0.37	-0.020	0.007	0.74	0.83	-0.063	0.006	0.35	0.45	-0.002	0.007	0.98	0.99
NICU stay	0.026	0.007	0.68	0.85	0.055	0.015	0.42	0.84	0.092	0.014	0.24	0.59	0.040	0.017	0.54	0.88
GA at delivery	0.126	0.001	0.07	0.30	-0.035	0.003	0.63	0.87	0.037	0.002	0.66	0.91	-0.058	0.003	0.41	0.65
GA at T1	-0.041	0.001	0.45	0.70	0.057	0.002	0.33	0.82	0.050	0.001	0.45	0.68	0.055	0.002	0.33	0.59
PMA at scan	0.567	0.001	<0.001	**<0.001**	-0.538	0.003	<0.001	**<0.001**	-0.367	0.002	<0.001	**<0.001**	-0.569	0.003	<0.001	**<0.001**
**Inferior Cingulum Bundle**																
IL-8 T1	0.012	0.001	0.86	0.91	0.006	0.002	0.93	0.93	0.012	0.003	0.86	0.97	0.000	0.002	0.99	0.99
Sex	0.010	0.002	0.89	0.89	-0.057	0.003	0.36	0.67	-0.044	0.004	0.53	0.53	-0.051	0.003	0.39	0.99
NICU stay	0.080	0.006	0.30	0.85	0.010	0.007	0.88	0.97	0.058	0.010	0.48	0.59	-0.018	0.008	0.80	0.90
GA at delivery	-0.160	0.001	0.06	0.30	0.135	0.001	0.08	0.72	0.021	0.002	0.81	0.91	0.171	0.001	0.02	0.21
GA at T1	-0.083	0.001	0.22	0.65	0.010	0.001	0.87	0.92	-0.037	0.001	0.60	0.68	0.037	0.001	0.54	0.76
PMA at scan	0.417	0.001	<0.001	**<0.001**	-0.573	0.001	<0.001	**<0.001**	-0.297	0.002	<0.001	**<0.001**	-0.617	0.001	<0.001	**<0.001**
**Fornix**																
IL-8 T1	-0.055	0.001	0.42	0.91	-0.053	0.002	0.36	0.78	-0.090	0.003	0.17	0.76	-0.025	0.002	0.67	0.83
Sex	-0.077	0.002	0.26	0.37	-0.026	0.003	0.65	0.83	-0.054	0.005	0.40	0.45	-0.001	0.003	0.99	0.99
NICU stay	0.031	0.005	0.69	0.85	0.039	0.008	0.56	0.84	0.052	0.011	0.49	0.59	0.027	0.008	0.69	0.88

GA at delivery	-0.044	0.001	0.61	0.68	0.075	0.001	0.30	0.87	0.054	0.002	0.50	0.91	0.081	0.001	0.26	0.65
GA at T1	-0.070	0.000	0.30	0.68	0.095	0.001	0.10	0.82	0.086	0.001	0.18	0.68	0.096	0.001	0.10	0.59
PMA at scan	0.329	0.001	<0.001	**<0.001**	-0.616	0.001	<0.001	**<0.001**	-0.469	0.002	<0.001	**<0.001**	-0.628	0.001	<0.001	**<0.001**

IL-8 Trimester 2

	FA				MD				AD				RD			
	**β**	**SE**	***p***	***q***	**β**	**SE**	***p***	***q***	**β**	**SE**	***p***	***q***	**β**	**SE**	***p***	***q***
**Corpus Callosum**																
IL-8 T2	-0.091	0.002	0.12	0.96	0.024	0.002	0.68	0.98	0.013	0.002	0.86	0.96	0.052	0.003	0.36	0.96
Sex	-0.080	0.004	0.19	0.81	-0.037	0.004	0.52	0.59	-0.105	0.004	0.14	0.24	0.021	0.005	0.72	0.89
NICU stay	-0.084	0.009	0.25	0.42	-0.025	0.010	0.74	0.90	0.026	0.010	0.77	0.91	0.001	0.013	0.99	0.99
GA at delivery	0.051	0.001	0.51	0.81	-0.044	0.001	0.58	0.90	0.157	0.002	0.10	0.41	-0.083	0.002	0.29	0.81
GA at T2	0.033	0.001	0.59	0.89	0.008	0.001	0.89	0.89	0.054	0.001	0.45	0.58	0.003	0.001	0.96	1.00
PMA at scan	0.482	0.001	<0.001	**<0.001**	-0.570	0.001	<0.001	**<0.001**	-0.198	0.002	0.01	**0.01**	-0.564	0.002	<0.001	**<0.001**
**Superior Cingulum Bundle**																
IL-8 T2	0.044	0.002	0.50	0.96	0.022	0.003	0.72	0.98	0.044	0.003	0.50	0.96	0.007	0.003	0.91	0.96
Sex	-0.073	0.003	0.27	0.81	-0.019	0.005	0.76	0.76	-0.094	0.006	0.16	0.24	0.017	0.006	0.79	0.89
NICU stay	-0.007	0.008	0.93	1.00	0.071	0.013	0.36	0.65	0.061	0.015	0.47	0.91	0.062	0.014	0.42	0.70
GA at delivery	0.021	0.001	0.81	0.81	0.121	0.002	0.15	0.90	0.133	0.002	0.14	0.41	0.094	0.002	0.26	0.81
GA at T2	-0.046	0.000	0.49	0.89	0.046	0.001	0.47	0.84	0.036	0.001	0.59	0.63	0.043	0.001	0.50	1.00
PMA at scan	0.384	0.001	<0.001	**<0.001**	-0.526	0.002	<0.001	**<0.001**	-0.384	0.002	<0.001	**<0.001**	-0.520	0.002	<0.001	**<0.001**
**Corticospinal Tract**																
IL-8 T2	-0.005	0.002	0.93	0.96	0.025	0.003	0.62	0.98	0.026	0.002	0.64	0.96	0.032	0.003	0.55	0.96
Sex	0.045	0.004	0.43	0.90	-0.061	0.005	0.24	0.54	-0.056	0.004	0.32	0.32	-0.055	0.006	0.30	0.67
NICU stay	-0.078	0.008	0.28	0.42	0.051	0.012	0.43	0.65	0.060	0.010	0.39	0.91	0.047	0.015	0.47	0.70
GA at delivery	0.033	0.001	0.67	0.81	-0.016	0.002	0.82	0.90	0.041	0.001	0.59	0.75	-0.002	0.002	0.98	0.98
GA at T2	0.052	0.000	0.38	0.89	0.008	0.001	0.88	0.89	0.052	0.001	0.36	0.55	-0.014	0.001	0.79	1.00
PMA at scan	0.542	0.001	<0.001	**<0.001**	-0.677	0.002	<0.001	**<0.001**	-0.624	0.002	<0.001	**<0.001**	-0.660	0.002	<0.001	**<0.001**
**Optic Radiation**																
IL-8 T2	-0.003	0.002	0.96	0.96	0.065	0.003	0.21	0.97	0.066	0.003	0.31	0.96	0.049	0.003	0.32	0.96
Sex	0.000	0.004	1.00	1.00	-0.137	0.006	0.01	**0.04**	-0.204	0.006	<0.01	**0.01**	-0.104	0.006	0.04	0.18
NICU stay	-0.123	0.009	0.08	0.42	0.127	0.013	0.05	0.48	0.075	0.014	0.36	0.91	0.134	0.015	0.03	0.31
GA at delivery	0.026	0.001	0.73	0.81	-0.052	0.002	0.46	0.90	-0.023	0.002	0.79	0.79	-0.053	0.002	0.43	0.81
GA at T2	0.065	0.001	0.25	0.89	0.043	0.001	0.42	0.84	0.095	0.001	0.15	0.29	0.018	0.001	0.73	1.00
PMA at scan	0.561	0.001	<0.001	**<0.001**	-0.611	0.002	<0.001	**<0.001**	-0.334	0.002	<0.001	**<0.001**	-0.642	0.002	<0.001	**<0.001**
**Uncinate Fasciculus**																
IL-8 T2	0.031	0.001	0.58	0.96	-0.074	0.002	0.22	0.97	-0.063	0.003	0.35	0.96	-0.069	0.003	0.22	0.96
Sex	-0.039	0.003	0.50	0.90	-0.052	0.005	0.40	0.59	-0.092	0.006	0.18	0.24	-0.031	0.005	0.59	0.88
NICU stay	-0.041	0.007	0.57	0.73	0.019	0.012	0.80	0.90	-0.013	0.014	0.88	0.91	0.031	0.012	0.66	0.85

	FA β	FA SE	FA *p*	FA *q*	MD β	MD SE	MD *p*	MD *q*	AD β	AD SE	AD *p*	AD *q*	RD β	RD SE	RD *p*	RD *q*
GA at delivery	0.116	0.001	0.13	0.40	0.041	0.002	0.62	0.90	0.140	0.002	0.13	0.41	-0.012	0.002	0.87	0.98
GA at T2	0.018	0.000	0.75	0.89	0.086	0.001	0.16	0.70	0.111	0.001	0.11	0.29	0.060	0.001	0.30	0.95
PMA at scan	0.521	0.001	<0.001	**<0.001**	-0.526	0.002	<0.001	**<0.001**	-0.222	0.002	<0.001	**<0.001**	-0.588	0.002	<0.001	<0.001
**Inferior Fronto-Occipital Fasciculus**																
IL-8 T2	0.042	0.002	0.41	0.96	-0.023	0.003	0.67	0.98	0.013	0.002	0.84	0.96	-0.029	0.003	0.56	0.96
Sex	0.014	0.004	0.79	0.93	-0.112	0.006	0.04	0.11	-0.185	0.004	<0.01	**0.01**	-0.080	0.007	0.12	0.36
NICU stay	-0.078	0.008	0.22	0.42	0.077	0.013	0.25	0.65	0.035	0.010	0.66	0.91	0.082	0.017	0.20	0.61
GA at delivery	0.077	0.001	0.26	0.59	-0.009	0.002	0.90	0.90	0.092	0.002	0.29	0.59	-0.032	0.002	0.65	0.97
GA at T2	0.043	0.000	0.40	0.89	0.031	0.001	0.57	0.85	0.119	0.001	0.07	0.29	0.007	0.001	0.89	1.00
PMA at scan	0.640	0.001	<0.001	**<0.001**	-0.645	0.002	<0.001	**<0.001**	-0.411	0.002	<0.001	**<0.001**	-0.658	0.003	<0.001	**<0.001**
**Anterior Limb of Internal Capsule**																
IL-8 T2	0.008	0.001	0.86	0.96	-0.002	0.003	0.98	0.98	0.003	0.003	0.97	0.96	-0.003	0.004	0.96	0.96
Sex	-0.011	0.003	0.83	0.93	-0.045	0.007	0.43	0.59	-0.071	0.006	0.28	0.31	-0.034	0.008	0.53	0.88
NICU stay	-0.086	0.007	0.17	0.42	0.056	0.016	0.43	0.65	0.016	0.014	0.84	0.91	0.067	0.018	0.32	0.70
GA at delivery	0.148	0.001	0.03	0.26	-0.024	0.002	0.75	0.90	0.075	0.002	0.39	0.59	-0.055	0.003	0.45	0.81
GA at T2	-0.014	0.000	0.79	0.89	0.069	0.001	0.23	0.70	0.104	0.001	0.12	0.29	0.055	0.001	0.32	0.95
PMA at scan	0.621	0.001	<0.001	**<0.001**	-0.590	0.002	<0.001	**<0.001**	-0.426	0.002	<0.001	**<0.001**	-0.618	0.003	<0.001	**<0.001**
**Inferior Cingulum Bundle**																
IL-8 T2	-0.026	0.001	0.68	0.96	-0.003	0.002	0.96	0.98	-0.027	0.002	0.68	0.96	0.009	0.002	0.87	0.96
Sex	-0.019	0.003	0.77	0.93	-0.198	0.003	<0.01	**0.01**	-0.203	0.004	<0.01	**0.01**	-0.166	0.003	<0.01	**0.03**
NICU stay	-0.131	0.006	0.11	0.42	0.080	0.007	0.26	0.65	-0.030	0.010	0.72	0.91	0.126	0.008	0.07	0.31
GA at delivery	-0.153	0.001	0.08	0.35	0.095	0.001	0.21	0.90	-0.039	0.001	0.67	0.75	0.148	0.001	0.05	0.41
GA at T2	-0.007	0.000	0.92	0.92	0.095	0.000	0.10	0.70	0.095	0.001	0.16	0.29	0.091	0.000	0.10	0.93
PMA at scan	0.448	0.001	<0.001	**<0.001**	-0.611	0.001	<0.001	**<0.001**	-0.299	0.002	<0.001	**<0.001**	-0.653	0.001	<0.001	**<0.001**
**Fornix**																
IL-8 T2	0.007	0.001	0.92	0.96	0.015	0.002	0.79	0.98	0.020	0.002	0.74	0.96	0.010	0.002	0.86	0.96
Sex	-0.105	0.002	0.11	0.81	-0.038	0.003	0.50	0.59	-0.087	0.005	0.17	0.24	0.000	0.003	0.99	0.99
NICU stay	0.000	0.005	1.00	1.00	-0.006	0.008	0.94	0.94	-0.009	0.011	0.91	0.91	-0.002	0.008	0.98	0.99
GA at delivery	0.041	0.001	0.64	0.81	0.045	0.001	0.55	0.90	0.079	0.002	0.35	0.59	0.022	0.001	0.76	0.98
GA at T2	-0.023	0.000	0.73	0.89	-0.013	0.000	0.82	0.89	-0.031	0.001	0.63	0.63	0.000	0.000	1.00	1.00
PMA at scan	0.373	0.001	<0.001	**<0.001**	-0.640	0.001	<0.001	**<0.001**	-0.483	0.002	<0.001	**<0.001**	-0.649	0.001	<0.001	**<0.001**

IL-8 Trimester 3

	FA				MD				AD				RD			
	**β**	**SE**	***p***	***q***	**β**	**SE**	***p***	***q***	**β**	**SE**	***p***	***q***	**β**	**SE**	***p***	***q***
**Corpus Callosum**																
IL-8 T3	-0.033	0.002	0.56	0.63	0.006	0.002	0.91	0.92	-0.032	0.002	0.62	0.92	0.025	0.002	0.63	0.82
Sex	-0.133	0.003	0.02	0.14	-0.004	0.003	0.94	0.94	-0.108	0.004	0.09	0.14	0.064	0.005	0.22	0.59
NICU stay	-0.035	0.009	0.57	0.64	-0.019	0.010	0.76	0.98	0.032	0.010	0.66	0.87	-0.005	0.013	0.93	0.93

GA at delivery	-0.003	0.001	0.97	0.99	0.006	0.001	0.93	0.93	0.080	0.002	0.33	0.58	-0.013	0.002	0.85	0.99
GA at T3	0.054	0.001	0.35	0.50	-0.038	0.001	0.49	0.91	0.014	0.001	0.83	0.94	-0.058	0.001	0.29	0.76
PMA at scan	0.456	0.001	<0.001	**<0.001**	-0.556	0.001	<0.001	**<0.001**	-0.176	0.002	0.01	**0.01**	-0.549	0.002	<0.001	**<0.001**
**Superior Cingulum Bundle**																
IL-8 T3	-0.043	0.002	0.47	0.63	0.034	0.002	0.54	0.86	0.014	0.003	0.82	0.92	0.044	0.003	0.43	0.71
Sex	-0.080	0.003	0.18	0.40	-0.037	0.005	0.50	0.74	-0.116	0.005	0.05	0.10	0.003	0.005	0.96	0.96
NICU stay	-0.060	0.008	0.38	0.57	0.011	0.013	0.87	0.98	-0.043	0.014	0.52	0.87	0.031	0.014	0.63	0.84
GA at delivery	0.001	0.001	0.99	0.99	0.091	0.002	0.21	0.93	0.089	0.002	0.24	0.58	0.075	0.002	0.31	0.99
GA at T3	-0.060	0.001	0.34	0.50	0.007	0.001	0.91	0.91	-0.040	0.001	0.52	0.94	0.026	0.001	0.66	0.85
PMA at scan	0.322	0.001	<0.001	**<0.001**	-0.515	0.002	<0.001	**<0.001**	-0.397	0.002	<0.001	**<0.001**	-0.492	0.002	<0.001	**<0.001**
**Corticospinal Tract**																
IL-8 T3	-0.081	0.002	0.12	0.63	0.071	0.002	0.13	0.63	0.030	0.002	0.58	0.92	0.077	0.003	0.11	0.71
Sex	-0.016	0.003	0.75	0.84	-0.046	0.004	0.31	0.56	-0.089	0.004	0.09	0.14	-0.026	0.005	0.58	0.83
NICU stay	-0.060	0.008	0.32	0.57	0.032	0.012	0.55	0.98	-0.017	0.010	0.78	0.88	0.035	0.014	0.51	0.84
GA at delivery	-0.018	0.001	0.79	0.99	0.010	0.002	0.87	0.93	0.005	0.002	0.95	0.95	0.033	0.002	0.57	0.99
GA at T3	0.100	0.001	0.07	0.50	-0.055	0.001	0.26	0.91	0.015	0.001	0.79	0.94	-0.072	0.001	0.14	0.76
PMA at scan	0.543	0.001	<0.001	**<0.001**	-0.663	0.002	<0.001	**<0.001**	-0.556	0.002	<0.001	**<0.001**	-0.655	0.002	<0.001	**<0.001**
**Optic Radiation**																
IL-8 T3	-0.039	0.002	0.46	0.63	0.075	0.003	0.14	0.63	0.037	0.003	0.54	0.92	0.069	0.003	0.16	0.71
Sex	0.003	0.003	0.96	0.96	-0.110	0.005	0.03	0.13	-0.167	0.005	0.01	**0.02**	-0.082	0.006	0.09	0.40
NICU stay	-0.099	0.009	0.10	0.57	0.059	0.014	0.31	0.98	-0.008	0.014	0.91	0.91	0.077	0.016	0.17	0.84
GA at delivery	-0.029	0.001	0.67	0.99	-0.014	0.002	0.83	0.93	-0.038	0.002	0.62	0.70	-0.001	0.002	0.99	0.99
GA at T3	0.037	0.001	0.51	0.57	0.008	0.001	0.88	0.91	0.054	0.001	0.39	0.94	-0.008	0.001	0.88	0.89
PMA at scan	0.544	0.001	<0.001	**<0.001**	-0.573	0.002	<0.001	**<0.001**	-0.286	0.002	<0.001	**<0.001**	-0.611	0.002	<0.001	**<0.001**
**Uncinate Fasciculus**																
IL-8 T3	0.032	0.001	0.55	0.63	-0.006	0.002	0.92	0.92	0.004	0.003	0.95	0.95	-0.013	0.002	0.81	0.91
Sex	-0.077	0.003	0.14	0.40	-0.064	0.004	0.24	0.53	-0.127	0.005	0.03	0.10	-0.028	0.005	0.59	0.83
NICU stay	-0.047	0.007	0.44	0.57	-0.113	0.012	0.08	0.70	-0.156	0.015	0.03	0.24	-0.072	0.013	0.24	0.84
GA at delivery	0.128	0.001	0.07	0.59	0.048	0.002	0.51	0.93	0.135	0.002	0.09	0.58	-0.007	0.002	0.92	0.99
GA at T3	-0.014	0.000	0.80	0.80	-0.011	0.001	0.85	0.91	-0.012	0.001	0.85	0.94	-0.008	0.001	0.89	0.89
PMA at scan	0.470	0.001	<0.001	**<0.001**	-0.513	0.002	<0.001	**<0.001**	-0.243	0.002	<0.001	**<0.001**	-0.568	0.002	<0.001	**<0.001**
**Inferior Fronto-Occipital Fasciculus**																
IL-8 T3	-0.050	0.002	0.30	0.63	0.037	0.002	0.45	0.86	0.014	0.002	0.81	0.92	0.040	0.003	0.40	0.71
Sex	-0.026	0.003	0.59	0.84	-0.087	0.005	0.07	0.21	-0.163	0.004	<0.01	**0.02**	-0.052	0.006	0.26	0.59
NICU stay	-0.052	0.008	0.35	0.57	-0.012	0.013	0.83	0.98	-0.062	0.011	0.36	0.80	0.010	0.016	0.85	0.93
GA at delivery	0.051	0.001	0.41	0.93	-0.011	0.002	0.86	0.93	0.058	0.002	0.45	0.58	-0.024	0.003	0.70	0.99
GA at T3	0.053	0.001	0.29	0.50	-0.020	0.001	0.70	0.91	0.029	0.001	0.63	0.94	-0.033	0.001	0.51	0.76
PMA at scan	0.603	0.001	<0.001	**<0.001**	-0.631	0.002	<0.001	**<0.001**	-0.398	0.002	<0.001	**<0.001**	-0.644	0.002	<0.001	**<0.001**

**Anterior Limb of Internal Capsule**																
IL-8 T3	-0.051	0.001	0.28	0.63	0.025	0.003	0.62	0.86	-0.028	0.003	0.63	0.92	0.036	0.003	0.47	0.71
Sex	-0.048	0.003	0.30	0.54	-0.028	0.006	0.57	0.74	-0.060	0.005	0.29	0.29	-0.012	0.007	0.80	0.90
NICU stay	-0.020	0.007	0.72	0.72	-0.042	0.016	0.47	0.98	-0.085	0.014	0.20	0.80	-0.025	0.018	0.65	0.84
GA at delivery	0.092	0.001	0.14	0.62	-0.012	0.002	0.86	0.93	0.061	0.002	0.42	0.58	-0.032	0.003	0.62	0.99
GA at T3	0.063	0.000	0.20	0.50	-0.037	0.001	0.48	0.91	-0.005	0.001	0.94	0.94	-0.045	0.001	0.38	0.76
PMA at scan	0.595	0.001	<0.001	**<0.001**	-0.592	0.002	<0.001	**<0.001**	-0.450	0.002	<0.001	**<0.001**	-0.614	0.003	<0.001	**<0.001**
**Inferior Cingulum Bundle**																
IL-8 T3	-0.068	0.001	0.25	0.63	-0.022	0.001	0.67	0.86	-0.070	0.002	0.24	0.92	0.004	0.002	0.93	0.93
Sex	0.025	0.002	0.67	0.84	-0.115	0.003	0.02	0.13	-0.081	0.004	0.17	0.22	-0.111	0.003	0.03	0.25
NICU stay	-0.097	0.006	0.15	0.57	-0.002	0.007	0.98	0.98	-0.065	0.010	0.34	0.80	0.051	0.008	0.39	0.84
GA at delivery	-0.072	0.001	0.35	0.93	0.135	0.001	0.05	0.43	0.072	0.002	0.36	0.58	0.148	0.001	0.03	0.24
GA at T3	-0.053	0.000	0.39	0.50	0.018	0.001	0.74	0.91	-0.020	0.001	0.75	0.94	0.038	0.001	0.48	0.76
PMA at scan	0.390	0.001	<0.001	**<0.001**	-0.626	0.001	<0.001	**<0.001**	-0.348	0.001	<0.001	**<0.001**	-0.641	0.001	<0.001	**<0.001**
**Fornix**																
IL-8 T3	-0.013	0.001	0.83	0.83	0.041	0.002	0.42	0.86	0.024	0.002	0.67	0.92	0.046	0.002	0.36	0.71
Sex	-0.106	0.002	0.07	0.31	-0.013	0.003	0.79	0.89	-0.067	0.004	0.23	0.26	0.023	0.003	0.64	0.83
NICU stay	-0.071	0.005	0.29	0.57	0.011	0.008	0.85	0.98	-0.027	0.011	0.68	0.87	0.032	0.008	0.58	0.84
GA at delivery	0.007	0.001	0.93	0.99	0.048	0.001	0.47	0.93	0.059	0.002	0.43	0.58	0.039	0.001	0.55	0.99
GA at T3	-0.089	0.000	0.15	0.50	0.045	0.001	0.40	0.91	-0.011	0.001	0.85	0.94	0.068	0.001	0.19	0.76
PMA at scan	0.357	0.001	<0.001	**<0.001**	-0.626	0.001	<0.001	**<0.001**	-0.473	0.002	<0.001	**<0.001**	-0.637	0.001	<0.001	**<0.001**

IL-10 Trimester 1

	FA				MD				AD				RD			
	**β**	**SE**	***p***	***q***	**β**	**SE**	***p***	***q***	**β**	**SE**	***p***	***q***	**β**	**SE**	***p***	***q***
**Corpus Callosum**																
IL-10 T1	0.017	0.002	0.78	0.83	0.012	0.002	0.85	1.00	0.064	0.002	0.37	0.69	-0.009	0.003	0.88	0.88
Sex	-0.126	0.004	0.05	0.25	0.066	0.004	0.28	0.68	-0.072	0.004	0.32	0.58	0.107	0.005	0.08	0.72
NICU stay	0.043	0.009	0.56	0.90	-0.056	0.009	0.43	0.86	0.112	0.010	0.17	0.58	-0.081	0.012	0.25	0.85
GA at delivery	-0.005	0.002	0.95	0.95	0.036	0.002	0.64	0.89	0.006	0.002	0.95	0.95	0.015	0.002	0.84	0.84
GA at T1	-0.038	0.001	0.55	0.86	0.063	0.001	0.31	0.94	0.037	0.001	0.61	0.32	0.060	0.001	0.32	0.84
PMA at scan	0.468	0.002	<0.001	**<0.001**	-0.527	0.002	<0.001	**<0.001**	-0.075	0.002	0.35	0.35	-0.534	0.002	<0.001	**<0.001**
**Superior Cingulum Bundle**																
IL-10 T1	-0.021	0.002	0.75	0.83	0.026	0.003	0.68	1.00	0.019	0.003	0.78	0.69	0.029	0.003	0.65	0.84
Sex	-0.112	0.003	0.10	0.30	-0.035	0.005	0.59	0.88	-0.128	0.006	0.06	0.13	0.010	0.006	0.88	0.89
NICU stay	0.032	0.008	0.68	0.90	0.058	0.012	0.43	0.86	0.071	0.014	0.37	0.61	0.038	0.014	0.61	0.85
GA at delivery	0.135	0.001	0.11	0.32	-0.009	0.002	0.91	0.91	0.083	0.002	0.32	0.88	-0.058	0.002	0.46	0.72
GA at T1	-0.026	0.001	0.70	0.87	-0.013	0.001	0.84	0.94	-0.045	0.001	0.50	0.93	-0.001	0.001	0.99	0.99
PMA at scan	0.285	0.001	<0.001	**<0.001**	-0.475	0.002	<0.001	**<0.001**	-0.373	0.002	<0.001	**<0.001**	-0.452	0.002	<0.001	**<0.001**
**Corticospinal Tract**																

IL-10 T1	-0.013	0.002	0.83	0.83	0.079	0.003	0.15	0.68	0.107	0.002	0.07	0.67	0.063	0.003	0.26	0.79
Sex	-0.073	0.003	0.24	0.36	0.013	0.005	0.82	0.94	-0.036	0.004	0.54	0.59	0.030	0.006	0.60	0.89
NICU stay	0.126	0.008	0.08	0.73	0.010	0.011	0.88	0.99	0.116	0.010	0.09	0.58	-0.027	0.013	0.67	0.85
GA at delivery	-0.042	0.001	0.58	0.65	0.062	0.002	0.36	0.81	0.057	0.002	0.44	0.88	0.058	0.002	0.40	0.72
GA at T1	0.018	0.001	0.77	0.87	0.026	0.001	0.64	0.94	0.064	0.001	0.29	0.76	0.011	0.001	0.84	0.95
PMA at scan	0.527	0.001	<0.001	**<0.001**	-0.662	0.002	<0.001	**<0.001**	-0.539	0.002	<0.001	**<0.001**	-0.651	0.002	<0.001	**<0.001**
**Optic Radiation**																
IL-10 T1	-0.017	0.002	0.77	0.83	0.053	0.003	0.38	0.93	0.058	0.003	0.40	0.69	0.048	0.004	0.41	0.79
Sex	-0.085	0.003	0.16	0.36	-0.055	0.006	0.37	0.68	-0.154	0.006	0.03	0.08	-0.018	0.007	0.76	0.89
NICU stay	-0.071	0.008	0.31	0.90	0.067	0.014	0.34	0.86	0.049	0.014	0.54	0.61	0.070	0.015	0.30	0.85
GA at delivery	-0.098	0.001	0.19	0.35	0.020	0.002	0.79	0.89	-0.034	0.002	0.69	0.88	0.040	0.003	0.58	0.72
GA at T1	-0.006	0.001	0.92	0.92	0.013	0.001	0.83	0.94	0.039	0.001	0.58	0.76	0.015	0.002	0.79	0.95
PMA at scan	0.564	0.001	<0.001	**<0.001**	-0.545	0.002	<0.001	**<0.001**	-0.265	0.002	<0.001	**<0.001**	-0.594	0.003	<0.001	**<0.001**
**Uncinate Fasciculus**																
IL-10 T1	0.041	0.002	0.50	0.83	-0.045	0.003	0.46	0.93	-0.026	0.003	0.71	0.78	-0.051	0.003	0.38	0.79
Sex	-0.119	0.003	0.06	0.25	-0.078	0.005	0.21	0.68	-0.171	0.006	0.01	0.06	-0.027	0.005	0.65	0.89
NICU stay	0.018	0.007	0.80	0.90	0.001	0.011	0.99	0.99	-0.004	0.014	0.96	0.96	0.005	0.012	0.94	0.94
GA at delivery	0.099	0.001	0.19	0.35	0.110	0.002	0.15	0.68	0.190	0.002	0.03	0.24	0.052	0.002	0.47	0.72
GA at T1	-0.098	0.001	0.11	0.84	0.029	0.001	0.64	0.94	-0.029	0.001	0.68	0.76	0.043	0.001	0.47	0.84
PMA at scan	0.445	0.001	<0.001	**<0.001**	-0.556	0.002	<0.001	**<0.001**	-0.305	0.002	<0.001	**<0.001**	-0.596	0.002	<0.001	**<0.001**
**Inferior Fronto-Occipital Fasciculus**																
IL-10 T1	-0.018	0.002	0.74	0.83	0.036	0.003	0.52	0.93	0.048	0.002	0.46	0.69	0.032	0.004	0.55	0.82
Sex	-0.051	0.003	0.35	0.40	-0.079	0.005	0.15	0.68	-0.171	0.005	0.01	0.06	-0.040	0.007	0.46	0.89
NICU stay	0.001	0.008	0.99	0.99	0.045	0.012	0.48	0.86	0.085	0.010	0.27	0.60	0.030	0.015	0.63	0.85
GA at delivery	0.050	0.001	0.46	0.65	-0.019	0.002	0.78	0.89	0.039	0.002	0.63	0.88	-0.031	0.003	0.64	0.72
GA at T1	-0.051	0.001	0.35	0.84	0.025	0.001	0.65	0.94	-0.011	0.001	0.87	0.87	0.042	0.002	0.44	0.84
PMA at scan	0.618	0.001	<0.001	**<0.001**	-0.636	0.002	<0.001	**<0.001**	-0.396	0.002	<0.001	**<0.001**	-0.650	0.003	<0.001	**<0.001**
**Anterior Limb of Internal Capsule**																
IL-10 T1	-0.030	0.002	0.59	0.83	0.000	0.004	1.00	1.00	-0.026	0.003	0.70	0.78	0.009	0.004	0.88	0.88
Sex	-0.069	0.003	0.21	0.36	-0.003	0.007	0.96	0.96	-0.041	0.006	0.54	0.59	0.012	0.007	0.83	0.89
NICU stay	0.017	0.007	0.79	0.90	0.067	0.015	0.33	0.86	0.101	0.014	0.19	0.58	0.051	0.017	0.43	0.85
GA at delivery	0.114	0.001	0.10	0.32	-0.019	0.003	0.79	0.89	0.052	0.002	0.53	0.88	-0.042	0.003	0.55	0.72
GA at T1	-0.031	0.001	0.58	0.84	0.046	0.002	0.43	0.94	0.041	0.001	0.54	0.76	0.045	0.002	0.43	0.84
PMA at scan	0.585	0.001	<0.001	**<0.001**	-0.556	0.003	<0.001	**<0.001**	-0.375	0.002	<0.001	**<0.001**	-0.589	0.003	<0.001	**<0.001**
**Inferior Cingulum Bundle**																
IL-10 T1	-0.040	0.001	0.54	0.83	0.093	0.002	0.13	0.68	0.073	0.002	0.29	0.69	0.087	0.002	0.14	0.79
Sex	0.007	0.002	0.91	0.91	-0.055	0.003	0.38	0.68	-0.043	0.004	0.53	0.59	-0.048	0.003	0.42	0.89
NICU stay	0.078	0.006	0.31	0.90	0.005	0.007	0.94	0.99	0.051	0.010	0.52	0.61	-0.022	0.008	0.75	0.85

GA at delivery	-0.154	0.001	0.06	0.32	0.120	0.001	0.12	0.68	0.008	0.002	0.93	0.95	0.157	0.001	0.03	0.31
GA at T1	-0.071	0.001	0.29	0.84	-0.004	0.001	0.94	0.94	-0.044	0.001	0.53	0.76	0.021	0.001	0.73	0.95
PMA at scan	0.407	0.001	<0.001	**<0.001**	-0.564	0.001	<0.001	**<0.001**	-0.291	0.002	<0.001	**<0.001**	-0.606	0.001	<0.001	**<0.001**
**Fornix**																
IL-10 T1	0.116	0.001	0.09	0.77	-0.007	0.002	0.90	1.00	0.059	0.003	0.36	0.69	-0.044	0.002	0.44	0.79
Sex	-0.067	0.002	0.33	0.40	-0.012	0.003	0.83	0.94	-0.034	0.005	0.59	0.59	0.008	0.003	0.89	0.89
NICU stay	0.033	0.005	0.67	0.90	0.032	0.008	0.63	0.95	0.045	0.011	0.55	0.61	0.021	0.008	0.75	0.85
GA at delivery	-0.054	0.001	0.52	0.65	0.071	0.001	0.32	0.81	0.045	0.002	0.57	0.88	0.081	0.001	0.25	0.72
GA at T1	-0.060	0.000	0.38	0.84	0.086	0.001	0.14	0.94	0.082	0.001	0.20	0.76	0.085	0.001	0.14	0.84
PMA at scan	0.349	0.001	<0.001	**<0.001**	-0.612	0.001	<0.001	**<0.001**	-0.456	0.002	<0.001	**<0.001**	-0.631	0.001	<0.001	**<0.001**
**IL-10 Trimester 2**																
	FA				MD				AD				RD			
	**β**	**SE**	***p***	***q***	**β**	**SE**	***p***	***q***	**β**	**SE**	***p***	***q***	**β**	**SE**	***p***	***q***
**Corpus Callosum**																
IL-10 T2	-0.143	0.002	0.02	**0.04**	0.136	0.002	0.02	0.06	-0.033	0.002	0.64	0.77	0.158	0.003	<0.01	**0.01**
Sex	-0.075	0.004	0.21	0.88	-0.024	0.004	0.67	0.67	-0.105	0.004	0.14	0.19	0.026	0.005	0.65	0.72
NICU stay	-0.047	0.009	0.52	0.89	-0.060	0.009	0.41	0.81	0.023	0.010	0.79	0.88	-0.049	0.012	0.49	0.75
GA at delivery	0.093	0.001	0.23	0.42	-0.072	0.001	0.36	0.76	0.155	0.001	0.11	0.49	-0.127	0.002	0.10	0.45
GA at T2	0.043	0.001	0.48	0.79	0.003	0.001	0.96	0.96	0.032	0.001	0.66	0.74	-0.017	0.001	0.77	0.99
PMA at scan	0.473	0.001	<0.001	**<0.001**	-0.573	0.001	<0.001	**<0.001**	-0.185	0.002	0.02	**0.02**	-0.557	0.002	<0.001	**<0.001**
**Superior Cingulum Bundle**																
IL-10 T2	0.011	0.002	0.86	0.86	0.023	0.003	0.71	0.80	0.042	0.003	0.52	0.77	0.010	0.003	0.87	0.87
Sex	-0.028	0.003	0.68	0.88	-0.058	0.005	0.36	0.40	-0.110	0.006	0.10	0.18	-0.027	0.006	0.66	0.72
NICU stay	0.012	0.007	0.89	0.89	0.056	0.012	0.48	0.81	0.052	0.014	0.54	0.88	0.045	0.014	0.57	0.75
GA at delivery	0.019	0.001	0.83	0.83	0.119	0.002	0.16	0.76	0.126	0.002	0.16	0.49	0.093	0.002	0.27	0.60
GA at T2	-0.042	0.000	0.53	0.79	0.055	0.001	0.39	0.75	0.041	0.001	0.54	0.70	0.050	0.001	0.44	0.98
PMA at scan	0.373	0.001	<0.001	**<0.001**	-0.522	0.002	<0.001	**<0.001**	-0.379	0.002	<0.001	**<0.001**	-0.513	0.002	<0.001	**<0.001**
**Corticospinal Tract**																
IL-10 T2	-0.231	0.002	<0.001	**<0.001**	0.175	0.003	<0.01	**<0.01**	0.047	0.002	0.40	0.77	0.197	0.003	<0.001	**<0.01**
Sex	0.058	0.003	0.30	0.88	-0.076	0.005	0.13	0.30	-0.071	0.004	0.21	0.24	-0.072	0.006	0.17	0.37
NICU stay	-0.031	0.008	0.66	0.89	0.007	0.012	0.92	0.92	0.039	0.010	0.59	0.88	0.004	0.014	0.95	0.95
GA at delivery	0.066	0.001	0.38	0.57	-0.046	0.002	0.51	0.76	0.029	0.001	0.70	0.70	-0.038	0.002	0.58	0.74
GA at T2	0.050	0.000	0.38	0.79	0.013	0.001	0.81	0.91	0.049	0.001	0.39	0.59	-0.007	0.001	0.89	0.99
PMA at scan	0.540	0.001	<0.001	**<0.001**	-0.673	0.002	<0.001	**<0.001**	-0.618	0.002	<0.001	**<0.001**	-0.657	0.002	<0.001	**<0.001**
**Optic Radiation**																
IL-10 T2	-0.123	0.002	0.02	**0.04**	0.115	0.003	0.03	0.06	0.055	0.003	0.39	0.77	0.125	0.003	0.01	**0.03**
Sex	0.008	0.004	0.88	0.91	-0.163	0.006	<0.01	**0.01**	-0.231	0.006	<0.001	**<0.01**	-0.127	0.006	0.01	0.06
NICU stay	-0.090	0.008	0.20	0.89	0.087	0.013	0.19	0.81	0.051	0.014	0.54	0.88	0.094	0.014	0.14	0.75

GA at delivery	0.053	0.001	0.48	0.62	-0.085	0.002	0.23	0.76	-0.041	0.002	0.64	0.70	-0.087	0.002	0.20	0.60
GA at T2	0.074	0.000	0.19	0.79	0.043	0.001	0.42	0.75	0.103	0.001	0.12	0.37	0.014	0.001	0.78	0.99
PMA at scan	0.558	0.001	<0.001	**<0.001**	-0.597	0.002	<0.001	**<0.001**	-0.325	0.002	<0.001	**<0.001**	-0.631	0.002	<0.001	**<0.001**
**Uncinate Fasciculus**																
IL-10 T2	-0.089	0.002	0.11	0.13	-0.044	0.003	0.47	0.67	-0.110	0.003	0.11	0.48	-0.015	0.003	0.79	0.87
Sex	-0.023	0.003	0.69	0.88	-0.067	0.005	0.27	0.35	-0.100	0.006	0.15	0.19	-0.048	0.005	0.41	0.61
NICU stay	-0.025	0.007	0.73	0.89	0.034	0.011	0.66	0.81	0.013	0.014	0.88	0.88	0.040	0.012	0.58	0.75
GA at delivery	0.123	0.001	0.11	0.31	0.062	0.002	0.46	0.76	0.167	0.002	0.07	0.49	0.003	0.002	0.97	0.97
GA at T2	0.019	0.000	0.74	0.79	0.085	0.001	0.17	0.70	0.110	0.001	0.12	0.37	0.057	0.001	0.33	0.98
PMA at scan	0.524	0.001	<0.001	**<0.001**	-0.529	0.002	<0.001	**<0.001**	-0.220	0.002	<0.001	**<0.001**	-0.592	0.002	<0.001	**<0.001**
**Inferior Fronto-Occipital Fasciculus**																
IL-10 T2	-0.124	0.002	0.01	**0.04**	0.054	0.003	0.30	0.54	-0.032	0.002	0.62	0.77	0.079	0.004	0.12	0.22
Sex	0.025	0.003	0.63	0.88	-0.132	0.006	0.01	**0.04**	-0.209	0.004	<0.01	**<0.01**	-0.097	0.007	0.06	0.18
NICU stay	-0.033	0.008	0.60	0.89	0.059	0.013	0.38	0.81	0.055	0.010	0.50	0.88	0.055	0.016	0.39	0.75
GA at delivery	0.101	0.001	0.14	0.31	-0.019	0.002	0.79	0.79	0.101	0.002	0.25	0.56	-0.047	0.002	0.50	0.74
GA at T2	0.053	0.000	0.31	0.79	0.024	0.001	0.65	0.91	0.118	0.001	0.07	0.37	0.000	0.001	0.99	0.99
PMA at scan	0.634	0.001	<0.001	**<0.001**	-0.643	0.002	<0.001	**<0.001**	-0.410	0.002	<0.001	**<0.001**	-0.655	0.002	<0.001	**<0.001**
**Anterior Limb of Internal Capsule**																
IL-10 T2	-0.111	0.001	0.02	**0.04**	0.036	0.003	0.52	0.67	-0.018	0.003	0.77	0.77	0.053	0.004	0.32	0.48
Sex	0.023	0.003	0.64	0.88	-0.065	0.007	0.25	0.35	-0.076	0.006	0.25	0.25	-0.058	0.008	0.29	0.52
NICU stay	-0.048	0.007	0.45	0.89	0.056	0.015	0.44	0.81	0.039	0.013	0.64	0.88	0.058	0.017	0.40	0.75
GA at delivery	0.165	0.001	0.02	0.14	-0.024	0.002	0.75	0.79	0.084	0.002	0.34	0.60	-0.059	0.003	0.42	0.74
GA at T2	-0.024	0.000	0.64	0.79	0.069	0.001	0.23	0.70	0.093	0.001	0.16	0.37	0.059	0.001	0.29	0.98
PMA at scan	0.617	0.001	<0.001	**<0.001**	-0.590	0.002	<0.001	**<0.001**	-0.427	0.002	<0.001	**<0.001**	-0.617	0.003	<0.001	**<0.001**
**Inferior Cingulum Bundle**																
IL-10 T2	-0.147	0.001	0.02	**0.04**	0.129	0.002	0.02	0.06	0.023	0.002	0.72	0.77	0.159	0.002	<0.01	**0.01**
Sex	-0.007	0.003	0.91	0.91	-0.223	0.003	<0.001	**<0.01**	-0.220	0.004	<0.01	**<0.01**	-0.190	0.003	<0.01	**<0.01**
NICU stay	-0.103	0.006	0.20	0.89	0.043	0.007	0.54	0.81	-0.048	0.010	0.57	0.88	0.086	0.008	0.21	0.75
GA at delivery	-0.135	0.001	0.12	0.31	0.074	0.001	0.33	0.76	-0.047	0.001	0.60	0.70	0.125	0.001	0.09	0.45
GA at T2	-0.017	0.000	0.79	0.79	0.089	0.000	0.12	0.70	0.080	0.001	0.24	0.43	0.091	0.000	0.10	0.91
PMA at scan	0.446	0.001	<0.001	**<0.001**	-0.608	0.001	<0.001	**<0.001**	-0.297	0.001	<0.001	**<0.001**	-0.650	0.001	<0.001	**<0.001**
**Fornix**																
IL-10 T2	0.113	0.001	0.08	0.10	0.006	0.002	0.92	0.92	0.099	0.002	0.11	0.48	-0.043	0.002	0.43	0.55
Sex	-0.107	0.002	0.10	0.88	-0.061	0.003	0.27	0.35	-0.111	0.004	0.08	0.17	-0.020	0.003	0.72	0.72
NICU stay	0.018	0.005	0.82	0.89	-0.025	0.008	0.72	0.81	-0.023	0.010	0.77	0.88	-0.023	0.008	0.74	0.84
GA at delivery	0.048	0.001	0.59	0.66	0.036	0.001	0.63	0.79	0.071	0.002	0.40	0.60	0.014	0.001	0.85	0.74
GA at T2	0.019	0.000	0.78	0.79	-0.014	0.000	0.81	0.91	-0.007	0.001	0.91	0.91	-0.015	0.000	0.79	0.99
PMA at scan	0.354	0.001	<0.001	**<0.001**	-0.639	0.001	<0.001	**<0.001**	-0.493	0.002	<0.001	**<0.001**	-0.641	0.001	<0.001	**<0.001**

IL-10 Trimester 3																
	FA				MD				AD				RD			
	β	SE	*p*	*q*	β	SE	*p*	*q*	β	SE	*p*	*q*	β	SE	*p*	*q*
**Corpus Callosum**																
IL-10 T3	-0.081	0.002	0.14	0.21	0.053	0.002	0.31	0.47	-0.066	0.002	0.30	0.68	0.065	0.002	0.21	0.27
Sex	-0.127	0.003	0.02	0.16	0.006	0.003	0.91	0.91	-0.083	0.004	0.18	0.26	0.068	0.005	0.18	0.54
NICU stay	-0.029	0.009	0.63	0.66	-0.034	0.009	0.58	0.89	0.018	0.009	0.80	0.80	-0.018	0.012	0.76	0.88
GA at delivery	0.004	0.001	0.95	0.95	0.016	0.001	0.81	0.89	0.101	0.002	0.22	0.60	-0.012	0.002	0.86	0.92
GA at T3	0.070	0.001	0.21	0.37	-0.057	0.001	0.29	0.95	0.009	0.001	0.89	0.91	-0.076	0.001	0.15	0.62
PMA at scan	0.463	0.001	<0.001	**<0.001**	-0.564	0.001	<0.001	**<0.001**	-0.190	0.001	0.01	**0.01**	-0.558	0.002	<0.001	**<0.001**
**Superior Cingulum Bundle**																
IL-10 T3	-0.097	0.002	0.11	0.19	0.064	0.002	0.25	0.47	0.021	0.003	0.71	0.71	0.083	0.003	0.14	0.25
Sex	-0.065	0.003	0.27	0.60	-0.037	0.005	0.50	0.75	-0.106	0.005	0.07	0.15	-0.002	0.005	0.97	0.97
NICU stay	-0.065	0.008	0.34	0.66	0.014	0.012	0.83	0.89	-0.042	0.014	0.54	0.71	0.034	0.014	0.59	0.88
GA at delivery	0.017	0.001	0.82	0.93	0.085	0.002	0.23	0.89	0.095	0.002	0.21	0.60	0.065	0.002	0.37	0.92
GA at T3	-0.080	0.001	0.19	0.37	0.023	0.001	0.69	0.95	-0.036	0.001	0.56	0.91	0.045	0.001	0.44	0.68
PMA at scan	0.306	0.001	<0.001	**<0.001**	-0.514	0.002	<0.001	**<0.001**	-0.405	0.002	<0.001	**<0.001**	-0.485	0.002	<0.001	**<0.001**
**Corticospinal Tract**																
IL-10 T3	-0.228	0.002	<0.001	**<0.001**	0.108	0.002	0.02	0.09	-0.039	0.002	0.45	0.68	0.148	0.003	<0.01	**0.01**
Sex	0.007	0.003	0.89	0.91	-0.050	0.004	0.26	0.48	-0.073	0.004	0.16	0.26	-0.036	0.005	0.43	0.73
NICU stay	-0.064	0.008	0.26	0.66	0.028	0.011	0.60	0.89	-0.029	0.010	0.63	0.71	0.035	0.014	0.51	0.88
GA at delivery	-0.024	0.001	0.71	0.93	0.008	0.002	0.89	0.89	-0.003	0.002	0.97	0.97	0.034	0.002	0.55	0.92
GA at T3	0.086	0.001	0.10	0.37	-0.045	0.001	0.35	0.95	0.017	0.001	0.75	0.91	-0.060	0.001	0.21	0.62
PMA at scan	0.520	0.001	<0.001	**<0.001**	-0.659	0.002	<0.001	**<0.001**	-0.571	0.002	<0.001	**<0.001**	-0.645	0.002	<0.001	**<0.001**
**Optic Radiation**																
IL-10 T3	-0.058	0.002	0.27	0.30	0.069	0.003	0.17	0.47	0.047	0.003	0.43	0.68	0.073	0.003	0.13	0.25
Sex	-0.006	0.003	0.91	0.91	-0.111	0.005	0.03	0.12	-0.174	0.005	<0.01	**0.03**	-0.081	0.006	0.09	0.42
NICU stay	-0.081	0.009	0.18	0.66	0.077	0.013	0.19	0.83	0.035	0.014	0.62	0.71	0.085	0.015	0.13	0.63
GA at delivery	-0.018	0.001	0.79	0.93	-0.032	0.002	0.62	0.89	-0.046	0.002	0.55	0.62	-0.018	0.002	0.77	0.92
GA at T3	0.047	0.001	0.39	0.44	0.017	0.001	0.75	0.95	0.073	0.001	0.24	0.91	-0.004	0.001	0.94	0.96
PMA at scan	0.541	0.001	<0.001	**<0.001**	-0.556	0.002	<0.001	**<0.001**	-0.262	0.002	<0.001	**<0.001**	-0.598	0.002	<0.001	**<0.001**
**Uncinate Fasciculus**																
IL-10 T3	-0.062	0.001	0.25	0.30	-0.127	0.002	0.02	0.09	-0.187	0.003	<0.01	**0.01**	-0.079	0.002	0.13	0.25
Sex	-0.060	0.003	0.26	0.60	-0.067	0.004	0.21	0.48	-0.116	0.005	0.05	0.14	-0.036	0.005	0.48	0.73
NICU stay	-0.049	0.007	0.43	0.66	-0.130	0.011	0.04	0.34	-0.176	0.014	0.01	0.09	-0.085	0.012	0.16	0.63
GA at delivery	0.122	0.001	0.08	0.49	0.029	0.002	0.68	0.89	0.114	0.002	0.13	0.60	-0.020	0.002	0.76	0.92
GA at T3	-0.020	0.000	0.72	0.72	-0.001	0.001	0.98	0.98	-0.008	0.001	0.90	0.91	0.003	0.001	0.96	0.96
PMA at scan	0.452	0.001	<0.001	**<0.001**	-0.533	0.002	<0.001	**<0.001**	-0.278	0.002	<0.001	**<0.001**	-0.578	0.002	<0.001	**<0.001**
**Inferior Fronto-Occipital Fasciculus**																

IL-10 T3	-0.117	0.002	0.01	0.06	0.029	0.002	0.55	0.66	-0.073	0.002	0.21	0.62	0.059	0.003	0.21	0.27
Sex	-0.023	0.003	0.62	0.80	-0.086	0.005	0.07	0.22	-0.158	0.004	0.01	**0.03**	-0.052	0.006	0.26	0.60
NICU stay	-0.040	0.008	0.46	0.66	-0.008	0.013	0.89	0.89	-0.037	0.010	0.58	0.71	0.008	0.015	0.88	0.88
GA at delivery	0.069	0.001	0.26	0.62	-0.021	0.002	0.74	0.89	0.063	0.002	0.40	0.60	-0.037	0.002	0.55	0.92
GA at T3	0.048	0.001	0.33	0.42	-0.014	0.001	0.78	0.95	0.037	0.001	0.54	0.91	-0.028	0.001	0.57	0.74
PMA at scan	0.591	0.001	<0.001	**<0.001**	-0.628	0.002	<0.001	**<0.001**	-0.405	0.002	<0.001	**<0.001**	-0.638	0.002	<0.001	**<0.001**
**Anterior Limb of Internal Capsule**																
IL-10 T3	-0.082	0.001	0.08	0.18	-0.022	0.003	0.66	0.66	-0.098	0.003	0.09	0.40	0.003	0.003	0.95	0.95
Sex	-0.045	0.003	0.33	0.60	-0.020	0.006	0.69	0.85	-0.043	0.005	0.44	0.44	-0.007	0.007	0.88	0.97
NICU stay	-0.025	0.007	0.64	0.66	-0.031	0.015	0.59	0.89	-0.072	0.013	0.27	0.71	-0.015	0.017	0.79	0.88
GA at delivery	0.097	0.001	0.11	0.49	-0.013	0.002	0.84	0.89	0.068	0.002	0.35	0.60	-0.034	0.003	0.58	0.92
GA at T3	0.056	0.000	0.25	0.37	-0.033	0.001	0.53	0.95	-0.007	0.001	0.91	0.91	-0.040	0.001	0.43	0.68
PMA at scan	0.589	0.001	<0.001	**<0.001**	-0.600	0.002	<0.001	**<0.001**	-0.465	0.002	<0.001	**<0.001**	-0.619	0.003	<0.001	**<0.001**
**Inferior Cingulum Bundle**																
IL-10 T3	-0.130	0.001	0.03	0.08	0.057	0.001	0.28	0.47	-0.032	0.002	0.59	0.71	0.086	0.002	0.09	0.25
Sex	0.048	0.002	0.40	0.60	-0.125	0.003	0.02	0.12	-0.075	0.004	0.21	0.26	-0.126	0.003	0.01	0.11
NICU stay	-0.134	0.006	0.05	0.42	0.010	0.007	0.86	0.89	-0.081	0.009	0.24	0.71	0.074	0.008	0.21	0.63
GA at delivery	-0.082	0.001	0.28	0.62	0.135	0.001	0.04	0.40	0.067	0.001	0.38	0.60	0.151	0.001	0.02	0.21
GA at T3	-0.072	0.000	0.23	0.37	0.011	0.001	0.84	0.95	-0.042	0.001	0.50	0.91	0.040	0.001	0.45	0.68
PMA at scan	0.363	0.001	<0.001	**<0.001**	-0.603	0.001	<0.001	**<0.001**	-0.344	0.001	<0.001	**<0.001**	-0.612	0.001	<0.001	**0.00**
**Fornix**																
IL-10 T3	-0.028	0.001	0.64	0.64	0.027	0.002	0.59	0.66	0.024	0.002	0.68	0.71	0.029	0.002	0.56	0.63
Sex	-0.082	0.002	0.16	0.60	-0.015	0.003	0.76	0.85	-0.057	0.004	0.31	0.35	0.013	0.003	0.79	0.97
NICU stay	-0.030	0.005	0.66	0.66	-0.028	0.008	0.63	0.89	-0.049	0.010	0.45	0.71	-0.012	0.008	0.83	0.88
GA at delivery	0.033	0.001	0.66	0.93	0.021	0.001	0.75	0.89	0.047	0.002	0.51	0.62	0.007	0.001	0.92	0.92
GA at T3	-0.084	0.000	0.17	0.37	0.045	0.001	0.39	0.95	-0.009	0.001	0.88	0.91	0.066	0.001	0.20	0.62
PMA at scan	0.355	0.001	<0.001	**<0.001**	-0.621	0.001	<0.001	**<0.001**	-0.475	0.002	<0.001	**<0.001**	-0.629	0.001	<0.001	**<0.001**

TNF-α Trimester 1

	FA				MD				AD				RD			
	**β**	**SE**	***p***	***q***	**β**	**SE**	***p***	***q***	**β**	**SE**	***p***	***q***	**β**	**SE**	***p***	***q***
**Corpus Callosum**																
TNF-α T1	-0.007	0.002	0.91	0.91	0.008	0.002	0.89	0.90	0.031	0.002	0.67	0.95	-0.005	0.003	0.94	0.94
Sex	-0.138	0.004	0.03	0.28	0.076	0.004	0.22	0.63	-0.075	0.004	0.30	0.55	0.118	0.005	0.05	0.49
NICU stay	0.039	0.009	0.60	0.92	-0.068	0.009	0.35	0.71	0.088	0.010	0.28	0.66	-0.085	0.012	0.23	0.79
GA at delivery	-0.004	0.002	0.96	0.96	0.012	0.002	0.87	0.92	-0.027	0.002	0.76	0.85	0.001	0.002	0.99	0.99
GA at T1	-0.041	0.001	0.52	0.84	0.074	0.001	0.23	0.94	0.050	0.001	0.49	0.75	0.068	0.001	0.27	0.91
PMA at scan	0.456	0.002	<0.001	**<0.001**	-0.517	0.002	<0.001	**<0.001**	-0.069	0.002	0.39	0.39	-0.522	0.002	<0.001	**<0.001**
**Superior Cingulum Bundle**																
TNF-α T1	-0.008	0.002	0.90	0.91	-0.008	0.003	0.90	0.90	0.006	0.004	0.93	0.95	-0.008	0.003	0.90	0.94

Sex	-0.108	0.003	0.12	0.34	-0.043	0.005	0.50	0.75	-0.134	0.006	0.05	0.11	0.001	0.006	0.99	0.99
NICU stay	0.022	0.008	0.78	0.92	0.068	0.012	0.37	0.71	0.073	0.014	0.36	0.66	0.048	0.014	0.52	0.79
GA at delivery	0.115	0.001	0.18	0.40	0.011	0.002	0.89	0.92	0.092	0.002	0.28	0.85	-0.035	0.002	0.66	0.85
GA at T1	-0.020	0.001	0.77	0.86	-0.019	0.001	0.77	0.94	-0.047	0.001	0.49	0.75	-0.007	0.001	0.91	0.91
PMA at scan	0.288	0.001	<0.001	**<0.001**	-0.476	0.002	<0.001	**<0.001**	-0.373	0.002	<0.001	**<0.001**	-0.454	0.002	<0.001	**<0.001**
**Corticospinal Tract**																
TNF-α T1	0.057	0.002	0.36	0.66	-0.007	0.003	0.90	0.90	0.054	0.002	0.37	0.84	-0.027	0.003	0.64	0.94
Sex	-0.076	0.003	0.23	0.33	0.010	0.005	0.86	0.92	-0.042	0.004	0.49	0.55	0.029	0.006	0.62	0.96
NICU stay	0.113	0.008	0.12	0.81	0.011	0.011	0.86	0.96	0.103	0.010	0.14	0.66	-0.022	0.014	0.74	0.84
GA at delivery	-0.058	0.001	0.46	0.37	0.069	0.002	0.32	0.72	0.053	0.002	0.48	0.85	0.069	0.002	0.33	0.82
GA at T1	0.028	0.001	0.65	0.82	0.025	0.001	0.66	0.94	0.071	0.001	0.24	0.75	0.007	0.001	0.91	0.91
PMA at scan	0.521	0.001	<0.001	**<0.001**	-0.662	0.002	<0.001	**<0.001**	-0.546	0.002	<0.001	**<0.001**	-0.650	0.002	<0.001	**<0.001**
**Optic Radiation**																
TNF-α T1	0.063	0.002	0.30	0.92	0.011	0.003	0.86	0.90	0.069	0.003	0.31	0.84	-0.007	0.004	0.90	0.94
Sex	-0.084	0.003	0.17	0.16	-0.066	0.006	0.28	0.63	-0.168	0.006	0.02	0.05	-0.027	0.007	0.64	0.96
NICU stay	-0.087	0.008	0.22	0.81	0.067	0.014	0.34	0.71	0.037	0.014	0.65	0.73	0.074	0.015	0.27	0.79
GA at delivery	-0.115	0.001	0.12	0.40	0.032	0.002	0.67	0.92	-0.032	0.002	0.71	0.85	0.055	0.003	0.45	0.82
GA at T1	0.000	0.001	1.00	1.00	0.013	0.001	0.83	0.94	0.042	0.001	0.54	0.75	0.014	0.002	0.81	0.91
PMA at scan	0.567	0.001	<0.001	**<0.001**	-0.561	0.002	<0.001	**<0.001**	-0.286	0.002	<0.001	**<0.001**	-0.607	0.003	<0.001	**<0.001**
**Uncinate Fasciculus**																
TNF-α T1	-0.086	0.002	0.16	0.54	0.053	0.003	0.39	0.90	-0.004	0.003	0.95	0.95	0.068	0.003	0.25	0.75
Sex	-0.115	0.003	0.06	0.29	-0.080	0.005	0.20	0.63	-0.169	0.006	0.02	0.05	-0.030	0.005	0.61	0.96
NICU stay	0.022	0.007	0.76	0.92	0.004	0.011	0.96	0.96	0.002	0.014	0.98	0.98	0.007	0.012	0.92	0.92
GA at delivery	0.087	0.001	0.26	0.47	0.121	0.002	0.12	0.54	0.192	0.002	0.03	0.25	0.067	0.002	0.37	0.82
GA at T1	-0.091	0.001	0.14	0.84	0.022	0.001	0.72	0.94	-0.030	0.001	0.66	0.75	0.035	0.001	0.56	0.91
PMA at scan	0.455	0.001	<0.001	**<0.001**	-0.556	0.002	<0.001	**<0.001**	-0.297	0.002	<0.001	**<0.001**	-0.600	0.002	<0.001	**<0.001**
**Inferior Fronto-Occipital Fasciculus**																
TNF-α T1	0.054	0.002	0.33	0.54	0.008	0.003	0.89	0.90	0.092	0.003	0.16	0.83	-0.013	0.004	0.81	0.94
Sex	-0.048	0.003	0.39	0.44	-0.084	0.005	0.13	0.63	-0.175	0.005	0.01	0.05	-0.044	0.007	0.42	0.96
NICU stay	-0.017	0.008	0.79	0.92	0.050	0.012	0.44	0.71	0.070	0.010	0.36	0.66	0.040	0.015	0.53	0.79
GA at delivery	0.026	0.001	0.71	0.79	-0.007	0.002	0.92	0.92	0.032	0.002	0.70	0.85	-0.015	0.003	0.82	0.92
GA at T1	-0.045	0.001	0.42	0.84	0.023	0.001	0.67	0.94	-0.007	0.001	0.92	0.92	0.039	0.002	0.47	0.91
PMA at scan	0.628	0.001	<0.001	**<0.001**	-0.641	0.002	<0.001	**<0.001**	-0.389	0.002	<0.001	**<0.001**	-0.656	0.003	<0.001	**<0.001**
**Anterior Limb of Internal Capsule**																
TNF-α T1	0.057	0.002	0.30	0.54	-0.010	0.004	0.87	0.90	0.030	0.003	0.66	0.95	-0.024	0.004	0.68	0.94
Sex	-0.061	0.003	0.27	0.35	-0.006	0.007	0.92	0.92	-0.040	0.006	0.55	0.55	0.008	0.008	0.89	0.99
NICU stay	0.010	0.007	0.88	0.92	0.068	0.015	0.33	0.71	0.096	0.014	0.22	0.66	0.054	0.017	0.42	0.79
GA at delivery	0.104	0.001	0.13	0.40	-0.020	0.003	0.79	0.92	0.043	0.002	0.61	0.85	-0.040	0.003	0.57	0.85

GA at T1	-0.030	0.001	0.59	0.84	0.047	0.002	0.43	0.94	0.043	0.001	0.53	0.75	0.045	0.002	0.43	0.91
PMA at scan	0.589	0.001	<0.001	**<0.001**	-0.552	0.003	<0.001	**<0.001**	-0.367	0.002	<0.001	**<0.001**	-0.587	0.003	<0.001	**<0.001**
**Inferior Cingulum Bundle**																
TNF-α T1	-0.118	0.001	0.08	0.54	0.058	0.002	0.35	0.90	-0.014	0.002	0.84	0.95	0.088	0.002	0.14	0.69
Sex	-0.006	0.002	0.93	0.93	-0.056	0.003	0.36	0.66	-0.053	0.004	0.45	0.55	-0.046	0.003	0.45	0.96
NICU stay	0.086	0.006	0.27	0.81	0.004	0.007	0.96	0.96	0.052	0.010	0.52	0.69	-0.024	0.008	0.73	0.84
GA at delivery	-0.158	0.001	0.06	0.40	0.132	0.001	0.09	0.54	0.016	0.002	0.85	0.85	0.170	0.001	0.02	0.22
GA at T1	-0.067	0.001	0.31	0.84	-0.002	0.001	0.98	0.98	-0.037	0.001	0.59	0.75	0.021	0.001	0.72	0.91
PMA at scan	0.399	0.001	<0.001	**<0.001**	-0.569	0.001	<0.001	**<0.001**	-0.303	0.002	<0.001	**<0.001**	-0.608	0.001	<0.001	**<0.001**
**Fornix**																
TNF-α T1	-0.008	0.001	0.90	0.91	-0.092	0.002	0.11	0.90	-0.085	0.003	0.18	0.83	-0.082	0.002	0.15	0.69
Sex	-0.090	0.002	0.19	0.34	-0.015	0.003	0.79	0.92	-0.048	0.005	0.45	0.55	0.013	0.003	0.83	0.99
NICU stay	0.008	0.005	0.92	0.92	0.049	0.008	0.47	0.71	0.046	0.011	0.54	0.69	0.044	0.008	0.51	0.79
GA at delivery	-0.076	0.001	0.37	0.56	0.084	0.001	0.25	0.72	0.044	0.002	0.58	0.85	0.100	0.001	0.16	0.73
GA at T1	-0.046	0.000	0.50	0.84	0.080	0.001	0.17	0.94	0.085	0.001	0.19	0.75	0.076	0.001	0.19	0.91
PMA at scan	0.327	0.001	<0.001	**<0.001**	-0.610	0.001	<0.001	**<0.001**	-0.464	0.002	<0.001	**<0.001**	-0.625	0.001	<0.001	**<0.001**
**TNF-α Trimester 2**																
	**FA**				**MD**				**AD**				**RD**			
	**β**	**SE**	***p***	***q***	**β**	**SE**	***p***	***q***	**β**	**SE**	***p***	***q***	**β**	**SE**	***p***	***q***
**Corpus Callosum**																
TNF-α T2	-0.102	0.002	0.09	0.17	0.060	0.002	0.30	0.81	-0.046	0.002	0.51	0.77	0.086	0.003	0.13	0.39
Sex	-0.084	0.004	0.17	0.75	-0.019	0.004	0.75	0.81	-0.097	0.004	0.18	0.28	0.035	0.005	0.54	0.81
NICU stay	-0.079	0.009	0.28	0.51	-0.030	0.010	0.69	0.85	0.031	0.010	0.73	0.99	-0.006	0.013	0.93	0.99
GA at delivery	0.068	0.001	0.39	0.70	-0.055	0.001	0.50	0.84	0.157	0.001	0.11	0.47	-0.095	0.002	0.23	0.69
GA at T2	0.017	0.001	0.78	0.88	0.007	0.001	0.91	0.91	0.037	0.001	0.61	0.69	0.010	0.001	0.87	0.98
PMA at scan	0.488	0.001	<0.001	**<0.001**	-0.570	0.001	<0.001	**<0.001**	-0.190	0.002	0.01	**0.01**	-0.565	0.002	<0.001	**<0.001**
**Superior Cingulum Bundle**																
TNF-α T2	0.015	0.002	0.82	0.92	0.030	0.003	0.63	0.81	0.051	0.003	0.45	0.77	0.020	0.003	0.75	0.75
Sex	-0.058	0.003	0.39	0.86	-0.015	0.005	0.81	0.81	-0.081	0.006	0.23	0.29	0.015	0.006	0.81	0.91
NICU stay	-0.014	0.008	0.87	0.96	0.077	0.012	0.32	0.70	0.062	0.014	0.46	0.99	0.068	0.014	0.38	0.69
GA at delivery	0.007	0.001	0.94	0.94	0.124	0.002	0.14	0.84	0.127	0.002	0.16	0.47	0.100	0.002	0.23	0.69
GA at T2	-0.067	0.000	0.32	0.82	0.069	0.001	0.27	0.49	0.049	0.001	0.47	0.61	0.066	0.001	0.29	0.88
PMA at scan	0.392	0.001	<0.001	**<0.001**	-0.539	0.002	<0.001	**<0.001**	-0.389	0.002	<0.001	**<0.001**	-0.529	0.002	<0.001	**<0.001**
**Corticospinal Tract**																
TNF-α T2	-0.099	0.002	0.08	0.17	0.101	0.003	0.05	0.42	0.047	0.002	0.40	0.77	0.102	0.003	0.05	0.38
Sex	0.033	0.004	0.57	0.86	-0.047	0.005	0.36	0.65	-0.047	0.004	0.40	0.40	-0.043	0.006	0.42	0.76
NICU stay	-0.066	0.008	0.36	0.54	0.035	0.012	0.58	0.85	0.056	0.010	0.42	0.99	0.033	0.015	0.61	0.85
GA at delivery	0.041	0.001	0.59	0.74	-0.031	0.002	0.66	0.84	0.032	0.001	0.67	0.68	-0.019	0.002	0.78	0.89
GA at T2	0.044	0.000	0.45	0.82	0.022	0.001	0.67	0.87	0.066	0.001	0.25	0.38	0.000	0.001	0.99	0.99

PMA at scan	0.552	0.001	<0.001	**<0.001**	-0.686	0.002	<0.001	**<0.001**	-0.631	0.001	<0.001	**<0.001**	-0.669	0.002	<0.001	**<0.001**
**Optic Radiation**																
TNF-α T2	-0.093	0.002	0.09	0.17	0.059	0.003	0.26	0.81	0.026	0.003	0.69	0.77	0.068	0.003	0.17	0.39
Sex	-0.017	0.004	0.76	0.86	-0.138	0.006	0.01	**0.04**	-0.217	0.006	<0.01	**0.01**	-0.100	0.006	0.05	0.22
NICU stay	-0.113	0.008	0.11	0.51	0.120	0.013	0.07	0.62	0.072	0.014	0.38	0.99	0.126	0.015	0.05	0.37
GA at delivery	0.034	0.001	0.65	0.74	-0.069	0.002	0.33	0.84	-0.036	0.002	0.68	0.68	-0.069	0.002	0.31	0.69
GA at T2	0.062	0.001	0.28	0.82	0.062	0.001	0.25	0.49	0.112	0.001	0.09	0.25	0.034	0.001	0.51	0.91
PMA at scan	0.566	0.001	<0.001	**<0.001**	-0.615	0.002	<0.001	**<0.001**	-0.341	0.002	<0.001	**<0.001**	-0.646	0.002	<0.001	**<0.001**
**Uncinate Fasciculus**																
TNF-α T2	-0.146	0.002	0.01	0.05	0.009	0.003	0.88	0.88	-0.098	0.003	0.15	0.77	0.048	0.003	0.40	0.71
Sex	-0.059	0.003	0.31	0.86	-0.038	0.005	0.54	0.69	-0.092	0.006	0.19	0.28	-0.014	0.005	0.81	0.91
NICU stay	-0.029	0.007	0.69	0.89	0.024	0.012	0.76	0.85	0.001	0.014	0.99	0.99	0.032	0.013	0.66	0.85
GA at delivery	0.107	0.001	0.16	0.49	0.056	0.002	0.50	0.84	0.150	0.002	0.11	0.47	0.002	0.002	0.98	0.98
GA at T2	0.031	0.000	0.59	0.88	0.072	0.001	0.26	0.49	0.105	0.001	0.14	0.25	0.044	0.001	0.46	0.91
PMA at scan	0.532	0.001	<0.001	**<0.001**	-0.533	0.002	<0.001	**<0.001**	-0.220	0.002	<0.001	**<0.001**	-0.596	0.002	<0.001	**<0.001**
**Inferior Fronto-Occipital Fasciculus**																
TNF-α T2	-0.043	0.002	0.39	0.59	0.010	0.003	0.84	0.88	-0.008	0.002	0.90	0.90	0.021	0.004	0.67	0.75
Sex	0.002	0.004	0.97	0.97	-0.104	0.006	0.05	0.16	-0.183	0.004	0.01	**0.02**	-0.071	0.007	0.17	0.52
NICU stay	-0.074	0.008	0.25	0.51	0.078	0.013	0.24	0.70	0.040	0.010	0.62	0.99	0.082	0.017	0.21	0.62
GA at delivery	0.077	0.001	0.26	0.59	-0.012	0.002	0.87	0.87	0.087	0.002	0.32	0.66	-0.034	0.002	0.62	0.89
GA at T2	0.039	0.001	0.45	0.82	0.040	0.001	0.47	0.70	0.129	0.001	0.05	0.25	0.015	0.001	0.78	0.98
PMA at scan	0.645	0.001	<0.001	**<0.001**	-0.650	0.002	<0.001	**<0.001**	-0.413	0.002	<0.001	**<0.001**	-0.664	0.003	<0.001	**<0.001**
**Anterior Limb of Internal Capsule**																
TNF-α T2	-0.003	0.002	0.96	0.96	-0.032	0.004	0.57	0.81	-0.045	0.003	0.49	0.77	-0.026	0.004	0.63	0.75
Sex	0.019	0.003	0.71	0.86	-0.056	0.007	0.33	0.65	-0.063	0.006	0.34	0.38	-0.049	0.008	0.37	0.76
NICU stay	-0.084	0.007	0.19	0.51	0.062	0.016	0.39	0.70	0.025	0.014	0.76	0.99	0.070	0.018	0.30	0.69
GA at delivery	0.138	0.001	0.04	0.32	-0.024	0.002	0.75	0.84	0.068	0.002	0.44	0.66	-0.053	0.003	0.47	0.85
GA at T2	-0.039	0.000	0.45	0.82	0.090	0.001	0.12	0.49	0.117	0.001	0.08	0.25	0.078	0.001	0.16	0.72
PMA at scan	0.627	0.001	<0.001	**<0.001**	-0.591	0.002	<0.001	**<0.001**	-0.429	0.002	<0.001	**<0.001**	-0.619	0.003	<0.001	**<0.001**
**Inferior Cingulum Bundle**																
TNF-α T2	-0.183	0.001	<0.01	**0.04**	0.039	0.002	0.48	0.81	-0.090	0.002	0.18	0.77	0.094	0.002	0.08	0.38
Sex	-0.042	0.003	0.51	0.86	-0.189	0.003	<0.01	**0.01**	-0.211	0.004	<0.01	**0.01**	-0.152	0.003	0.01	0.06
NICU stay	-0.119	0.006	0.13	0.51	0.078	0.007	0.27	0.70	-0.023	0.010	0.79	0.99	0.120	0.008	0.08	0.37
GA at delivery	-0.154	0.001	0.07	0.32	0.091	0.001	0.23	0.84	-0.042	0.001	0.64	0.68	0.145	0.001	0.05	0.45
GA at T2	-0.006	0.000	0.93	0.93	0.102	0.000	0.08	0.49	0.103	0.001	0.13	0.25	0.096	0.000	0.09	0.72
PMA at scan	0.467	0.001	<0.001	**<0.001**	-0.617	0.001	<0.001	**<0.001**	-0.295	0.001	<0.001	**<0.001**	-0.663	0.001	<0.001	**<0.001**
**Fornix**																
TNF-α T2	-0.022	0.001	0.73	0.92	-0.031	0.002	0.57	0.81	-0.030	0.002	0.63	0.77	-0.021	0.002	0.69	0.75
Sex	-0.106	0.002	0.11	0.75	-0.036	0.003	0.52	0.69	-0.084	0.004	0.19	0.28	0.000	0.003	0.99	0.99

NICU stay	0.004	0.005	0.96	0.96	-0.001	0.008	0.99	0.99	-0.003	0.010	0.97	0.99	0.001	0.008	0.99	0.99
GA at delivery	0.039	0.001	0.66	0.74	0.039	0.001	0.60	0.84	0.072	0.002	0.40	0.66	0.020	0.001	0.79	0.89
GA at T2	-0.025	0.000	0.70	0.88	0.007	0.000	0.91	0.91	-0.011	0.001	0.87	0.87	0.016	0.000	0.78	0.98
PMA at scan	0.391	0.001	<0.001	**<0.001**	-0.648	0.001	<0.001	**<0.001**	-0.491	0.002	<0.001	**<0.001**	-0.657	0.001	<0.001	**<0.001**

TNF-α Trimester 3

	FA				MD				AD				RD			
	β	SE	*p*	*q*	β	SE	*p*	*q*	β	SE	*p*	*q*	β	SE	*p*	*q*
**Corpus Callosum**																
TNF-α T3	0.027	0.002	0.62	0.85	-0.087	0.002	0.10	0.44	-0.093	0.002	0.14	0.41	-0.068	0.002	0.19	0.84
Sex	-0.140	0.003	0.01	0.09	0.003	0.003	0.96	0.98	-0.107	0.004	0.09	0.15	0.071	0.005	0.17	0.50
NICU stay	-0.046	0.009	0.45	0.55	-0.025	0.009	0.68	0.94	0.009	0.009	0.90	0.90	-0.004	0.012	0.94	0.94
GA at delivery	0.003	0.001	0.97	0.97	0.004	0.001	0.95	0.99	0.087	0.002	0.28	0.58	-0.019	0.002	0.78	0.86
GA at T3	0.053	0.001	0.35	0.52	-0.043	0.001	0.43	0.94	0.007	0.001	0.91	0.99	-0.060	0.001	0.27	0.69
PMA at scan	0.461	0.001	<0.001	**<0.001**	-0.565	0.001	<0.001	**<0.001**	-0.189	0.001	0.01	**0.01**	-0.557	0.002	<0.001	**<0.001**
**Superior Cingulum Bundle**																
TNF-α T3	-0.016	0.002	0.79	0.85	0.010	0.002	0.85	0.95	0.007	0.003	0.90	0.93	0.011	0.003	0.85	0.96
Sex	-0.083	0.003	0.16	0.41	-0.023	0.005	0.67	0.86	-0.100	0.005	0.08	0.15	0.017	0.005	0.76	0.85
NICU stay	-0.069	0.008	0.32	0.55	0.017	0.012	0.79	0.94	-0.038	0.014	0.58	0.78	0.040	0.014	0.54	0.94
GA at delivery	0.019	0.001	0.81	0.97	0.085	0.002	0.23	0.97	0.098	0.002	0.19	0.58	0.066	0.002	0.36	0.86
GA at T3	-0.074	0.001	0.23	0.51	0.021	0.001	0.71	0.94	-0.031	0.001	0.61	0.99	0.043	0.001	0.46	0.69
PMA at scan	0.314	0.001	<0.001	**<0.001**	-0.519	0.002	<0.001	**<0.001**	-0.406	0.002	<0.001	**<0.001**	-0.493	0.002	<0.001	**<0.001**
**Corticospinal Tract**																
TNF-α T3	-0.036	0.002	0.48	0.85	-0.008	0.002	0.86	0.95	-0.041	0.002	0.43	0.65	0.002	0.003	0.97	0.97
Sex	-0.017	0.003	0.75	0.84	-0.044	0.004	0.34	0.61	-0.085	0.004	0.10	0.15	-0.023	0.005	0.62	0.79
NICU stay	-0.071	0.008	0.24	0.55	0.031	0.011	0.56	0.94	-0.030	0.010	0.62	0.78	0.039	0.014	0.47	0.94
GA at delivery	-0.015	0.001	0.82	0.97	0.000	0.002	0.99	0.99	-0.007	0.002	0.92	0.92	0.028	0.002	0.64	0.86
GA at T3	0.091	0.001	0.09	0.42	-0.047	0.001	0.33	0.94	0.017	0.001	0.75	0.99	-0.063	0.001	0.20	0.69
PMA at scan	0.547	0.001	<0.001	**<0.001**	-0.672	0.002	<0.001	**<0.001**	-0.564	0.002	<0.001	**<0.001**	-0.663	0.002	<0.001	**<0.001**
**Optic Radiation**																
TNF-α T3	-0.016	0.002	0.75	0.85	-0.020	0.003	0.69	0.95	-0.031	0.003	0.61	0.78	-0.010	0.003	0.84	0.96
Sex	-0.002	0.003	0.97	0.97	-0.113	0.005	0.02	0.11	-0.173	0.005	<0.01	**0.02**	-0.083	0.006	0.09	0.39
NICU stay	-0.086	0.009	0.16	0.55	0.075	0.013	0.20	0.92	0.027	0.014	0.70	0.78	0.084	0.015	0.14	0.68
GA at delivery	-0.015	0.001	0.83	0.97	-0.044	0.002	0.50	0.97	-0.058	0.002	0.45	0.58	-0.028	0.002	0.66	0.86
GA at T3	0.035	0.001	0.52	0.59	0.013	0.001	0.81	0.94	0.056	0.001	0.37	0.99	-0.004	0.001	0.94	0.97
PMA at scan	0.543	0.001	<0.001	**<0.001**	-0.563	0.002	<0.001	**<0.001**	-0.271	0.002	<0.001	**<0.001**	-0.604	0.002	<0.001	**<0.001**
**Uncinate Fasciculus**																
TNF-α T3	-0.091	0.001	0.09	0.44	-0.053	0.002	0.33	0.75	-0.129	0.003	0.03	0.27	-0.011	0.002	0.83	0.96
Sex	-0.070	0.003	0.18	0.41	-0.063	0.004	0.25	0.56	-0.119	0.005	0.04	0.13	-0.029	0.005	0.58	0.79
NICU stay	-0.042	0.007	0.49	0.55	-0.126	0.011	0.05	0.43	-0.168	0.014	0.02	0.14	-0.084	0.012	0.17	0.68

GA at delivery	0.119	0.001	0.08	0.40	0.038	0.002	0.59	0.97	0.120	0.002	0.12	0.58	-0.012	0.002	0.86	0.86
GA at T3	-0.013	0.000	0.82	0.82	0.002	0.001	0.98	0.98	0.001	0.001	0.99	0.99	0.002	0.001	0.97	0.97
PMA at scan	0.460	0.001	<0.001	**<0.001**	-0.510	0.002	<0.001	<0.001	-0.247	0.002	<0.001	**<0.001**	-0.562	0.002	<0.001	**<0.001**
**Inferior Fronto-Occipital Fasciculus**																
TNF-α T3	0.031	0.002	0.52	0.85	-0.039	0.002	0.42	0.75	-0.005	0.002	0.93	0.93	-0.041	0.003	0.38	0.86
Sex	-0.035	0.003	0.47	0.70	-0.081	0.005	0.09	0.28	-0.164	0.004	<0.01	**0.02**	-0.044	0.006	0.34	0.77
NICU stay	-0.053	0.008	0.34	0.55	-0.004	0.013	0.94	0.94	-0.047	0.010	0.48	0.78	0.015	0.015	0.78	0.94
GA at delivery	0.074	0.001	0.24	0.65	-0.027	0.002	0.67	0.97	0.059	0.002	0.43	0.58	-0.043	0.002	0.49	0.86
GA at T3	0.040	0.001	0.42	0.54	-0.011	0.001	0.83	0.94	0.031	0.001	0.60	0.99	-0.023	0.001	0.64	0.83
PMA at scan	0.598	0.001	<0.001	**<0.001**	-0.630	0.002	<0.001	**<0.001**	-0.400	0.002	<0.001	**<0.001**	-0.642	0.002	<0.001	**<0.001**
**Anterior Limb of Internal Capsule**																
TNF-α T3	0.009	0.001	0.85	0.85	-0.058	0.003	0.25	0.75	-0.070	0.003	0.22	0.49	-0.051	0.003	0.30	0.86
Sex	-0.048	0.003	0.31	0.55	-0.022	0.006	0.66	0.86	-0.049	0.005	0.38	0.38	-0.007	0.007	0.88	0.88
NICU stay	-0.027	0.007	0.62	0.62	-0.031	0.015	0.60	0.94	-0.074	0.013	0.27	0.78	-0.014	0.017	0.80	0.94
GA at delivery	0.104	0.001	0.09	0.40	-0.020	0.002	0.76	0.97	0.063	0.002	0.39	0.58	-0.042	0.003	0.50	0.86
GA at T3	0.058	0.000	0.24	0.51	-0.030	0.001	0.56	0.94	-0.001	0.001	0.98	0.99	-0.038	0.001	0.45	0.69
PMA at scan	0.596	0.001	<0.001	**<0.001**	-0.597	0.002	<0.001	**<0.001**	-0.454	0.002	<0.001	**<0.001**	-0.618	0.003	<0.001	**<0.001**
**Inferior Cingulum Bundle**																
TNF-α T3	-0.096	0.001	0.10	0.44	0.003	0.001	0.95	0.95	-0.061	0.002	0.30	0.55	0.029	0.002	0.57	0.96
Sex	0.029	0.002	0.62	0.79	-0.121	0.003	0.02	0.11	-0.085	0.004	0.15	0.19	-0.116	0.003	0.02	0.20
NICU stay	-0.131	0.006	0.05	0.48	0.010	0.007	0.87	0.94	-0.078	0.009	0.26	0.78	0.072	0.008	0.23	0.94
GA at delivery	-0.080	0.001	0.29	0.65	0.128	0.001	0.06	0.52	0.063	0.002	0.42	0.58	0.144	0.001	0.03	0.28
GA at T3	-0.065	0.000	0.29	0.51	0.016	0.001	0.77	0.94	-0.031	0.001	0.62	0.99	0.041	0.001	0.44	0.69
PMA at scan	0.382	0.001	<0.001	**<0.001**	-0.608	0.001	<0.001	**<0.001**	-0.338	0.001	<0.001	**<0.001**	-0.623	0.001	<0.001	**<0.001**
**Fornix**																
TNF-α T3	-0.012	0.001	0.83	0.85	-0.091	0.002	0.07	0.44	-0.105	0.002	0.06	0.27	-0.068	0.002	0.17	0.84
Sex	-0.112	0.002	0.05	0.25	-0.001	0.003	0.98	0.98	-0.056	0.004	0.31	0.35	0.034	0.003	0.49	0.79
NICU stay	-0.054	0.005	0.43	0.55	-0.012	0.008	0.83	0.94	-0.045	0.010	0.48	0.78	0.009	0.008	0.88	0.94
GA at delivery	0.013	0.001	0.86	0.97	0.022	0.001	0.73	0.97	0.036	0.002	0.62	0.69	0.015	0.001	0.81	0.86
GA at T3	-0.105	0.000	0.08	0.42	0.051	0.001	0.33	0.94	-0.015	0.001	0.80	0.99	0.079	0.001	0.13	0.69
PMA at scan	0.346	0.001	<0.001	**<0.001**	-0.623	0.001	<0.001	**<0.001**	-0.475	0.002	<0.001	**<0.001**	-0.632	0.001	<0.001	**<0.001**

Bolded values represent statistical significance after FDR correction for multiple comparisons. β, standardized beta coefficient; SE, standard error; *q*, FDR-corrected p-value; FA, fractional anisotropy; MD, mean diffusivity; AD, axial diffusivity; RD, radial diffusivity; IL, interleukin; TNF-α, tumor necrosis factor alpha; GA, gestational age; PMA, infant postmenstrual age; Sex, child sex; T, trimester.

We investigated the moderating role of family socioeconomic group in the relationship between maternal cytokine concentrations by trimester and neonatal dMRI parameters. As shown in Table S3, family SES moderated the relationship between maternal IL-6 at trimester 3 and CC FA (β = -0.14; *q* = 0.01). Additionally, family SES moderated the relationship between maternal TNF-α at trimester 1 and 1) CC FA (β = -0.21; *q* = 0.01), MD (β = 0.19; *q* = 0.03), and RD (β = 0.20; *q* = 0.03); 2) CB MD (β = 0.26; *q* = 0.01) and RD (β = 0.24; *q* = 0.03); 3) CST (β = 0.16; *q* = 0.04); 4) IFOF MD (β = 0.18; *q* = 0.03); ALIC MD (β = 0.24; *q* = 0.01), AD (β = 0.25; *q* = 0.04), and RD (β = 0.22; *q* = 0.02); and 4) CBIF MD (β = 0.21; *q* = 0.03). Lastly, family SES moderated the relationship between maternal TNF-α at trimester 3 and CB AD (β = 0.21; *q* = 0.02) and CBIF AD (β = 0.26; *q* < 0.01).

Table S5.

**IL-6 Trimester 1**																
	**FA**				**MD**				**AD**				**RD**			
	**β**	**SE**	***p***	***q***	**β**	**SE**	***p***	***q***	**β**	**SE**	***p***	***q***	**β**	**SE**	***p***	***q***
**Corpus Callosum**																
IL-6 T1	0.018	0.002	0.82	0.99	-0.033	0.002	0.69	0.88	-0.046	0.003	0.62	0.71	-0.038	0.003	0.63	0.81
SES group	0.128	0.004	0.05	0.22	-0.009	0.004	0.88	0.88	0.193	0.005	0.01	**0.02**	-0.082	0.005	0.19	0.21
Sex	-0.141	0.004	0.03	0.17	0.066	0.004	0.29	0.80	-0.088	0.004	0.22	0.40	0.113	0.005	0.06	0.56
NICU stay	0.017	0.009	0.82	0.94	-0.071	0.009	0.32	1.00	0.060	0.010	0.46	0.96	-0.080	0.012	0.25	0.88
GA at delivery	-0.048	0.002	0.54	0.61	0.046	0.002	0.55	0.80	-0.049	0.002	0.58	0.93	0.044	0.002	0.56	0.83
GA at T1	-0.074	0.001	0.24	0.70	0.094	0.001	0.13	0.95	0.026	0.001	0.72	0.78	0.096	0.001	0.11	0.85
PMA at scan	0.455	0.002	<0.001	**<0.001**	-0.540	0.001	<0.001	**<0.001**	-0.105	0.002	<0.001	**<0.001**	-0.534	0.002	<0.001	**<0.001**
IL-6 T1:SES group	-0.079	0.004	0.34	0.86	0.110	0.004	0.17	0.38	0.066	0.004	0.49	0.73	0.110	0.005	0.16	0.51
**Superior Cingulum Bundle**																
IL-6 T1	0.008	0.002	0.93	0.99	-0.074	0.003	0.37	0.78	-0.033	0.004	0.71	0.71	-0.083	0.004	0.31	0.81
SES group	-0.147	0.004	0.04	0.22	0.181	0.006	0.01	**0.01**	0.083	0.007	0.24	0.24	0.195	0.006	<0.01	**0.01**
Sex	-0.109	0.003	0.11	0.34	-0.033	0.005	0.60	0.85	-0.126	0.006	0.07	0.15	0.010	0.006	0.88	1.00
NICU stay	0.028	0.008	0.72	0.94	0.026	0.012	0.72	1.00	0.040	0.014	0.61	0.96	0.011	0.014	0.88	0.88
GA at delivery	0.148	0.001	0.08	0.28	-0.043	0.002	0.58	0.80	0.060	0.003	0.48	0.93	-0.091	0.002	0.25	0.83
GA at T1	-0.020	0.001	0.76	0.85	-0.022	0.001	0.73	0.95	-0.051	0.001	0.46	0.78	-0.010	0.001	0.87	0.97
PMA at scan	0.292	0.001	<0.001	**<0.001**	-0.490	0.002	<0.001	**<0.001**	-0.387	0.002	<0.001	**<0.001**	-0.465	0.002	<0.001	**<0.001**
IL-6 T1:SES group	-0.008	0.003	0.93	0.93	0.134	0.005	0.10	0.38	0.114	0.006	0.20	0.68	0.123	0.006	0.13	0.51
**Corticospinal Tract**																
IL-6 T1	0.022	0.002	0.79	0.99	-0.084	0.003	0.24	0.78	-0.095	0.003	0.21	0.71	-0.074	0.004	0.31	0.81
SES group	0.069	0.004	0.28	0.80	0.117	0.005	0.04	**0.04**	0.255	0.004	<0.001	**<0.001**	0.060	0.006	0.30	0.30
Sex	-0.081	0.003	0.20	0.37	0.020	0.005	0.71	0.85	-0.031	0.004	0.59	0.63	0.038	0.006	0.50	1.00
NICU stay	0.101	0.008	0.16	0.94	0.000	0.011	1.00	1.00	0.075	0.009	0.26	0.96	-0.026	0.013	0.69	0.88
GA at delivery	-0.030	0.001	0.70	0.70	0.015	0.002	0.83	0.83	-0.007	0.002	0.93	0.93	0.020	0.002	0.78	0.88
GA at T1	0.024	0.001	0.71	0.85	0.011	0.001	0.84	0.95	0.045	0.001	0.44	0.78	-0.002	0.001	0.97	0.97
PMA at scan	0.516	0.001	<0.001	**<0.001**	-0.675	0.002	<0.001	**<0.001**	-0.573	0.002	<0.001	**<0.001**	-0.657	0.002	<0.001	**<0.001**
IL-6 T1:SES group	-0.071	0.003	0.38	0.86	0.100	0.005	0.16	0.38	0.076	0.004	0.32	0.68	0.101	0.006	0.17	0.51
**Optic Radiation**																
IL-6 T1	-0.013	0.002	0.87	0.99	-0.056	0.004	0.47	0.78	-0.044	0.004	0.61	0.71	-0.038	0.004	0.62	0.81
SES group	0.056	0.004	0.37	0.80	0.181	0.006	<0.01	**0.01**	0.294	0.006	<0.001	**<0.001**	0.127	0.007	0.04	0.06

Sex	-0.078	0.004	0.21	0.37	-0.070	0.006	0.24	0.80	-0.175	0.006	0.01	0.06	-0.031	0.007	0.60	1.00
NICU stay	-0.087	0.008	0.22	0.94	0.035	0.014	0.62	1.00	-0.004	0.014	0.96	0.96	0.050	0.016	0.46	0.88
GA at delivery	-0.107	0.001	0.16	0.30	-0.037	0.002	0.62	0.80	-0.119	0.002	0.15	0.68	-0.002	0.003	0.98	0.98
GA at T1	-0.012	0.001	0.85	0.85	0.001	0.001	0.98	0.98	0.019	0.001	0.78	0.78	0.008	0.002	0.89	0.97
PMA at scan	0.553	0.001	<0.001	**<0.001**	-0.561	0.002	<0.001	**<0.001**	-0.303	0.002	<0.001	**<0.001**	-0.602	0.003	<0.001	**<0.001**
IL-6 T1:SES group	0.039	0.003	0.62	0.93	-0.015	0.006	0.85	0.85	-0.010	0.006	0.91	0.91	-0.027	0.007	0.73	0.97
**Uncinate Fasciculus**																
IL-6 T1	-0.076	0.002	0.35	0.99	-0.020	0.003	0.81	0.91	-0.089	0.004	0.33	0.71	0.010	0.003	0.89	0.89
SES group	-0.024	0.003	0.71	0.91	0.155	0.005	0.02	**0.03**	0.160	0.006	0.03	**0.03**	0.127	0.006	0.04	0.06
Sex	-0.129	0.003	0.04	0.17	-0.057	0.005	0.36	0.80	-0.159	0.006	0.02	0.07	-0.006	0.006	0.93	1.00
NICU stay	0.005	0.007	0.94	0.94	0.005	0.012	0.94	1.00	-0.009	0.014	0.91	0.96	0.012	0.013	0.86	0.88
GA at delivery	0.106	0.001	0.17	0.30	0.089	0.002	0.25	0.80	0.173	0.002	0.05	0.41	0.033	0.002	0.66	0.84
GA at T1	-0.107	0.001	0.08	0.70	0.024	0.001	0.70	0.98	-0.044	0.001	0.52	0.78	0.043	0.001	0.47	0.85
PMA at scan	0.451	0.001	<0.001	**<0.001**	-0.576	0.002	<0.001	**<0.001**	-0.318	0.002	<0.001	**<0.001**	-0.610	0.002	<0.001	**<0.001**
IL-6 T1:SES group	-0.011	0.003	0.89	0.93	0.034	0.005	0.67	0.85	0.041	0.006	0.65	0.79	0.028	0.005	0.72	0.97
**Inferior Fronto-Occipital Fasciculus**																
IL-6 T1	0.014	0.002	0.85	0.99	-0.046	0.003	0.52	0.78	-0.062	0.003	0.46	0.71	-0.036	0.004	0.61	0.81
SES group	0.000	0.004	1.00	1.00	0.165	0.006	<0.01	**0.01**	0.307	0.004	<0.001	**<0.001**	0.118	0.007	0.04	0.06
Sex	-0.028	0.004	0.62	0.70	-0.083	0.005	0.13	0.80	-0.158	0.004	0.01	0.06	-0.049	0.007	0.37	1.00
NICU stay	-0.008	0.008	0.90	0.94	0.028	0.012	0.66	1.00	0.043	0.010	0.56	0.96	0.021	0.016	0.73	0.88
GA at delivery	0.061	0.001	0.39	0.50	-0.053	0.002	0.43	0.80	-0.010	0.002	0.90	0.93	-0.059	0.003	0.38	0.83
GA at T1	-0.059	0.001	0.30	0.70	0.023	0.001	0.67	0.95	-0.022	0.001	0.72	0.78	0.043	0.002	0.42	0.85
PMA at scan	0.604	0.001	<0.001	**<0.001**	-0.653	0.002	<0.001	**<0.001**	-0.442	0.002	<0.001	**<0.001**	-0.657	0.003	<0.001	**<0.001**
IL-6 T1:SES group	0.039	0.004	0.60	0.93	0.019	0.005	0.79	0.85	0.087	0.004	0.29	0.68	0.000	0.007	1.00	1.00
**Anterior Limb of Internal Capsule**																
IL-6 T1	-0.001	0.002	0.99	0.99	-0.071	0.004	0.36	0.78	-0.090	0.004	0.30	0.71	-0.061	0.005	0.42	0.81
SES group	0.001	0.003	0.98	1.00	0.132	0.007	0.03	**0.04**	0.189	0.006	0.01	**0.01**	0.105	0.008	0.08	0.10
Sex	-0.041	0.003	0.46	0.59	-0.011	0.007	0.85	0.85	-0.034	0.006	0.61	0.63	0.000	0.008	1.00	1.00
NICU stay	0.018	0.007	0.79	0.94	0.033	0.015	0.63	1.00	0.055	0.013	0.47	0.96	0.024	0.017	0.71	0.88
GA at delivery	0.118	0.001	0.09	0.28	-0.042	0.003	0.57	0.80	0.024	0.002	0.77	0.93	-0.061	0.003	0.39	0.83
GA at T1	-0.041	0.001	0.47	0.70	0.040	0.002	0.50	0.95	0.026	0.001	0.70	0.78	0.042	0.002	0.46	0.85
PMA at scan	0.588	0.001	<0.001	**<0.001**	-0.576	0.003	<0.001	**<0.001**	-0.404	0.002	<0.001	**<0.001**	-0.604	0.003	<0.001	**<0.001**
IL-6 T1:SES group	0.072	0.003	0.33	0.86	0.021	0.007	0.79	0.85	0.077	0.006	0.38	0.68	0.001	0.007	0.99	1.00
**Inferior Cingulum Bundle**																
IL-6 T1	-0.045	0.002	0.61	0.99	-0.002	0.002	0.98	0.98	-0.034	0.003	0.71	0.71	0.019	0.002	0.81	0.89
SES group	-0.039	0.003	0.58	0.86	0.214	0.003	<0.01	**0.01**	0.179	0.004	0.01	**0.02**	0.192	0.004	<0.01	**0.01**
Sex	-0.008	0.003	0.90	0.90	-0.047	0.003	0.44	0.80	-0.047	0.004	0.50	0.63	-0.036	0.003	0.55	1.00
NICU stay	0.056	0.006	0.47	0.94	-0.026	0.007	0.71	1.00	0.005	0.010	0.95	0.96	-0.037	0.008	0.59	0.88
GA at delivery	-0.160	0.001	0.06	0.28	0.067	0.001	0.38	0.80	-0.048	0.002	0.58	0.93	0.115	0.001	0.12	0.83
GA at T1	-0.069	0.001	0.31	0.70	-0.017	0.001	0.79	0.95	-0.054	0.001	0.43	0.78	0.010	0.001	0.86	0.97
PMA at scan	0.415	0.001	<0.001	**<0.001**	-0.594	0.001	<0.001	**<0.001**	-0.317	0.002	<0.001	**<0.001**	-0.631	0.001	<0.001	**<0.001**
IL-6 T1:SES group	-0.014	0.002	0.87	0.93	0.033	0.003	0.68	0.85	0.034	0.004	0.70	0.79	0.024	0.003	0.75	0.97
**Fornix**																

IL-6 T1	-0.039	0.001	0.67	0.99	-0.147	0.002	0.06	0.50	-0.170	0.003	0.04	0.40	-0.115	0.002	0.13	0.81
SES group	-0.055	0.002	0.44	0.80	0.137	0.004	0.02	**0.03**	0.132	0.005	0.05	0.05	0.129	0.004	0.03	0.06
Sex	-0.068	0.002	0.33	0.50	-0.012	0.003	0.84	0.85	-0.031	0.005	0.63	0.63	0.006	0.003	0.91	1.00
NICU stay	0.019	0.005	0.81	0.94	0.017	0.008	0.80	1.00	0.022	0.010	0.77	0.96	0.012	0.008	0.86	0.88
GA at delivery	-0.087	0.001	0.32	0.47	0.018	0.001	0.81	0.83	-0.036	0.002	0.65	0.93	0.050	0.001	0.49	0.83
GA at T1	-0.057	0.000	0.41	0.70	0.064	0.001	0.27	0.95	0.058	0.001	0.36	0.78	0.067	0.001	0.25	0.85
PMA at scan	0.356	0.001	<0.001	**<0.001**	-0.606	0.001	<0.001	**<0.001**	-0.453	0.002	<0.001	**<0.001**	-0.628	0.001	<0.001	**<0.001**
IL-6 T1:SES group	0.092	0.002	0.31	0.86	0.119	0.003	0.12	0.38	0.181	0.004	0.03	0.28	0.070	0.003	0.36	0.80

IL-6 Trimester 2

	FA				MD				AD				RD			
	**β**	**SE**	***p***	***q***	**β**	**SE**	***p***	***q***	**β**	**SE**	***p***	***q***	**β**	**SE**	***p***	***q***
**Corpus Callosum**																
IL-6 T2	-0.008	0.002	0.91	1.00	-0.002	0.002	0.98	0.99	-0.043	0.003	0.61	0.69	0.007	0.003	0.92	0.98
SES group	-0.041	0.004	0.53	0.70	0.026	0.004	0.69	0.69	0.041	0.005	0.60	0.81	0.030	0.006	0.63	0.71
Sex	-0.071	0.004	0.24	0.79	-0.027	0.004	0.65	0.73	-0.090	0.004	0.20	0.33	0.024	0.005	0.68	0.89
NICU stay	-0.067	0.009	0.36	0.75	-0.036	0.009	0.63	0.91	0.033	0.010	0.72	0.96	-0.015	0.013	0.83	0.94
GA at delivery	0.066	0.001	0.41	0.59	-0.055	0.001	0.50	0.75	0.145	0.001	0.14	0.42	-0.096	0.002	0.23	0.68
GA at T2	0.038	0.001	0.54	0.86	0.006	0.001	0.92	0.95	0.057	0.001	0.44	0.65	0.001	0.001	0.99	0.99
PMA at scan	0.458	0.001	<0.001	**<0.001**	-0.564	0.001	<0.001	**<0.001**	-0.223	0.002	<0.001	**<0.001**	-0.548	0.002	<0.001	**<0.001**
IL-6 T2:SES group	-0.207	0.004	0.01	0.06	0.106	0.004	0.15	0.71	-0.092	0.005	0.30	0.90	0.151	0.006	0.04	0.33
**Superior Cingulum Bundle**																
IL-6 T2	0.073	0.002	0.36	0.59	-0.115	0.003	0.13	0.58	-0.046	0.004	0.58	0.69	-0.127	0.004	0.09	0.41
SES group	-0.185	0.004	0.01	0.05	0.114	0.006	0.09	0.21	-0.017	0.007	0.81	0.81	0.157	0.007	0.02	0.09
Sex	-0.065	0.003	0.32	0.79	-0.012	0.005	0.84	0.84	-0.077	0.006	0.26	0.33	0.016	0.006	0.79	0.89
NICU stay	0.030	0.008	0.72	0.81	0.058	0.012	0.46	0.91	0.070	0.014	0.41	0.96	0.039	0.014	0.62	0.94
GA at delivery	0.067	0.001	0.46	0.59	0.094	0.002	0.27	0.75	0.140	0.002	0.13	0.42	0.055	0.002	0.51	0.75
GA at T2	-0.014	0.000	0.84	0.86	0.017	0.001	0.79	0.95	0.029	0.001	0.67	0.67	0.008	0.001	0.90	0.99
PMA at scan	0.396	0.001	<0.001	**<0.001**	-0.537	0.002	<0.001	**<0.001**	-0.394	0.002	<0.001	**<0.001**	-0.528	0.002	<0.001	**<0.001**
IL-6 T2:SES group	-0.002	0.004	0.98	0.98	0.060	0.006	0.44	0.71	0.038	0.007	0.65	0.90	0.060	0.007	0.44	0.90
**Corticospinal Tract**																
IL-6 T2	-0.231	0.002	<0.01	**0.01**	0.153	0.003	0.01	0.12	0.025	0.002	0.71	0.71	0.170	0.004	0.01	0.07
SES group	-0.052	0.004	0.40	0.70	0.124	0.006	0.03	0.08	0.160	0.005	0.01	**0.04**	0.104	0.007	0.07	0.14
Sex	0.041	0.003	0.47	0.79	-0.046	0.005	0.37	0.71	-0.035	0.004	0.52	0.52	-0.045	0.006	0.39	0.88
NICU stay	-0.028	0.008	0.70	0.81	0.000	0.012	1.00	1.00	0.022	0.009	0.75	0.96	0.002	0.014	0.98	0.98
GA at delivery	0.050	0.001	0.52	0.59	-0.054	0.002	0.44	0.75	-0.004	0.001	0.96	0.96	-0.038	0.002	0.58	0.75
GA at T2	0.038	0.000	0.52	0.86	0.003	0.001	0.95	0.95	0.027	0.001	0.64	0.67	-0.012	0.001	0.82	0.99
PMA at scan	0.541	0.001	<0.001	**<0.001**	-0.693	0.002	<0.001	**<0.001**	-0.651	0.001	<0.001	**<0.001**	-0.672	0.002	<0.001	**<0.001**
IL-6 T2:SES group	0.055	0.004	0.44	0.98	-0.047	0.005	0.46	0.71	-0.028	0.004	0.69	0.90	-0.043	0.006	0.51	0.90
**Optic Radiation**																
IL-6 T2	-0.171	0.002	0.01	0.06	0.051	0.003	0.42	0.95	-0.066	0.003	0.39	0.69	0.086	0.004	0.16	0.49
SES group	0.014	0.004	0.82	0.86	0.136	0.006	0.02	0.08	0.206	0.007	<0.01	**0.03**	0.099	0.007	0.07	0.14
Sex	-0.004	0.004	0.94	0.99	-0.147	0.006	0.01	**0.03**	-0.222	0.006	<0.01	**0.01**	-0.110	0.006	0.03	0.14
NICU stay	-0.096	0.008	0.18	0.75	0.076	0.013	0.25	0.91	0.026	0.013	0.75	0.96	0.088	0.015	0.17	0.77

GA at delivery	0.030	0.001	0.69	0.69	-0.113	0.002	0.12	0.75	-0.101	0.002	0.25	0.51	-0.101	0.002	0.15	0.68
GA at T2	0.046	0.001	0.43	0.86	0.039	0.001	0.47	0.95	0.071	0.001	0.29	0.52	0.021	0.001	0.69	0.99
PMA at scan	0.548	0.001	<0.001	**<0.001**	-0.615	0.002	<0.001	**<0.001**	-0.351	0.002	<0.001	**<0.001**	-0.641	0.002	<0.001	**<0.001**
IL-6 T2:SES group	0.037	0.004	0.60	0.98	-0.022	0.006	0.73	0.92	-0.010	0.006	0.90	0.90	-0.024	0.007	0.70	0.90
**Uncinate Fasciculus**																
IL-6 T2	-0.138	0.002	0.05	0.14	-0.023	0.003	0.76	0.98	-0.117	0.003	0.16	0.69	0.013	0.003	0.86	0.98
SES group	-0.079	0.003	0.21	0.46	0.068	0.006	0.30	0.45	0.018	0.007	0.81	0.81	0.077	0.006	0.21	0.29
Sex	-0.050	0.003	0.38	0.79	-0.036	0.005	0.55	0.71	-0.085	0.006	0.22	0.33	-0.015	0.005	0.79	0.89
NICU stay	-0.012	0.007	0.87	0.87	0.019	0.011	0.81	0.91	0.004	0.013	0.96	0.96	0.024	0.012	0.73	0.94
GA at delivery	0.121	0.001	0.12	0.46	0.043	0.002	0.60	0.75	0.148	0.002	0.11	0.42	-0.012	0.002	0.88	0.95
GA at T2	0.010	0.000	0.86	0.86	0.077	0.001	0.22	0.95	0.099	0.001	0.16	0.46	0.053	0.001	0.37	0.99
PMA at scan	0.537	0.001	<0.001	**<0.001**	-0.570	0.002	<0.001	**<0.001**	-0.257	0.002	<0.001	**<0.001**	-0.625	0.002	<0.001	**<0.001**
IL-6 T2:SES group	-0.008	0.003	0.91	0.98	-0.059	0.005	0.44	0.71	-0.089	0.006	0.30	0.90	-0.033	0.006	0.64	0.90
**Inferior Fronto-Occipital Fasciculus**																
IL-6 T2	-0.054	0.002	0.39	0.59	-0.026	0.003	0.68	0.98	-0.102	0.003	0.19	0.69	-0.001	0.004	0.98	0.98
SES group	-0.034	0.004	0.54	0.70	0.077	0.007	0.19	0.34	0.115	0.005	0.10	0.30	0.068	0.008	0.23	0.29
Sex	0.014	0.004	0.78	0.99	-0.102	0.006	0.06	0.17	-0.168	0.004	0.01	**0.03**	-0.073	0.007	0.16	0.49
NICU stay	-0.047	0.008	0.47	0.75	0.065	0.013	0.34	0.91	0.051	0.010	0.54	0.96	0.063	0.016	0.34	0.77
GA at delivery	0.089	0.001	0.21	0.46	-0.024	0.002	0.74	0.75	0.081	0.002	0.36	0.51	-0.048	0.002	0.50	0.75
GA at T2	0.043	0.001	0.43	0.86	0.017	0.001	0.75	0.95	0.095	0.001	0.15	0.46	-0.003	0.001	0.96	0.99
PMA at scan	0.631	0.001	<0.001	**<0.001**	-0.658	0.002	<0.001	**<0.001**	-0.449	0.002	<0.001	**<0.001**	-0.664	0.003	<0.001	**<0.001**
IL-6 T2:SES group	-0.065	0.004	0.31	0.98	0.048	0.006	0.47	0.71	0.014	0.005	0.86	0.90	0.055	0.008	0.40	0.90
**Anterior Limb of Internal Capsule**																
IL-6 T2	-0.031	0.002	0.62	0.79	-0.036	0.004	0.60	0.98	-0.073	0.003	0.35	0.69	-0.023	0.005	0.73	0.98
SES group	0.010	0.003	0.86	0.86	0.028	0.008	0.65	0.69	0.046	0.007	0.52	0.81	0.020	0.009	0.74	0.74
Sex	0.001	0.003	0.99	0.99	-0.035	0.007	0.54	0.71	-0.046	0.006	0.48	0.52	-0.029	0.008	0.60	0.89
NICU stay	-0.061	0.007	0.35	0.75	0.065	0.016	0.37	0.91	0.047	0.013	0.57	0.96	0.068	0.018	0.33	0.77
GA at delivery	0.143	0.001	0.04	0.38	-0.024	0.002	0.75	0.75	0.074	0.002	0.41	0.51	-0.055	0.003	0.47	0.75
GA at T2	-0.013	0.000	0.80	0.86	0.062	0.001	0.30	0.95	0.097	0.001	0.15	0.46	0.049	0.001	0.39	0.99
PMA at scan	0.614	0.001	<0.001	**<0.001**	-0.601	0.002	<0.001	**<0.001**	-0.452	0.002	<0.001	**<0.001**	-0.623	0.003	<0.001	**<0.001**
IL-6 T2:SES group	-0.004	0.003	0.95	0.98	-0.012	0.007	0.86	0.92	-0.020	0.006	0.81	0.90	-0.010	0.008	0.89	0.97
**Inferior Cingulum Bundle**																
IL-6 T2	-0.083	0.002	0.29	0.59	0.001	0.002	0.99	0.99	-0.058	0.002	0.47	0.69	0.037	0.002	0.58	0.98
SES group	-0.136	0.003	0.05	0.16	0.197	0.003	<0.01	**0.01**	0.107	0.005	0.14	0.31	0.178	0.004	<0.01	**0.02**
Sex	-0.041	0.003	0.53	0.79	-0.178	0.003	<0.01	**0.01**	-0.198	0.004	<0.01	**0.01**	-0.144	0.003	0.01	0.08
NICU stay	-0.084	0.006	0.31	0.75	0.031	0.007	0.65	0.91	-0.047	0.009	0.58	0.96	0.072	0.008	0.30	0.77
GA at delivery	-0.125	0.001	0.16	0.46	0.047	0.001	0.53	0.75	-0.069	0.001	0.45	0.51	0.105	0.001	0.16	0.68
GA at T2	0.040	0.000	0.55	0.86	0.055	0.000	0.34	0.95	0.087	0.001	0.20	0.46	0.045	0.000	0.42	0.99
PMA at scan	0.460	0.001	<0.001	**<0.001**	-0.650	0.001	<0.001	**<0.001**	-0.334	0.001	<0.001	**<0.001**	-0.683	0.001	<0.001	**<0.001**
IL-6 T2:SES group	-0.011	0.003	0.89	0.98	-0.050	0.003	0.46	0.71	-0.056	0.004	0.50	0.90	-0.065	0.004	0.33	0.90
**Fornix**																
IL-6 T2	0.000	0.001	1.00	1.00	-0.063	0.002	0.36	0.95	-0.069	0.003	0.37	0.69	-0.050	0.002	0.45	0.98
SES group	-0.190	0.002	0.01	0.05	0.057	0.004	0.35	0.45	-0.024	0.005	0.72	0.81	0.105	0.004	0.08	0.14

Sex	-0.098	0.002	0.13	0.79	-0.037	0.003	0.51	0.71	-0.080	0.005	0.21	0.33	-0.003	0.003	0.96	0.96
NICU stay	0.056	0.005	0.50	0.75	-0.018	0.008	0.80	0.91	0.007	0.011	0.93	0.96	-0.031	0.008	0.66	0.94
GA at delivery	0.089	0.001	0.32	0.57	0.028	0.001	0.71	0.75	0.084	0.002	0.34	0.51	-0.005	0.001	0.95	0.95
GA at T2	0.018	0.000	0.79	0.86	-0.035	0.000	0.55	0.95	-0.033	0.001	0.61	0.67	-0.032	0.000	0.58	0.99
PMA at scan	0.392	0.001	<0.001	**<0.001**	-0.654	0.001	<0.001	**<0.001**	-0.495	0.002	<0.001	**<0.001**	-0.665	0.001	<0.001	**<0.001**
IL-6 T2:SES group	0.007	0.002	0.93	0.98	0.007	0.004	0.92	0.92	0.031	0.005	0.70	0.90	-0.003	0.004	0.97	0.97

IL-6 Trimester 3

	FA				MD				AD				RD			
	**β**	**SE**	***p***	***q***	**β**	**SE**	***p***	***q***	**β**	**SE**	***p***	***q***	**β**	**SE**	***p***	***q***
**Corpus Callosum**																
IL-6 T3	0.063	0.002	0.35	0.53	-0.164	0.002	0.01	0.06	-0.116	0.003	0.14	0.16	-0.126	0.003	0.05	0.19
SES group	0.103	0.004	0.08	0.25	-0.026	0.004	0.64	0.64	0.137	0.004	0.04	0.06	-0.060	0.005	0.27	0.27
Sex	-0.121	0.003	0.03	0.05	0.003	0.003	0.96	0.96	-0.079	0.004	0.21	0.37	0.063	0.005	0.22	0.65
NICU stay	-0.056	0.009	0.36	0.72	-0.029	0.009	0.64	0.82	-0.015	0.010	0.84	0.84	-0.003	0.012	0.95	0.95
GA at delivery	-0.019	0.001	0.78	0.17	-0.006	0.001	0.93	0.93	0.036	0.002	0.66	0.99	-0.016	0.002	0.81	0.99
GA at T3	0.006	0.001	0.92	0.68	-0.006	0.001	0.92	0.95	-0.015	0.001	0.83	0.93	-0.016	0.001	0.77	0.93
PMA at scan	0.463	0.001	<0.001	**<0.001**	-0.580	0.001	<0.001	**<0.001**	-0.201	0.002	<0.001	**<0.001**	-0.567	0.002	<0.001	**<0.001**
IL-6 T3:SES group	-0.137	0.004	0.04	**0.01**	0.157	0.004	0.01	0.11	0.012	0.004	0.88	0.97	0.156	0.006	0.01	0.11
**Superior Cingulum Bundle**																
IL-6 T3	-0.026	0.002	0.73	0.73	-0.103	0.003	0.13	0.19	-0.152	0.004	0.03	**0.04**	-0.063	0.004	0.36	0.54
SES group	-0.130	0.003	0.04	0.19	0.143	0.005	0.02	**0.03**	0.050	0.006	0.42	0.42	0.163	0.006	0.01	**0.02**
Sex	-0.095	0.003	0.11	0.33	-0.012	0.005	0.82	0.96	-0.097	0.005	0.09	0.26	0.027	0.005	0.62	0.80
NICU stay	-0.049	0.008	0.48	0.72	-0.013	0.012	0.83	0.94	-0.057	0.014	0.40	0.52	0.006	0.014	0.92	0.95
GA at delivery	0.059	0.001	0.46	0.72	0.027	0.002	0.71	0.93	0.061	0.002	0.43	0.97	0.004	0.002	0.95	0.99
GA at T3	-0.047	0.001	0.47	0.88	0.004	0.001	0.95	0.95	-0.027	0.001	0.67	0.93	0.016	0.001	0.78	0.93
PMA at scan	0.324	0.001	<0.001	**<0.001**	-0.529	0.002	<0.001	**<0.001**	-0.413	0.002	<0.001	**<0.001**	-0.504	0.002	<0.001	**<0.001**
IL-6 T3:SES group	0.103	0.004	0.15	0.42	0.066	0.006	0.32	0.48	0.162	0.006	0.02	0.20	0.009	0.006	0.89	0.89
**Corticospinal Tract**																
IL-6 T3	-0.123	0.002	0.05	0.23	-0.015	0.003	0.78	0.78	-0.146	0.002	0.02	0.03	0.028	0.004	0.63	0.81
SES group	0.008	0.003	0.89	0.89	0.135	0.005	0.01	**0.02**	0.245	0.004	<0.001	**<0.001**	0.090	0.006	0.07	0.11
Sex	0.006	0.003	0.91	0.91	-0.039	0.004	0.39	0.87	-0.051	0.004	0.30	0.45	-0.029	0.005	0.53	0.80
NICU stay	-0.064	0.008	0.28	0.72	-0.001	0.011	0.98	0.98	-0.079	0.009	0.18	0.32	0.015	0.014	0.78	0.95
GA at delivery	-0.023	0.001	0.73	0.78	-0.046	0.002	0.44	0.80	-0.097	0.001	0.14	0.65	0.000	0.002	0.99	0.99
GA at T3	0.082	0.001	0.14	0.88	-0.065	0.001	0.18	0.95	-0.026	0.001	0.63	0.93	-0.073	0.001	0.15	0.93
PMA at scan	0.532	0.001	<0.001	**<0.001**	-0.669	0.002	<0.001	**<0.001**	-0.576	0.001	<0.001	**<0.001**	-0.656	0.002	<0.001	**<0.001**
IL-6 T3:SES group	-0.073	0.004	0.24	0.42	0.076	0.005	0.17	0.37	0.045	0.004	0.45	0.58	0.083	0.006	0.14	0.36
**Optic Radiation**																
IL-6 T3	-0.050	0.002	0.45	0.58	-0.025	0.004	0.68	0.77	-0.061	0.004	0.38	0.38	-0.004	0.004	0.95	0.95
SES group	0.083	0.004	0.14	0.32	0.183	0.006	<0.01	**<0.01**	0.321	0.006	<0.001	**<0.001**	0.116	0.007	0.03	0.05
Sex	0.013	0.003	0.80	0.90	-0.102	0.005	0.04	0.27	-0.149	0.005	0.01	0.08	-0.078	0.006	0.11	0.47
NICU stay	-0.101	0.009	0.10	0.47	0.040	0.013	0.49	0.82	-0.034	0.013	0.60	0.68	0.062	0.015	0.28	0.95
GA at delivery	-0.041	0.001	0.56	0.72	-0.109	0.002	0.10	0.80	-0.173	0.002	0.02	0.20	-0.068	0.002	0.29	0.65
GA at T3	0.004	0.001	0.94	0.94	-0.008	0.001	0.88	0.95	0.003	0.001	0.96	0.96	-0.011	0.001	0.83	0.93

PMA at scan	0.538	0.001	<0.001	**<0.001**	-0.561	0.002	<0.001	**<0.001**	-0.274	0.002	<0.001	**<0.001**	-0.600	0.002	<0.001	**<0.001**
IL-6 T3:SES group	-0.020	0.004	0.75	0.75	0.019	0.006	0.75	0.75	0.002	0.006	0.97	0.97	0.022	0.007	0.71	0.79
**Uncinate Fasciculus**																
IL-6 T3	-0.090	0.002	0.17	0.50	-0.103	0.003	0.12	0.19	-0.178	0.004	0.01	**0.02**	-0.059	0.003	0.36	0.54
SES group	-0.048	0.003	0.40	0.60	0.140	0.005	0.01	**0.03**	0.117	0.006	0.06	0.08	0.127	0.005	0.02	0.05
Sex	-0.073	0.003	0.16	0.36	-0.031	0.004	0.55	0.96	-0.090	0.005	0.12	0.26	-0.002	0.005	0.97	0.97
NICU stay	-0.032	0.007	0.61	0.72	-0.155	0.011	0.01	0.12	-0.194	0.013	<0.01	**0.04**	-0.110	0.012	0.07	0.62
GA at delivery	0.144	0.001	0.04	0.37	-0.014	0.002	0.85	0.93	0.083	0.002	0.28	0.83	-0.062	0.002	0.36	0.65
GA at T3	-0.027	0.001	0.64	0.88	-0.031	0.001	0.59	0.95	-0.044	0.001	0.48	0.93	-0.018	0.001	0.75	0.93
PMA at scan	0.455	0.001	<0.001	**<0.001**	-0.540	0.002	<0.001	**<0.001**	-0.280	0.002	<0.001	**<0.001**	-0.583	0.002	<0.001	**<0.001**
IL-6 T3:SES group	-0.030	0.003	0.64	0.72	-0.081	0.005	0.21	0.37	-0.110	0.006	0.12	0.34	-0.052	0.006	0.41	0.73
**Inferior Fronto-Occipital Fasciculus**																
IL-6 T3	0.025	0.002	0.68	0.73	-0.137	0.003	0.02	0.06	-0.188	0.003	0.01	**0.02**	-0.108	0.004	0.06	0.19
SES group	0.013	0.004	0.81	0.89	0.120	0.005	0.02	**0.03**	0.233	0.004	<0.001	**<0.001**	0.083	0.007	0.10	0.11
Sex	-0.020	0.003	0.68	0.87	-0.069	0.005	0.14	0.43	-0.127	0.004	0.02	0.10	-0.040	0.006	0.38	0.80
NICU stay	-0.047	0.008	0.41	0.72	-0.031	0.013	0.57	0.82	-0.088	0.010	0.18	0.32	-0.007	0.016	0.90	0.95
GA at delivery	0.081	0.001	0.21	0.62	-0.074	0.002	0.25	0.80	-0.017	0.002	0.82	0.99	-0.079	0.003	0.21	0.65
GA at T3	0.021	0.001	0.69	0.88	-0.026	0.001	0.61	0.95	-0.018	0.001	0.76	0.93	-0.028	0.001	0.58	0.93
PMA at scan	0.599	0.001	<0.001	**<0.001**	-0.640	0.002	<0.001	**<0.001**	-0.419	0.002	<0.001	**<0.001**	-0.649	0.002	<0.001	**<0.001**
IL-6 T3:SES group	-0.053	0.004	0.36	0.46	0.086	0.006	0.14	0.37	0.088	0.005	0.20	0.34	0.080	0.007	0.16	0.36
**Anterior Limb of Internal Capsule**																
IL-6 T3	0.061	0.002	0.30	0.53	-0.145	0.004	0.02	0.06	-0.177	0.003	0.01	**0.02**	-0.131	0.005	0.03	0.19
SES group	0.011	0.003	0.82	0.89	0.113	0.007	0.04	**0.04**	0.164	0.006	0.01	**0.01**	0.088	0.007	0.09	0.11
Sex	-0.044	0.003	0.35	0.63	-0.007	0.006	0.89	0.96	-0.025	0.005	0.65	0.65	0.004	0.007	0.94	0.97
NICU stay	-0.026	0.007	0.64	0.72	-0.056	0.015	0.34	0.82	-0.108	0.013	0.10	0.30	-0.035	0.017	0.54	0.95
GA at delivery	0.110	0.001	0.08	0.37	-0.061	0.002	0.36	0.80	0.008	0.002	0.91	0.99	-0.076	0.003	0.24	0.65
GA at T3	0.038	0.001	0.46	0.88	-0.049	0.001	0.37	0.95	-0.040	0.001	0.51	0.93	-0.048	0.001	0.36	0.93
PMA at scan	0.600	0.001	<0.001	**<0.001**	-0.604	0.002	<0.001	**<0.001**	-0.465	0.002	<0.001	**<0.001**	-0.625	0.003	<0.001	**<0.001**
IL-6 T3:SES group	-0.068	0.003	0.24	0.42	0.084	0.007	0.17	0.37	0.082	0.006	0.23	0.34	0.082	0.008	0.16	0.36
**Inferior Cingulum Bundle**																
IL-6 T3	-0.194	0.002	0.01	0.07	-0.083	0.002	0.18	0.24	-0.221	0.003	<0.01	**0.02**	0.013	0.002	0.83	0.94
SES group	-0.062	0.003	0.32	0.58	0.209	0.003	<0.001	**<0.01**	0.167	0.004	0.01	**0.01**	0.168	0.003	<0.01	**0.02**
Sex	0.041	0.002	0.48	0.72	-0.094	0.003	0.06	0.27	-0.048	0.004	0.41	0.46	-0.100	0.003	0.05	0.42
NICU stay	-0.118	0.006	0.08	0.47	-0.032	0.007	0.59	0.82	-0.113	0.009	0.10	0.30	0.039	0.008	0.51	0.95
GA at delivery	-0.073	0.001	0.35	0.72	0.059	0.001	0.38	0.80	-0.005	0.002	0.95	0.99	0.093	0.001	0.17	0.65
GA at T3	-0.046	0.000	0.47	0.88	-0.030	0.001	0.59	0.95	-0.063	0.001	0.32	0.93	0.001	0.001	0.98	0.98
PMA at scan	0.371	0.001	<0.001	**<0.001**	-0.615	0.001	<0.001	**<0.001**	-0.349	0.001	<0.001	**<0.001**	-0.625	0.001	<0.001	**<0.001**
IL-6 T3:SES group	0.070	0.003	0.32	0.46	0.039	0.003	0.52	0.67	0.095	0.004	0.18	0.34	-0.026	0.004	0.67	0.79
**Fornix**																
IL-6 T3	-0.075	0.001	0.30	0.53	-0.132	0.002	0.04	0.08	-0.181	0.003	0.01	**0.02**	-0.082	0.002	0.18	0.41
SES group	-0.150	0.002	0.02	0.16	0.116	0.003	0.03	**0.04**	0.052	0.005	0.39	0.42	0.144	0.003	0.01	**0.02**
Sex	-0.106	0.002	0.07	0.30	0.005	0.003	0.93	0.96	-0.048	0.004	0.39	0.46	0.037	0.003	0.45	0.80
NICU stay	-0.022	0.005	0.75	0.75	-0.048	0.008	0.41	0.82	-0.069	0.011	0.30	0.45	-0.031	0.008	0.59	0.95

GA at delivery	0.045	0.001	0.56	0.72	-0.024	0.001	0.72	0.93	0.001	0.002	0.99	0.99	-0.033	0.001	0.62	0.93
GA at T3	-0.032	0.000	0.62	0.88	0.004	0.001	0.95	0.95	-0.027	0.001	0.66	0.93	0.016	0.001	0.77	0.93
PMA at scan	0.343	0.001	<0.001	**<0.001**	-0.622	0.001	<0.001	**<0.001**	-0.474	0.002	<0.001	**<0.001**	-0.631	0.001	<0.001	**<0.001**
IL-6 T3:SES group	0.127	0.002	0.07	0.33	0.022	0.004	0.72	0.75	0.105	0.005	0.12	0.34	-0.028	0.004	0.64	0.79

IL-8 Trimester 1

	FA				MD				AD				RD			
	β	SE	*p*	*q*	β	SE	*p*	*q*	β	SE	*p*	*q*	β	SE	*p*	*q*
**Corpus Callosum**																
IL-8 T1	-0.002	0.003	0.99	0.99	0.134	0.003	0.10	0.48	0.099	0.004	0.30	0.75	0.097	0.004	0.23	0.73
SES group	0.139	0.004	0.03	0.21	-0.003	0.004	0.97	0.97	0.218	0.005	<0.01	**0.01**	-0.084	0.005	0.18	0.21
Sex	-0.146	0.004	0.02	0.16	0.051	0.004	0.41	0.70	-0.111	0.005	0.13	0.23	0.106	0.005	0.08	0.76
NICU stay	0.013	0.009	0.86	0.90	-0.089	0.009	0.22	0.98	0.041	0.010	0.61	0.91	-0.092	0.012	0.20	0.99
GA at delivery	-0.055	0.002	0.49	0.63	0.054	0.002	0.49	0.79	-0.030	0.002	0.73	0.95	0.050	0.002	0.52	0.76
GA at T1	-0.077	0.001	0.23	0.50	0.089	0.001	0.15	0.77	0.016	0.001	0.83	0.83	0.097	0.001	0.12	0.62
PMA at scan	0.455	0.002	<0.001	**<0.001**	-0.531	0.002	<0.001	**<0.001**	-0.116	0.002	0.16	0.16	-0.527	0.002	<0.001	**<0.001**
IL-8 T1:SES group	-0.005	0.005	0.95	0.95	-0.098	0.005	0.23	0.54	-0.113	0.006	0.24	0.71	-0.055	0.006	0.49	0.92
**Superior Cingulum Bundle**																
IL-8 T1	-0.024	0.003	0.79	0.99	0.034	0.004	0.69	0.88	0.022	0.005	0.81	0.91	0.036	0.005	0.67	0.86
SES group	-0.141	0.004	0.05	0.21	0.193	0.006	0.00	0.01	0.102	0.007	0.15	0.15	0.204	0.007	<0.01	**0.01**
Sex	-0.120	0.003	0.08	0.25	-0.048	0.006	0.45	0.70	-0.149	0.006	0.03	0.07	0.001	0.006	0.98	0.98
NICU stay	0.030	0.008	0.71	0.90	0.025	0.013	0.74	0.98	0.034	0.015	0.67	0.91	0.011	0.014	0.88	0.99
GA at delivery	0.153	0.001	0.08	0.23	-0.037	0.002	0.64	0.79	0.069	0.003	0.43	0.95	-0.087	0.003	0.28	0.76
GA at T1	-0.022	0.001	0.75	0.84	-0.021	0.001	0.74	0.95	-0.051	0.001	0.46	0.83	-0.009	0.001	0.89	0.89
PMA at scan	0.286	0.001	<0.001	**<0.001**	-0.498	0.002	<0.001	**<0.001**	-0.402	0.003	<0.001	**<0.001**	-0.470	0.002	<0.001	**<0.001**
IL-8 T1:SES group	-0.032	0.004	0.73	0.83	-0.004	0.007	0.96	0.96	-0.032	0.008	0.73	1.00	0.008	0.008	0.93	0.93
**Corticospinal Tract**																
IL-8 T1	0.020	0.003	0.81	0.99	0.013	0.004	0.86	0.92	0.031	0.003	0.68	0.88	0.005	0.005	0.95	0.95
SES group	0.087	0.004	0.18	0.54	0.127	0.005	0.03	**0.03**	0.284	0.004	<0.001	**<0.001**	0.063	0.006	0.28	0.28
Sex	-0.077	0.003	0.23	0.39	0.005	0.005	0.93	0.93	-0.049	0.004	0.41	0.52	0.024	0.006	0.67	0.98
NICU stay	0.098	0.008	0.18	0.90	0.001	0.011	0.98	0.98	0.074	0.010	0.28	0.91	-0.024	0.014	0.72	0.99
GA at delivery	-0.026	0.001	0.74	0.84	0.018	0.002	0.79	0.79	0.005	0.002	0.95	0.95	0.021	0.002	0.77	0.87
GA at T1	0.005	0.001	0.94	0.94	0.022	0.001	0.69	0.95	0.045	0.001	0.45	0.83	0.013	0.001	0.83	0.89
PMA at scan	0.517	0.001	<0.001	**<0.001**	-0.678	0.002	<0.001	**<0.001**	-0.582	0.002	<0.001	**<0.001**	-0.660	0.002	<0.001	**<0.001**
IL-8 T1:SES group	-0.052	0.004	0.53	0.83	0.010	0.006	0.89	0.96	-0.031	0.005	0.68	1.00	0.024	0.008	0.75	0.92
**Optic Radiation**																
IL-8 T1	0.043	0.003	0.60	0.99	-0.008	0.005	0.92	0.92	0.004	0.005	0.97	0.97	-0.019	0.006	0.81	0.91
SES group	0.056	0.004	0.38	0.85	0.194	0.007	<0.01	**0.01**	0.305	0.006	<0.001	**<0.001**	0.138	0.007	0.02	0.07
Sex	-0.074	0.004	0.23	0.39	-0.070	0.006	0.25	0.70	-0.174	0.006	0.01	0.05	-0.032	0.007	0.59	0.98
NICU stay	-0.088	0.008	0.22	0.90	0.045	0.014	0.52	0.98	0.008	0.014	0.91	0.91	0.059	0.016	0.40	0.99
GA at delivery	-0.097	0.001	0.21	0.38	-0.028	0.003	0.71	0.79	-0.102	0.002	0.23	0.95	0.002	0.003	0.97	0.97
GA at T1	-0.025	0.001	0.69	0.84	0.012	0.001	0.84	0.95	0.028	0.001	0.68	0.83	0.020	0.002	0.74	0.89
PMA at scan	0.555	0.001	<0.001	**<0.001**	-0.562	0.003	<0.001	**<0.001**	-0.303	0.002	<0.001	**<0.001**	-0.603	0.003	<0.001	**<0.001**
IL-8 T1:SES group	-0.064	0.004	0.43	0.83	0.036	0.008	0.65	0.96	0.021	0.008	0.81	1.00	0.047	0.009	0.54	0.92

**Uncinate Fasciculus**																
IL-8 T1	0.018	0.002	0.83	0.99	0.076	0.004	0.35	0.63	0.093	0.005	0.31	0.75	0.061	0.004	0.44	0.79
SES group	0.006	0.003	0.93	0.95	0.162	0.005	0.01	**0.02**	0.189	0.006	0.01	**0.01**	0.124	0.006	0.04	0.07
Sex	-0.132	0.003	0.03	0.16	-0.062	0.005	0.32	0.70	-0.164	0.006	0.02	0.05	-0.009	0.005	0.87	0.98
NICU stay	0.009	0.007	0.90	0.90	-0.008	0.012	0.91	0.98	-0.019	0.014	0.81	0.91	-0.001	0.013	0.99	0.99
GA at delivery	0.110	0.001	0.16	0.36	0.099	0.002	0.21	0.79	0.184	0.002	0.03	0.31	0.040	0.002	0.59	0.76
GA at T1	-0.127	0.001	0.04	0.37	0.021	0.001	0.73	0.95	-0.059	0.001	0.39	0.83	0.047	0.001	0.43	0.78
PMA at scan	0.450	0.001	<0.001	**<0.001**	-0.584	0.002	<0.001	**<0.001**	-0.333	0.002	<0.001	**<0.001**	-0.616	0.002	<0.001	**<0.001**
IL-8 T1:SES group	-0.045	0.004	0.58	0.83	-0.095	0.006	0.24	0.54	-0.131	0.008	0.15	0.71	-0.066	0.007	0.40	0.92
**Inferior Fronto-Occipital Fasciculus**																
IL-8 T1	0.001	0.003	0.99	0.99	0.048	0.004	0.51	0.77	0.080	0.003	0.34	0.75	0.032	0.005	0.65	0.86
SES group	0.003	0.004	0.95	0.95	0.168	0.006	<0.01	**0.01**	0.315	0.005	<0.001	**<0.001**	0.120	0.007	0.03	0.07
Sex	-0.034	0.004	0.55	0.62	-0.091	0.006	0.10	0.70	-0.180	0.004	<0.01	**0.04**	-0.053	0.007	0.33	0.98
NICU stay	-0.009	0.008	0.89	0.90	0.025	0.013	0.70	0.98	0.038	0.010	0.60	0.91	0.020	0.016	0.76	0.99
GA at delivery	0.064	0.001	0.36	0.55	-0.053	0.002	0.44	0.79	-0.007	0.002	0.93	0.95	-0.060	0.003	0.39	0.76
GA at T1	-0.076	0.001	0.18	0.50	0.037	0.001	0.50	0.95	-0.016	0.001	0.80	0.83	0.058	0.002	0.29	0.78
PMA at scan	0.604	0.001	<0.001	**<0.001**	-0.644	0.002	<0.001	**<0.001**	-0.440	0.002	<0.001	**<0.001**	-0.650	0.003	<0.001	**<0.001**
IL-8 T1:SES group	-0.028	0.004	0.70	0.83	0.013	0.007	0.86	0.96	0.000	0.005	1.00	1.00	0.016	0.009	0.82	0.92
**Anterior Limb of Internal Capsule**																
IL-8 T1	-0.060	0.002	0.41	0.99	0.075	0.005	0.34	0.63	0.062	0.005	0.48	0.78	0.075	0.006	0.32	0.73
SES group	0.011	0.003	0.85	0.95	0.139	0.007	0.02	**0.03**	0.202	0.006	<0.01	**0.01**	0.109	0.008	0.07	0.09
Sex	-0.059	0.003	0.29	0.39	-0.020	0.007	0.74	0.83	-0.060	0.006	0.37	0.52	-0.003	0.008	0.95	0.98
NICU stay	0.020	0.007	0.76	0.90	0.028	0.016	0.69	0.98	0.048	0.014	0.54	0.91	0.020	0.017	0.77	0.99
GA at delivery	0.134	0.001	0.06	0.23	-0.052	0.003	0.48	0.79	0.020	0.002	0.81	0.95	-0.074	0.003	0.31	0.76
GA at T1	-0.046	0.001	0.41	0.61	0.050	0.002	0.40	0.95	0.037	0.001	0.58	0.83	0.051	0.002	0.37	0.78
PMA at scan	0.567	0.001	<0.001	**<0.001**	-0.560	0.003	<0.001	**<0.001**	-0.400	0.002	<0.001	**<0.001**	-0.587	0.003	<0.001	**<0.001**
IL-8 T1:SES group	-0.064	0.004	0.38	0.83	0.027	0.008	0.73	0.96	-0.008	0.007	0.93	1.00	0.038	0.009	0.62	0.92
**Inferior Cingulum Bundle**																
IL-8 T1	0.038	0.002	0.67	0.99	-0.082	0.003	0.30	0.63	-0.058	0.003	0.52	0.78	-0.082	0.003	0.29	0.73
SES group	-0.023	0.003	0.75	0.95	0.222	0.003	<0.001	**<0.01**	0.196	0.005	0.01	**0.01**	0.193	0.004	<0.01	**0.01**
Sex	-0.003	0.003	0.97	0.97	-0.044	0.003	0.47	0.70	-0.039	0.004	0.58	0.58	-0.036	0.003	0.54	0.98
NICU stay	0.064	0.006	0.42	0.90	-0.008	0.007	0.91	0.98	0.028	0.010	0.73	0.91	-0.026	0.008	0.70	0.99
GA at delivery	-0.153	0.001	0.08	0.23	0.076	0.001	0.32	0.79	-0.034	0.002	0.70	0.95	0.120	0.001	0.11	0.76
GA at T1	-0.084	0.001	0.22	0.50	-0.004	0.001	0.95	0.95	-0.052	0.001	0.46	0.83	0.026	0.001	0.66	0.89
PMA at scan	0.423	0.001	<0.001	**<0.001**	-0.605	0.001	<0.001	**<0.001**	-0.321	0.002	<0.001	**<0.001**	-0.645	0.001	<0.001	**<0.001**
IL-8 T1:SES group	-0.030	0.003	0.74	0.83	0.130	0.004	0.10	0.54	0.116	0.005	0.20	0.71	0.115	0.004	0.14	0.74
**Fornix**																
IL-8 T1	-0.019	0.002	0.84	0.99	-0.125	0.003	0.11	0.48	-0.148	0.004	0.09	0.75	-0.096	0.003	0.21	0.73
SES group	-0.010	0.002	0.88	0.95	0.146	0.004	0.02	**0.02**	0.163	0.005	0.02	**0.02**	0.123	0.004	0.04	0.07
Sex	-0.072	0.002	0.30	0.39	-0.019	0.003	0.74	0.83	-0.043	0.005	0.51	0.57	0.003	0.004	0.96	0.98
NICU stay	0.031	0.005	0.71	0.90	0.046	0.008	0.50	0.98	0.059	0.011	0.43	0.91	0.033	0.008	0.63	0.99
GA at delivery	-0.049	0.001	0.58	0.65	0.024	0.001	0.74	0.79	-0.007	0.002	0.93	0.95	0.043	0.001	0.56	0.76
GA at T1	-0.076	0.000	0.28	0.50	0.081	0.001	0.17	0.77	0.065	0.001	0.32	0.83	0.087	0.001	0.14	0.62

PMA at scan	0.327	0.001	<0.001	**<0.001**	-0.625	0.001	<0.001	**<0.001**	-0.477	0.002	<0.001	**<0.001**	-0.636	0.001	<0.001	**<0.001**
IL-8 T1:SES group	-0.076	0.003	0.40	0.83	0.098	0.004	0.20	0.54	0.062	0.006	0.46	1.00	0.106	0.004	0.17	0.74
**IL-8 Trimester 2**																
	**FA**				**MD**				**AD**				**RD**			
	**β**	**SE**	***p***	***q***	**β**	**SE**	***p***	***q***	**β**	**SE**	***p***	***q***	**β**	**SE**	***p***	***q***
**Corpus Callosum**																
IL-8 T2	-0.041	0.002	0.61	0.98	-0.008	0.002	0.91	1.00	0.051	0.003	0.58	0.97	0.019	0.003	0.80	0.92
SES group	0.034	0.004	0.59	0.87	0.005	0.004	0.93	0.93	0.093	0.005	0.21	0.27	-0.015	0.006	0.80	0.80
Sex	-0.088	0.004	0.15	0.45	-0.026	0.004	0.66	0.74	-0.093	0.004	0.19	0.34	0.032	0.005	0.58	0.87
NICU stay	-0.085	0.009	0.25	0.46	-0.024	0.010	0.75	0.91	0.023	0.010	0.79	0.93	0.000	0.013	1.00	1.00
GA at delivery	0.046	0.001	0.57	0.73	-0.042	0.001	0.61	0.79	0.145	0.002	0.14	0.54	-0.079	0.002	0.33	0.85
GA at T2	0.048	0.001	0.45	0.88	0.001	0.001	0.99	0.99	0.060	0.001	0.42	0.63	-0.010	0.001	0.87	0.90
PMA at scan	0.478	0.001	<0.001	**<0.001**	-0.571	0.001	<0.001	**<0.001**	-0.217	0.002	0.01	**0.01**	-0.563	0.002	<0.001	**<0.001**
IL-8 T2:SES group	-0.054	0.004	0.49	0.65	0.050	0.004	0.50	0.92	-0.018	0.004	0.84	0.90	0.039	0.005	0.60	0.96
**Superior Cingulum Bundle**																
IL-8 T2	-0.024	0.002	0.77	0.98	0.067	0.003	0.40	1.00	0.051	0.004	0.56	0.97	0.065	0.004	0.41	0.92
SES group	-0.213	0.003	<0.01	**0.02**	0.138	0.006	0.03	0.07	-0.004	0.007	0.96	0.96	0.184	0.007	<0.01	**0.02**
Sex	-0.097	0.003	0.14	0.45	0.010	0.005	0.88	0.88	-0.076	0.006	0.27	0.34	0.046	0.006	0.46	0.83
NICU stay	0.030	0.008	0.72	0.73	0.050	0.013	0.53	0.91	0.064	0.015	0.45	0.93	0.032	0.014	0.68	0.88
GA at delivery	0.078	0.001	0.38	0.57	0.087	0.002	0.31	0.79	0.139	0.002	0.14	0.54	0.047	0.002	0.58	0.85
GA at T2	0.016	0.000	0.81	0.88	0.008	0.001	0.91	0.99	0.036	0.001	0.61	0.69	-0.008	0.001	0.90	0.90
PMA at scan	0.401	0.001	<0.001	**<0.001**	-0.541	0.002	<0.001	**<0.001**	-0.389	0.002	<0.001	**<0.001**	-0.536	0.002	<0.001	**<0.001**
IL-8 T2:SES group	0.141	0.003	0.09	0.65	-0.076	0.005	0.34	0.92	0.010	0.006	0.90	0.90	-0.107	0.006	0.18	0.96
**Corticospinal Tract**																
IL-8 T2	-0.015	0.002	0.85	0.98	0.000	0.003	1.00	1.00	-0.023	0.003	0.74	0.97	0.020	0.004	0.77	0.92
SES group	-0.023	0.004	0.71	0.87	0.123	0.006	0.02	0.07	0.187	0.005	<0.01	**0.01**	0.093	0.007	0.09	0.15
Sex	0.044	0.004	0.45	0.81	-0.051	0.005	0.33	0.74	-0.038	0.004	0.50	0.50	-0.048	0.006	0.37	0.83
NICU stay	-0.074	0.008	0.32	0.47	0.031	0.012	0.64	0.91	0.031	0.010	0.65	0.93	0.032	0.015	0.62	0.88
GA at delivery	0.035	0.001	0.66	0.74	-0.045	0.002	0.53	0.79	-0.004	0.001	0.95	0.95	-0.022	0.002	0.76	0.85
GA at T2	0.063	0.001	0.30	0.88	-0.010	0.001	0.85	0.99	0.031	0.001	0.60	0.69	-0.030	0.001	0.59	0.90
PMA at scan	0.545	0.001	<0.001	**<0.001**	-0.691	0.002	<0.001	**<0.001**	-0.643	0.002	<0.001	**<0.001**	-0.671	0.002	<0.001	**<0.001**
IL-8 T2:SES group	0.032	0.004	0.67	0.67	0.023	0.005	0.73	0.92	0.065	0.004	0.36	0.90	0.002	0.006	0.98	0.98
**Optic Radiation**																
IL-8 T2	-0.029	0.002	0.69	0.98	0.055	0.004	0.41	1.00	0.021	0.004	0.79	0.97	0.047	0.004	0.47	0.92
SES group	0.036	0.004	0.54	0.87	0.137	0.006	0.01	0.05	0.230	0.006	<0.01	**0.01**	0.094	0.007	0.08	0.15
Sex	-0.001	0.004	0.99	0.99	-0.140	0.006	0.01	**0.04**	-0.214	0.006	<0.01	**0.01**	-0.105	0.006	0.04	0.19
NICU stay	-0.129	0.009	0.07	0.46	0.104	0.013	0.12	0.91	0.037	0.014	0.64	0.93	0.118	0.015	0.07	0.60
GA at delivery	0.017	0.001	0.83	0.83	-0.091	0.002	0.20	0.79	-0.088	0.002	0.32	0.54	-0.080	0.002	0.25	0.85
GA at T2	0.066	0.001	0.27	0.88	0.031	0.001	0.57	0.99	0.079	0.001	0.23	0.42	0.008	0.001	0.88	0.90
PMA at scan	0.554	0.001	<0.001	**<0.001**	-0.623	0.002	<0.001	**<0.001**	-0.359	0.002	<0.001	**<0.001**	-0.649	0.002	<0.001	**<0.001**
IL-8 T2:SES group	0.036	0.004	0.62	0.67	-0.009	0.006	0.89	0.92	0.030	0.006	0.72	0.90	-0.015	0.006	0.82	0.96
**Uncinate Fasciculus**																
IL-8 T2	-0.004	0.002	0.95	0.98	-0.086	0.003	0.27	1.00	-0.098	0.004	0.28	0.97	-0.069	0.003	0.35	0.92

SES group	-0.041	0.003	0.49	0.87	0.110	0.005	0.08	0.15	0.095	0.006	0.19	0.27	0.098	0.006	0.10	0.15
Sex	-0.054	0.003	0.36	0.80	-0.030	0.005	0.63	0.74	-0.079	0.006	0.26	0.34	-0.009	0.005	0.88	0.90
NICU stay	-0.035	0.007	0.63	0.73	0.009	0.012	0.91	0.91	-0.021	0.014	0.81	0.93	0.021	0.012	0.77	0.88
GA at delivery	0.126	0.001	0.11	0.51	0.023	0.002	0.79	0.79	0.128	0.002	0.18	0.54	-0.030	0.002	0.70	0.85
GA at T2	0.026	0.000	0.67	0.88	0.083	0.001	0.19	0.99	0.113	0.001	0.12	0.42	0.055	0.001	0.36	0.90
PMA at scan	0.533	0.001	<0.001	**<0.001**	-0.549	0.002	<0.001	**<0.001**	-0.235	0.002	<0.001	**<0.001**	-0.609	0.002	<0.001	**<0.001**
IL-8 T2:SES group	0.049	0.003	0.50	0.65	0.039	0.005	0.62	0.92	0.074	0.006	0.40	0.90	0.018	0.005	0.80	0.96
**Inferior Fronto-Occipital Fasciculus**																
IL-8 T2	0.001	0.002	0.98	0.98	-0.010	0.004	0.89	1.00	-0.004	0.003	0.97	0.97	-0.009	0.005	0.89	0.92
SES group	-0.005	0.004	0.93	0.93	0.082	0.006	0.14	0.21	0.157	0.005	0.02	0.05	0.063	0.008	0.25	0.32
Sex	0.012	0.004	0.82	0.92	-0.101	0.006	0.06	0.19	-0.169	0.004	0.01	**0.03**	-0.071	0.007	0.18	0.53
NICU stay	-0.076	0.009	0.25	0.46	0.066	0.014	0.33	0.91	0.018	0.010	0.83	0.93	0.073	0.017	0.27	0.81
GA at delivery	0.082	0.001	0.25	0.56	-0.024	0.002	0.75	0.79	0.069	0.002	0.44	0.54	-0.045	0.003	0.53	0.85
GA at T2	0.053	0.001	0.32	0.88	0.013	0.001	0.82	0.99	0.097	0.001	0.15	0.42	-0.010	0.001	0.86	0.90
PMA at scan	0.637	0.001	<0.001	**<0.001**	-0.653	0.002	<0.001	**<0.001**	-0.435	0.002	<0.001	**<0.001**	-0.663	0.003	<0.001	**<0.001**
IL-8 T2:SES group	0.065	0.004	0.32	0.65	-0.025	0.006	0.72	0.92	0.020	0.004	0.81	0.90	-0.037	0.007	0.58	0.96
**Anterior Limb of Internal Capsule**																
IL-8 T2	-0.017	0.002	0.80	0.98	0.023	0.004	0.76	1.00	0.028	0.004	0.74	0.97	0.020	0.005	0.78	0.92
SES group	0.015	0.003	0.77	0.87	0.055	0.008	0.35	0.40	0.089	0.006	0.19	0.27	0.041	0.008	0.47	0.53
Sex	-0.015	0.003	0.77	0.92	-0.032	0.007	0.58	0.74	-0.054	0.006	0.42	0.47	-0.022	0.008	0.69	0.88
NICU stay	-0.085	0.007	0.19	0.46	0.050	0.016	0.49	0.91	0.009	0.014	0.91	0.93	0.061	0.018	0.38	0.85
GA at delivery	0.149	0.001	0.03	0.30	-0.034	0.002	0.67	0.79	0.064	0.002	0.48	0.54	-0.064	0.003	0.40	0.85
GA at T2	-0.010	0.000	0.86	0.88	0.054	0.001	0.37	0.99	0.087	0.001	0.20	0.42	0.042	0.001	0.46	0.90
PMA at scan	0.619	0.001	<0.001	**<0.001**	-0.596	0.002	<0.001	**<0.001**	-0.439	0.002	<0.001	**<0.001**	-0.622	0.003	<0.001	**<0.001**
IL-8 T2:SES group	0.048	0.003	0.46	0.65	-0.034	0.007	0.64	0.92	-0.024	0.006	0.78	0.90	-0.036	0.008	0.61	0.96
**Inferior Cingulum Bundle**																
IL-8 T2	-0.062	0.002	0.46	0.98	-0.020	0.002	0.78	1.00	-0.066	0.003	0.44	0.97	0.007	0.002	0.92	0.92
SES group	-0.114	0.003	0.09	0.27	0.232	0.003	<0.001	**<0.01**	0.158	0.005	0.02	0.05	0.206	0.004	<0.001	**<0.01**
Sex	-0.039	0.003	0.55	0.82	-0.173	0.003	<0.01	**0.02**	-0.191	0.004	<0.01	**0.02**	-0.141	0.003	0.01	0.09
NICU stay	-0.105	0.006	0.20	0.46	0.045	0.007	0.52	0.91	-0.048	0.010	0.57	0.93	0.091	0.008	0.18	0.81
GA at delivery	-0.118	0.001	0.18	0.55	0.041	0.001	0.59	0.79	-0.071	0.001	0.44	0.54	0.098	0.001	0.19	0.85
GA at T2	0.039	0.000	0.56	0.88	0.066	0.000	0.25	0.99	0.096	0.001	0.17	0.42	0.056	0.000	0.32	0.90
PMA at scan	0.462	0.001	<0.001	**<0.001**	-0.639	0.001	<0.001	**<0.001**	-0.322	0.002	<0.001	**<0.001**	-0.677	0.001	<0.001	**<0.001**
IL-8 T2:SES group	0.101	0.003	0.22	0.65	0.007	0.003	0.92	0.92	0.074	0.004	0.38	0.90	-0.025	0.003	0.72	0.96
**Fornix**																
IL-8 T2	-0.029	0.001	0.73	0.98	0.005	0.002	0.94	1.00	-0.012	0.003	0.88	0.97	0.016	0.002	0.82	0.92
SES group	-0.162	0.002	0.02	0.08	0.078	0.004	0.18	0.24	0.010	0.005	0.88	0.96	0.115	0.004	0.05	0.14
Sex	-0.113	0.002	0.09	0.45	-0.034	0.004	0.55	0.74	-0.087	0.005	0.18	0.34	0.007	0.004	0.90	0.90
NICU stay	0.028	0.005	0.73	0.73	-0.016	0.008	0.82	0.91	-0.007	0.011	0.93	0.93	-0.019	0.008	0.78	0.88
GA at delivery	0.086	0.001	0.34	0.57	0.025	0.001	0.75	0.79	0.079	0.002	0.37	0.54	-0.008	0.001	0.92	0.92
GA at T2	0.010	0.000	0.88	0.88	-0.017	0.000	0.77	0.99	-0.019	0.001	0.77	0.77	-0.014	0.001	0.81	0.90
PMA at scan	0.386	0.001	<0.001	**<0.001**	-0.646	0.001	<0.001	**<0.001**	-0.481	0.002	<0.001	**<0.001**	-0.659	0.001	<0.001	**<0.001**
IL-8 T2:SES group	0.081	0.002	0.33	0.65	0.020	0.003	0.79	0.92	0.068	0.005	0.41	0.90	-0.013	0.004	0.85	0.96

IL-8 Trimester 3																
	**FA**				**MD**				**AD**				**RD**			
	**β**	**SE**	***p***	***q***	**β**	**SE**	***p***	***q***	**β**	**SE**	***p***	***q***	**β**	**SE**	***p***	***q***
**Corpus Callosum**																
IL-8 T3	0.011	0.002	0.87	0.93	0.016	0.002	0.81	0.81	0.018	0.002	0.82	0.88	0.023	0.003	0.73	0.88
SES group	0.104	0.004	0.08	0.24	0.005	0.004	0.93	0.93	0.188	0.004	0.01	**0.01**	-0.042	0.005	0.45	0.45
Sex	-0.127	0.003	0.02	0.20	-0.006	0.004	0.92	0.99	-0.095	0.004	0.13	0.24	0.060	0.005	0.25	0.75
NICU stay	-0.048	0.009	0.45	0.65	-0.022	0.010	0.72	0.96	0.004	0.010	0.96	0.96	-0.002	0.013	0.97	0.97
GA at delivery	-0.025	0.002	0.72	0.83	0.000	0.002	1.00	1.00	0.032	0.002	0.70	0.95	-0.008	0.002	0.91	0.99
GA at T3	0.023	0.001	0.70	0.88	-0.031	0.001	0.59	0.66	-0.034	0.001	0.62	0.64	-0.040	0.001	0.49	0.73
PMA at scan	0.465	0.001	<0.001	**<0.001**	-0.561	0.001	<0.001	**<0.001**	-0.165	0.002	0.02	**0.02**	-0.556	0.002	<0.001	**<0.001**
IL-8 T3:SES group	-0.032	0.003	0.65	0.84	-0.051	0.004	0.45	0.98	-0.068	0.004	0.40	0.89	-0.034	0.005	0.60	0.99
**Superior Cingulum Bundle**																
IL-8 T3	-0.059	0.002	0.44	0.93	0.030	0.003	0.67	0.81	-0.019	0.003	0.80	0.88	0.059	0.003	0.41	0.88
SES group	-0.134	0.003	0.04	0.17	0.157	0.005	0.01	**0.01**	0.062	0.006	0.32	0.32	0.175	0.006	<0.01	**0.01**
Sex	-0.090	0.003	0.13	0.30	-0.022	0.005	0.69	0.98	-0.105	0.005	0.07	0.17	0.017	0.005	0.76	0.91
NICU stay	-0.045	0.008	0.52	0.65	-0.012	0.013	0.86	0.96	-0.056	0.015	0.42	0.54	0.008	0.015	0.91	0.97
GA at delivery	0.027	0.001	0.74	0.83	0.054	0.002	0.47	0.78	0.071	0.002	0.37	0.84	0.036	0.002	0.63	0.95
GA at T3	-0.022	0.001	0.74	0.88	-0.034	0.001	0.58	0.66	-0.055	0.001	0.39	0.64	-0.020	0.001	0.74	0.84
PMA at scan	0.316	0.001	<0.001	**<0.001**	-0.511	0.002	<0.001	**<0.001**	-0.398	0.002	<0.001	**<0.001**	-0.486	0.002	<0.001	**<0.001**
IL-8 T3:SES group	0.006	0.003	0.93	0.99	0.013	0.005	0.85	0.98	0.045	0.006	0.55	0.91	-0.010	0.006	0.88	0.99
**Corticospinal Tract**																
IL-8 T3	0.021	0.002	0.75	0.93	0.041	0.003	0.48	0.81	0.087	0.002	0.18	0.82	0.017	0.003	0.78	0.88
SES group	0.030	0.003	0.59	0.82	0.145	0.005	<0.01	**0.01**	0.282	0.004	<0.001	**<0.001**	0.090	0.006	0.07	0.08
Sex	-0.021	0.003	0.68	0.80	-0.031	0.004	0.49	0.89	-0.066	0.004	0.19	0.29	-0.014	0.005	0.76	0.91
NICU stay	-0.054	0.008	0.37	0.65	0.002	0.012	0.97	0.96	-0.063	0.010	0.29	0.45	0.013	0.014	0.80	0.97
GA at delivery	-0.010	0.001	0.88	0.88	-0.040	0.002	0.52	0.78	-0.072	0.002	0.29	0.84	0.000	0.002	0.99	0.99
GA at T3	0.095	0.001	0.09	0.80	-0.094	0.001	0.06	0.54	-0.059	0.001	0.29	0.64	-0.098	0.001	0.05	0.49
PMA at scan	0.549	0.001	<0.001	**<0.001**	-0.659	0.002	<0.001	**<0.001**	-0.542	0.001	<0.001	**<0.001**	-0.656	0.002	<0.001	**<0.001**
IL-8 T3:SES group	-0.134	0.003	0.04	0.35	0.037	0.004	0.52	0.98	-0.076	0.004	0.23	0.70	0.078	0.005	0.19	0.99
**Optic Radiation**																
IL-8 T3	0.010	0.002	0.88	0.93	0.079	0.003	0.21	0.81	0.063	0.003	0.39	0.87	0.057	0.004	0.37	0.88
SES group	0.091	0.004	0.11	0.24	0.191	0.006	<0.001	**<0.01**	0.339	0.006	<0.001	**<0.001**	0.119	0.007	0.02	**0.04**
Sex	0.008	0.003	0.88	0.88	-0.100	0.005	0.04	0.34	-0.150	0.005	0.01	0.07	-0.075	0.006	0.12	0.55
NICU stay	-0.114	0.009	0.07	0.60	0.024	0.014	0.68	0.96	-0.070	0.014	0.30	0.45	0.055	0.016	0.34	0.97
GA at delivery	-0.053	0.001	0.46	0.83	-0.075	0.002	0.26	0.78	-0.144	0.002	0.06	0.55	-0.039	0.003	0.55	0.95
GA at T3	0.015	0.001	0.79	0.88	-0.039	0.001	0.47	0.66	-0.029	0.001	0.64	0.64	-0.038	0.001	0.48	0.73
PMA at scan	0.548	0.001	<0.001	**<0.001**	-0.560	0.002	<0.001	**<0.001**	-0.270	0.002	<0.001	**<0.001**	-0.602	0.002	<0.001	**<0.001**
IL-8 T3:SES group	-0.052	0.003	0.43	0.71	0.013	0.005	0.84	0.98	0.005	0.005	0.95	0.95	0.026	0.006	0.67	0.99
**Uncinate Fasciculus**																
IL-8 T3	0.054	0.002	0.43	0.93	0.039	0.003	0.58	0.81	0.066	0.003	0.39	0.87	0.015	0.003	0.83	0.88
SES group	-0.027	0.003	0.64	0.82	0.166	0.005	<0.01	**0.01**	0.159	0.006	0.01	**0.02**	0.141	0.005	0.01	**0.03**
Sex	-0.086	0.003	0.11	0.30	-0.049	0.004	0.36	0.81	-0.118	0.005	0.05	0.14	-0.012	0.005	0.81	0.91

NICU stay	-0.035	0.007	0.58	0.65	-0.134	0.012	0.04	0.33	-0.172	0.014	0.02	0.14	-0.092	0.013	0.13	0.97
GA at delivery	0.145	0.001	0.04	0.40	0.014	0.002	0.85	1.00	0.113	0.002	0.16	0.74	-0.039	0.002	0.58	0.95
GA at T3	-0.009	0.001	0.88	0.88	-0.049	0.001	0.41	0.66	-0.050	0.001	0.44	0.64	-0.040	0.001	0.48	0.73
PMA at scan	0.470	0.001	<0.001	**<0.001**	-0.514	0.002	<0.001	**<0.001**	-0.242	0.002	<0.01	**0.02**	-0.567	0.002	<0.001	**<0.001**
IL-8 T3:SES group	-0.048	0.003	0.48	0.71	-0.081	0.004	0.24	0.98	-0.121	0.005	0.11	0.51	-0.046	0.005	0.48	0.99
**Inferior Fronto-Occipital Fasciculus**																
IL-8 T3	-0.013	0.002	0.83	0.93	0.028	0.003	0.65	0.81	0.041	0.003	0.57	0.88	0.023	0.004	0.71	0.88
SES group	0.016	0.004	0.76	0.85	0.135	0.005	0.01	**0.01**	0.259	0.004	<0.001	**<0.001**	0.093	0.007	0.07	0.08
Sex	-0.020	0.003	0.68	0.80	-0.076	0.005	0.11	0.34	-0.137	0.004	0.02	0.07	-0.046	0.006	0.33	0.75
NICU stay	-0.052	0.009	0.36	0.65	-0.032	0.013	0.57	0.96	-0.100	0.011	0.13	0.32	-0.005	0.016	0.93	0.97
GA at delivery	0.053	0.001	0.42	0.83	-0.045	0.002	0.49	0.78	-0.001	0.002	0.99	0.99	-0.050	0.003	0.44	0.95
GA at T3	0.046	0.001	0.38	0.80	-0.050	0.001	0.34	0.66	-0.034	0.001	0.58	0.64	-0.052	0.001	0.31	0.73
PMA at scan	0.603	0.001	<0.001	**<0.001**	-0.629	0.002	<0.001	**<0.001**	-0.398	0.002	<0.001	**<0.001**	-0.642	0.002	<0.001	**<0.001**
IL-8 T3:SES group	-0.046	0.003	0.45	0.71	0.007	0.005	0.91	0.98	-0.037	0.004	0.61	0.91	0.019	0.006	0.75	0.99
**Anterior Limb of Internal Capsule**																
IL-8 T3	-0.006	0.002	0.93	0.93	0.023	0.004	0.72	0.81	-0.011	0.003	0.88	0.88	0.024	0.004	0.70	0.88
SES group	0.009	0.003	0.86	0.86	0.132	0.007	0.02	**0.02**	0.189	0.006	<0.01	**<0.01**	0.103	0.008	0.05	0.07
Sex	-0.047	0.003	0.32	0.58	-0.015	0.006	0.77	0.98	-0.040	0.005	0.48	0.48	-0.002	0.007	0.96	0.96
NICU stay	-0.017	0.007	0.75	0.75	-0.063	0.016	0.29	0.96	-0.113	0.014	0.09	0.32	-0.042	0.018	0.46	0.97
GA at delivery	0.095	0.001	0.14	0.62	-0.045	0.003	0.51	0.78	0.018	0.002	0.81	0.95	-0.060	0.003	0.37	0.95
GA at T3	0.056	0.001	0.28	0.80	-0.071	0.001	0.20	0.66	-0.059	0.001	0.35	0.64	-0.070	0.001	0.19	0.73
PMA at scan	0.598	0.001	<0.001	**<0.001**	-0.588	0.002	<0.001	**<0.001**	-0.444	0.002	<0.001	**<0.001**	-0.611	0.003	<0.001	**<0.001**
IL-8 T3:SES group	-0.057	0.003	0.34	0.71	0.002	0.006	0.98	0.98	-0.017	0.005	0.81	0.92	0.014	0.007	0.82	0.99
**Inferior Cingulum Bundle**																
IL-8 T3	-0.145	0.001	0.05	0.49	-0.056	0.002	0.39	0.81	-0.154	0.002	0.04	0.39	0.010	0.002	0.88	0.88
SES group	-0.031	0.003	0.62	0.82	0.210	0.003	<0.001	**<0.01**	0.192	0.004	<0.01	**<0.01**	0.159	0.003	<0.01	**0.01**
Sex	0.021	0.002	0.72	0.80	-0.089	0.003	0.08	0.34	-0.055	0.004	0.35	0.40	-0.091	0.003	0.07	0.55
NICU stay	-0.094	0.006	0.18	0.65	-0.039	0.007	0.51	0.96	-0.103	0.010	0.14	0.32	0.025	0.008	0.68	0.97
GA at delivery	-0.066	0.001	0.41	0.83	0.077	0.001	0.26	0.78	0.016	0.002	0.84	0.95	0.106	0.001	0.12	0.95
GA at T3	-0.049	0.000	0.45	0.80	-0.037	0.001	0.50	0.66	-0.076	0.001	0.24	0.64	-0.001	0.001	0.99	0.99
PMA at scan	0.387	0.001	<0.001	**<0.001**	-0.624	0.001	<0.001	**<0.001**	-0.343	0.001	<0.001	**<0.001**	-0.639	0.001	<0.001	**<0.001**
IL-8 T3:SES group	0.106	0.002	0.16	0.71	0.069	0.003	0.28	0.98	0.140	0.004	0.06	0.51	0.006	0.003	0.93	0.99
**Fornix**																
IL-8 T3	-0.022	0.001	0.77	0.93	0.046	0.002	0.48	0.81	0.020	0.003	0.79	0.88	0.055	0.002	0.39	0.88
SES group	-0.136	0.002	0.03	0.17	0.139	0.003	0.01	**0.01**	0.083	0.005	0.18	0.20	0.159	0.003	<0.01	**0.01**
Sex	-0.115	0.002	0.05	0.23	0.001	0.003	0.99	0.99	-0.057	0.004	0.32	0.40	0.037	0.003	0.45	0.81
NICU stay	-0.053	0.005	0.44	0.65	-0.014	0.008	0.81	0.96	-0.047	0.011	0.48	0.54	0.006	0.008	0.92	0.97
GA at delivery	0.037	0.001	0.64	0.83	0.007	0.001	0.92	1.00	0.026	0.002	0.73	0.95	-0.004	0.001	0.95	0.99
GA at T3	-0.050	0.000	0.43	0.80	0.000	0.001	1.00	1.00	-0.040	0.001	0.53	0.64	0.019	0.001	0.73	0.84
PMA at scan	0.349	0.001	<0.001	**<0.001**	-0.613	0.001	<0.001	**<0.001**	-0.461	0.002	<0.001	**<0.001**	-0.626	0.001	<0.001	**<0.001**
IL-8 T3:SES group	0.001	0.002	0.99	0.99	0.006	0.003	0.93	0.98	0.017	0.004	0.82	0.92	0.001	0.003	0.99	0.99
**IL-10 Trimester 1**																
	**FA**				**MD**				**AD**				**RD**			

	β	SE	*p*	*q*	β	SE	*p*	*q*	β	SE	*p*	*q*	β	SE	*p*	*q*
**Corpus Callosum**																
IL-10 T1	0.063	0.003	0.46	0.93	-0.069	0.003	0.41	0.93	-0.020	0.003	0.84	0.94	-0.073	0.004	0.37	0.86
SES group	0.150	0.004	0.02	0.20	-0.027	0.004	0.67	0.67	0.191	0.005	0.01	**0.02**	-0.102	0.005	0.11	0.12
Sex	-0.150	0.004	0.02	0.09	0.086	0.004	0.17	0.84	-0.067	0.005	0.36	0.65	0.129	0.005	0.04	0.32
NICU stay	0.019	0.009	0.79	0.98	-0.057	0.009	0.42	0.85	0.083	0.010	0.31	0.84	-0.073	0.012	0.30	0.79
GA at delivery	-0.048	0.002	0.54	0.61	0.063	0.002	0.41	0.74	-0.024	0.002	0.79	0.95	0.055	0.002	0.46	0.74
GA at T1	-0.075	0.001	0.24	0.64	0.095	0.001	0.13	0.90	0.030	0.001	0.68	0.81	0.098	0.001	0.11	0.76
PMA at scan	0.463	0.002	<0.001	**<0.001**	-0.538	0.002	<0.001	**<0.001**	-0.090	0.002	0.27	0.27	-0.540	0.002	<0.001	**<0.001**
IL-10 T1:SES group	-0.093	0.004	0.29	0.86	0.124	0.004	0.15	0.57	0.088	0.005	0.38	0.76	0.112	0.006	0.18	0.55
**Superior Cingulum Bundle**																
IL-10 T1	0.001	0.003	0.99	0.99	-0.039	0.004	0.66	0.96	-0.032	0.005	0.74	0.94	-0.026	0.005	0.76	0.86
SES group	-0.135	0.004	0.06	0.27	0.171	0.006	0.01	**0.02**	0.076	0.007	0.29	0.29	0.187	0.007	0.01	**0.04**
Sex	-0.116	0.004	0.10	0.29	-0.029	0.006	0.65	0.84	-0.124	0.007	0.08	0.17	0.014	0.006	0.82	0.91
NICU stay	0.032	0.008	0.69	0.98	0.036	0.012	0.62	0.85	0.046	0.015	0.56	0.84	0.020	0.014	0.79	0.79
GA at delivery	0.151	0.001	0.08	0.27	-0.033	0.002	0.67	0.74	0.070	0.003	0.41	0.95	-0.083	0.002	0.29	0.74
GA at T1	-0.017	0.001	0.81	0.89	-0.017	0.001	0.80	0.90	-0.043	0.001	0.54	0.81	-0.008	0.001	0.91	0.96
PMA at scan	0.294	0.001	<0.001	**<0.001**	-0.493	0.002	<0.001	**<0.001**	-0.385	0.003	<0.001	**<0.001**	-0.470	0.002	<0.001	**<0.001**
IL-10 T1:SES group	0.001	0.004	0.99	0.99	0.059	0.006	0.51	0.57	0.063	0.007	0.51	0.76	0.040	0.007	0.65	0.73
**Corticospinal Tract**																
IL-10 T1	-0.031	0.003	0.71	0.93	0.007	0.004	0.92	0.96	-0.017	0.003	0.83	0.94	0.013	0.004	0.86	0.86
SES group	0.084	0.004	0.20	0.60	0.105	0.005	0.07	0.08	0.247	0.004	<0.001	**<0.001**	0.047	0.006	0.43	0.43
Sex	-0.082	0.003	0.20	0.36	0.030	0.005	0.60	0.84	-0.016	0.004	0.78	0.78	0.045	0.006	0.43	0.91
NICU stay	0.108	0.008	0.14	0.98	0.012	0.011	0.85	0.85	0.097	0.010	0.15	0.84	-0.019	0.013	0.77	0.79
GA at delivery	-0.027	0.001	0.73	0.73	0.023	0.002	0.74	0.74	0.010	0.002	0.89	0.95	0.025	0.002	0.72	0.81
GA at T1	0.023	0.001	0.72	0.89	0.020	0.001	0.71	0.90	0.057	0.001	0.34	0.81	0.006	0.001	0.91	0.96
PMA at scan	0.522	0.001	<0.001	**<0.001**	-0.668	0.002	<0.001	**<0.001**	-0.555	0.002	<0.001	**<0.001**	-0.655	0.002	<0.001	**<0.001**
IL-10 T1:SES group	0.008	0.004	0.93	0.99	0.086	0.005	0.26	0.57	0.131	0.005	0.11	0.76	0.065	0.007	0.41	0.66
**Optic Radiation**																
IL-10 T1	-0.030	0.003	0.72	0.93	0.039	0.005	0.64	0.96	0.018	0.004	0.84	0.94	0.045	0.005	0.58	0.86
SES group	0.063	0.004	0.32	0.73	0.189	0.007	<0.01	**0.01**	0.302	0.006	<0.001	**<0.001**	0.133	0.007	0.03	0.09
Sex	-0.083	0.004	0.18	0.36	-0.062	0.006	0.32	0.84	-0.164	0.006	0.02	0.06	-0.024	0.007	0.69	0.91
NICU stay	-0.078	0.008	0.27	0.98	0.041	0.014	0.56	0.85	0.009	0.014	0.91	0.98	0.052	0.016	0.44	0.79
GA at delivery	-0.104	0.001	0.17	0.31	-0.027	0.002	0.72	0.74	-0.104	0.002	0.21	0.94	0.006	0.003	0.94	0.94
GA at T1	-0.009	0.001	0.89	0.89	-0.003	0.001	0.97	0.97	0.018	0.001	0.79	0.81	0.003	0.002	0.96	0.96
PMA at scan	0.556	0.001	<0.001	**<0.001**	-0.559	0.002	<0.001	**<0.001**	-0.293	0.002	<0.001	**<0.001**	-0.603	0.003	<0.001	**<0.001**
IL-10 T1:SES group	0.010	0.004	0.91	0.99	-0.006	0.007	0.94	0.94	0.018	0.007	0.84	0.86	-0.015	0.008	0.85	0.85
**Uncinate Fasciculus**																
IL-10 T1	0.172	0.002	0.04	0.35	-0.151	0.004	0.07	0.66	-0.055	0.005	0.56	0.94	-0.176	0.004	0.03	0.27
SES group	0.013	0.003	0.83	0.92	0.154	0.005	0.02	**0.03**	0.184	0.006	0.01	**0.02**	0.115	0.006	0.06	0.09
Sex	-0.156	0.003	0.01	0.09	-0.051	0.005	0.41	0.84	-0.169	0.006	0.02	0.06	0.007	0.006	0.91	0.91
NICU stay	-0.002	0.007	0.98	0.98	0.014	0.011	0.84	0.85	-0.002	0.014	0.98	0.98	0.022	0.012	0.75	0.79
GA at delivery	0.105	0.001	0.17	0.31	0.094	0.002	0.22	0.74	0.177	0.002	0.04	0.37	0.038	0.002	0.60	0.78

GA at T1	-0.116	0.001	0.06	0.58	0.036	0.001	0.56	0.90	-0.034	0.001	0.62	0.81	0.055	0.001	0.36	0.76
PMA at scan	0.435	0.001	<0.001	**<0.001**	-0.571	0.002	<0.001	**<0.001**	-0.326	0.002	<0.001	**<0.001**	-0.602	0.002	<0.001	**<0.001**
IL-10 T1:SES group	-0.197	0.003	0.02	0.20	0.105	0.006	0.22	0.57	-0.017	0.007	0.86	0.86	0.146	0.006	0.08	0.55
**Inferior Fronto-Occipital Fasciculus**																
IL-10 T1	0.002	0.003	0.98	0.99	-0.024	0.004	0.75	0.96	-0.041	0.003	0.64	0.94	-0.019	0.005	0.80	0.86
SES group	0.010	0.004	0.87	0.92	0.161	0.006	0.01	**0.02**	0.305	0.005	<0.001	**<0.001**	0.112	0.007	0.05	0.09
Sex	-0.044	0.004	0.44	0.50	-0.070	0.006	0.21	0.84	-0.149	0.004	0.02	0.06	-0.036	0.007	0.52	0.91
NICU stay	-0.004	0.008	0.95	0.98	0.034	0.012	0.59	0.85	0.060	0.010	0.41	0.84	0.025	0.015	0.69	0.79
GA at delivery	0.060	0.001	0.39	0.59	-0.044	0.002	0.52	0.74	0.004	0.002	0.95	0.95	-0.052	0.003	0.44	0.74
GA at T1	-0.056	0.001	0.32	0.64	0.025	0.001	0.65	0.90	-0.015	0.001	0.81	0.81	0.044	0.002	0.42	0.76
PMA at scan	0.614	0.001	<0.001	**<0.001**	-0.652	0.002	<0.001	**<0.001**	-0.435	0.002	<0.001	**<0.001**	-0.659	0.003	<0.001	**<0.001**
IL-10 T1:SES group	-0.037	0.004	0.63	0.99	0.053	0.006	0.48	0.57	0.059	0.005	0.50	0.76	0.053	0.008	0.48	0.66
**Anterior Limb of Internal Capsule**																
IL-10 T1	-0.039	0.002	0.61	0.93	-0.076	0.005	0.35	0.93	-0.133	0.004	0.14	0.94	-0.052	0.006	0.51	0.86
SES group	0.012	0.003	0.84	0.92	0.132	0.007	0.03	**0.04**	0.196	0.006	0.01	**0.01**	0.103	0.008	0.09	0.11
Sex	-0.061	0.003	0.29	0.43	0.004	0.007	0.94	0.97	-0.026	0.006	0.70	0.78	0.017	0.008	0.77	0.91
NICU stay	0.018	0.007	0.78	0.98	0.047	0.015	0.49	0.85	0.073	0.014	0.34	0.84	0.036	0.017	0.59	0.79
GA at delivery	0.120	0.001	0.09	0.27	-0.032	0.003	0.66	0.74	0.039	0.002	0.64	0.95	-0.054	0.003	0.45	0.74
GA at T1	-0.033	0.001	0.56	0.85	0.049	0.002	0.41	0.90	0.043	0.001	0.52	0.81	0.048	0.002	0.41	0.76
PMA at scan	0.585	0.001	<0.001	**<0.001**	-0.572	0.003	<0.001	**<0.001**	-0.400	0.002	<0.001	**<0.001**	-0.601	0.003	<0.001	**<0.001**
IL-10 T1:SES group	0.006	0.003	0.94	0.99	0.082	0.007	0.32	0.57	0.111	0.007	0.23	0.76	0.068	0.008	0.39	0.66
**Inferior Cingulum Bundle**																
IL-10 T1	0.054	0.002	0.56	0.93	0.004	0.002	0.96	0.96	0.042	0.003	0.66	0.94	-0.015	0.003	0.85	0.86
SES group	-0.007	0.003	0.92	0.92	0.193	0.003	<0.01	**0.01**	0.175	0.005	0.02	**0.02**	0.165	0.004	0.01	**0.04**
Sex	-0.017	0.003	0.80	0.80	-0.037	0.003	0.55	0.84	-0.040	0.004	0.57	0.78	-0.026	0.003	0.67	0.91
NICU stay	0.058	0.006	0.46	0.98	-0.020	0.007	0.77	0.85	0.013	0.010	0.87	0.98	-0.035	0.008	0.61	0.79
GA at delivery	-0.148	0.001	0.08	0.27	0.073	0.001	0.33	0.74	-0.034	0.002	0.69	0.95	0.116	0.001	0.12	0.74
GA at T1	-0.082	0.001	0.23	0.64	-0.016	0.001	0.79	0.90	-0.063	0.001	0.37	0.81	0.015	0.001	0.80	0.96
PMA at scan	0.402	0.001	<0.001	**<0.001**	-0.583	0.001	<0.001	**<0.001**	-0.310	0.002	<0.001	**<0.001**	-0.620	0.001	<0.001	**<0.001**
IL-10 T1:SES group	-0.134	0.003	0.16	0.71	0.095	0.004	0.26	0.57	0.018	0.005	0.85	0.86	0.118	0.004	0.15	0.55
**Fornix**																
IL-10 T1	0.167	0.002	0.08	0.35	-0.067	0.003	0.40	0.93	-0.001	0.004	0.99	0.99	-0.101	0.003	0.20	0.86
SES group	-0.038	0.002	0.60	0.92	0.138	0.004	0.02	**0.04**	0.138	0.005	0.04	0.05	0.125	0.004	0.04	0.09
Sex	-0.068	0.002	0.33	0.43	-0.002	0.004	0.97	0.97	-0.020	0.005	0.77	0.78	0.015	0.004	0.80	0.91
NICU stay	0.033	0.005	0.67	0.98	0.032	0.008	0.64	0.85	0.047	0.011	0.53	0.84	0.019	0.008	0.77	0.79
GA at delivery	-0.061	0.001	0.48	0.61	0.028	0.001	0.70	0.74	-0.010	0.002	0.90	0.95	0.049	0.001	0.49	0.74
GA at T1	-0.064	0.000	0.36	0.64	0.071	0.001	0.23	0.90	0.064	0.001	0.33	0.81	0.074	0.001	0.21	0.76
PMA at scan	0.349	0.001	<0.001	**<0.001**	-0.612	0.001	<0.001	**<0.001**	-0.451	0.002	<0.001	**<0.001**	-0.633	0.001	<0.001	**<0.001**
IL-10 T1:SES group	-0.058	0.002	0.54	0.99	0.058	0.004	0.48	0.57	0.063	0.005	0.49	0.76	0.053	0.004	0.51	0.66

IL-10 Trimester 2

	FA				MD				AD				RD			
	β	SE	*p*	*q*	β	SE	*p*	*q*	β	SE	*p*	*q*	β	SE	*p*	*q*
**Corpus Callosum**																

IL-10 T2	-0.083	0.002	0.26	0.41	0.087	0.002	0.22	0.56	-0.059	0.003	0.50	0.91	0.110	0.003	0.11	0.33
SES group	0.030	0.004	0.63	0.81	0.007	0.004	0.91	0.91	0.082	0.005	0.27	0.35	-0.015	0.005	0.80	0.80
Sex	-0.089	0.004	0.14	0.63	-0.009	0.004	0.88	0.88	-0.101	0.004	0.16	0.28	0.042	0.005	0.46	0.68
NICU stay	-0.043	0.009	0.56	0.79	-0.061	0.009	0.41	0.92	0.018	0.010	0.84	0.95	-0.051	0.012	0.48	0.87
GA at delivery	0.098	0.001	0.22	0.46	-0.083	0.001	0.30	0.79	0.132	0.002	0.19	0.51	-0.134	0.002	0.09	0.40
GA at T2	0.077	0.001	0.21	0.64	-0.017	0.001	0.77	0.90	0.043	0.001	0.56	0.80	-0.045	0.001	0.44	0.93
PMA at scan	0.458	0.001	<0.001	**<0.001**	-0.565	0.001	<0.001	**<0.001**	-0.193	0.002	0.01	**0.01**	-0.547	0.002	<0.001	**<0.001**
IL-10 T2:SES group	-0.108	0.004	0.14	0.42	0.117	0.004	0.09	0.42	0.093	0.005	0.28	0.74	0.104	0.006	0.13	0.59
**Superior Cingulum Bundle**																
IL-10 T2	-0.030	0.002	0.71	0.71	0.039	0.003	0.61	0.99	0.040	0.004	0.63	0.91	0.033	0.004	0.67	0.86
SES group	-0.193	0.004	0.01	0.05	0.149	0.006	0.02	0.05	0.019	0.007	0.79	0.88	0.186	0.007	<0.01	**0.02**
Sex	-0.048	0.003	0.47	0.78	-0.029	0.005	0.64	0.72	-0.089	0.006	0.19	0.28	0.000	0.006	1.00	1.00
NICU stay	0.044	0.007	0.60	0.79	0.030	0.012	0.71	0.92	0.047	0.014	0.58	0.95	0.012	0.014	0.88	0.88
GA at delivery	0.065	0.001	0.48	0.54	0.078	0.002	0.37	0.79	0.117	0.002	0.21	0.51	0.044	0.002	0.61	0.78
GA at T2	-0.006	0.000	0.93	0.93	0.025	0.001	0.69	0.90	0.034	0.001	0.62	0.80	0.014	0.001	0.83	0.93
PMA at scan	0.394	0.001	<0.001	**<0.001**	-0.536	0.002	<0.001	**<0.001**	-0.381	0.002	<0.001	**<0.001**	-0.530	0.002	<0.001	**<0.001**
IL-10 T2:SES group	0.044	0.004	0.59	0.66	0.029	0.006	0.70	0.85	0.051	0.007	0.54	0.74	0.015	0.007	0.84	0.89
**Corticospinal Tract**																
IL-10 T2	-0.246	0.002	<0.001	**<0.01**	0.178	0.003	<0.01	**0.04**	0.033	0.003	0.63	0.91	0.200	0.004	<0.01	**0.01**
SES group	-0.031	0.004	0.59	0.81	0.129	0.005	0.01	**0.04**	0.184	0.005	<0.01	**0.01**	0.102	0.006	0.06	0.11
Sex	0.050	0.003	0.38	0.78	-0.058	0.005	0.25	0.53	-0.049	0.004	0.39	0.39	-0.057	0.006	0.28	0.62
NICU stay	-0.027	0.008	0.70	0.79	-0.015	0.012	0.81	0.92	0.009	0.010	0.90	0.95	-0.013	0.014	0.84	0.88
GA at delivery	0.066	0.001	0.39	0.51	-0.078	0.002	0.26	0.79	-0.022	0.001	0.77	0.77	-0.060	0.002	0.39	0.58
GA at T2	0.062	0.000	0.29	0.64	-0.010	0.001	0.85	0.90	0.022	0.001	0.71	0.80	-0.027	0.001	0.61	0.93
PMA at scan	0.547	0.001	<0.001	**<0.001**	-0.688	0.002	<0.001	**<0.001**	-0.634	0.002	<0.001	**<0.001**	-0.670	0.002	<0.001	**<0.001**
IL-10 T2:SES group	0.027	0.004	0.69	0.69	0.019	0.005	0.76	0.85	0.065	0.005	0.34	0.74	0.010	0.007	0.87	0.89
**Optic Radiation**																
IL-10 T2	-0.094	0.002	0.17	0.41	0.074	0.004	0.25	0.56	0.026	0.004	0.74	0.91	0.082	0.004	0.18	0.41
SES group	0.038	0.004	0.51	0.81	0.143	0.006	0.01	**0.04**	0.236	0.006	<0.001	**<0.01**	0.098	0.007	0.06	0.11
Sex	0.007	0.004	0.90	0.90	-0.164	0.006	<0.01	**0.01**	-0.238	0.006	<0.001	**<0.01**	-0.126	0.006	0.01	0.06
NICU stay	-0.096	0.008	0.18	0.79	0.065	0.013	0.33	0.92	0.016	0.013	0.84	0.95	0.079	0.014	0.22	0.87
GA at delivery	0.048	0.001	0.54	0.54	-0.128	0.002	0.08	0.69	-0.106	0.002	0.23	0.51	-0.119	0.002	0.09	0.40
GA at T2	0.076	0.001	0.19	0.64	0.028	0.001	0.61	0.90	0.085	0.001	0.20	0.43	0.001	0.001	0.99	0.99
PMA at scan	0.545	0.001	<0.001	**<0.001**	-0.606	0.002	<0.001	**<0.001**	-0.352	0.002	<0.001	**<0.001**	-0.633	0.002	<0.001	**<0.001**
IL-10 T2:SES group	-0.051	0.004	0.46	0.66	0.061	0.006	0.34	0.74	0.032	0.006	0.67	0.76	0.066	0.007	0.28	0.80
**Uncinate Fasciculus**																
IL-10 T2	-0.078	0.002	0.27	0.41	-0.006	0.003	0.94	0.99	-0.056	0.004	0.51	0.91	0.009	0.003	0.89	0.89
SES group	-0.041	0.003	0.49	0.81	0.103	0.005	0.11	0.19	0.086	0.006	0.23	0.35	0.092	0.006	0.12	0.19
Sex	-0.038	0.003	0.52	0.78	-0.045	0.005	0.47	0.61	-0.086	0.006	0.22	0.28	-0.025	0.005	0.66	0.85
NICU stay	-0.017	0.007	0.82	0.82	0.022	0.011	0.78	0.92	0.006	0.014	0.95	0.95	0.028	0.012	0.70	0.88
GA at delivery	0.140	0.001	0.08	0.29	0.043	0.002	0.61	0.79	0.162	0.002	0.09	0.51	-0.018	0.002	0.82	0.85
GA at T2	0.022	0.000	0.71	0.84	0.086	0.001	0.18	0.90	0.115	0.001	0.11	0.43	0.056	0.001	0.34	0.93
PMA at scan	0.530	0.001	<0.001	**<0.001**	-0.554	0.002	<0.001	**<0.001**	-0.239	0.002	<0.01	**<0.01**	-0.612	0.002	<0.001	**<0.001**

	β	SE	*p*	*q*	β	SE	*p*	*q*	β	SE	*p*	*q*	β	SE	*p*	*q*
IL-10 T2:SES group	-0.055	0.003	0.43	0.66	-0.010	0.006	0.90	0.90	-0.059	0.007	0.49	0.74	0.013	0.006	0.86	0.89
**Inferior Fronto-Occipital Fasciculus**																
IL-10 T2	-0.055	0.002	0.38	0.49	0.001	0.004	0.99	0.99	-0.048	0.003	0.55	0.91	0.016	0.005	0.80	0.89
SES group	-0.003	0.004	0.96	0.96	0.074	0.006	0.18	0.27	0.143	0.005	0.03	0.10	0.057	0.008	0.29	0.37
Sex	0.026	0.004	0.61	0.78	-0.123	0.006	0.02	0.07	-0.194	0.004	<0.01	**0.01**	-0.091	0.007	0.08	0.25
NICU stay	-0.031	0.008	0.64	0.79	0.048	0.013	0.47	0.92	0.038	0.010	0.65	0.95	0.046	0.016	0.49	0.87
GA at delivery	0.117	0.001	0.10	0.29	-0.043	0.002	0.56	0.79	0.075	0.002	0.40	0.59	-0.070	0.002	0.32	0.58
GA at T2	0.061	0.001	0.25	0.64	0.007	0.001	0.90	0.90	0.098	0.001	0.15	0.43	-0.016	0.001	0.76	0.93
PMA at scan	0.617	0.001	<0.001	**<0.001**	-0.640	0.002	<0.001	**<0.001**	-0.428	0.002	<0.001	**<0.001**	-0.647	0.003	<0.001	**<0.001**
IL-10 T2:SES group	-0.125	0.004	0.05	0.41	0.115	0.006	0.08	0.42	0.062	0.005	0.43	0.74	0.127	0.008	0.04	0.39
**Anterior Limb of Internal Capsule**																
IL-10 T2	-0.077	0.002	0.22	0.41	0.017	0.004	0.80	0.99	-0.017	0.004	0.84	0.91	0.029	0.005	0.66	0.86
SES group	0.014	0.003	0.79	0.89	0.055	0.007	0.36	0.41	0.087	0.006	0.21	0.35	0.041	0.008	0.47	0.53
Sex	0.020	0.003	0.69	0.78	-0.054	0.007	0.35	0.53	-0.061	0.006	0.36	0.39	-0.048	0.008	0.39	0.68
NICU stay	-0.046	0.007	0.47	0.79	0.048	0.016	0.51	0.92	0.030	0.013	0.72	0.95	0.051	0.018	0.47	0.87
GA at delivery	0.170	0.001	0.02	0.15	-0.041	0.002	0.61	0.79	0.067	0.002	0.46	0.59	-0.074	0.003	0.33	0.58
GA at T2	-0.021	0.000	0.69	0.84	0.057	0.001	0.33	0.90	0.081	0.001	0.24	0.43	0.048	0.001	0.40	0.93
PMA at scan	0.610	0.001	<0.001	**<0.001**	-0.591	0.002	<0.001	**<0.001**	-0.435	0.002	<0.001	**<0.001**	-0.616	0.003	<0.001	**<0.001**
IL-10 T2:SES group	-0.050	0.003	0.42	0.66	0.059	0.008	0.40	0.74	0.045	0.007	0.58	0.74	0.060	0.009	0.37	0.80
**Inferior Cingulum Bundle**																
IL-10 T2	-0.186	0.002	0.02	0.08	0.177	0.002	0.01	**0.04**	0.049	0.003	0.55	0.91	0.212	0.002	<0.01	**0.01**
SES group	-0.143	0.003	0.03	0.10	0.235	0.003	<0.001	**<0.001**	0.139	0.005	0.05	0.11	0.220	0.004	<0.001	**<0.01**
Sex	-0.035	0.003	0.59	0.78	-0.189	0.003	<0.01	**0.01**	-0.205	0.004	<0.01	**0.01**	-0.155	0.003	<0.01	**0.03**
NICU stay	-0.073	0.006	0.37	0.79	0.007	0.007	0.92	0.92	-0.065	0.010	0.45	0.95	0.050	0.008	0.46	0.87
GA at delivery	-0.100	0.001	0.26	0.46	0.022	0.001	0.77	0.87	-0.077	0.001	0.41	0.59	0.076	0.001	0.29	0.58
GA at T2	0.021	0.000	0.75	0.84	0.063	0.000	0.26	0.90	0.081	0.001	0.24	0.43	0.060	0.000	0.27	0.93
PMA at scan	0.469	0.001	<0.001	**<0.001**	-0.644	0.001	<0.001	**<0.001**	-0.321	0.002	<0.001	**<0.001**	-0.683	0.001	<0.001	**<0.001**
IL-10 T2:SES group	0.046	0.003	0.55	0.66	-0.032	0.003	0.62	0.85	-0.004	0.005	0.96	0.96	-0.049	0.004	0.45	0.80
**Fornix**																
IL-10 T2	0.044	0.001	0.58	0.65	-0.018	0.002	0.79	0.99	0.009	0.003	0.91	0.91	-0.031	0.002	0.65	0.86
SES group	-0.173	0.002	0.01	0.05	0.072	0.004	0.22	0.29	-0.004	0.005	0.96	0.96	0.111	0.004	0.06	0.11
Sex	-0.116	0.002	0.08	0.63	-0.058	0.003	0.31	0.53	-0.112	0.005	0.08	0.17	-0.014	0.004	0.80	0.90
NICU stay	0.045	0.005	0.58	0.79	-0.035	0.008	0.62	0.92	-0.022	0.010	0.78	0.95	-0.039	0.008	0.58	0.87
GA at delivery	0.081	0.001	0.36	0.51	0.012	0.001	0.88	0.88	0.055	0.002	0.53	0.59	-0.014	0.001	0.85	0.85
GA at T2	0.036	0.000	0.60	0.84	-0.019	0.000	0.74	0.90	-0.008	0.001	0.90	0.90	-0.024	0.000	0.68	0.93
PMA at scan	0.381	0.001	<0.001	**<0.001**	-0.640	0.001	<0.001	**<0.001**	-0.474	0.002	<0.001	**<0.001**	-0.653	0.001	<0.001	**<0.001**
IL-10 T2:SES group	0.118	0.002	0.14	0.42	0.056	0.004	0.41	0.74	0.173	0.005	0.02	0.21	-0.009	0.004	0.89	0.89

IL-10 Trimester 3

	FA				MD				AD				RD			
	**β**	**SE**	***p***	***q***	**β**	**SE**	***p***	***q***	**β**	**SE**	***p***	***q***	**β**	**SE**	***p***	***q***
**Corpus Callosum**																
IL-10 T3	-0.031	0.002	0.65	0.65	-0.010	0.002	0.87	0.90	-0.124	0.002	0.12	0.35	0.007	0.003	0.91	0.91
SES group	0.090	0.004	0.13	0.28	0.008	0.004	0.89	0.89	0.175	0.004	0.01	**0.01**	-0.035	0.005	0.53	0.53

Sex	-0.121	0.003	0.02	0.22	0.006	0.003	0.92	0.94	-0.071	0.004	0.26	0.44	0.065	0.005	0.20	0.60
NICU stay	-0.044	0.009	0.48	0.73	-0.034	0.009	0.59	0.78	-0.006	0.010	0.93	0.93	-0.012	0.012	0.84	0.92
GA at delivery	-0.016	0.001	0.82	0.82	0.013	0.001	0.85	0.96	0.056	0.002	0.50	0.90	-0.005	0.002	0.94	0.97
GA at T3	0.039	0.001	0.49	0.70	-0.051	0.001	0.36	0.62	-0.035	0.001	0.61	0.73	-0.059	0.001	0.28	0.80
PMA at scan	0.469	0.001	<0.001	**<0.001**	-0.567	0.001	<0.001	**<0.001**	-0.179	0.002	0.01	**0.01**	-0.563	0.002	<0.001	**<0.001**
IL-10 T3:SES group	-0.069	0.003	0.31	0.55	0.095	0.004	0.15	0.52	0.102	0.004	0.20	0.71	0.084	0.005	0.19	0.53
**Superior Cingulum Bundle**																
IL-10 T3	-0.184	0.002	0.01	0.06	0.075	0.003	0.28	0.47	-0.032	0.003	0.66	0.66	0.127	0.003	0.07	0.36
SES group	-0.131	0.003	0.04	0.18	0.157	0.005	0.01	**0.01**	0.068	0.006	0.28	0.28	0.172	0.006	<0.01	**0.02**
Sex	-0.076	0.003	0.19	0.47	-0.024	0.005	0.66	0.94	-0.098	0.005	0.09	0.21	0.011	0.005	0.84	0.94
NICU stay	-0.044	0.008	0.53	0.73	-0.011	0.012	0.86	0.97	-0.051	0.014	0.46	0.59	0.007	0.014	0.92	0.92
GA at delivery	0.042	0.001	0.59	0.76	0.051	0.002	0.49	0.79	0.078	0.002	0.32	0.71	0.028	0.002	0.70	0.90
GA at T3	-0.039	0.001	0.54	0.70	-0.017	0.001	0.77	0.87	-0.046	0.001	0.46	0.73	-0.002	0.001	0.97	0.97
PMA at scan	0.306	0.001	<0.001	**<0.001**	-0.512	0.002	<0.001	**<0.001**	-0.404	0.002	<0.001	**<0.001**	-0.484	0.002	<0.001	**<0.001**
IL-10 T3:SES group	0.136	0.003	0.07	0.46	-0.012	0.005	0.86	0.96	0.087	0.006	0.24	0.71	-0.065	0.006	0.35	0.53
**Corticospinal Tract**																
IL-10 T3	-0.162	0.002	0.01	0.06	0.061	0.003	0.28	0.47	-0.044	0.002	0.49	0.66	0.089	0.003	0.12	0.36
SES group	0.010	0.003	0.85	0.91	0.150	0.005	<0.01	**0.01**	0.270	0.004	<0.001	**<0.001**	0.101	0.006	0.04	0.05
Sex	0.009	0.003	0.86	0.97	-0.040	0.004	0.38	0.68	-0.051	0.004	0.31	0.44	-0.030	0.005	0.51	0.92
NICU stay	-0.064	0.008	0.28	0.73	0.000	0.011	1.00	1.00	-0.078	0.009	0.19	0.43	0.016	0.014	0.76	0.92
GA at delivery	-0.016	0.001	0.81	0.82	-0.038	0.002	0.53	0.79	-0.074	0.002	0.27	0.71	0.002	0.002	0.97	0.97
GA at T3	0.075	0.001	0.16	0.70	-0.080	0.001	0.10	0.62	-0.056	0.001	0.30	0.73	-0.080	0.001	0.10	0.80
PMA at scan	0.523	0.001	<0.001	**<0.001**	-0.656	0.002	<0.001	**<0.001**	-0.562	0.001	<0.001	**<0.001**	-0.644	0.002	<0.001	**<0.001**
IL-10 T3:SES group	-0.102	0.003	0.10	0.46	0.075	0.004	0.19	0.52	0.010	0.004	0.88	0.96	0.094	0.005	0.11	0.53
**Optic Radiation**																
IL-10 T3	-0.048	0.002	0.46	0.65	0.087	0.003	0.16	0.47	0.082	0.003	0.25	0.45	0.083	0.004	0.18	0.39
SES group	0.092	0.004	0.11	0.28	0.195	0.006	<0.001	**<0.01**	0.343	0.006	<0.001	**<0.001**	0.122	0.007	0.02	**0.04**
Sex	0.000	0.003	0.99	0.99	-0.104	0.005	0.04	0.20	-0.163	0.005	<0.01	**0.04**	-0.077	0.006	0.11	0.51
NICU stay	-0.098	0.009	0.11	0.51	0.039	0.013	0.50	0.78	-0.031	0.013	0.64	0.72	0.061	0.015	0.29	0.92
GA at delivery	-0.043	0.001	0.54	0.76	-0.091	0.002	0.17	0.79	-0.149	0.002	0.05	0.43	-0.055	0.002	0.39	0.83
GA at T3	0.024	0.001	0.67	0.76	-0.029	0.001	0.59	0.75	-0.008	0.001	0.89	0.89	-0.033	0.001	0.53	0.80
PMA at scan	0.543	0.001	<0.001	**<0.001**	-0.546	0.002	<0.001	**<0.001**	-0.249	0.002	<0.001	**<0.001**	-0.590	0.002	<0.001	**<0.001**
IL-10 T3:SES group	-0.003	0.003	0.97	0.99	-0.011	0.005	0.86	0.96	-0.019	0.005	0.78	0.96	-0.006	0.006	0.92	0.92
**Uncinate Fasciculus**																
IL-10 T3	-0.042	0.002	0.53	0.65	-0.085	0.003	0.20	0.47	-0.134	0.003	0.06	0.29	-0.049	0.003	0.45	0.67
SES group	-0.052	0.003	0.37	0.67	0.152	0.005	0.01	**0.01**	0.127	0.006	0.04	0.05	0.138	0.005	0.01	**0.03**
Sex	-0.067	0.003	0.21	0.47	-0.048	0.004	0.36	0.68	-0.102	0.005	0.08	0.21	-0.018	0.005	0.72	0.93
NICU stay	-0.035	0.007	0.58	0.73	-0.157	0.011	0.01	0.12	-0.197	0.014	<0.01	**0.04**	-0.110	0.012	0.07	0.63
GA at delivery	0.145	0.001	0.04	0.38	0.001	0.002	0.99	0.99	0.101	0.002	0.19	0.71	-0.050	0.002	0.46	0.83
GA at T3	-0.014	0.001	0.81	0.81	-0.047	0.001	0.42	0.62	-0.052	0.001	0.40	0.73	-0.036	0.001	0.52	0.80
PMA at scan	0.451	0.001	<0.001	**<0.001**	-0.539	0.002	<0.001	**<0.001**	-0.282	0.002	<0.001	**<0.001**	-0.581	0.002	<0.001	**<0.001**
IL-10 T3:SES group	-0.042	0.003	0.53	0.79	-0.079	0.004	0.24	0.52	-0.109	0.005	0.14	0.71	-0.055	0.005	0.40	0.53
**Inferior Fronto-Occipital Fasciculus**																

IL-10 T3	-0.073	0.002	0.22	0.49	-0.008	0.003	0.90	0.90	-0.092	0.002	0.19	0.43	0.017	0.004	0.77	0.86
SES group	-0.007	0.003	0.90	0.91	0.147	0.005	0.01	**0.01**	0.258	0.004	<0.001	**<0.001**	0.108	0.007	0.03	0.05
Sex	-0.014	0.003	0.76	0.97	-0.076	0.005	0.11	0.33	-0.133	0.004	0.02	0.08	-0.048	0.006	0.31	0.69
NICU stay	-0.040	0.008	0.47	0.73	-0.029	0.013	0.60	0.78	-0.077	0.010	0.25	0.45	-0.008	0.016	0.89	0.92
GA at delivery	0.075	0.001	0.24	0.71	-0.055	0.002	0.39	0.79	0.008	0.002	0.91	0.91	-0.064	0.003	0.31	0.83
GA at T3	0.039	0.001	0.44	0.70	-0.044	0.001	0.40	0.62	-0.028	0.001	0.64	0.73	-0.046	0.001	0.36	0.80
PMA at scan	0.588	0.001	<0.001	**<0.001**	-0.625	0.002	<0.001	**<0.001**	-0.406	0.002	<0.001	**<0.001**	-0.634	0.002	<0.001	**<0.001**
IL-10 T3:SES group	-0.079	0.003	0.19	0.51	0.065	0.005	0.28	0.52	0.031	0.004	0.66	0.96	0.074	0.006	0.21	0.53
**Anterior Limb of Internal Capsule**																
IL-10 T3	-0.040	0.002	0.49	0.65	-0.062	0.004	0.32	0.47	-0.133	0.003	0.06	0.29	-0.038	0.004	0.54	0.69
SES group	-0.006	0.003	0.91	0.91	0.137	0.007	0.01	**0.02**	0.188	0.006	<0.01	**0.01**	0.111	0.008	0.04	0.05
Sex	-0.040	0.003	0.39	0.69	-0.009	0.006	0.86	0.94	-0.024	0.005	0.67	0.67	0.001	0.007	0.99	0.99
NICU stay	-0.025	0.007	0.65	0.73	-0.052	0.016	0.38	0.78	-0.101	0.013	0.13	0.39	-0.032	0.018	0.58	0.92
GA at delivery	0.102	0.001	0.11	0.47	-0.045	0.003	0.50	0.79	0.028	0.002	0.71	0.91	-0.061	0.003	0.35	0.83
GA at T3	0.046	0.001	0.36	0.70	-0.065	0.001	0.23	0.62	-0.059	0.001	0.33	0.73	-0.063	0.001	0.23	0.80
PMA at scan	0.589	0.001	<0.001	**<0.001**	-0.596	0.002	<0.001	**<0.001**	-0.460	0.002	<0.001	**<0.001**	-0.615	0.003	<0.001	**<0.001**
IL-10 T3:SES group	-0.071	0.003	0.23	0.51	0.067	0.006	0.29	0.52	0.054	0.005	0.45	0.96	0.069	0.007	0.26	0.53
**Inferior Cingulum Bundle**																
IL-10 T3	-0.144	0.001	0.05	0.15	0.063	0.002	0.32	0.47	-0.034	0.002	0.65	0.66	0.107	0.002	0.09	0.36
SES group	-0.045	0.003	0.47	0.71	0.220	0.003	<0.001	**<0.01**	0.191	0.004	<0.01	**0.01**	0.170	0.003	<0.01	**0.02**
Sex	0.043	0.002	0.46	0.69	-0.101	0.003	0.04	0.20	-0.054	0.004	0.36	0.44	-0.107	0.003	0.03	0.30
NICU stay	-0.126	0.006	0.07	0.51	-0.030	0.007	0.61	0.78	-0.118	0.009	0.09	0.39	0.043	0.008	0.47	0.92
GA at delivery	-0.070	0.001	0.37	0.74	0.077	0.001	0.25	0.79	0.015	0.002	0.85	0.91	0.107	0.001	0.11	0.83
GA at T3	-0.063	0.000	0.32	0.70	-0.044	0.001	0.42	0.62	-0.093	0.001	0.15	0.73	-0.002	0.001	0.97	0.97
PMA at scan	0.364	0.001	<0.001	**<0.001**	-0.601	0.001	<0.001	**<0.001**	-0.339	0.001	<0.001	**<0.001**	-0.612	0.001	<0.001	**<0.001**
IL-10 T3:SES group	0.014	0.002	0.85	0.99	-0.003	0.003	0.96	0.96	0.003	0.004	0.96	0.96	-0.027	0.003	0.68	0.76
**Fornix**																
IL-10 T3	-0.033	0.001	0.65	0.65	0.057	0.002	0.37	0.47	0.034	0.003	0.63	0.66	0.061	0.002	0.32	0.58
SES group	-0.138	0.002	0.03	0.18	0.119	0.003	0.03	**0.03**	0.066	0.005	0.28	0.28	0.140	0.003	0.01	**0.03**
Sex	-0.090	0.002	0.12	0.47	-0.004	0.003	0.94	0.94	-0.048	0.004	0.39	0.44	0.025	0.003	0.61	0.92
NICU stay	-0.009	0.005	0.89	0.89	-0.054	0.008	0.37	0.78	-0.068	0.011	0.31	0.46	-0.040	0.008	0.50	0.92
GA at delivery	0.064	0.001	0.41	0.74	-0.014	0.001	0.84	0.96	0.021	0.002	0.79	0.91	-0.030	0.001	0.65	0.90
GA at T3	-0.046	0.000	0.47	0.70	0.005	0.001	0.93	0.93	-0.033	0.001	0.59	0.73	0.021	0.001	0.69	0.88
PMA at scan	0.348	0.001	<0.001	**<0.001**	-0.613	0.001	<0.001	**<0.001**	-0.465	0.002	<0.001	**<0.001**	-0.622	0.001	<0.001	**<0.001**
IL-10 T3:SES group	0.001	0.002	0.99	0.99	-0.049	0.003	0.44	0.67	-0.022	0.004	0.76	0.96	-0.051	0.003	0.41	0.53

TNF-α Trimester 1

	FA				MD				AD				RD			
	**β**	**SE**	***p***	***q***	**β**	**SE**	***p***	***q***	**β**	**SE**	***p***	***q***	**β**	**SE**	***p***	***q***
**Corpus Callosum**																
TNF-α T1	0.119	0.003	0.15	0.31	-0.144	0.003	0.08	0.10	-0.058	0.003	0.55	0.62	-0.144	0.004	0.07	0.11
SES group	0.152	0.004	0.02	0.21	-0.010	0.004	0.87	0.87	0.214	0.005	0.01	**0.01**	-0.089	0.005	0.16	0.16
Sex	-0.152	0.004	0.02	0.14	0.082	0.004	0.18	0.70	-0.077	0.005	0.29	0.52	0.128	0.005	0.03	0.30
NICU stay	0.010	0.009	0.89	0.96	-0.059	0.009	0.41	0.75	0.061	0.010	0.46	0.71	-0.065	0.012	0.35	0.80

GA at delivery	-0.054	0.002	0.49	0.63	0.046	0.002	0.56	0.88	-0.061	0.002	0.49	0.97	0.049	0.002	0.52	0.72
GA at T1	-0.068	0.001	0.27	0.77	0.093	0.001	0.13	0.97	0.033	0.001	0.65	0.76	0.093	0.001	0.12	0.96
PMA at scan	0.444	0.002	<0.001	**<0.001**	-0.527	0.001	<0.001	**<0.001**	-0.087	0.002	0.29	0.29	-0.525	0.002	<0.001	**<0.001**
TNF-α T1:SES group	-0.211	0.005	0.01	**0.01**	0.191	0.005	0.02	**0.03**	0.045	0.005	0.63	0.70	0.200	0.006	0.01	**0.03**
**Superior Cingulum Bundle**																
TNF-α T1	0.120	0.003	0.20	0.31	-0.241	0.004	<0.01	**0.01**	-0.153	0.005	0.10	0.17	-0.233	0.005	0.01	**0.02**
SES group	-0.141	0.004	0.06	0.26	0.194	0.006	<0.01	**0.01**	0.090	0.007	0.22	0.22	0.209	0.007	<0.01	**0.02**
Sex	-0.115	0.004	0.10	0.29	-0.041	0.005	0.52	0.77	-0.135	0.006	0.05	0.11	0.005	0.006	0.94	0.94
NICU stay	0.012	0.008	0.88	0.96	0.059	0.012	0.41	0.75	0.056	0.015	0.48	0.71	0.045	0.014	0.54	0.80
GA at delivery	0.131	0.001	0.13	0.29	-0.012	0.002	0.88	0.88	0.081	0.003	0.35	0.97	-0.060	0.002	0.45	0.72
GA at T1	-0.010	0.001	0.88	0.95	-0.030	0.001	0.63	0.97	-0.052	0.001	0.45	0.76	-0.020	0.001	0.75	0.96
PMA at scan	0.289	0.001	<0.001	**<0.001**	-0.483	0.002	<0.001	**<0.001**	-0.378	0.003	<0.001	**<0.001**	-0.461	0.002	<0.001	**<0.001**
TNF-α T1:SES group	-0.126	0.004	0.16	0.37	0.258	0.006	<0.01	**0.01**	0.191	0.008	0.03	0.08	0.242	0.007	<0.01	**0.02**
**Corticospinal Tract**																
TNF-α T1	0.132	0.003	0.12	0.31	-0.170	0.004	0.02	**0.04**	-0.118	0.003	0.13	0.17	-0.173	0.004	0.02	0.05
SES group	0.059	0.004	0.38	1.00	0.142	0.005	0.02	**0.02**	0.275	0.005	<0.001	**<0.001**	0.084	0.006	0.16	0.16
Sex	-0.088	0.003	0.16	0.31	0.022	0.005	0.69	0.89	-0.033	0.004	0.57	0.60	0.041	0.006	0.47	0.94
NICU stay	0.089	0.008	0.22	0.96	0.020	0.011	0.76	0.75	0.089	0.010	0.19	0.71	-0.006	0.013	0.92	0.92
GA at delivery	-0.041	0.001	0.60	0.64	0.027	0.002	0.69	0.88	0.002	0.002	0.97	0.97	0.033	0.002	0.64	0.72
GA at T1	0.033	0.001	0.60	0.77	0.009	0.001	0.87	0.97	0.049	0.001	0.40	0.76	-0.006	0.001	0.92	0.96
PMA at scan	0.511	0.001	<0.001	**<0.001**	-0.666	0.002	<0.001	**<0.001**	-0.562	0.002	<0.001	**<0.001**	-0.650	0.002	<0.001	**<0.001**
TNF-α T1:SES group	-0.118	0.004	0.15	0.37	0.164	0.006	0.02	**0.04**	0.126	0.005	0.10	0.15	0.164	0.007	0.03	0.05
**Optic Radiation**																
TNF-α T1	0.073	0.003	0.38	0.42	-0.063	0.005	0.44	0.44	-0.004	0.005	0.96	0.96	-0.071	0.005	0.37	0.42
SES group	0.046	0.004	0.48	1.00	0.195	0.007	<0.01	**0.01**	0.296	0.007	<0.01	**<0.01**	0.141	0.008	0.03	0.05
Sex	-0.084	0.004	0.17	0.31	-0.071	0.006	0.24	0.70	-0.179	0.006	0.01	0.06	-0.031	0.007	0.60	0.94
NICU stay	-0.096	0.008	0.18	0.96	0.047	0.014	0.50	0.75	-0.001	0.014	0.99	0.99	0.062	0.016	0.36	0.80
GA at delivery	-0.122	0.001	0.12	0.29	-0.016	0.003	0.83	0.88	-0.104	0.002	0.21	0.96	0.019	0.003	0.80	0.80
GA at T1	-0.004	0.001	0.95	0.95	-0.002	0.001	0.98	0.98	0.020	0.001	0.76	0.76	0.004	0.002	0.95	0.96
PMA at scan	0.560	0.001	<0.001	**<0.001**	-0.572	0.002	<0.001	**<0.001**	-0.312	0.002	<0.001	**<0.001**	-0.613	0.003	<0.001	**<0.001**
TNF-α T1:SES group	-0.016	0.004	0.84	0.95	0.049	0.008	0.53	0.53	0.034	0.007	0.70	0.70	0.045	0.009	0.56	0.56
**Uncinate Fasciculus**																
TNF-α T1	-0.074	0.002	0.37	0.42	-0.069	0.004	0.41	0.44	-0.141	0.005	0.13	0.17	-0.031	0.004	0.70	0.70
SES group	0.016	0.003	0.82	1.00	0.159	0.006	0.02	**0.02**	0.193	0.007	0.01	**0.01**	0.119	0.006	0.07	0.08
Sex	-0.129	0.003	0.04	0.18	-0.064	0.005	0.31	0.70	-0.164	0.006	0.02	0.06	-0.013	0.006	0.83	0.94
NICU stay	0.016	0.007	0.83	0.96	0.020	0.012	0.79	0.75	0.017	0.014	0.84	0.94	0.021	0.013	0.77	0.86
GA at delivery	0.092	0.001	0.24	0.43	0.105	0.002	0.18	0.88	0.178	0.002	0.04	0.38	0.053	0.002	0.49	0.72
GA at T1	-0.089	0.001	0.15	0.77	0.019	0.001	0.76	0.97	-0.035	0.001	0.61	0.76	0.032	0.001	0.59	0.96
PMA at scan	0.453	0.001	<0.001	**<0.001**	-0.572	0.002	<0.001	**<0.001**	-0.313	0.002	<0.001	**<0.001**	-0.610	0.002	<0.001	**<0.001**
TNF-α T1:SES group	-0.016	0.004	0.84	0.95	0.067	0.006	0.41	0.46	0.076	0.007	0.41	0.52	0.057	0.007	0.46	0.52
**Inferior Fronto-Occipital Fasciculus**																
TNF-α T1	0.099	0.003	0.19	0.31	-0.172	0.004	0.02	**0.04**	-0.165	0.003	0.05	0.12	-0.157	0.005	0.03	0.06
SES group	0.000	0.004	1.00	1.00	0.175	0.006	<0.01	**0.01**	0.316	0.005	<0.001	**<0.001**	0.126	0.007	0.03	0.05

Sex	-0.038	0.004	0.50	0.56	-0.078	0.005	0.15	0.70	-0.159	0.004	0.01	0.06	-0.043	0.007	0.43	0.94
NICU stay	-0.024	0.008	0.72	0.96	0.050	0.012	0.42	0.75	0.062	0.010	0.39	0.71	0.042	0.015	0.50	0.80
GA at delivery	0.033	0.001	0.64	0.63	-0.031	0.002	0.65	0.88	-0.003	0.002	0.97	0.97	-0.035	0.003	0.61	0.72
GA at T1	-0.047	0.001	0.40	0.77	0.018	0.001	0.74	0.97	-0.019	0.001	0.76	0.76	0.036	0.002	0.50	0.96
PMA at scan	0.623	0.001	<0.001	**<0.001**	-0.650	0.002	<0.001	**<0.001**	-0.418	0.002	<0.001	**<0.001**	-0.660	0.003	<0.001	**<0.001**
TNF-α T1:SES group	-0.077	0.004	0.29	0.53	0.175	0.007	0.01	**0.03**	0.209	0.005	0.01	0.05	0.150	0.008	0.03	0.05
**Anterior Limb of Internal Capsule**																
TNF-α T1	0.140	0.002	0.06	0.31	-0.222	0.005	<0.01	**0.01**	-0.212	0.004	0.02	0.06	-0.214	0.006	0.01	**0.02**
SES group	-0.005	0.003	0.93	1.00	0.149	0.007	0.02	**0.02**	0.203	0.006	<0.01	**0.01**	0.122	0.008	0.05	0.07
Sex	-0.056	0.003	0.32	0.41	-0.005	0.007	0.93	0.93	-0.035	0.006	0.60	0.60	0.008	0.008	0.89	0.94
NICU stay	0.004	0.007	0.96	0.96	0.059	0.015	0.38	0.75	0.080	0.013	0.30	0.71	0.049	0.017	0.45	0.80
GA at delivery	0.109	0.001	0.12	0.29	-0.030	0.003	0.68	0.88	0.034	0.002	0.68	0.97	-0.050	0.003	0.48	0.72
GA at T1	-0.032	0.001	0.56	0.77	0.041	0.002	0.48	0.97	0.033	0.001	0.62	0.76	0.041	0.002	0.47	0.96
PMA at scan	0.584	0.001	<0.001	**<0.001**	-0.560	0.003	<0.001	**<0.001**	-0.382	0.002	<0.001	**<0.001**	-0.591	0.003	<0.001	**<0.001**
TNF-α T1:SES group	-0.122	0.004	0.10	0.37	0.237	0.008	<0.01	**0.01**	0.252	0.007	<0.01	**0.04**	0.220	0.009	<0.01	**0.02**
**Inferior Cingulum Bundle**																
TNF-α T1	-0.115	0.002	0.21	0.31	-0.146	0.002	0.07	0.10	-0.216	0.003	0.02	0.06	-0.076	0.003	0.34	0.42
SES group	0.007	0.003	0.93	1.00	0.207	0.003	<0.01	**0.01**	0.198	0.005	0.01	**0.01**	0.171	0.004	0.01	**0.04**
Sex	-0.014	0.003	0.84	0.84	-0.047	0.003	0.44	0.77	-0.049	0.004	0.48	0.60	-0.035	0.003	0.56	0.94
NICU stay	0.073	0.006	0.36	0.96	-0.011	0.007	0.87	0.75	0.029	0.010	0.72	0.92	-0.032	0.008	0.65	0.83
GA at delivery	-0.153	0.001	0.08	0.29	0.086	0.001	0.26	0.88	-0.025	0.002	0.77	0.97	0.129	0.001	0.08	0.72
GA at T1	-0.065	0.001	0.34	0.77	-0.024	0.001	0.69	0.97	-0.059	0.001	0.39	0.76	0.003	0.001	0.96	0.96
PMA at scan	0.397	0.001	<0.001	**<0.001**	-0.581	0.001	<0.001	**<0.001**	-0.312	0.002	<0.001	**<0.001**	-0.617	0.001	<0.001	**<0.001**
TNF-α T1:SES group	-0.005	0.003	0.95	0.95	0.209	0.004	0.01	**0.03**	0.214	0.005	0.02	0.05	0.165	0.004	0.04	0.05
**Fornix**																
TNF-α T1	0.038	0.002	0.69	0.69	-0.248	0.003	<0.01	**0.01**	-0.239	0.004	0.01	0.05	-0.221	0.003	<0.01	**0.02**
SES group	-0.023	0.002	0.76	1.00	0.179	0.004	<0.01	**0.01**	0.186	0.005	0.01	**0.01**	0.157	0.004	0.01	**0.04**
Sex	-0.084	0.002	0.24	0.36	-0.006	0.003	0.92	0.93	-0.035	0.005	0.59	0.60	0.018	0.003	0.76	0.94
NICU stay	0.009	0.005	0.91	0.96	0.056	0.008	0.40	0.75	0.055	0.011	0.46	0.71	0.049	0.008	0.46	0.80
GA at delivery	-0.087	0.001	0.33	0.49	0.038	0.001	0.60	0.88	-0.015	0.002	0.85	0.97	0.067	0.001	0.36	0.72
GA at T1	-0.044	0.000	0.53	0.77	0.060	0.001	0.30	0.97	0.060	0.001	0.35	0.76	0.059	0.001	0.30	0.96
PMA at scan	0.324	0.001	<0.001	**<0.001**	-0.608	0.001	<0.001	**<0.001**	-0.458	0.002	<0.001	**<0.001**	-0.625	0.001	<0.001	**<0.001**
TNF-α T1:SES group	-0.058	0.003	0.53	0.80	0.159	0.004	0.04	0.05	0.155	0.006	0.07	0.12	0.141	0.004	0.06	0.08
**TNF-α Trimester 2**																
	**FA**				**MD**				**AD**				**RD**			
	**β**	**SE**	***p***	***q***	**β**	**SE**	***p***	***q***	**β**	**SE**	***p***	***q***	**β**	**SE**	***p***	***q***
**Corpus Callosum**																
TNF-α T2	-0.038	0.003	0.60	0.78	0.018	0.003	0.80	0.94	-0.074	0.003	0.40	0.72	0.047	0.003	0.50	0.89
SES group	0.044	0.004	0.48	0.72	-0.010	0.004	0.87	0.87	0.097	0.005	0.19	0.34	-0.029	0.006	0.62	0.62
Sex	-0.085	0.004	0.17	0.51	-0.018	0.004	0.77	0.95	-0.103	0.004	0.16	0.29	0.040	0.005	0.50	0.83
NICU stay	-0.078	0.009	0.29	0.53	-0.024	0.010	0.75	0.86	0.024	0.010	0.79	0.97	-0.001	0.013	0.99	0.99
GA at delivery	0.063	0.001	0.43	0.64	-0.051	0.001	0.54	0.81	0.137	0.001	0.17	0.54	-0.089	0.002	0.27	0.81
GA at T2	0.043	0.001	0.50	0.80	-0.004	0.001	0.94	0.99	0.049	0.001	0.51	0.60	-0.008	0.001	0.89	1.00

PMA at scan	0.472	0.001	<0.001	**<0.001**	-0.566	0.001	<0.001	**<0.001**	-0.205	0.002	<0.001	**<0.001**	-0.559	0.002	<0.001	**<0.001**
TNF-α T2:SES group	-0.115	0.004	0.12	0.56	0.080	0.005	0.26	0.74	0.050	0.005	0.57	0.85	0.072	0.006	0.30	0.90
**Superior Cingulum Bundle**																
TNF-α T2	-0.010	0.002	0.90	0.90	-0.005	0.003	0.94	0.94	-0.001	0.004	0.99	0.99	0.000	0.004	0.99	0.99
SES group	-0.197	0.004	<0.01	**0.04**	0.134	0.006	0.04	0.11	-0.004	0.007	0.96	0.96	0.177	0.006	0.01	**0.03**
Sex	-0.078	0.003	0.24	0.51	-0.004	0.005	0.95	0.95	-0.079	0.006	0.25	0.38	0.029	0.006	0.64	0.83
NICU stay	0.019	0.008	0.82	0.82	0.053	0.012	0.50	0.86	0.061	0.014	0.48	0.97	0.037	0.014	0.64	0.92
GA at delivery	0.053	0.001	0.56	0.72	0.088	0.002	0.30	0.81	0.125	0.002	0.18	0.54	0.055	0.002	0.51	0.82
GA at T2	-0.022	0.000	0.75	0.85	0.042	0.001	0.52	0.93	0.052	0.001	0.46	0.60	0.029	0.001	0.65	1.00
PMA at scan	0.410	0.001	<0.001	**<0.001**	-0.546	0.002	<0.001	**<0.001**	-0.385	0.002	<0.001	**<0.001**	-0.540	0.002	<0.001	**<0.001**
TNF-α T2:SES group	0.037	0.004	0.64	0.72	0.063	0.006	0.40	0.74	0.087	0.007	0.29	0.65	0.038	0.007	0.61	1.00
**Corticospinal Tract**																
TNF-α T2	-0.127	0.002	0.08	0.17	0.133	0.003	0.03	0.30	0.063	0.003	0.36	0.72	0.132	0.004	0.04	0.28
SES group	-0.008	0.004	0.89	0.97	0.104	0.005	0.05	0.11	0.170	0.004	<0.01	**0.01**	0.076	0.007	0.16	0.24
Sex	0.023	0.004	0.70	0.91	-0.027	0.005	0.60	0.89	-0.026	0.004	0.65	0.65	-0.026	0.006	0.62	0.83
NICU stay	-0.068	0.008	0.36	0.54	0.019	0.012	0.76	0.86	0.031	0.010	0.66	0.97	0.022	0.015	0.74	0.92
GA at delivery	0.035	0.001	0.66	0.74	-0.050	0.002	0.47	0.81	-0.006	0.001	0.94	0.94	-0.031	0.002	0.66	0.85
GA at T2	0.055	0.001	0.37	0.80	-0.001	0.001	0.99	0.99	0.036	0.001	0.53	0.60	-0.019	0.001	0.73	1.00
PMA at scan	0.555	0.001	<0.001	**<0.001**	-0.700	0.002	<0.001	**<0.001**	-0.651	0.001	<0.001	**<0.001**	-0.680	0.002	<0.001	**<0.001**
TNF-α T2:SES group	0.041	0.004	0.56	0.72	-0.042	0.006	0.49	0.74	-0.016	0.005	0.81	0.89	-0.039	0.007	0.55	1.00
**Optic Radiation**																
TNF-α T2	-0.159	0.002	0.02	0.07	0.094	0.004	0.14	0.64	0.034	0.004	0.66	0.88	0.113	0.004	0.07	0.28
SES group	0.042	0.004	0.47	0.72	0.128	0.006	0.02	0.08	0.222	0.006	<0.01	**0.01**	0.084	0.007	0.11	0.20
Sex	-0.031	0.004	0.59	0.89	-0.129	0.006	0.02	0.07	-0.218	0.006	<0.01	**0.01**	-0.089	0.006	0.09	0.38
NICU stay	-0.123	0.009	0.09	0.53	0.102	0.013	0.13	0.86	0.039	0.014	0.63	0.97	0.115	0.015	0.07	0.66
GA at delivery	0.018	0.001	0.82	0.82	-0.098	0.002	0.17	0.81	-0.091	0.002	0.30	0.60	-0.086	0.002	0.22	0.81
GA at T2	0.058	0.001	0.33	0.80	0.044	0.001	0.42	0.93	0.084	0.001	0.21	0.38	0.021	0.001	0.69	1.00
PMA at scan	0.567	0.001	<0.001	**<0.001**	-0.629	0.002	<0.001	**<0.001**	-0.365	0.002	<0.001	**<0.001**	-0.656	0.002	<0.001	**<0.001**
TNF-α T2:SES group	0.105	0.004	0.13	0.56	-0.055	0.006	0.38	0.74	-0.010	0.007	0.89	0.89	-0.072	0.007	0.24	0.90
**Uncinate Fasciculus**																
TNF-α T2	-0.170	0.002	0.02	0.07	0.088	0.003	0.24	0.71	-0.023	0.004	0.78	0.88	0.117	0.004	0.10	0.28
SES group	-0.045	0.003	0.44	0.72	0.107	0.005	0.09	0.17	0.087	0.006	0.23	0.34	0.097	0.006	0.10	0.20
Sex	-0.072	0.003	0.22	0.51	-0.006	0.005	0.93	0.95	-0.066	0.006	0.35	0.40	0.016	0.005	0.79	0.88
NICU stay	-0.022	0.007	0.76	0.82	0.015	0.012	0.85	0.86	-0.004	0.014	0.97	0.97	0.023	0.012	0.76	0.92
GA at delivery	0.116	0.001	0.14	0.42	0.044	0.002	0.60	0.81	0.147	0.002	0.12	0.54	-0.010	0.002	0.89	0.91
GA at T2	0.029	0.000	0.62	0.80	0.070	0.001	0.27	0.82	0.104	0.001	0.15	0.35	0.043	0.001	0.47	1.00
PMA at scan	0.546	0.001	<0.001	**<0.001**	-0.565	0.002	<0.001	**<0.001**	-0.243	0.002	<0.001	**<0.001**	-0.625	0.002	<0.001	**<0.001**
TNF-α T2:SES group	0.047	0.003	0.50	0.72	-0.131	0.006	0.08	0.71	-0.121	0.007	0.15	0.65	-0.115	0.006	0.10	0.88
**Inferior Fronto-Occipital Fasciculus**																
TNF-α T2	-0.056	0.002	0.37	0.56	0.010	0.004	0.88	0.94	-0.028	0.003	0.72	0.88	0.022	0.005	0.73	0.93
SES group	0.002	0.004	0.97	0.97	0.071	0.006	0.20	0.26	0.147	0.005	0.03	0.08	0.052	0.008	0.33	0.43
Sex	0.001	0.004	0.99	0.99	-0.098	0.006	0.08	0.23	-0.175	0.004	0.01	**0.03**	-0.066	0.007	0.22	0.66
NICU stay	-0.074	0.009	0.26	0.53	0.069	0.014	0.31	0.86	0.022	0.010	0.79	0.97	0.075	0.017	0.26	0.78

GA at delivery	0.078	0.001	0.27	0.61	-0.023	0.002	0.75	0.81	0.066	0.002	0.46	0.60	-0.043	0.003	0.54	0.82
GA at T2	0.039	0.001	0.47	0.80	0.027	0.001	0.63	0.95	0.106	0.001	0.12	0.35	0.005	0.001	0.93	1.00
PMA at scan	0.642	0.001	<0.001	**<0.001**	-0.658	0.002	<0.001	**<0.001**	-0.434	0.002	<0.001	**<0.001**	-0.667	0.003	<0.001	**<0.001**
TNF-α T2:SES group	0.019	0.004	0.76	0.76	0.002	0.007	0.97	0.97	0.032	0.005	0.68	0.88	0.000	0.008	1.00	1.00
**Anterior Limb of Internal Capsule**																
TNF-α T2	-0.025	0.002	0.69	0.78	-0.044	0.005	0.54	0.94	-0.076	0.004	0.35	0.72	-0.030	0.005	0.66	0.93
SES group	0.018	0.003	0.73	0.94	0.043	0.008	0.48	0.53	0.073	0.006	0.29	0.37	0.030	0.009	0.59	0.62
Sex	0.009	0.003	0.86	0.97	-0.050	0.007	0.40	0.89	-0.062	0.006	0.36	0.40	-0.043	0.008	0.45	0.83
NICU stay	-0.086	0.007	0.19	0.53	0.057	0.016	0.44	0.86	0.018	0.014	0.83	0.97	0.066	0.018	0.34	0.78
GA at delivery	0.134	0.001	0.06	0.42	-0.032	0.002	0.69	0.81	0.057	0.002	0.53	0.60	-0.058	0.003	0.44	0.82
GA at T2	-0.039	0.000	0.46	0.80	0.083	0.001	0.17	0.82	0.110	0.001	0.11	0.35	0.072	0.001	0.21	1.00
PMA at scan	0.627	0.001	<0.001	**<0.001**	-0.595	0.002	<0.001	**<0.001**	-0.435	0.002	<0.001	**<0.001**	-0.622	0.003	<0.001	**<0.001**
TNF-α T2:SES group	0.037	0.003	0.55	0.72	0.021	0.008	0.77	0.86	0.055	0.007	0.49	0.85	0.008	0.009	0.90	1.00
**Inferior Cingulum Bundle**																
TNF-α T2	-0.230	0.002	<0.01	**0.03**	0.021	0.002	0.76	0.94	-0.144	0.003	0.08	0.71	0.101	0.002	0.12	0.28
SES group	-0.113	0.003	0.09	0.26	0.218	0.003	<0.001	**<0.01**	0.145	0.005	0.04	0.08	0.194	0.004	<0.01	**0.01**
Sex	-0.070	0.003	0.28	0.51	-0.171	0.003	<0.01	**0.02**	-0.212	0.004	0.00	**0.01**	-0.128	0.003	0.02	0.18
NICU stay	-0.096	0.006	0.23	0.53	0.045	0.007	0.52	0.86	-0.041	0.010	0.62	0.97	0.089	0.008	0.19	0.78
GA at delivery	-0.129	0.001	0.14	0.42	0.042	0.001	0.57	0.81	-0.076	0.001	0.41	0.60	0.103	0.001	0.16	0.81
GA at T2	0.033	0.000	0.61	0.80	0.070	0.000	0.22	0.82	0.099	0.001	0.15	0.35	0.061	0.000	0.28	1.00
PMA at scan	0.486	0.001	<0.001	**<0.001**	-0.642	0.001	<0.001	**<0.001**	-0.311	0.001	<0.001	**<0.001**	-0.687	0.001	<0.001	**<0.001**
TNF-α T2:SES group	0.081	0.003	0.29	0.66	0.032	0.003	0.63	0.81	0.092	0.005	0.26	0.65	-0.013	0.004	0.84	1.00
**Fornix**																
TNF-α T2	-0.077	0.001	0.33	0.56	-0.053	0.002	0.44	0.94	-0.109	0.003	0.16	0.72	-0.014	0.002	0.84	0.94
SES group	-0.169	0.002	0.01	0.05	0.079	0.004	0.18	0.26	0.004	0.005	0.95	0.96	0.118	0.004	0.04	0.12
Sex	-0.126	0.002	0.06	0.51	-0.036	0.003	0.52	0.89	-0.100	0.005	0.12	0.28	0.009	0.004	0.88	0.88
NICU stay	0.030	0.005	0.72	0.82	-0.012	0.008	0.86	0.86	-0.004	0.011	0.96	0.97	-0.016	0.008	0.82	0.92
GA at delivery	0.075	0.001	0.40	0.64	0.018	0.001	0.81	0.81	0.063	0.002	0.47	0.60	-0.009	0.001	0.91	0.91
GA at T2	0.005	0.000	0.94	0.94	-0.002	0.000	0.98	0.99	-0.006	0.001	0.93	0.93	0.000	0.001	1.00	1.00
PMA at scan	0.410	0.001	<0.001	**<0.001**	-0.651	0.001	<0.001	**<0.001**	-0.478	0.002	<0.001	**<0.001**	-0.667	0.001	<0.001	**<0.001**
TNF-α T2:SES group	0.085	0.002	0.28	0.66	0.048	0.004	0.48	0.74	0.139	0.005	0.07	0.65	-0.001	0.004	0.99	1.00

TNF-α Trimester 3

	FA				MD				AD				RD			
	**β**	**SE**	***p***	***q***	**β**	**SE**	***p***	***q***	**β**	**SE**	***p***	***q***	**β**	**SE**	***p***	***q***
**Corpus Callosum**																
TNF-α T3	0.077	0.002	0.26	0.56	-0.137	0.002	0.04	0.11	-0.113	0.002	0.15	0.27	-0.117	0.003	0.07	0.19
SES group	0.116	0.004	0.05	0.14	-0.012	0.004	0.83	0.83	0.174	0.004	0.01	**0.01**	-0.058	0.005	0.29	0.29
Sex	-0.133	0.003	0.02	0.14	0.003	0.003	0.96	0.96	-0.093	0.004	0.14	0.25	0.068	0.005	0.19	0.56
NICU stay	-0.063	0.009	0.31	0.64	-0.024	0.009	0.70	0.90	-0.019	0.010	0.79	0.79	0.003	0.013	0.96	0.97
GA at delivery	-0.023	0.001	0.74	0.84	0.005	0.001	0.94	0.96	0.046	0.002	0.57	0.97	-0.008	0.002	0.91	0.98
GA at T3	0.021	0.001	0.72	0.93	-0.038	0.001	0.50	0.64	-0.043	0.001	0.52	0.60	-0.042	0.001	0.45	0.76
PMA at scan	0.467	0.001	<0.001	**<0.001**	-0.569	0.001	<0.001	**<0.001**	-0.182	0.002	0.01	**0.01**	-0.563	0.002	<0.001	**<0.001**
TNF-α T3:SES group	-0.044	0.003	0.52	0.59	0.049	0.004	0.46	0.46	0.034	0.004	0.66	0.66	0.044	0.005	0.49	0.56

**Superior Cingulum Bundle**																
TNF-α T3	-0.022	0.002	0.77	0.86	-0.086	0.003	0.21	0.31	-0.129	0.003	0.08	0.17	-0.053	0.003	0.45	0.67
SES group	-0.144	0.003	0.02	0.12	0.160	0.005	0.01	**0.01**	0.060	0.006	0.33	0.33	0.183	0.006	<0.01	**0.01**
Sex	-0.093	0.003	0.12	0.35	-0.014	0.005	0.79	0.96	-0.097	0.005	0.09	0.21	0.028	0.005	0.61	0.90
NICU stay	-0.050	0.008	0.48	0.67	-0.009	0.012	0.89	0.96	-0.051	0.014	0.45	0.58	0.012	0.014	0.85	0.97
GA at delivery	0.046	0.001	0.57	0.76	0.051	0.002	0.48	0.75	0.083	0.002	0.28	0.64	0.028	0.002	0.70	0.97
GA at T3	-0.034	0.001	0.60	0.90	-0.028	0.001	0.63	0.71	-0.055	0.001	0.38	0.60	-0.011	0.001	0.85	0.93
PMA at scan	0.310	0.001	<0.001	**<0.001**	-0.509	0.002	<0.001	**<0.001**	-0.398	0.002	<0.001	**<0.001**	-0.485	0.002	<0.001	**<0.001**
TNF-α T3:SES group	-0.002	0.003	0.98	0.98	0.157	0.005	0.02	0.10	0.207	0.005	<0.01	**0.02**	0.110	0.006	0.11	0.30
**Corticospinal Tract**																
TNF-α T3	0.030	0.002	0.64	0.86	-0.065	0.003	0.25	0.33	-0.061	0.002	0.33	0.41	-0.068	0.003	0.25	0.45
SES group	0.041	0.003	0.46	0.65	0.133	0.005	0.01	**0.01**	0.272	0.004	<0.001	**<0.001**	0.077	0.006	0.12	0.14
Sex	-0.013	0.003	0.80	0.90	-0.033	0.004	0.46	0.83	-0.061	0.004	0.22	0.29	-0.018	0.005	0.70	0.90
NICU stay	-0.071	0.008	0.24	0.64	0.003	0.011	0.96	0.96	-0.080	0.010	0.18	0.33	0.020	0.014	0.71	0.97
GA at delivery	-0.013	0.001	0.84	0.84	-0.041	0.002	0.50	0.75	-0.077	0.002	0.25	0.64	0.001	0.002	0.98	0.98
GA at T3	0.082	0.001	0.14	0.73	-0.086	0.001	0.08	0.64	-0.060	0.001	0.27	0.60	-0.088	0.001	0.08	0.74
PMA at scan	0.548	0.001	<0.001	**<0.001**	-0.667	0.002	<0.001	**<0.001**	-0.554	0.001	<0.001	**<0.001**	-0.661	0.002	<0.001	**<0.001**
TNF-α T3:SES group	-0.079	0.003	0.22	0.50	0.074	0.005	0.20	0.30	0.030	0.004	0.63	0.66	0.089	0.005	0.13	0.30
**Optic Radiation**																
TNF-α T3	0.026	0.002	0.69	0.86	-0.040	0.003	0.52	0.58	-0.016	0.003	0.82	0.82	-0.038	0.004	0.54	0.69
SES group	0.096	0.004	0.09	0.20	0.193	0.006	<0.001	**<0.01**	0.346	0.006	<0.001	**<0.001**	0.120	0.007	0.02	**0.04**
Sex	0.007	0.003	0.90	0.90	-0.107	0.005	0.03	0.19	-0.161	0.005	<0.01	**0.04**	-0.080	0.006	0.10	0.45
NICU stay	-0.103	0.009	0.10	0.45	0.036	0.013	0.54	0.90	-0.041	0.013	0.54	0.61	0.060	0.016	0.30	0.97
GA at delivery	-0.039	0.001	0.58	0.76	-0.100	0.002	0.13	0.75	-0.158	0.002	0.04	0.33	-0.063	0.003	0.33	0.74
GA at T3	0.013	0.001	0.82	0.93	-0.038	0.001	0.48	0.64	-0.032	0.001	0.60	0.60	-0.036	0.001	0.49	0.76
PMA at scan	0.542	0.001	<0.001	**<0.001**	-0.551	0.002	<0.001	**<0.001**	-0.256	0.002	<0.001	**<0.001**	-0.594	0.002	<0.001	**<0.001**
TNF-α T3:SES group	-0.046	0.003	0.48	0.59	0.064	0.005	0.30	0.39	0.045	0.005	0.53	0.66	0.061	0.006	0.32	0.41
**Uncinate Fasciculus**																
TNF-α T3	-0.067	0.002	0.31	0.56	-0.035	0.003	0.61	0.61	-0.096	0.003	0.19	0.29	-0.004	0.003	0.95	0.95
SES group	-0.041	0.003	0.47	0.65	0.176	0.005	<0.01	**0.01**	0.159	0.006	0.01	**0.01**	0.154	0.005	0.01	**0.01**
Sex	-0.075	0.003	0.16	0.35	-0.041	0.004	0.45	0.83	-0.100	0.005	0.09	0.21	-0.009	0.005	0.86	0.97
NICU stay	-0.027	0.007	0.67	0.67	-0.152	0.011	0.02	0.90	-0.187	0.014	0.01	0.07	-0.109	0.012	0.08	0.70
GA at delivery	0.140	0.001	0.05	0.43	0.004	0.002	0.96	0.96	0.099	0.002	0.20	0.64	-0.046	0.002	0.50	0.91
GA at T3	-0.003	0.001	0.95	0.95	-0.041	0.001	0.48	0.64	-0.039	0.001	0.54	0.60	-0.035	0.001	0.53	0.76
PMA at scan	0.458	0.001	<0.001	**<0.001**	-0.516	0.002	<0.001	**<0.001**	-0.253	0.002	<0.001	**<0.001**	-0.564	0.002	<0.001	**<0.001**
TNF-α T3:SES group	-0.046	0.003	0.49	0.59	-0.058	0.005	0.39	0.44	-0.095	0.005	0.20	0.40	-0.030	0.005	0.64	0.64
**Inferior Fronto-Occipital Fasciculus**																
TNF-α T3	0.078	0.002	0.20	0.56	-0.102	0.003	0.09	0.20	-0.064	0.002	0.37	0.41	-0.102	0.004	0.09	0.19
SES group	0.013	0.003	0.81	0.85	0.137	0.005	0.01	**0.01**	0.261	0.004	<0.001	**<0.001**	0.094	0.007	0.06	0.08
Sex	-0.024	0.003	0.61	0.90	-0.073	0.005	0.13	0.39	-0.138	0.004	0.01	0.06	-0.041	0.006	0.38	0.68
NICU stay	-0.053	0.008	0.36	0.64	-0.027	0.013	0.63	0.90	-0.089	0.010	0.18	0.33	-0.002	0.016	0.97	0.97
GA at delivery	0.074	0.001	0.25	0.75	-0.058	0.002	0.36	0.75	0.003	0.002	0.97	0.97	-0.066	0.003	0.30	0.74
GA at T3	0.037	0.001	0.48	0.87	-0.048	0.001	0.36	0.64	-0.040	0.001	0.51	0.60	-0.048	0.001	0.35	0.76

PMA at scan	0.594	0.001	<0.001	**<0.001**	-0.626	0.002	<0.001	**<0.001**	-0.400	0.002	<0.001	**<0.001**	-0.637	0.002	<0.001	**<0.001**
TNF-α T3:SES group	-0.081	0.003	0.18	0.50	0.094	0.005	0.12	0.26	0.077	0.004	0.28	0.41	0.095	0.006	0.11	0.30
**Anterior Limb of Internal Capsule**																
TNF-α T3	0.081	0.002	0.17	0.56	-0.135	0.004	0.03	0.11	-0.136	0.003	0.06	0.17	-0.129	0.004	0.03	0.19
SES group	0.010	0.003	0.85	0.85	0.127	0.007	0.02	**0.02**	0.185	0.006	<0.01	**<0.01**	0.099	0.007	0.06	0.08
Sex	-0.040	0.003	0.39	0.70	-0.012	0.006	0.81	0.96	-0.030	0.005	0.59	0.59	-0.001	0.007	0.98	0.98
NICU stay	-0.026	0.007	0.65	0.67	-0.053	0.015	0.37	0.90	-0.104	0.013	0.12	0.33	-0.032	0.018	0.57	0.97
GA at delivery	0.105	0.001	0.10	0.43	-0.049	0.002	0.46	0.75	0.023	0.002	0.75	0.97	-0.066	0.003	0.31	0.74
GA at T3	0.055	0.001	0.28	0.73	-0.070	0.001	0.20	0.64	-0.060	0.001	0.32	0.60	-0.068	0.001	0.19	0.76
PMA at scan	0.593	0.001	<0.001	**<0.001**	-0.590	0.002	<0.001	**<0.001**	-0.447	0.002	<0.001	**<0.001**	-0.611	0.003	<0.001	**<0.001**
TNF-α T3:SES group	-0.113	0.003	0.06	0.50	0.114	0.006	0.07	0.20	0.089	0.005	0.21	0.40	0.117	0.007	0.05	0.30
**Inferior Cingulum Bundle**																
TNF-α T3	-0.174	0.001	0.02	0.17	-0.101	0.002	0.11	0.20	-0.226	0.002	<0.01	**0.02**	-0.013	0.002	0.83	0.94
SES group	-0.041	0.003	0.51	0.65	0.219	0.003	<0.001	**<0.001**	0.193	0.004	<0.01	**<0.01**	0.170	0.003	<0.01	**0.01**
Sex	0.020	0.002	0.73	0.90	-0.102	0.003	0.04	0.19	-0.070	0.004	0.22	0.29	-0.099	0.003	0.05	0.45
NICU stay	-0.125	0.006	0.07	0.45	-0.032	0.007	0.59	0.90	-0.118	0.009	0.08	0.33	0.041	0.008	0.49	0.97
GA at delivery	-0.070	0.001	0.37	0.76	0.071	0.001	0.29	0.75	0.010	0.001	0.89	0.97	0.101	0.001	0.14	0.74
GA at T3	-0.062	0.000	0.33	0.73	-0.049	0.001	0.36	0.64	-0.097	0.001	0.12	0.60	-0.005	0.001	0.93	0.93
PMA at scan	0.387	0.001	<0.001	**<0.001**	-0.599	0.001	<0.001	**<0.001**	-0.322	0.001	<0.001	**<0.001**	-0.619	0.001	<0.001	**<0.001**
TNF-α T3:SES group	0.116	0.002	0.11	0.50	0.168	0.003	0.01	0.07	0.260	0.004	<0.001	**<0.01**	0.069	0.003	0.28	0.41
**Fornix**																
TNF-α T3	0.010	0.001	0.89	0.89	-0.143	0.002	0.02	0.11	-0.161	0.003	0.02	0.10	-0.115	0.002	0.06	0.19
SES group	-0.139	0.002	0.03	0.12	0.124	0.003	0.02	**0.02**	0.064	0.004	0.29	0.32	0.148	0.003	0.01	**0.01**
Sex	-0.118	0.002	0.04	0.19	0.009	0.003	0.86	0.96	-0.050	0.004	0.37	0.42	0.045	0.003	0.36	0.68
NICU stay	-0.034	0.005	0.63	0.67	-0.037	0.008	0.54	0.90	-0.063	0.011	0.34	0.51	-0.018	0.008	0.76	0.97
GA at delivery	0.042	0.001	0.59	0.76	-0.011	0.001	0.87	0.96	0.011	0.002	0.88	0.97	-0.021	0.001	0.75	0.97
GA at T3	-0.063	0.000	0.32	0.73	0.006	0.001	0.91	0.91	-0.043	0.001	0.48	0.60	0.029	0.001	0.59	0.76
PMA at scan	0.338	0.001	<0.001	**<0.001**	-0.610	0.001	<0.001	**<0.001**	-0.461	0.002	<0.001	**<0.001**	-0.621	0.001	<0.001	**<0.001**
TNF-α T3:SES group	-0.049	0.002	0.50	0.59	0.086	0.003	0.17	0.30	0.087	0.004	0.22	0.40	0.081	0.003	0.19	0.35

Bolded values represent statistical significance after FDR correction for multiple comparisons. β, standardized beta coefficient; SE, standard error; *q*, FDR-corrected p-value; FA, fractional anisotropy; MD, mean diffusivity; AD, axial diffusivity; RD, radial diffusivity; IL, interleukin; TNF-α, tumor necrosis factor alpha; GA, gestational age; PMA, infant postmenstrual age; Sex, child sex; T, trimester.

In addition to dichotomizing INR into SES groups, we also examined the association between continuous INR and neonatal dMRI parameters. As shown in Table S4, there was a positive relationship between INR and 1) CC AD; 2) CB MD and RD; 3) CST MD and AD; 4) OR MD, AD, and RD; 5) UF MD and AD; 6) IFOF MD and AD; 7) ALIC MD and AD; 8) CBIF MD, AD, and RD; and 9) FX RD. There was also a negative relationship between INR and FX FA.

Table S6. Multiple linear regression results of the association between continuous income-to-needs ratio and neonatal dMRI parameters.

	FA				MD				AD				RD			
	β	SE	*p*	*q*	β	SE	*p*	*q*	β	SE	*p*	*q*	β	SE	*p*	*q*
**Corpus Callosum**																
INR	0.064	0.001	0.21	0.60	0.026	0.001	0.59	0.59	0.132	0.001	0.02	**0.03**	-0.013	0.001	0.79	0.79
NICU stay	-0.102	0.007	0.09	0.32	-0.002	0.007	0.98	0.98	-0.003	0.007	0.97	0.97	0.029	0.010	0.62	0.78
GA at delivery	0.009	0.001	0.89	0.89	0.011	0.001	0.86	0.93	0.114	0.001	0.13	0.39	-0.017	0.002	0.79	0.89
Sex	-0.102	0.003	0.04	0.18	-0.005	0.003	0.92	0.92	-0.085	0.003	0.13	0.15	0.049	0.004	0.29	0.66
PMA at scan	0.460	0.001	<0.001	**<0.001**	-0.588	0.001	<0.001	**<0.001**	-0.237	0.001	<0.001	**<0.001**	-0.568	0.002	<0.001	**<0.001**
**Superior Cingulum Bundle**																
INR	-0.100	0.000	0.06	0.29	0.128	0.001	0.01	**0.02**	0.063	0.001	0.24	0.27	0.138	0.001	0.01	**0.02**
NICU stay	-0.063	0.006	0.35	0.44	0.079	0.009	0.20	0.76	0.037	0.011	0.58	0.94	0.084	0.011	0.17	0.51
GA at delivery	0.032	0.001	0.66	0.89	0.064	0.002	0.33	0.80	0.089	0.002	0.21	0.39	0.041	0.002	0.54	0.89
Sex	-0.105	0.003	0.05	0.18	-0.025	0.004	0.61	0.69	-0.118	0.005	0.03	0.06	0.020	0.005	0.69	0.88
PMA at scan	0.357	0.001	<0.001	**<0.001**	-0.548	0.002	<0.001	**<0.001**	-0.422	0.002	<0.001	**<0.001**	-0.527	0.002	<0.001	**<0.001**
**Corticospinal Tract**																
INR	0.054	0.000	0.26	0.60	0.107	0.001	0.01	**0.02**	0.228	0.001	<0.001	**<0.001**	0.060	0.001	0.16	0.18
NICU stay	-0.030	0.006	0.62	0.70	0.022	0.009	0.67	0.76	0.031	0.007	0.58	0.94	0.020	0.011	0.70	0.78
GA at delivery	0.023	0.001	0.72	0.89	-0.005	0.001	0.93	0.93	0.031	0.001	0.60	0.67	0.008	0.002	0.89	0.89
Sex	0.004	0.003	0.94	0.99	-0.045	0.004	0.27	0.56	-0.067	0.003	0.12	0.15	-0.031	0.005	0.46	0.82
PMA at scan	0.540	0.001	<0.001	**<0.001**	-0.718	0.001	<0.001	**<0.001**	-0.662	0.001	<0.001	**<0.001**	-0.692	0.002	<0.001	**<0.001**
**Optic Radiation**																
INR	0.042	0.001	0.38	0.68	0.173	0.001	<0.001	**<0.01**	0.269	0.001	<0.001	**<0.001**	0.121	0.001	0.01	**0.02**
NICU stay	-0.157	0.007	0.01	0.07	0.096	0.010	0.08	0.72	0.003	0.010	0.96	0.97	0.122	0.012	0.02	0.21
GA at delivery	-0.016	0.001	0.79	0.89	-0.060	0.002	0.31	0.80	-0.075	0.002	0.28	0.42	-0.044	0.002	0.44	0.89
Sex	-0.013	0.003	0.78	0.99	-0.122	0.005	0.01	0.05	-0.199	0.005	<0.001	**<0.01**	-0.085	0.005	0.04	0.28
PMA at scan	0.544	0.001	<0.001	**<0.001**	-0.600	0.002	<0.001	**<0.001**	-0.341	0.002	<0.001	**<0.001**	-0.628	0.002	<0.001	**<0.001**
**Uncinate Fasciculus**																
INR	0.009	0.000	0.86	0.96	0.118	0.001	0.02	**0.02**	0.135	0.001	0.02	**0.02**	0.088	0.001	0.06	0.07
NICU stay	-0.060	0.005	0.31	0.44	-0.031	0.009	0.61	0.76	-0.084	0.011	0.22	0.94	0.000	0.009	0.99	0.99
GA at delivery	0.088	0.001	0.17	0.38	0.060	0.001	0.36	0.80	0.133	0.002	0.07	0.39	0.015	0.002	0.81	0.89
Sex	-0.082	0.002	0.08	0.18	-0.049	0.004	0.31	0.56	-0.117	0.005	0.03	0.06	-0.012	0.004	0.78	0.88
PMA at scan	0.509	0.001	<0.001	**<0.001**	-0.589	0.001	<0.001	**<0.001**	-0.302	0.002	<0.001	**<0.001**	-0.636	0.002	<0.001	**<0.001**
**Inferior Fronto-Occipital Fasciculus**																
INR	0.001	0.000	0.99	0.99	0.118	0.001	0.01	**0.02**	0.221	0.001	<0.001	**<0.001**	0.087	0.001	0.04	0.07
NICU stay	-0.070	0.006	0.19	0.42	0.052	0.010	0.32	0.76	0.031	0.008	0.63	0.94	0.060	0.012	0.25	0.51
GA at delivery	0.088	0.001	0.12	0.37	-0.028	0.002	0.63	0.93	0.087	0.001	0.20	0.39	-0.052	0.002	0.35	0.89

Sex	0.001	0.003	0.99	0.99	-0.099	0.004	0.02	0.05	-0.164	0.003	<0.01	**0.01**	-0.068	0.005	0.10	0.29
PMA at scan	0.618	0.001	<0.001	**<0.001**	-0.673	0.002	<0.001	**<0.001**	-0.483	0.001	<0.001	**<0.001**	-0.675	0.002	<0.001	**<0.001**
**Anterior Limb of Internal Capsule**																
INR	-0.021	0.000	0.63	0.95	0.105	0.001	0.02	**0.03**	0.129	0.001	0.01	**0.02**	0.090	0.001	0.04	0.07
NICU stay	-0.052	0.005	0.33	0.44	0.054	0.012	0.34	0.76	0.037	0.010	0.57	0.94	0.059	0.014	0.28	0.51
GA at delivery	0.128	0.001	0.03	0.24	-0.013	0.002	0.83	0.93	0.093	0.002	0.18	0.39	-0.043	0.002	0.47	0.89
Sex	-0.032	0.002	0.46	0.82	-0.032	0.005	0.48	0.66	-0.053	0.005	0.31	0.31	-0.019	0.006	0.66	0.88
PMA at scan	0.602	0.001	<0.001	**<0.001**	-0.609	0.002	<0.001	**<0.001**	-0.467	0.002	<0.001	**<0.001**	-0.629	0.002	<0.001	**<0.001**
**Inferior Cingulum Bundle**																
INR	-0.013	0.000	0.81	0.96	0.193	0.000	<0.001	**<0.001**	0.193	0.001	<0.001	**<0.01**	0.154	0.000	<0.01	**<0.01**
NICU stay	-0.106	0.005	0.11	0.32	0.037	0.005	0.51	0.76	-0.033	0.007	0.63	0.94	0.081	0.006	0.15	0.51
GA at delivery	-0.119	0.001	0.09	0.37	0.081	0.001	0.18	0.80	-0.010	0.001	0.89	0.89	0.121	0.001	0.05	0.42
Sex	-0.025	0.002	0.63	0.94	-0.108	0.002	0.02	0.05	-0.118	0.003	0.03	0.06	-0.083	0.003	0.06	0.28
PMA at scan	0.440	0.001	<0.001	**<0.001**	-0.645	0.001	<0.001	**<0.001**	-0.345	0.001	<0.001	**<0.001**	-0.667	0.001	<0.001	**<0.001**
**Fornix**																
INR	-0.153	0.000	<0.01	**0.04**	0.074	0.000	0.11	0.12	0.015	0.001	0.77	0.77	0.107	0.000	0.02	**0.04**
NICU stay	-0.013	0.004	0.84	0.84	0.030	0.006	0.60	0.76	0.017	0.008	0.79	0.97	0.033	0.006	0.55	0.78
GA at delivery	0.034	0.001	0.63	0.89	0.031	0.001	0.61	0.93	0.053	0.001	0.44	0.57	0.016	0.001	0.79	0.89
Sex	-0.094	0.002	0.07	0.18	-0.029	0.003	0.51	0.66	-0.079	0.004	0.12	0.15	0.006	0.003	0.90	0.90
PMA at scan	0.378	0.001	<0.001	**<0.001**	-0.649	0.001	<0.001	**<0.001**	-0.499	0.001	<0.001	**<0.001**	-0.659	0.001	<0.001	**<0.001**

Bolded values represent statistical significance after FDR correction for multiple comparisons. β, standardized beta coefficient; SE, standard error; *q*, FDR-corrected p-value; FA, fractional anisotropy; MD, mean diffusivity; AD, axial diffusivity; RD, radial diffusivity; GA, gestational age; PMA, infant postmenstrual age; INR, income-to-needs ratio.

We also investigated the moderating role of continuous INR in the relationship between average maternal cytokine concentrations and neonatal dMRI parameters. As show in Table S5, continuous INR did not moderate this association.

Table S7. Multiple linear regression results of the moderating role of continuous income-to-needs ratio in the relationship between average maternal cytokine concentration and neonatal dMRI parameters.

**IL-6**																
	**FA**				**MD**				**AD**				**RD**			
	**β**	**SE**	***p***	***q***	**β**	**SE**	***p***	***q***	**β**	**SE**	***p***	***q***	**β**	**SE**	***p***	***q***
**Corpus Callosum**																
IL-6	-0.019	0.003	0.79	0.79	-0.064	0.002	0.33	0.42	-0.126	0.003	0.12	0.15	-0.027	0.003	0.68	0.75
INR	0.041	0.001	0.45	0.85	0.033	0.001	0.52	0.52	0.112	0.001	0.07	0.11	0.001	0.001	0.99	0.99
NICU stay	-0.097	0.007	0.11	0.34	-0.009	0.007	0.88	0.88	-0.004	0.007	0.95	0.98	0.023	0.010	0.70	0.78
GA at delivery	0.011	0.001	0.86	0.95	-0.006	0.001	0.93	0.93	0.092	0.001	0.23	0.55	-0.028	0.002	0.66	0.95
PMA at scan	0.456	0.001	<0.001	**<0.001**	-0.585	0.001	<0.001	**<0.001**	-0.235	0.001	<0.001	**<0.001**	-0.564	0.002	<0.001	**<0.001**
Sex	-0.101	0.003	0.04	0.21	0.001	0.003	0.98	0.98	-0.077	0.003	0.18	0.20	0.053	0.004	0.26	0.57
IL-6:INR	-0.066	0.001	0.37	0.75	0.098	0.001	0.16	0.72	0.057	0.001	0.49	0.74	0.086	0.001	0.22	0.81
**Superior Cingulum Bundle**																
IL-6	0.083	0.002	0.27	0.41	-0.121	0.003	0.08	0.30	-0.065	0.004	0.38	0.38	-0.129	0.004	0.06	0.28
INR	-0.090	0.001	0.12	0.56	0.135	0.001	0.01	**0.02**	0.078	0.001	0.18	0.20	0.139	0.001	0.01	**0.04**
NICU stay	-0.060	0.006	0.37	0.48	0.073	0.009	0.24	0.76	0.032	0.011	0.63	0.98	0.078	0.011	0.21	0.53
GA at delivery	0.043	0.001	0.56	0.92	0.043	0.002	0.52	0.93	0.073	0.002	0.31	0.55	0.020	0.002	0.76	0.95
PMA at scan	0.356	0.001	<0.001	**<0.001**	-0.543	0.002	<0.001	**<0.001**	-0.419	0.002	<0.001	**<0.001**	-0.522	0.002	<0.001	**<0.001**
Sex	-0.106	0.003	0.05	0.21	-0.020	0.004	0.68	0.77	-0.113	0.005	0.03	0.06	0.023	0.005	0.63	0.93
IL-6:INR	-0.048	0.001	0.54	0.75	0.116	0.001	0.11	0.72	0.088	0.001	0.26	0.65	0.111	0.001	0.12	0.81
**Corticospinal Tract**																
IL-6	-0.223	0.002	<0.01	**0.01**	0.071	0.003	0.21	0.31	-0.090	0.003	0.14	0.16	0.111	0.004	0.06	0.28
INR	0.020	0.001	0.69	0.85	0.111	0.001	0.01	**0.02**	0.200	0.001	<0.001	**<0.001**	0.075	0.001	0.10	0.14
NICU stay	-0.030	0.006	0.61	0.69	0.021	0.009	0.68	0.76	0.030	0.007	0.58	0.98	0.020	0.011	0.70	0.78
GA at delivery	0.004	0.001	0.95	0.95	-0.005	0.001	0.92	0.93	0.014	0.001	0.81	0.81	0.010	0.002	0.85	0.95
PMA at scan	0.541	0.001	<0.001	**<0.001**	-0.720	0.001	<0.001	**<0.001**	-0.668	0.001	<0.001	**<0.001**	-0.694	0.002	<0.001	**<0.001**
Sex	-0.001	0.003	0.98	0.98	-0.037	0.004	0.36	0.65	-0.058	0.003	0.18	0.20	-0.024	0.005	0.56	0.93
IL-6:INR	0.029	0.001	0.67	0.75	-0.002	0.001	0.98	0.98	0.013	0.001	0.84	0.84	0.000	0.001	1.00	1.00
**Optic Radiation**																
IL-6	-0.162	0.002	0.01	0.06	0.008	0.004	0.90	0.90	-0.122	0.004	0.09	0.14	0.056	0.004	0.35	0.60
INR	0.046	0.001	0.37	0.85	0.149	0.001	<0.01	**0.01**	0.241	0.001	0.00	0.00	0.102	0.001	0.03	0.09
NICU stay	-0.161	0.007	0.01	0.05	0.099	0.010	0.07	0.66	0.001	0.010	0.98	0.98	0.125	0.012	0.02	0.18
GA at delivery	-0.032	0.001	0.61	0.92	-0.064	0.002	0.28	0.93	-0.094	0.002	0.18	0.55	-0.042	0.002	0.46	0.95
PMA at scan	0.548	0.001	<0.001	**<0.001**	-0.603	0.002	<0.001	**<0.001**	-0.343	0.002	0.00	0.00	-0.631	0.002	0.00	0.00
Sex	-0.019	0.003	0.68	0.88	-0.114	0.005	0.01	0.07	-0.193	0.005	0.00	0.00	-0.077	0.005	0.07	0.35
IL-6:INR	0.089	0.001	0.19	0.75	-0.037	0.001	0.56	0.89	0.029	0.001	0.70	0.84	-0.058	0.001	0.36	0.81
**Uncinate Fasciculus**																
IL-6	-0.117	0.002	0.07	0.16	-0.135	0.003	0.04	0.30	-0.232	0.004	<0.01	**0.02**	-0.074	0.003	0.25	0.58

INR	-0.010	0.000	0.85	0.85	0.086	0.001	0.10	0.13	0.087	0.001	0.14	0.18	0.067	0.001	0.18	0.20
NICU stay	-0.057	0.005	0.33	0.48	-0.032	0.009	0.59	0.76	-0.084	0.011	0.21	0.98	-0.001	0.009	0.98	0.98
GA at delivery	0.079	0.001	0.22	0.48	0.043	0.001	0.51	0.93	0.108	0.002	0.14	0.55	0.004	0.002	0.95	0.95
PMA at scan	0.509	0.001	<0.001	**<0.001**	-0.589	0.001	<0.001	**<0.001**	-0.301	0.002	<0.001	**<0.001**	-0.636	0.002	<0.001	**<0.001**
Sex	-0.084	0.002	0.07	0.21	-0.045	0.004	0.34	0.65	-0.115	0.005	0.03	0.06	-0.009	0.004	0.84	0.94
IL-6:INR	-0.017	0.001	0.80	0.80	0.028	0.001	0.69	0.89	0.019	0.001	0.81	0.84	0.026	0.001	0.69	0.95
**Inferior Fronto-Occipital Fasciculus**																
IL-6	-0.032	0.002	0.59	0.76	-0.081	0.004	0.17	0.30	-0.180	0.003	0.01	0.05	-0.048	0.004	0.40	0.60
INR	-0.009	0.001	0.85	0.85	0.119	0.001	0.01	**0.02**	0.220	0.001	<0.001	**<0.001**	0.092	0.001	0.04	0.09
NICU stay	-0.068	0.006	0.20	0.46	0.048	0.010	0.36	0.76	0.025	0.008	0.69	0.98	0.056	0.012	0.28	0.53
GA at delivery	0.090	0.001	0.12	0.35	-0.044	0.002	0.44	0.93	0.061	0.001	0.36	0.55	-0.064	0.002	0.25	0.95
PMA at scan	0.616	0.001	<0.001	**<0.001**	-0.670	0.002	<0.001	**<0.001**	-0.478	0.001	<0.001	**<0.001**	-0.672	0.002	<0.001	**<0.001**
Sex	-0.004	0.003	0.92	0.98	-0.094	0.004	0.02	0.07	-0.161	0.003	<0.01	**<0.01**	-0.063	0.005	0.12	0.36
IL-6:INR	-0.031	0.001	0.62	0.75	0.071	0.001	0.25	0.75	0.106	0.001	0.15	0.65	0.062	0.001	0.31	0.81
**Anterior Limb of Internal Capsule**																
IL-6	-0.017	0.002	0.77	0.79	-0.093	0.004	0.14	0.30	-0.156	0.004	0.03	0.07	-0.070	0.005	0.26	0.58
INR	-0.009	0.000	0.85	0.85	0.093	0.001	0.06	0.09	0.122	0.001	0.03	0.06	0.078	0.001	0.11	0.14
NICU stay	-0.054	0.005	0.32	0.48	0.052	0.012	0.36	0.76	0.033	0.010	0.61	0.98	0.058	0.014	0.29	0.53
GA at delivery	0.125	0.001	0.03	0.26	-0.028	0.002	0.66	0.93	0.070	0.002	0.32	0.55	-0.053	0.002	0.37	0.95
PMA at scan	0.604	0.001	<0.001	**<0.001**	-0.607	0.002	<0.001	**<0.001**	-0.464	0.002	<0.001	**<0.001**	-0.627	0.002	<0.001	**<0.001**
Sex	-0.032	0.002	0.45	0.81	-0.028	0.005	0.54	0.70	-0.047	0.005	0.36	0.36	-0.016	0.006	0.72	0.93
IL-6:INR	0.030	0.001	0.64	0.75	0.037	0.001	0.58	0.89	0.081	0.001	0.29	0.65	0.021	0.001	0.75	0.95
**Inferior Cingulum Bundle**																
IL-6	-0.144	0.002	0.05	0.15	-0.038	0.002	0.55	0.61	-0.143	0.003	0.06	0.10	0.020	0.002	0.75	0.75
INR	-0.024	0.000	0.68	0.85	0.189	0.000	<0.001	**<0.01**	0.182	0.001	<0.01	**<0.01**	0.152	0.001	<0.01	**0.02**
NICU stay	-0.109	0.005	0.10	0.34	0.036	0.005	0.52	0.76	-0.036	0.007	0.59	0.98	0.082	0.006	0.15	0.53
GA at delivery	-0.136	0.001	0.06	0.26	0.073	0.001	0.24	0.93	-0.032	0.001	0.66	0.79	0.120	0.001	0.05	0.45
PMA at scan	0.443	0.001	<0.001	**<0.001**	-0.644	0.001	<0.001	**<0.001**	-0.342	0.001	<0.001	**<0.001**	-0.666	0.001	<0.001	**<0.001**
Sex	-0.026	0.002	0.62	0.88	-0.104	0.002	0.02	0.07	-0.113	0.003	0.03	0.06	-0.079	0.003	0.08	0.35
IL-6:INR	0.055	0.000	0.47	0.75	0.018	0.001	0.79	0.89	0.065	0.001	0.41	0.73	-0.013	0.001	0.85	0.95
**Fornix**																
IL-6	-0.116	0.001	0.12	0.21	-0.094	0.002	0.13	0.30	-0.170	0.003	0.02	0.05	-0.039	0.002	0.53	0.68
INR	-0.145	0.000	0.01	0.11	0.064	0.001	0.20	0.22	0.019	0.001	0.73	0.73	0.091	0.001	0.06	0.11
NICU stay	-0.021	0.004	0.75	0.75	0.029	0.006	0.61	0.76	0.011	0.008	0.86	0.98	0.036	0.006	0.52	0.78
GA at delivery	0.011	0.001	0.88	0.95	0.020	0.001	0.75	0.93	0.026	0.001	0.70	0.79	0.015	0.001	0.80	0.95
PMA at scan	0.391	0.001	<0.001	**<0.001**	-0.647	0.001	<0.001	**<0.001**	-0.495	0.001	<0.001	**<0.001**	-0.660	0.001	<0.001	**<0.001**
Sex	-0.088	0.002	0.10	0.21	-0.028	0.003	0.53	0.70	-0.074	0.004	0.14	0.20	0.003	0.003	0.94	0.94
IL-6:INR	0.123	0.000	0.12	0.75	0.019	0.001	0.77	0.89	0.115	0.001	0.12	0.65	-0.032	0.001	0.62	0.95
**IL-8**																
	**FA**				**MD**				**AD**				**RD**			
	**β**	**SE**	***p***	***q***	**β**	**SE**	***p***	***q***	**β**	**SE**	***p***	***q***	**β**	**SE**	***p***	***q***
**Corpus Callosum**																
IL-8	0.012	0.003	0.87	0.88	0.062	0.003	0.36	0.65	0.131	0.003	0.11	0.47	0.041	0.004	0.54	0.79

INR	0.061	0.001	0.23	0.68	0.018	0.001	0.70	0.70	0.116	0.001	0.04	0.06	-0.017	0.001	0.72	0.72
NICU stay	-0.102	0.007	0.09	0.35	-0.004	0.007	0.94	0.94	-0.008	0.007	0.91	1.00	0.028	0.010	0.64	0.80
GA at delivery	0.005	0.001	0.94	0.94	0.000	0.001	1.00	1.00	0.091	0.001	0.23	0.37	-0.022	0.002	0.73	0.94
PMA at scan	0.462	0.001	<0.001	**<0.001**	-0.581	0.001	<0.001	**<0.001**	-0.220	0.001	<0.001	**<0.001**	-0.564	0.002	<0.001	**<0.001**
Sex	-0.101	0.003	0.04	0.19	-0.001	0.003	0.98	0.98	-0.076	0.003	0.18	0.20	0.052	0.004	0.27	0.61
IL-8:INR	-0.030	0.001	0.67	0.85	-0.098	0.001	0.15	0.58	-0.203	0.001	0.01	0.12	-0.052	0.001	0.44	0.77
**Superior Cingulum Bundle**																
IL-8	-0.044	0.002	0.56	0.88	0.098	0.004	0.16	0.65	0.076	0.004	0.31	0.52	0.098	0.004	0.17	0.79
INR	-0.096	0.000	0.08	0.36	0.120	0.001	0.02	**0.03**	0.058	0.001	0.29	0.32	0.130	0.001	0.01	**0.03**
NICU stay	-0.062	0.006	0.36	0.46	0.076	0.009	0.21	0.78	0.035	0.011	0.60	0.98	0.081	0.011	0.19	0.54
GA at delivery	0.037	0.001	0.61	0.94	0.059	0.002	0.38	0.85	0.086	0.002	0.23	0.37	0.035	0.002	0.60	0.94
PMA at scan	0.353	0.001	<0.001	**<0.001**	-0.539	0.002	<0.001	**<0.001**	-0.416	0.002	<0.001	**<0.001**	-0.518	0.002	<0.001	**<0.001**
Sex	-0.108	0.003	0.04	0.19	-0.019	0.004	0.69	0.78	-0.114	0.005	0.03	0.07	0.025	0.005	0.61	0.89
IL-8:INR	0.059	0.001	0.44	0.85	-0.089	0.001	0.21	0.58	-0.058	0.001	0.45	0.50	-0.093	0.001	0.19	0.77
**Corticospinal Tract**																
IL-8	0.025	0.002	0.71	0.88	0.056	0.003	0.33	0.65	0.102	0.003	0.10	0.47	0.033	0.004	0.58	0.79
INR	0.049	0.000	0.32	0.72	0.103	0.001	0.01	**0.03**	0.217	0.001	<0.001	**<0.001**	0.060	0.001	0.17	0.19
NICU stay	-0.031	0.006	0.61	0.68	0.020	0.009	0.70	0.78	0.027	0.007	0.61	0.98	0.020	0.011	0.71	0.80
GA at delivery	0.011	0.001	0.86	0.94	-0.006	0.001	0.92	1.00	0.018	0.001	0.76	0.86	0.012	0.002	0.83	0.94
PMA at scan	0.545	0.001	<0.001	**<0.001**	-0.714	0.001	<0.001	**<0.001**	-0.652	0.001	<0.001	**<0.001**	-0.691	0.002	<0.001	**<0.001**
Sex	0.005	0.003	0.91	0.98	-0.042	0.004	0.30	0.61	-0.061	0.003	0.16	0.20	-0.030	0.005	0.48	0.87
IL-8:INR	-0.084	0.001	0.22	0.85	-0.035	0.001	0.55	0.70	-0.139	0.001	0.03	0.12	0.008	0.001	0.90	0.90
**Optic Radiation**																
IL-8	0.025	0.003	0.71	0.88	0.077	0.004	0.22	0.65	0.104	0.004	0.16	0.47	0.048	0.005	0.43	0.79
INR	0.039	0.001	0.42	0.76	0.167	0.001	<0.001	**<0.01**	0.259	0.001	<0.001	**<0.001**	0.118	0.001	0.01	**0.03**
NICU stay	-0.158	0.007	0.01	0.07	0.094	0.010	0.09	0.78	0.000	0.010	1.00	1.00	0.121	0.012	0.02	0.22
GA at delivery	-0.021	0.001	0.74	0.94	-0.063	0.002	0.29	0.85	-0.084	0.002	0.23	0.37	-0.045	0.002	0.44	0.94
PMA at scan	0.547	0.001	<0.001	**<0.001**	-0.593	0.002	<0.001	**<0.001**	-0.332	0.002	<0.001	**<0.001**	-0.624	0.002	<0.001	**<0.001**
Sex	-0.011	0.003	0.81	0.98	-0.118	0.005	0.01	0.06	-0.193	0.005	<0.001	**<0.01**	-0.083	0.005	0.05	0.31
IL-8:INR	-0.040	0.001	0.55	0.85	-0.062	0.001	0.32	0.58	-0.115	0.001	0.12	0.36	-0.032	0.001	0.60	0.77
**Uncinate Fasciculus**																
IL-8	0.027	0.002	0.69	0.88	0.041	0.003	0.55	0.77	0.061	0.004	0.44	0.56	0.024	0.004	0.72	0.79
INR	0.010	0.000	0.83	0.99	0.110	0.001	0.03	**0.04**	0.127	0.001	0.02	**0.04**	0.081	0.001	0.08	0.10
NICU stay	-0.060	0.005	0.31	0.46	-0.033	0.009	0.59	0.78	-0.086	0.011	0.21	0.98	-0.001	0.009	0.99	0.99
GA at delivery	0.097	0.001	0.13	0.31	0.043	0.001	0.51	0.85	0.121	0.002	0.10	0.37	-0.002	0.002	0.98	0.98
PMA at scan	0.508	0.001	<0.001	**<0.001**	-0.582	0.001	<0.001	**<0.001**	-0.295	0.002	<0.001	**<0.001**	-0.630	0.002	<0.001	**<0.001**
Sex	-0.081	0.002	0.08	0.19	-0.046	0.004	0.34	0.61	-0.114	0.005	0.04	0.07	-0.010	0.004	0.82	0.89
IL-8:INR	0.044	0.001	0.52	0.85	-0.125	0.001	0.07	0.58	-0.107	0.001	0.17	0.38	-0.114	0.001	0.08	0.72
**Inferior Fronto-Occipital Fasciculus**																
IL-8	0.011	0.002	0.86	0.88	0.032	0.004	0.60	0.77	0.080	0.003	0.26	0.52	0.016	0.005	0.79	0.79
INR	0.001	0.000	0.99	0.99	0.114	0.001	0.01	**0.03**	0.213	0.001	<0.001	**<0.001**	0.085	0.001	0.04	0.08
NICU stay	-0.071	0.006	0.19	0.42	0.051	0.010	0.33	0.78	0.028	0.008	0.66	0.98	0.059	0.012	0.25	0.54
GA at delivery	0.091	0.001	0.12	0.31	-0.033	0.002	0.56	0.85	0.079	0.001	0.24	0.37	-0.056	0.002	0.32	0.94

PMA at scan	0.619	0.001	<0.001	**<0.001**	-0.669	0.002	<0.001	**<0.001**	-0.475	0.001	<0.001	**<0.001**	-0.673	0.002	<0.001	**<0.001**
Sex	0.001	0.003	0.98	0.98	-0.097	0.004	0.02	0.06	-0.159	0.003	<0.01	**0.01**	-0.067	0.005	0.10	0.31
IL-8:INR	0.012	0.001	0.85	0.85	-0.051	0.001	0.40	0.60	-0.089	0.001	0.22	0.38	-0.037	0.001	0.53	0.77
**Anterior Limb of Internal Capsule**																
IL-8	-0.032	0.002	0.60	0.88	0.061	0.005	0.35	0.65	0.036	0.004	0.62	0.70	0.058	0.005	0.36	0.79
INR	-0.019	0.000	0.67	0.99	0.099	0.001	0.03	**0.04**	0.124	0.001	0.02	**0.04**	0.085	0.001	0.06	0.09
NICU stay	-0.052	0.005	0.34	0.46	0.052	0.012	0.36	0.78	0.036	0.010	0.59	0.98	0.057	0.014	0.30	0.54
GA at delivery	0.127	0.001	0.03	0.26	-0.019	0.002	0.75	0.97	0.082	0.002	0.25	0.37	-0.047	0.002	0.43	0.94
PMA at scan	0.600	0.001	<0.001	**<0.001**	-0.603	0.002	<0.001	**<0.001**	-0.462	0.002	<0.001	**<0.001**	-0.624	0.002	<0.001	**<0.001**
Sex	-0.033	0.002	0.44	0.79	-0.028	0.005	0.53	0.69	-0.051	0.005	0.33	0.33	-0.016	0.006	0.72	0.89
IL-8:INR	0.015	0.001	0.80	0.85	-0.071	0.001	0.28	0.58	-0.086	0.001	0.25	0.38	-0.058	0.001	0.35	0.77
**Inferior Cingulum Bundle**																
IL-8	-0.107	0.002	0.15	0.88	0.000	0.002	0.99	0.99	-0.072	0.003	0.35	0.52	0.036	0.002	0.58	0.79
INR	-0.001	0.000	0.98	0.99	0.192	0.000	<0.001	**<0.001**	0.199	0.001	<0.001	**<0.01**	0.150	0.000	<0.01	**0.01**
NICU stay	-0.103	0.005	0.12	0.35	0.037	0.005	0.51	0.78	-0.030	0.007	0.65	0.98	0.079	0.006	0.16	0.54
GA at delivery	-0.106	0.001	0.14	0.31	0.079	0.001	0.20	0.85	-0.004	0.001	0.95	0.95	0.114	0.001	0.06	0.55
PMA at scan	0.429	0.001	<0.001	**<0.001**	-0.644	0.001	<0.001	**<0.001**	-0.352	0.001	<0.001	**<0.001**	-0.663	0.001	<0.001	**<0.001**
Sex	-0.032	0.002	0.54	0.82	-0.108	0.002	0.02	0.06	-0.122	0.003	0.02	0.07	-0.081	0.003	0.07	0.31
IL-8:INR	0.142	0.000	0.06	0.53	-0.015	0.001	0.81	0.81	0.077	0.001	0.32	0.41	-0.059	0.001	0.36	0.77
**Fornix**																
IL-8	-0.011	0.001	0.88	0.88	0.016	0.002	0.81	0.91	0.001	0.003	0.98	0.98	0.021	0.002	0.74	0.79
INR	-0.153	0.000	<0.001	**0.04**	0.075	0.000	0.11	0.12	0.015	0.001	0.77	0.77	0.107	0.000	0.02	**0.04**
NICU stay	-0.013	0.004	0.85	0.85	0.029	0.006	0.60	0.78	0.017	0.008	0.79	1.00	0.033	0.006	0.56	0.80
GA at delivery	0.030	0.001	0.67	0.94	0.036	0.001	0.56	0.85	0.055	0.001	0.42	0.55	0.022	0.001	0.72	0.94
PMA at scan	0.378	0.001	<0.001	**<0.001**	-0.649	0.001	<0.001	**<0.001**	-0.500	0.001	<0.001	**<0.001**	-0.659	0.001	<0.001	**<0.001**
Sex	-0.094	0.002	0.07	0.19	-0.029	0.003	0.52	0.69	-0.079	0.004	0.12	0.18	0.006	0.003	0.89	0.89
IL-8:INR	-0.016	0.000	0.83	0.85	0.021	0.001	0.74	0.81	0.014	0.001	0.85	0.85	0.023	0.001	0.71	0.80
**IL-10**																
	**FA**				**MD**				**AD**				**RD**			
	**β**	**SE**	***p***	***q***	**β**	**SE**	***p***	***q***	**β**	**SE**	***p***	***q***	**β**	**SE**	***p***	***q***
**Corpus Callosum**																
IL-10	-0.057	-0.833	0.41	0.46	0.094	1.428	0.15	0.28	-0.030	0.003	0.71	0.71	0.095	0.004	0.15	0.26
INR	0.065	1.298	0.20	0.59	0.026	0.532	0.59	0.59	0.135	0.001	0.02	**0.03**	-0.014	0.001	0.77	0.77
NICU stay	-0.088	-1.447	0.15	0.45	-0.014	-0.239	0.81	0.83	0.003	0.007	0.97	0.97	0.015	0.010	0.80	0.90
GA at delivery	0.015	0.234	0.82	0.83	0.009	0.145	0.88	1.00	0.119	0.001	0.12	0.36	-0.021	0.002	0.74	0.83
PMA at scan	0.467	8.729	<0.001	**<0.001**	-0.590	-11.412	<0.001	**<0.001**	-0.233	0.001	<0.001	**<0.001**	-0.572	0.002	<0.001	**<0.001**
Sex	-0.106	-2.159	0.03	0.22	0.004	0.081	0.94	0.94	-0.080	0.003	0.16	0.18	0.057	0.004	0.22	0.50
IL-10:INR	-0.021	-0.305	0.76	0.95	-0.002	-0.028	0.98	0.98	0.036	0.001	0.66	0.97	-0.004	0.001	0.95	0.95
**Superior Cingulum Bundle**																
IL-10	-0.100	-1.340	0.18	0.23	0.106	1.545	0.12	0.28	0.066	0.004	0.38	0.56	0.117	0.004	0.09	0.21
INR	-0.097	-1.789	0.07	0.34	0.129	2.586	0.01	**0.02**	0.066	0.001	0.22	0.25	0.138	0.001	0.01	**0.02**
NICU stay	-0.061	-0.900	0.37	0.50	0.077	1.245	0.21	0.80	0.034	0.011	0.61	0.92	0.082	0.011	0.19	0.54
GA at delivery	0.033	0.453	0.65	0.83	0.067	1.016	0.31	0.83	0.092	0.002	0.20	0.36	0.043	0.002	0.52	0.83

PMA at scan	0.356	6.071	<0.001	**<0.001**	-0.543	-10.068	<0.001	**<0.001**	-0.417	0.002	<0.001	**<0.001**	-0.523	0.002	<0.001	**<0.001**
Sex	-0.105	-1.973	0.05	0.22	-0.027	-0.555	0.58	0.65	-0.120	0.005	0.02	0.05	0.017	0.005	0.72	0.81
IL-10:INR	0.100	1.340	0.18	0.95	-0.058	-0.839	0.40	0.88	0.005	0.001	0.95	0.97	-0.083	0.001	0.23	0.95
**Corticospinal Tract**																
IL-10	-0.221	-3.410	<0.01	**0.01**	0.176	3.122	0.00	**0.02**	0.058	0.003	0.35	0.56	0.190	0.004	<0.01	**0.01**
INR	0.047	0.990	0.32	0.68	0.111	2.725	0.01	**0.02**	0.226	0.001	<0.001	**<0.001**	0.066	0.001	0.12	0.13
NICU stay	-0.018	-0.314	0.75	0.83	0.011	0.217	0.83	0.83	0.029	0.007	0.60	0.92	0.009	0.011	0.86	0.90
GA at delivery	0.013	0.210	0.83	0.83	0.000	-0.002	1.00	1.00	0.031	0.001	0.60	0.67	0.012	0.002	0.83	0.83
PMA at scan	0.532	10.470	<0.001	**<0.001**	-0.711	-16.184	<0.001	**<0.001**	-0.660	0.001	<0.001	**<0.001**	-0.684	0.002	<0.001	**<0.001**
Sex	0.001	0.016	0.99	0.99	-0.039	-0.981	0.33	0.59	-0.061	0.003	0.16	0.18	-0.027	0.005	0.52	0.81
IL-10:INR	0.008	0.128	0.90	0.95	-0.036	-0.631	0.53	0.88	-0.046	0.001	0.46	0.97	-0.020	0.001	0.74	0.95
**Optic Radiation**																
IL-10	-0.113	-1.738	0.08	0.15	0.169	2.772	0.01	**0.03**	0.135	0.004	0.06	0.38	0.165	0.005	0.01	**0.03**
INR	0.042	0.882	0.38	0.68	0.170	3.844	<0.001	**<0.01**	0.266	0.001	**0.00**	**<0.001**	0.119	0.001	0.01	**0.02**
NICU stay	-0.147	-2.511	0.01	0.11	0.089	1.628	0.10	0.80	0.002	0.010	0.97	0.97	0.113	0.012	0.03	0.31
GA at delivery	-0.016	-0.258	0.80	0.83	-0.059	-1.009	0.31	0.83	-0.074	0.002	0.29	0.43	-0.044	0.002	0.44	0.83
PMA at scan	0.543	10.674	<0.001	**<0.001**	-0.598	-12.537	<0.001	**<0.001**	-0.339	0.002	<0.001	**<0.001**	-0.625	0.002	<0.001	**<0.001**
Sex	-0.019	-0.404	0.69	0.88	-0.115	-2.669	0.01	0.06	-0.196	0.005	<0.001	**<0.01**	-0.079	0.005	0.06	0.28
IL-10:INR	0.026	0.396	0.69	0.95	-0.116	-1.909	0.06	0.52	-0.139	0.001	0.05	0.49	-0.096	0.001	0.11	0.95
**Uncinate Fasciculus**																
IL-10	-0.037	-0.557	0.58	0.58	-0.067	-0.994	0.32	0.48	-0.107	0.004	0.16	0.38	-0.038	0.004	0.55	0.71
INR	0.004	0.081	0.94	0.97	0.117	2.378	0.02	**0.03**	0.129	0.001	0.02	**0.03**	0.088	0.001	0.06	0.08
NICU stay	-0.060	-0.999	0.32	0.50	-0.022	-0.364	0.72	0.83	-0.073	0.011	0.28	0.92	0.007	0.009	0.90	0.90
GA at delivery	0.083	1.295	0.20	0.44	0.059	0.904	0.37	0.83	0.128	0.002	0.08	0.36	0.015	0.002	0.81	0.83
PMA at scan	0.504	9.669	<0.001	**<0.001**	-0.589	-11.141	<0.001	**<0.001**	-0.302	0.002	<0.001	**<0.001**	-0.635	0.002	<0.001	**<0.001**
Sex	-0.076	-1.600	0.11	0.25	-0.058	-1.217	0.22	0.51	-0.123	0.005	0.02	0.05	-0.022	0.004	0.63	0.81
IL-10:INR	-0.030	-0.452	0.65	0.95	-0.037	-0.548	0.58	0.88	-0.057	0.001	0.46	0.97	-0.028	0.001	0.67	0.95
**Inferior Fronto-Occipital Fasciculus**																
IL-10	-0.104	-1.770	0.08	0.15	0.048	0.815	0.42	0.53	-0.033	0.003	0.64	0.71	0.063	0.005	0.27	0.41
INR	-0.002	-0.044	0.97	0.97	0.120	2.792	0.01	**0.02**	0.224	0.001	<0.001	**<0.001**	0.090	0.001	0.03	0.06
NICU stay	-0.059	-1.126	0.26	0.50	0.049	0.926	0.36	0.80	0.038	0.008	0.55	0.92	0.054	0.012	0.30	0.54
GA at delivery	0.086	1.522	0.13	0.39	-0.025	-0.446	0.66	0.98	0.090	0.001	0.19	0.36	-0.049	0.002	0.38	0.83
PMA at scan	0.619	13.427	<0.001	**<0.001**	-0.670	-14.503	<0.001	**<0.001**	-0.478	0.001	<0.001	**<0.001**	-0.673	0.002	<0.001	**<0.001**
Sex	-0.004	-0.096	0.92	0.99	-0.099	-2.367	0.02	0.06	-0.170	0.003	<0.01	**<0.01**	-0.067	0.005	0.10	0.30
IL-10:INR	-0.008	-0.133	0.89	0.95	0.002	0.037	0.97	0.98	0.004	0.001	0.95	0.97	0.012	0.001	0.83	0.95
**Anterior Limb of Internal Capsule**																
IL-10	-0.085	-1.426	0.16	0.23	-0.016	-0.254	0.80	0.80	-0.101	0.004	0.17	0.38	0.009	0.005	0.89	0.92
INR	-0.023	-0.528	0.60	0.90	0.108	2.313	0.02	**0.03**	0.132	0.001	0.01	**0.03**	0.093	0.001	0.04	0.06
NICU stay	-0.046	-0.865	0.39	0.50	0.054	0.943	0.35	0.80	0.043	0.010	0.51	0.92	0.058	0.014	0.30	0.54
GA at delivery	0.125	2.178	0.03	0.27	-0.011	-0.182	0.86	1.00	0.095	0.002	0.18	0.36	-0.040	0.002	0.50	0.83
PMA at scan	0.600	12.865	<0.001	**<0.001**	-0.607	-12.069	<0.001	**<0.001**	-0.465	0.002	<0.001	**<0.001**	-0.627	0.002	<0.001	**<0.001**
Sex	-0.034	-0.813	0.42	0.75	-0.034	-0.733	0.46	0.65	-0.057	0.005	0.27	0.27	-0.020	0.006	0.65	0.81
IL-10:INR	0.004	0.062	0.95	0.95	0.035	0.544	0.59	0.88	0.063	0.001	0.39	0.97	0.026	0.001	0.67	0.95
**Inferior Cingulum Bundle**																

IL-10	-0.145	-1.974	0.05	0.15	0.146	2.319	0.02	0.06	0.055	0.003	0.46	0.60	0.163	0.002	0.01	**0.03**
INR	-0.019	-0.352	0.73	0.93	0.194	4.230	<0.001	**<0.001**	0.189	0.001	<0.01	**<0.01**	0.157	0.000	<0.01	**0.01**
NICU stay	-0.103	-1.570	0.12	0.45	0.030	0.524	0.60	0.83	-0.039	0.007	0.57	0.92	0.074	0.006	0.18	0.54
GA at delivery	-0.129	-1.820	0.07	0.31	0.084	1.380	0.17	0.83	-0.014	0.001	0.84	0.84	0.126	0.001	0.04	0.33
PMA at scan	0.432	7.534	<0.001	**<0.001**	-0.639	-12.926	<0.001	**<0.001**	-0.346	0.001	<0.001	**<0.001**	-0.660	0.001	<0.001	**<0.001**
Sex	-0.023	-0.438	0.66	0.88	-0.108	-2.412	0.02	0.06	-0.116	0.003	0.03	0.06	-0.085	0.003	0.06	0.28
IL-10:INR	0.016	0.214	0.83	0.95	-0.051	-0.809	0.42	0.88	-0.046	0.001	0.54	0.97	-0.044	0.001	0.48	0.95
**Fornix**																
IL-10	0.134	1.812	0.07	0.15	0.040	0.626	0.53	0.60	0.099	0.003	0.16	0.38	-0.006	0.002	0.92	0.92
INR	-0.153	-2.845	<0.01	**0.04**	0.075	1.625	0.11	0.12	0.018	0.001	0.73	0.73	0.106	0.000	0.02	**0.04**
NICU stay	-0.015	-0.220	0.83	0.83	0.025	0.447	0.66	0.83	0.012	0.008	0.85	0.97	0.030	0.006	0.59	0.89
GA at delivery	0.039	0.548	0.58	0.83	0.031	0.511	0.61	0.98	0.057	0.001	0.40	0.52	0.014	0.001	0.81	0.83
PMA at scan	0.381	6.576	<0.001	**<0.001**	-0.648	-13.085	<0.001	**<0.001**	-0.498	0.001	<0.001	**<0.001**	-0.659	0.001	<0.001	**<0.001**
Sex	-0.084	-1.597	0.11	0.25	-0.027	-0.594	0.55	0.65	-0.071	0.004	0.16	0.18	0.004	0.003	0.92	0.92
IL-10:INR	-0.078	-1.052	0.29	0.95	-0.003	-0.041	0.97	0.98	-0.003	0.001	0.97	0.97	0.011	0.001	0.86	0.95
**TNF-α**																
	**FA**				**MD**				**AD**				**RD**			
	**β**	**SE**	***p***	***q***	**β**	**SE**	***p***	***q***	**β**	**SE**	***p***	***q***	**β**	**SE**	***p***	***q***
**Corpus Callosum**																
TNF-α	0.073	0.003	0.29	0.65	-0.091	0.003	0.17	0.43	-0.064	0.003	0.41	0.62	-0.083	0.004	0.20	0.50
INR	0.056	0.001	0.27	0.69	0.030	0.001	0.54	0.54	0.132	0.001	0.02	**0.03**	-0.008	0.001	0.87	0.87
NICU stay	-0.105	0.007	0.09	0.31	-0.001	0.007	0.99	0.99	-0.004	0.007	0.95	0.97	0.031	0.010	0.60	0.75
GA at delivery	0.014	0.001	0.83	0.83	0.001	0.001	0.99	0.99	0.107	0.001	0.16	0.40	-0.026	0.002	0.69	0.87
PMA at scan	0.457	0.001	<0.001	**<0.001**	-0.586	0.001	<0.001	**<0.001**	-0.240	0.001	<0.001	**<0.001**	-0.566	0.002	<0.001	**<0.001**
Sex	-0.105	0.003	0.03	0.15	-0.008	0.003	0.87	0.87	-0.094	0.003	0.10	0.13	0.049	0.004	0.30	0.67
TNF-α:INR	-0.075	0.001	0.27	0.76	0.047	0.001	0.47	0.71	-0.003	0.001	0.97	0.97	0.055	0.001	0.40	0.72
**Superior Cingulum Bundle**																
TNF-α	0.034	0.002	0.64	0.84	-0.081	0.004	0.23	0.43	-0.060	0.004	0.41	0.62	-0.073	0.004	0.29	0.52
INR	-0.104	0.000	0.06	0.27	0.141	0.001	0.01	**0.01**	0.076	0.001	0.16	0.18	0.149	0.001	<0.01	**0.02**
NICU stay	-0.066	0.006	0.33	0.46	0.079	0.009	0.20	0.74	0.033	0.011	0.62	0.97	0.085	0.011	0.17	0.49
GA at delivery	0.033	0.001	0.64	0.83	0.058	0.002	0.39	0.87	0.082	0.002	0.25	0.40	0.035	0.002	0.60	0.87
PMA at scan	0.355	0.001	<0.001	**<0.001**	-0.541	0.002	<0.001	**<0.001**	-0.416	0.002	<0.001	**<0.001**	-0.522	0.002	<0.001	**<0.001**
Sex	-0.109	0.003	0.04	0.15	-0.026	0.004	0.59	0.66	-0.122	0.005	0.02	0.05	0.020	0.005	0.69	0.82
TNF-α:INR	-0.029	0.001	0.70	0.90	0.122	0.001	0.07	0.40	0.123	0.001	0.09	0.28	0.100	0.001	0.15	0.49
**Corticospinal Tract**																
TNF-α	-0.016	0.002	0.81	0.84	0.007	0.003	0.90	0.90	-0.021	0.003	0.73	0.81	0.002	0.004	0.97	0.97
INR	0.050	0.000	0.30	0.69	0.108	0.001	0.01	**0.02**	0.225	0.001	<0.001	**<0.001**	0.063	0.001	0.15	0.17
NICU stay	-0.033	0.006	0.58	0.66	0.023	0.009	0.66	0.74	0.030	0.007	0.58	0.97	0.023	0.011	0.67	0.75
GA at delivery	0.018	0.001	0.78	0.83	-0.004	0.001	0.94	0.99	0.027	0.001	0.65	0.69	0.010	0.002	0.86	0.87
PMA at scan	0.538	0.001	<0.001	**<0.001**	-0.717	0.001	<0.001	**<0.001**	-0.663	0.001	<0.001	**<0.001**	-0.691	0.002	<0.001	**<0.001**
Sex	-0.002	0.003	0.96	0.99	-0.041	0.004	0.31	0.58	-0.067	0.003	0.13	0.15	-0.027	0.005	0.53	0.82
TNF-α:INR	-0.031	0.001	0.64	0.90	0.004	0.001	0.95	0.95	-0.016	0.001	0.79	0.91	0.020	0.001	0.73	0.83
**Optic Radiation**																
TNF-α	-0.017	0.003	0.79	0.84	0.022	0.004	0.71	0.80	0.017	0.004	0.81	0.81	0.025	0.005	0.67	0.95

INR	0.041	0.001	0.40	0.71	0.170	0.001	<0.001	**<0.01**	0.265	0.001	<0.001	**<0.001**	0.119	0.001	0.01	**0.02**
NICU stay	-0.158	0.007	0.01	0.07	0.093	0.010	0.09	0.74	-0.002	0.010	0.97	0.97	0.120	0.012	0.03	0.24
GA at delivery	-0.018	0.001	0.77	0.83	-0.061	0.002	0.31	0.87	-0.078	0.002	0.27	0.40	-0.044	0.002	0.44	0.87
PMA at scan	0.543	0.001	<0.001	**<0.001**	-0.601	0.002	<0.001	**<0.001**	-0.344	0.002	<0.001	**<0.001**	-0.628	0.002	<0.001	**<0.001**
Sex	-0.017	0.003	0.72	0.93	-0.122	0.005	0.01	0.05	-0.202	0.005	<0.001	**<0.01**	-0.084	0.005	0.05	0.27
TNF-α:INR	-0.010	0.001	0.88	0.97	-0.014	0.001	0.82	0.92	-0.017	0.001	0.81	0.91	-0.012	0.001	0.84	0.84
**Uncinate Fasciculus**																
TNF-α	-0.115	0.002	0.08	0.34	-0.036	0.003	0.59	0.76	-0.124	0.004	0.10	0.22	0.009	0.004	0.89	0.97
INR	0.010	0.000	0.83	0.96	0.116	0.001	0.02	**0.03**	0.133	0.001	0.02	**0.03**	0.086	0.001	0.07	0.09
NICU stay	-0.055	0.005	0.36	0.46	-0.027	0.009	0.66	0.74	-0.075	0.011	0.27	0.97	0.002	0.009	0.97	0.97
GA at delivery	0.078	0.001	0.22	0.49	0.058	0.001	0.37	0.87	0.124	0.002	0.09	0.40	0.016	0.002	0.79	0.87
PMA at scan	0.508	0.001	<0.001	**<0.001**	-0.588	0.001	<0.001	**<0.001**	-0.301	0.002	<0.001	**<0.001**	-0.635	0.002	<0.001	**<0.001**
Sex	-0.085	0.002	0.07	0.16	-0.048	0.004	0.32	0.58	-0.118	0.005	0.03	0.05	-0.011	0.004	0.81	0.82
TNF-α:INR	0.002	0.000	0.97	0.97	-0.028	0.001	0.68	0.87	-0.032	0.001	0.67	0.91	-0.025	0.001	0.70	0.83
**Inferior Fronto-Occipital Fasciculus**																
TNF-α	0.050	0.002	0.39	0.70	-0.069	0.004	0.24	0.43	-0.047	0.003	0.50	0.65	-0.070	0.005	0.22	0.50
INR	-0.006	0.000	0.89	0.96	0.124	0.001	<0.01	**0.01**	0.225	0.001	<0.001	**<0.001**	0.095	0.001	0.03	**0.04**
NICU stay	-0.073	0.006	0.17	0.39	0.053	0.010	0.32	0.74	0.027	0.008	0.67	0.97	0.061	0.012	0.24	0.49
GA at delivery	0.090	0.001	0.12	0.35	-0.034	0.002	0.55	0.94	0.080	0.001	0.24	0.40	-0.057	0.002	0.31	0.87
PMA at scan	0.614	0.001	<0.001	**<0.001**	-0.668	0.002	<0.001	**<0.001**	-0.481	0.001	<0.001	**<0.001**	-0.670	0.002	<0.001	**<0.001**
Sex	0.000	0.003	0.99	0.99	-0.101	0.004	0.02	0.05	-0.169	0.003	<0.01	**<0.01**	-0.070	0.005	0.09	0.27
TNF-α:INR	-0.057	0.001	0.34	0.76	0.058	0.001	0.32	0.58	0.049	0.001	0.48	0.87	0.063	0.001	0.27	0.60
**Anterior Limb of Internal Capsule**																
TNF-α	0.075	0.002	0.20	0.61	-0.133	0.005	0.03	0.15	-0.131	0.004	0.07	0.21	-0.126	0.005	0.04	0.21
INR	-0.028	0.000	0.52	0.78	0.115	0.001	0.01	**0.02**	0.138	0.001	0.01	**0.02**	0.100	0.001	0.03	**0.04**
NICU stay	-0.052	0.005	0.33	0.46	0.056	0.012	0.33	0.74	0.039	0.010	0.55	0.97	0.061	0.014	0.27	0.49
GA at delivery	0.134	0.001	0.02	0.18	-0.025	0.002	0.68	0.94	0.081	0.002	0.25	0.40	-0.054	0.002	0.36	0.87
PMA at scan	0.597	0.001	<0.001	**<0.001**	-0.603	0.002	<0.001	**<0.001**	-0.462	0.002	<0.001	**<0.001**	-0.623	0.002	<0.001	**<0.001**
Sex	-0.028	0.002	0.51	0.76	-0.036	0.005	0.43	0.64	-0.057	0.005	0.27	0.27	-0.023	0.006	0.59	0.82
TNF-α:INR	-0.071	0.000	0.23	0.76	0.094	0.001	0.13	0.40	0.082	0.001	0.26	0.57	0.093	0.001	0.13	0.49
**Inferior Cingulum Bundle**																
TNF-α	-0.180	0.002	0.01	0.11	-0.048	0.002	0.44	0.66	-0.177	0.003	0.02	0.10	0.021	0.002	0.74	0.95
INR	-0.003	0.000	0.96	0.96	0.201	0.000	<0.001	**<0.001**	0.208	0.001	<0.001	**<0.001**	0.157	0.000	<0.01	**0.01**
NICU stay	-0.107	0.005	0.10	0.31	0.035	0.005	0.54	0.74	-0.035	0.007	0.60	0.97	0.079	0.006	0.16	0.49
GA at delivery	-0.138	0.001	0.05	0.23	0.076	0.001	0.22	0.87	-0.029	0.001	0.69	0.69	0.123	0.001	0.04	0.40
PMA at scan	0.444	0.001	<0.001	**<0.001**	-0.639	0.001	<0.001	**<0.001**	-0.340	0.001	<0.001	**<0.001**	-0.664	0.001	<0.001	**<0.001**
Sex	-0.038	0.002	0.47	0.76	-0.111	0.002	0.01	0.05	-0.130	0.003	0.01	**0.04**	-0.081	0.003	0.07	0.27
TNF-α:INR	0.085	0.000	0.24	0.76	0.082	0.000	0.19	0.43	0.145	0.001	0.05	0.28	0.035	0.001	0.57	0.83
**Fornix**																
TNF-α	0.015	0.001	0.84	0.84	-0.144	0.002	0.02	0.15	-0.159	0.003	0.02	0.10	-0.122	0.002	0.05	0.21
INR	-0.159	0.000	<0.01	**0.03**	0.087	0.000	0.06	0.07	0.028	0.001	0.59	0.59	0.118	0.000	0.01	**0.02**
NICU stay	-0.022	0.004	0.74	0.74	0.036	0.006	0.53	0.74	0.018	0.008	0.78	0.97	0.041	0.006	0.46	0.68
GA at delivery	0.028	0.001	0.70	0.83	0.021	0.001	0.73	0.94	0.037	0.001	0.59	0.69	0.010	0.001	0.87	0.87
PMA at scan	0.374	0.001	<0.001	**<0.001**	-0.644	0.001	<0.001	**<0.001**	-0.493	0.001	<0.001	**<0.001**	-0.655	0.001	<0.001	**<0.001**

Sex	-0.105	0.002	0.05	0.15	-0.028	0.003	0.53	0.66	-0.084	0.004	0.09	0.13	0.010	0.003	0.82	0.82
TNF-α:INR	-0.029	0.000	0.70	0.90	0.103	0.001	0.10	0.40	0.130	0.001	0.06	0.28	0.085	0.001	0.16	0.49

β, standardized beta coefficient; SE, standard error; *q*, FDR-corrected p-value; IL, interleukin; TNF-α, tumor necrosis factor alpha; FA, fractional anisotropy; MD, mean diffusivity; AD, axial diffusivity; RD, radial diffusivity; GA, gestational age; PMA, infant postmenstrual age; INR, income-to-needs ratio.

We performed a series of specificity tests excluding neonates born <34 weeks' gestation and/or weighing <2,000g at birth to examine if results held. As seen in Table S6, there was a positive relationship between family SES group and 1) CC AD; 2) CB MD and RD; 3) CST MD and RD; 4) OR MD, AD, and RD; 5) IFOF MD, AD, and RD; 6) ALIC MD and AD; 7) CBIF MD, AD, and RD; and 8) FX MD and RD. These results similarly reflect those in the main analysis where all neonates were included.

Table S8. Specificity test excluding neonates born <34 weeks' gestation and/or weighing <2,000g at birth. Multiple linear regression results of the association between family socioeconomic status group and neonatal dMRI parameters.

	FA				MD				AD				RD			
	β	SE	*p*	*q*	β	SE	*p*	*q*	β	SE	*p*	*q*	β	SE	*p*	*q*
**Corpus Callosum**																
SES group	0.095	0.003	0.06	0.19	-0.010	0.003	0.83	0.83	0.153	0.004	0.01	**0.01**	-0.055	0.004	0.27	0.27
GA at delivery	0.038	0.001	0.49	0.63	0.050	0.001	0.35	0.46	0.095	0.001	0.13	0.40	0.023	0.002	0.67	0.87
PMA at scan	0.469	0.001	<0.001	**<0.001**	-0.575	0.001	<0.001	**<0.001**	-0.222	0.001	<0.001	**<0.001**	-0.567	0.002	<0.001	**<0.001**
Sex	-0.084	0.003	0.09	0.23	-0.014	0.003	0.77	0.77	-0.095	0.003	0.10	0.13	0.040	0.004	0.41	0.74
**Superior Cingulum Bundle**																
SES group	-0.155	0.003	0.01	0.05	0.159	0.004	<0.01	**0.01**	0.057	0.005	0.30	0.30	0.182	0.005	<0.001	**<0.01**
GA at delivery	0.132	0.001	0.03	0.06	-0.051	0.002	0.35	0.46	0.029	0.002	0.63	0.83	-0.082	0.002	0.14	0.25
PMA at scan	0.313	0.001	<0.001	**<0.001**	-0.505	0.002	<0.001	**<0.001**	-0.403	0.002	<0.001	**<0.001**	-0.480	0.002	<0.001	**<0.001**
Sex	-0.097	0.003	0.07	0.23	-0.044	0.004	0.37	0.54	-0.135	0.005	0.01	**0.03**	0.002	0.005	0.97	0.97
**Corticospinal Tract**																
SES group	0.039	0.003	0.43	0.70	0.112	0.004	0.01	**0.01**	0.224	0.004	<0.001	**<0.001**	0.066	0.005	0.13	0.15
GA at delivery	0.040	0.001	0.45	0.63	0.019	0.002	0.68	0.76	0.056	0.001	0.26	0.58	0.002	0.002	0.97	0.97
PMA at scan	0.540	0.001	<0.001	**<0.001**	-0.711	0.002	<0.001	**<0.001**	-0.641	0.001	<0.001	**<0.001**	-0.685	0.002	<0.001	**<0.001**
Sex	-0.009	0.003	0.86	0.93	-0.050	0.004	0.24	0.43	-0.085	0.003	0.06	0.09	-0.035	0.005	0.41	0.74
**Optic Radiation**																
SES group	0.075	0.003	0.13	0.30	0.178	0.005	<0.001	**<0.01**	0.304	0.005	<0.001	**<0.001**	0.116	0.006	0.01	**0.02**
GA at delivery	0.012	0.001	0.83	0.83	-0.110	0.002	0.03	0.26	-0.127	0.002	0.02	0.11	-0.091	0.002	0.07	0.15
PMA at scan	0.531	0.001	<0.001	**<0.001**	-0.570	0.002	<0.001	**<0.001**	-0.313	0.002	<0.001	**<0.001**	-0.603	0.002	<0.001	**<0.001**
Sex	0.004	0.003	0.93	0.93	-0.128	0.005	<0.01	**0.04**	-0.191	0.005	<0.001	**<0.01**	-0.097	0.006	0.03	0.14
**Uncinate Fasciculus**																
SES group	-0.036	0.003	0.47	0.70	0.141	0.004	0.01	**0.01**	0.129	0.005	0.02	**0.03**	0.123	0.004	0.01	**0.02**
GA at delivery	0.129	0.001	0.02	0.05	0.065	0.002	0.23	0.46	0.170	0.002	0.01	0.05	0.003	0.002	0.95	0.97
PMA at scan	0.489	0.001	<0.001	**<0.001**	-0.565	0.001	<0.001	**<0.001**	-0.288	0.002	<0.001	**<0.001**	-0.612	0.002	<0.001	**<0.001**
Sex	-0.081	0.002	0.10	0.23	-0.067	0.004	0.17	0.39	-0.135	0.005	0.02	**0.03**	-0.029	0.004	0.54	0.75
**Inferior Fronto-Occipital Fasciculus**																
SES group	-0.001	0.003	0.98	0.98	0.133	0.005	<0.01	**0.01**	0.243	0.004	<0.001	**<0.001**	0.096	0.006	0.03	**0.04**
GA at delivery	0.139	0.001	0.00	**0.04**	-0.083	0.002	0.08	0.27	0.019	0.001	0.74	0.83	-0.105	0.002	0.03	0.15
PMA at scan	0.594	0.001	<0.001	**<0.001**	-0.643	0.002	<0.001	**<0.001**	-0.461	0.001	<0.001	**<0.001**	-0.647	0.002	<0.001	**<0.001**
Sex	0.005	0.003	0.92	0.93	-0.109	0.005	0.01	**0.04**	-0.175	0.004	<0.01	**<0.01**	-0.077	0.006	0.07	0.21
**Anterior Limb of Internal Capsule**																
SES group	0.011	0.003	0.80	0.90	0.121	0.006	0.01	**0.01**	0.176	0.005	<0.01	**<0.01**	0.096	0.007	0.04	0.05
GA at delivery	0.117	0.001	0.02	0.05	-0.087	0.002	0.09	0.27	-0.019	0.002	0.74	0.83	-0.101	0.002	0.04	0.15

PMA at scan	0.595	0.001	<0.001	**<0.001**	-0.579	0.002	<0.001	**<0.001**	-0.440	0.002	<0.001	**<0.001**	-0.601	0.002	<0.001	**<0.001**
Sex	-0.028	0.002	0.53	0.93	-0.037	0.006	0.42	0.54	-0.059	0.005	0.25	0.25	-0.025	0.006	0.58	0.75
**Inferior Cingulum Bundle**																
SES group	-0.032	0.002	0.56	0.72	0.200	0.003	<0.001	**<0.001**	0.182	0.004	<0.01	**<0.01**	0.160	0.003	<0.01	**<0.01**
GA at delivery	-0.101	0.001	0.09	0.16	0.057	0.001	0.26	0.46	-0.036	0.001	0.55	0.83	0.096	0.001	0.06	0.15
PMA at scan	0.421	0.001	<0.001	**<0.001**	-0.618	0.001	<0.001	**<0.001**	-0.330	0.001	<0.001	**<0.001**	-0.643	0.001	<0.001	**<0.001**
Sex	-0.006	0.002	0.91	0.93	-0.120	0.002	0.01	**0.04**	-0.112	0.003	0.04	0.07	-0.103	0.003	0.03	0.14
**Fornix**																
SES group	-0.109	0.002	0.05	0.19	0.114	0.003	0.01	**0.02**	0.071	0.004	0.17	0.20	0.129	0.003	0.01	**0.02**
GA at delivery	0.025	0.001	0.68	0.76	-0.013	0.001	0.79	0.79	0.005	0.001	0.92	0.92	-0.021	0.001	0.68	0.87
PMA at scan	0.355	0.001	<0.001	**<0.001**	-0.634	0.001	<0.001	**<0.001**	-0.492	0.001	<0.001	**<0.001**	-0.640	0.001	<0.001	**<0.001**
Sex	-0.091	0.002	0.09	0.23	-0.031	0.003	0.50	0.56	-0.079	0.004	0.12	0.14	0.003	0.003	0.94	0.97

Bolded values represent statistical significance after FDR correction for multiple comparisons. β, standardized beta coefficient; SE, standard error; *q*, FDR-corrected p-value; FA, fractional anisotropy; MD, mean diffusivity; AD, axial diffusivity; RD, radial diffusivity; GA, gestational age; PMA, infant postmenstrual age; Sex, child sex.

Excluding neonates born <34 weeks' gestation and/or weighing <2,000g at birth, there was a negative relationship between average maternal IL-6 and 1) CST FA; 2) OR AD; 3) UF MD and AD; 4) IFOF AD; and 5) ALIC AD. There was a negative relationship between average maternal IL-10 and CST, IFOF, and CBIF AD but a positive relationship with CST MD and RD and CBIF RD.

Table S9. Specificity test excluding neonates born <34 weeks' gestation and/or weighing <2,000g at birth. Multiple linear regression results of the association between maternal cytokine concentrations and neonatal dMRI parameters.

	IL-6															
	FA				MD				AD				RD			
	β	SE	*p*	*q*	β	SE	*p*	*q*	β	SE	*p*	*q*	β	SE	*p*	*q*
**Corpus Callosum**																
IL-6	-0.093	0.002	0.07	0.12	-0.006	0.002	0.91	0.91	-0.106	0.002	0.06	0.08	0.036	0.002	0.46	0.59
GA at delivery	0.035	0.001	0.53	0.79	0.023	0.001	0.66	0.74	0.066	0.001	0.29	0.62	0.006	0.002	0.91	1.00
PMA at scan	0.468	0.001	<0.001	**<0.001**	-0.568	0.001	<0.001	**<0.001**	-0.203	0.001	<0.01	**<0.01**	-0.561	0.002	<0.001	**<0.001**
Sex	-0.074	0.003	0.14	0.42	-0.010	0.003	0.84	0.84	-0.080	0.003	0.16	0.18	0.038	0.004	0.43	0.65
**Superior Cingulum Bundle**																
IL-6	0.080	0.002	0.14	0.21	-0.081	0.002	0.11	0.24	-0.027	0.003	0.62	0.62	-0.093	0.003	0.07	0.22
GA at delivery	0.125	0.001	0.04	0.08	-0.060	0.002	0.27	0.41	0.014	0.002	0.81	0.91	-0.087	0.002	0.11	0.22
PMA at scan	0.307	0.001	<0.001	**<0.001**	-0.491	0.002	<0.001	**<0.001**	-0.393	0.002	**<0.001**	**<0.001**	-0.466	0.002	<0.001	**<0.001**
Sex	-0.093	0.003	0.08	0.42	-0.041	0.004	0.41	0.55	-0.131	0.005	0.01	**0.04**	0.004	0.005	0.93	0.98
**Corticospinal Tract**																
IL-6	-0.197	0.002	<0.001	**<0.001**	0.056	0.002	0.19	0.28	-0.103	0.002	0.03	0.05	0.103	0.003	0.02	0.15
GA at delivery	-0.023	0.001	0.65	0.84	0.054	0.002	0.23	0.41	0.055	0.001	0.27	0.62	0.047	0.002	0.31	0.47
PMA at scan	0.549	0.001	<0.001	**<0.001**	-0.708	0.001	<0.001	**<0.001**	-0.628	0.001	<0.001	**<0.001**	-0.685	0.002	<0.001	**<0.001**
Sex	-0.004	0.003	0.93	0.98	-0.047	0.004	0.26	0.48	-0.078	0.003	0.09	0.13	-0.034	0.005	0.43	0.65
**Optic Radiation**																
IL-6	-0.102	0.002	0.04	0.11	-0.048	0.003	0.30	0.39	-0.138	0.003	0.01	**0.03**	-0.006	0.003	0.89	0.98
GA at delivery	0.001	0.001	0.98	0.98	-0.095	0.002	0.06	0.20	-0.115	0.002	0.05	0.22	-0.076	0.002	0.12	0.22
PMA at scan	0.542	0.001	<0.001	**<0.001**	-0.562	0.002	<0.001	**<0.001**	-0.292	0.002	<0.001	**<0.001**	-0.601	0.002	<0.001	**<0.001**
Sex	-0.001	0.003	0.98	0.98	-0.114	0.005	0.01	0.05	-0.173	0.005	<0.01	**0.01**	-0.086	0.005	0.06	0.25

**Uncinate Fasciculus**																
IL-6	-0.122	0.001	0.01	0.06	-0.140	0.002	<0.01	**0.04**	-0.234	0.003	<0.001	**<0.001**	-0.078	0.002	0.10	0.22
GA at delivery	0.088	0.001	0.10	0.18	0.052	0.002	0.33	0.42	0.129	0.002	0.03	0.22	0.005	0.002	0.92	1.00
PMA at scan	0.494	0.001	<0.001	**<0.001**	-0.548	0.001	<0.001	**<0.001**	-0.269	0.002	<0.001	**<0.001**	-0.600	0.002	<0.001	**<0.001**
Sex	-0.063	0.002	0.19	0.43	-0.074	0.004	0.13	0.29	-0.129	0.005	0.02	**0.04**	-0.040	0.004	0.39	0.65
**Inferior Fronto-Occipital Fasciculus**																
IL-6	-0.033	0.002	0.45	0.58	-0.059	0.003	0.18	0.28	-0.130	0.002	0.01	**0.03**	-0.032	0.003	0.46	0.59
GA at delivery	0.126	0.001	0.01	0.06	-0.086	0.002	0.07	0.20	0.000	0.001	1.00	1.00	-0.103	0.002	0.03	0.08
PMA at scan	0.601	0.001	<0.001	**<0.001**	-0.633	0.002	<0.001	**<0.001**	-0.429	0.001	<0.001	**<0.001**	-0.642	0.002	<0.001	**<0.001**
Sex	-0.005	0.003	0.90	0.98	-0.103	0.004	0.02	0.05	-0.173	0.004	<0.01	**0.01**	-0.070	0.005	0.09	0.28
**Anterior Limb of Internal Capsule**																
IL-6	0.001	0.001	0.97	0.97	-0.096	0.003	0.04	0.18	-0.137	0.003	0.01	**0.03**	-0.078	0.004	0.08	0.22
GA at delivery	0.110	0.001	0.02	0.07	-0.105	0.002	0.04	0.20	-0.054	0.002	0.34	0.62	-0.114	0.002	0.02	0.08
PMA at scan	0.598	0.001	<0.001	**<0.001**	-0.564	0.002	<0.001	**<0.001**	-0.416	0.002	<0.001	**<0.001**	-0.590	0.002	<0.001	**<0.001**
Sex	-0.030	0.002	0.49	0.89	-0.031	0.005	0.49	0.55	-0.051	0.005	0.33	0.33	-0.020	0.006	0.66	0.85
**Inferior Cingulum Bundle**																
IL-6	-0.105	0.001	0.05	0.12	-0.042	0.001	0.37	0.42	-0.113	0.002	0.04	0.06	-0.001	0.002	0.98	0.98
GA at delivery	-0.146	0.001	0.01	0.06	0.079	0.001	0.13	0.28	-0.044	0.001	0.46	0.69	0.125	0.001	0.01	0.08
PMA at scan	0.422	0.001	<0.001	**<0.001**	-0.600	0.001	<0.001	**<0.001**	-0.309	0.001	<0.001	**<0.001**	-0.629	0.001	<0.001	**<0.001**
Sex	0.012	0.002	0.82	0.98	-0.123	0.003	0.01	0.05	-0.104	0.003	0.05	0.10	-0.111	0.003	0.02	0.15
**Fornix**																
IL-6	0.004	0.001	0.94	0.97	-0.079	0.002	0.08	0.24	-0.074	0.002	0.15	0.16	-0.070	0.002	0.13	0.23
GA at delivery	0.001	0.001	0.98	0.98	0.004	0.001	0.93	0.93	0.016	0.001	0.77	0.91	0.000	0.001	1.00	1.00
PMA at scan	0.354	0.001	<0.001	**<0.001**	-0.629	0.001	<0.001	**<0.001**	-0.498	0.001	<0.001	**<0.001**	-0.634	0.001	<0.001	**<0.001**
Sex	-0.083	0.002	0.12	0.42	-0.032	0.003	0.47	0.55	-0.076	0.004	0.13	0.17	-0.001	0.003	0.98	0.98

**IL-8**																
	**FA**				**MD**				**AD**				**RD**			

	**β**	**SE**	***p***	***q***	**β**	**SE**	***p***	***q***	**β**	**SE**	***p***	***q***	**β**	**SE**	***p***	***q***
**Corpus Callosum**																
IL-8	-0.045	0.002	0.37	0.83	-0.003	0.002	0.95	0.95	-0.052	0.002	0.36	0.99	0.025	0.002	0.61	0.89
GA at delivery	0.048	0.001	0.38	0.56	0.034	0.001	0.51	0.62	0.095	0.001	0.13	0.38	0.008	0.002	0.88	0.88
PMA at scan	0.463	0.001	<0.001	**<0.001**	-0.563	0.001	<0.001	**<0.001**	-0.204	0.001	<0.01	**<0.01**	-0.556	0.002	<0.001	**<0.001**
Sex	-0.075	0.003	0.13	0.40	-0.020	0.003	0.67	0.67	-0.094	0.003	0.10	0.11	0.032	0.004	0.51	0.71
**Superior Cingulum Bundle**																
IL-8	-0.015	0.002	0.79	0.86	0.024	0.003	0.63	0.95	0.015	0.003	0.77	0.99	0.027	0.003	0.59	0.89
GA at delivery	0.103	0.001	0.08	0.14	-0.031	0.002	0.57	0.62	0.030	0.002	0.60	0.77	-0.057	0.002	0.30	0.53
PMA at scan	0.305	0.001	<0.001	**<0.001**	-0.488	0.002	<0.001	**<0.001**	-0.392	0.002	<0.001	**<0.001**	-0.464	0.002	<0.001	**<0.001**
Sex	-0.089	0.003	0.10	0.40	-0.053	0.004	0.29	0.44	-0.140	0.005	0.01	**0.02**	-0.006	0.005	0.90	1.00
**Corticospinal Tract**																
IL-8	-0.059	0.002	0.22	0.83	0.044	0.002	0.30	0.95	-0.001	0.002	0.98	0.99	0.054	0.003	0.21	0.89
GA at delivery	0.010	0.001	0.85	0.88	0.056	0.002	0.21	0.32	0.088	0.001	0.08	0.36	0.038	0.002	0.41	0.61
PMA at scan	0.541	0.001	<0.001	**<0.001**	-0.699	0.002	<0.001	**<0.001**	-0.621	0.001	<0.001	**<0.001**	-0.677	0.002	<0.001	**<0.001**
Sex	-0.007	0.003	0.88	0.99	-0.054	0.004	0.20	0.36	-0.093	0.003	0.04	0.07	-0.038	0.005	0.37	0.67
**Optic Radiation**																
IL-8	-0.010	0.002	0.84	0.86	0.038	0.003	0.40	0.95	0.024	0.003	0.65	0.99	0.032	0.003	0.47	0.89
GA at delivery	0.018	0.001	0.73	0.88	-0.073	0.002	0.14	0.29	-0.073	0.002	0.21	0.47	-0.064	0.002	0.19	0.42
PMA at scan	0.539	0.001	<0.001	**<0.001**	-0.557	0.002	<0.001	**<0.001**	-0.289	0.002	<0.001	**<0.001**	-0.597	0.002	<0.001	**<0.001**
Sex	0.000	0.003	0.99	0.99	-0.124	0.005	0.01	**0.03**	-0.185	0.005	<0.01	**<0.01**	-0.093	0.005	0.04	0.17
**Uncinate Fasciculus**																
IL-8	0.063	0.001	0.20	0.83	-0.055	0.002	0.26	0.95	-0.020	0.003	0.72	0.99	-0.064	0.002	0.17	0.89
GA at delivery	0.114	0.001	0.03	0.07	0.083	0.002	0.12	0.29	0.180	0.002	<0.01	**0.03**	0.022	0.002	0.66	0.84
PMA at scan	0.490	0.001	<0.001	**<0.001**	-0.551	0.001	<0.001	**<0.001**	-0.275	0.002	<0.001	**<0.001**	-0.602	0.002	<0.001	**<0.001**
Sex	-0.061	0.002	0.21	0.46	-0.085	0.004	0.08	0.19	-0.140	0.005	0.01	**0.03**	-0.049	0.004	0.29	0.65
**Inferior Fronto-Occipital Fasciculus**																
IL-8	0.008	0.002	0.86	0.86	0.003	0.003	0.94	0.95	0.015	0.002	0.76	0.99	-0.001	0.003	0.98	1.00
GA at delivery	0.127	0.001	0.01	0.07	-0.065	0.002	0.16	0.29	0.036	0.001	0.52	0.77	-0.088	0.002	0.05	0.18
PMA at scan	0.599	0.001	<0.001	**<0.001**	-0.632	0.002	<0.001	**<0.001**	-0.430	0.001	<0.001	**<0.001**	-0.641	0.002	<0.001	**<0.001**
Sex	0.001	0.003	0.99	0.99	-0.112	0.004	0.01	**0.03**	-0.184	0.004	<0.001	**<0.01**	-0.079	0.005	0.06	0.18
**Anterior Limb of Internal Capsule**																
IL-8	-0.045	0.001	0.30	0.83	0.004	0.003	0.92	0.95	-0.052	0.003	0.32	0.99	0.018	0.004	0.69	0.89
GA at delivery	0.106	0.001	0.03	0.07	-0.078	0.002	0.12	0.29	-0.019	0.002	0.73	0.79	-0.091	0.002	0.06	0.18
PMA at scan	0.595	0.001	<0.001	**<0.001**	-0.566	0.002	<0.001	**<0.001**	-0.420	0.002	<0.001	**<0.001**	-0.591	0.002	<0.001	**<0.001**
Sex	-0.031	0.002	0.48	0.86	-0.040	0.005	0.38	0.48	-0.065	0.005	0.21	0.21	-0.027	0.006	0.55	0.71
**Inferior Cingulum Bundle**																
IL-8	-0.030	0.001	0.58	0.86	-0.015	0.001	0.74	0.95	-0.042	0.002	0.44	0.99	0.000	0.002	1.00	1.00
GA at delivery	-0.125	0.001	0.03	0.07	0.093	0.001	0.07	0.29	-0.015	0.001	0.79	0.79	0.130	0.001	0.01	0.09
PMA at scan	0.418	0.001	<0.001	**<0.001**	-0.601	0.001	<0.001	**<0.001**	-0.313	0.001	<0.001	**<0.001**	-0.630	0.001	<0.001	**<0.001**
Sex	0.007	0.002	0.90	0.99	-0.131	0.003	0.01	**0.03**	-0.117	0.003	0.03	0.06	-0.115	0.003	0.01	0.12
**Fornix**																
IL-8	-0.030	0.001	0.57	0.86	0.023	0.002	0.61	0.95	0.000	0.002	0.99	0.99	0.033	0.002	0.47	0.89

	β	SE	*p*	*q*	β	SE	*p*	*q*	β	SE	*p*	*q*	β	SE	*p*	*q*
GA at delivery	0.009	0.001	0.88	0.88	0.024	0.001	0.62	0.62	0.040	0.001	0.46	0.77	0.015	0.001	0.76	0.86
PMA at scan	0.347	0.001	<0.001	**<0.001**	-0.631	0.001	<0.001	**<0.001**	-0.497	0.001	<0.001	**<0.001**	-0.635	0.001	<0.001	**<0.001**
Sex	-0.094	0.002	0.08	0.40	-0.036	0.003	0.43	0.48	-0.087	0.004	0.09	0.11	0.000	0.003	1.00	1.00

IL-10

	**FA**				**MD**				**AD**				**RD**			
	**β**	**SE**	***p***	***q***	**β**	**SE**	***p***	***q***	**β**	**SE**	***p***	***q***	**β**	**SE**	***p***	***q***
**Corpus Callosum**																
IL-10	-0.081	0.002	0.11	0.16	0.075	0.002	0.12	0.22	-0.024	0.002	0.68	0.73	0.082	0.002	0.08	0.18
GA at delivery	0.054	0.001	0.32	0.48	0.035	0.001	0.50	0.63	0.099	0.001	0.11	0.34	0.010	0.002	0.84	0.84
PMA at scan	0.471	0.001	<0.001	**<0.001**	-0.565	0.001	<0.001	**<0.001**	-0.197	0.001	<0.01	**<0.01**	-0.568	0.002	<0.001	**<0.001**
Sex	-0.079	0.003	0.11	0.35	-0.013	0.003	0.79	0.79	-0.087	0.003	0.13	0.14	0.034	0.004	0.48	0.68
**Superior Cingulum Bundle**																
IL-10	-0.035	0.002	0.51	0.51	0.055	0.003	0.27	0.36	0.054	0.003	0.31	0.70	0.052	0.003	0.31	0.40
GA at delivery	0.102	0.001	0.08	0.15	-0.028	0.002	0.60	0.63	0.033	0.002	0.56	0.73	-0.052	0.002	0.35	0.52
PMA at scan	0.305	0.001	<0.001	**<0.001**	-0.485	0.002	<0.001	**<0.001**	-0.387	0.002	<0.001	**<0.001**	-0.463	0.002	<0.001	**<0.001**
Sex	-0.085	0.003	0.12	0.35	-0.059	0.004	0.24	0.36	-0.143	0.005	0.01	**0.02**	-0.015	0.005	0.77	0.87
**Corticospinal Tract**																
IL-10	-0.219	0.002	<0.001	**<0.001**	0.153	0.002	<0.001	**<0.01**	0.022	0.002	0.64	0.73	0.181	0.003	<0.001	**<0.001**
GA at delivery	-0.001	0.001	0.99	0.99	0.064	0.002	0.15	0.27	0.089	0.001	0.08	0.34	0.049	0.002	0.28	0.51
PMA at scan	0.533	0.001	<0.001	**<0.001**	-0.693	0.001	<0.001	**<0.001**	-0.619	0.001	<0.001	**<0.001**	-0.670	0.002	<0.001	**<0.001**
Sex	-0.003	0.003	0.94	0.94	-0.054	0.004	0.20	0.35	-0.089	0.003	0.06	0.09	-0.040	0.005	0.34	0.61
**Optic Radiation**																
IL-10	-0.099	0.002	0.04	0.08	0.094	0.003	0.04	0.09	0.044	0.003	0.41	0.73	0.104	0.003	0.02	0.06
GA at delivery	0.016	0.001	0.76	0.85	-0.070	0.002	0.16	0.27	-0.070	0.002	0.23	0.51	-0.058	0.002	0.23	0.51
PMA at scan	0.538	0.001	<0.001	**<0.001**	-0.557	0.002	<0.001	**<0.001**	-0.288	0.002	<0.001	**<0.001**	-0.598	0.002	<0.001	**<0.001**
Sex	-0.004	0.003	0.93	0.94	-0.122	0.005	0.01	**0.03**	-0.186	0.005	<0.01	**<0.01**	-0.094	0.005	0.04	0.16
**Uncinate Fasciculus**																
IL-10	-0.051	0.001	0.30	0.33	-0.103	0.002	0.04	0.09	-0.151	0.003	0.01	0.05	-0.068	0.003	0.14	0.22
GA at delivery	0.104	0.001	0.05	0.11	0.085	0.002	0.11	0.27	0.174	0.002	<0.01	**0.03**	0.030	0.002	0.56	0.72
PMA at scan	0.482	0.001	<0.001	**<0.001**	-0.548	0.001	<0.001	**<0.001**	-0.275	0.002	<0.001	**<0.001**	-0.601	0.002	<0.001	**<0.001**
Sex	-0.055	0.002	0.26	0.59	-0.093	0.004	0.06	0.13	-0.144	0.005	0.01	**0.02**	-0.060	0.004	0.20	0.44
**Inferior Fronto-Occipital Fasciculus**																
IL-10	-0.110	0.002	0.01	**0.03**	0.046	0.003	0.28	0.36	-0.032	0.002	0.54	0.73	0.069	0.003	0.10	0.18
GA at delivery	0.122	0.001	0.01	0.08	-0.063	0.002	0.18	0.27	0.036	0.001	0.51	0.73	-0.082	0.002	0.07	0.21
PMA at scan	0.598	0.001	<0.001	**<0.001**	-0.630	0.002	<0.001	**<0.001**	-0.426	0.001	<0.001	**<0.001**	-0.641	0.002	<0.001	**<0.001**
Sex	-0.003	0.003	0.94	0.94	-0.113	0.004	0.01	**0.03**	-0.190	0.004	<0.001	**<0.01**	-0.080	0.005	0.06	0.17
**Anterior Limb of Internal Capsule**																
IL-10	-0.088	0.001	0.05	0.08	-0.002	0.003	0.97	0.97	-0.073	0.003	0.16	0.48	0.020	0.004	0.66	0.74
GA at delivery	0.105	0.001	0.03	0.08	-0.078	0.002	0.12	0.27	-0.019	0.002	0.73	0.80	-0.090	0.002	0.07	0.21
PMA at scan	0.594	0.001	<0.001	**<0.001**	-0.565	0.002	<0.001	**<0.001**	-0.418	0.002	<0.001	**<0.001**	-0.591	0.002	<0.001	**<0.001**
Sex	-0.031	0.002	0.48	0.86	-0.041	0.006	0.38	0.48	-0.065	0.005	0.22	0.22	-0.028	0.006	0.53	0.68
**Inferior Cingulum Bundle**																
IL-10	-0.137	0.001	0.01	**0.03**	0.108	0.002	0.02	0.09	0.019	0.002	0.73	0.73	0.133	0.002	0.00	**0.02**

GA at delivery	-0.136	0.001	0.02	0.08	0.100	0.001	0.05	0.27	-0.015	0.001	0.80	0.80	0.140	0.001	0.01	0.05
PMA at scan	0.409	0.001	<0.001	**<0.001**	-0.593	0.001	<0.001	**<0.001**	-0.312	0.001	<0.001	**<0.001**	-0.621	0.001	<0.001	**<0.001**
Sex	0.015	0.002	0.78	0.94	-0.134	0.003	<0.01	**0.03**	-0.114	0.003	0.04	0.07	-0.122	0.003	0.01	0.07
**Fornix**																
IL-10	0.082	0.001	0.13	0.17	0.039	0.002	0.39	0.44	0.100	0.002	0.05	0.21	0.002	0.002	0.97	0.97
GA at delivery	0.019	0.001	0.74	0.85	0.024	0.001	0.63	0.63	0.046	0.001	0.39	0.71	0.015	0.001	0.76	0.84
PMA at scan	0.353	0.001	<0.001	**<0.001**	-0.631	0.001	<0.001	**<0.001**	-0.494	0.001	<0.001	**<0.001**	-0.641	0.001	<0.001	**<0.001**
Sex	-0.088	0.002	0.10	0.35	-0.034	0.003	0.45	0.51	-0.081	0.004	0.11	0.14	-0.004	0.003	0.92	0.92

TNF-α

	FA				MD				AD				RD			
	**β**	**SE**	***p***	***q***	**β**	**SE**	***p***	***q***	**β**	**SE**	***p***	***q***	**β**	**SE**	***p***	***q***
**Corpus Callosum**																
TNF-α	-0.015	0.002	0.77	0.96	-0.046	0.002	0.35	0.78	-0.072	0.002	0.21	0.47	-0.024	0.003	0.62	0.88
GA at delivery	0.053	0.001	0.33	0.50	0.026	0.001	0.61	0.69	0.090	0.001	0.15	0.40	0.000	0.002	1.00	1.00
PMA at scan	0.468	0.001	<0.001	**<0.001**	-0.561	0.001	<0.001	**<0.001**	-0.200	0.001	<0.01	**<0.01**	-0.557	0.002	<0.001	**<0.001**
Sex	-0.079	0.003	0.12	0.34	-0.020	0.003	0.67	0.67	-0.100	0.003	0.08	0.10	0.034	0.004	0.49	0.68
**Superior Cingulum Bundle**																
TNF-α	0.008	0.002	0.89	0.96	0.014	0.003	0.78	0.78	0.035	0.003	0.51	0.77	0.006	0.003	0.91	0.91
GA at delivery	0.106	0.001	0.07	0.13	-0.034	0.002	0.53	0.68	0.030	0.002	0.60	0.70	-0.060	0.002	0.27	0.48
PMA at scan	0.305	0.001	<0.001	**<0.001**	-0.491	0.002	<0.001	**<0.001**	-0.395	0.002	<0.001	**<0.001**	-0.466	0.002	<0.001	**<0.001**
Sex	-0.092	0.003	0.09	0.34	-0.048	0.004	0.34	0.48	-0.136	0.005	0.01	**0.02**	-0.001	0.005	0.98	0.98
**Corticospinal Tract**																
TNF-α	-0.043	0.002	0.37	0.96	0.020	0.003	0.64	0.78	-0.022	0.002	0.64	0.82	0.029	0.003	0.50	0.88
GA at delivery	0.008	0.001	0.88	0.90	0.055	0.002	0.23	0.35	0.082	0.001	0.10	0.40	0.038	0.002	0.42	0.62
PMA at scan	0.546	0.001	<0.001	**<0.001**	-0.703	0.002	<0.001	**<0.001**	-0.621	0.001	<0.001	**<0.001**	-0.681	0.002	<0.001	**<0.001**
Sex	-0.010	0.003	0.83	0.97	-0.053	0.004	0.21	0.38	-0.094	0.003	0.04	0.06	-0.036	0.005	0.40	0.68
**Optic Radiation**																
TNF-α	-0.028	0.002	0.56	0.96	0.013	0.003	0.77	0.78	0.002	0.003	0.97	0.97	0.020	0.003	0.66	0.88
GA at delivery	0.016	0.001	0.76	0.90	-0.077	0.002	0.12	0.26	-0.078	0.002	0.18	0.40	-0.067	0.002	0.17	0.38
PMA at scan	0.540	0.001	<0.001	**<0.001**	-0.563	0.002	<0.001	**<0.001**	-0.295	0.002	<0.001	**<0.001**	-0.601	0.002	<0.001	**<0.001**
Sex	-0.004	0.003	0.94	0.97	-0.128	0.005	0.01	**0.03**	-0.194	0.005	<0.001	**<0.01**	-0.096	0.005	0.03	0.15
**Uncinate Fasciculus**																
TNF-α	-0.125	0.002	0.01	0.09	-0.039	0.002	0.43	0.78	-0.134	0.003	0.02	0.14	0.008	0.003	0.86	0.91
GA at delivery	0.096	0.001	0.07	0.13	0.084	0.002	0.12	0.26	0.168	0.002	0.01	0.05	0.029	0.002	0.57	0.73
PMA at scan	0.491	0.001	<0.001	**<0.001**	-0.544	0.002	<0.001	**<0.001**	-0.266	0.002	<0.001	**<0.001**	-0.597	0.002	<0.001	**<0.001**
Sex	-0.070	0.002	0.15	0.34	-0.083	0.004	0.09	0.21	-0.143	0.005	0.01	**0.02**	-0.045	0.004	0.34	0.68
**Inferior Fronto-Occipital Fasciculus**																
TNF-α	0.013	0.002	0.76	0.96	-0.015	0.003	0.72	0.78	0.014	0.002	0.79	0.89	-0.017	0.003	0.69	0.88
GA at delivery	0.128	0.001	0.01	0.06	-0.069	0.002	0.14	0.26	0.033	0.001	0.55	0.70	-0.091	0.002	0.05	0.15
PMA at scan	0.597	0.001	<0.001	**<0.001**	-0.631	0.002	<0.001	**<0.001**	-0.433	0.001	<0.001	**<0.001**	-0.640	0.002	<0.001	**<0.001**
Sex	-0.002	0.003	0.96	0.97	-0.112	0.004	0.01	**0.03**	-0.186	0.004	<0.001	**<0.01**	-0.078	0.006	0.07	0.20
**Anterior Limb of Internal Capsule**																
TNF-α	0.021	0.002	0.64	0.96	-0.062	0.003	0.18	0.78	-0.068	0.003	0.19	0.47	-0.056	0.004	0.21	0.63

GA at delivery	0.113	0.001	0.02	0.06	-0.087	0.002	0.08	0.26	-0.026	0.002	0.65	0.70	-0.100	0.002	0.04	0.15
PMA at scan	0.596	0.001	<0.001	**<0.001**	-0.563	0.002	<0.001	**<0.001**	-0.416	0.002	<0.001	**<0.001**	-0.589	0.002	<0.001	**<0.001**
Sex	-0.027	0.002	0.53	0.96	-0.041	0.005	0.37	0.48	-0.063	0.005	0.23	0.23	-0.028	0.006	0.53	0.68
**Inferior Cingulum Bundle**																
TNF-α	-0.123	0.001	0.02	0.09	0.023	0.002	0.62	0.78	-0.060	0.002	0.27	0.49	0.058	0.002	0.21	0.63
GA at delivery	-0.139	0.001	0.02	0.06	0.094	0.001	0.07	0.26	-0.022	0.001	0.70	0.70	0.135	0.001	0.01	0.07
PMA at scan	0.424	0.001	<0.001	**<0.001**	-0.601	0.001	<0.001	**<0.001**	-0.312	0.001	<0.001	**<0.001**	-0.632	0.001	<0.001	**<0.001**
Sex	-0.002	0.002	0.97	0.97	-0.130	0.003	0.01	**0.03**	-0.121	0.003	0.03	0.05	-0.112	0.003	0.02	0.14
**Fornix**																
TNF-α	-0.003	0.001	0.96	0.96	-0.075	0.002	0.10	0.78	-0.071	0.002	0.16	0.47	-0.065	0.002	0.15	0.63
GA at delivery	0.007	0.001	0.90	0.90	0.013	0.001	0.79	0.79	0.029	0.001	0.60	0.70	0.005	0.001	0.92	1.00
PMA at scan	0.347	0.001	<0.001	**<0.001**	-0.629	0.001	<0.001	**<0.001**	-0.495	0.001	<0.001	**<0.001**	-0.635	0.001	<0.001	**<0.001**
Sex	-0.101	0.002	0.06	0.34	-0.034	0.003	0.46	0.51	-0.087	0.004	0.09	0.10	0.004	0.003	0.93	0.98

Bolded values represent statistical significance after FDR correction for multiple comparisons. β, standardized beta coefficient; SE, standard error; *q*, FDR-corrected p-value; IL, interleukin; TNF-α, tumor necrosis factor alpha; FA, fractional anisotropy; MD, mean diffusivity; AD, axial diffusivity; RD, radial diffusivity; GA, gestational age; PMA, infant postmenstrual age; Sex, child sex.

Excluding neonates born <34 weeks' gestation and/or weighing <2,000g at birth, we investigated the moderating role of family SES group in the relationship between maternal cytokine concentrations and neonatal dMRI parameters. As shown in Table S8, family SES moderated the relationship between maternal TNF-α and CB AD (β = 0.21; *q* = 0.02) and CBIF AD (β = 0.20; *q* = 0.02).

Table S10. Specificity test excluding neonates born <34 weeks' gestation and/or weighing <2,000g at birth. Multiple linear regression results of the moderating role of family SES group in the relationship between maternal cytokine concentrations and neonatal dMRI parameters.

IL-6																
	FA				MD				AD				RD			
	β	SE	*p*	*q*	β	SE	*p*	*q*	β	SE	*p*	*q*	β	SE	*p*	*q*
**Corpus Callosum**																
IL-6	-0.020	0.002	0.76	0.85	-0.090	0.002	0.14	0.26	-0.096	0.002	0.19	0.20	-0.052	0.003	0.39	0.53
SES group	0.080	0.003	0.12	0.37	-0.018	0.003	0.72	0.72	0.126	0.004	0.03	0.05	-0.053	0.004	0.28	0.28
GA at delivery	0.028	0.001	0.62	0.93	0.039	0.001	0.47	0.63	0.072	0.001	0.26	0.73	0.019	0.002	0.72	0.79
PMA at scan	0.468	0.001	<0.001	**<0.001**	-0.574	0.001	<0.001	**<0.001**	-0.224	0.001	<0.001	**<0.001**	-0.565	0.002	<0.001	**<0.001**
Sex	-0.081	0.003	0.11	0.27	-0.008	0.003	0.87	0.87	-0.083	0.003	0.15	0.18	0.042	0.004	0.38	0.82
IL-6:SES group	-0.089	0.004	0.16	0.72	0.120	0.004	0.05	0.45	0.013	0.004	0.86	0.86	0.118	0.005	0.05	0.46
**Superior Cingulum Bundle**																
IL-6	0.026	0.002	0.71	0.85	-0.100	0.003	0.11	0.26	-0.087	0.004	0.20	0.20	-0.090	0.004	0.15	0.51
SES group	-0.140	0.003	0.01	0.12	0.144	0.005	0.01	**0.02**	0.050	0.005	0.36	0.36	0.165	0.005	<0.01	**0.01**
GA at delivery	0.144	0.001	0.02	0.05	-0.069	0.002	0.21	0.48	0.018	0.002	0.76	0.90	-0.101	0.002	0.07	0.13
PMA at scan	0.317	0.001	<0.001	**<0.001**	-0.504	0.002	<0.001	**<0.001**	-0.400	0.002	<0.001	**<0.001**	-0.480	0.002	<0.001	**<0.001**
Sex	-0.103	0.003	0.06	0.27	-0.037	0.004	0.46	0.60	-0.131	0.005	0.02	0.05	0.010	0.005	0.84	0.94
IL-6:SES group	0.057	0.003	0.40	0.88	0.073	0.005	0.25	0.80	0.118	0.006	0.08	0.44	0.040	0.006	0.52	0.75
**Corticospinal Tract**																
IL-6	-0.164	0.002	0.01	0.06	0.034	0.003	0.52	0.67	-0.095	0.002	0.10	0.14	0.074	0.004	0.17	0.51
SES group	0.011	0.003	0.82	0.92	0.112	0.004	0.01	**0.02**	0.199	0.003	<0.001	**<0.001**	0.075	0.005	0.09	0.10
GA at delivery	0.010	0.001	0.86	0.96	0.022	0.002	0.63	0.63	0.032	0.001	0.52	0.77	0.013	0.002	0.78	0.79
PMA at scan	0.543	0.001	<0.001	**<0.001**	-0.714	0.001	<0.001	**<0.001**	-0.647	0.001	<0.001	**<0.001**	-0.688	0.002	<0.001	**<0.001**
Sex	-0.005	0.003	0.91	1.00	-0.044	0.004	0.30	0.53	-0.072	0.003	0.11	0.16	-0.032	0.005	0.45	0.82
IL-6:SES group	-0.041	0.003	0.49	0.88	0.044	0.005	0.40	0.80	0.017	0.004	0.76	0.86	0.049	0.006	0.36	0.75
**Optic Radiation**																
IL-6	-0.105	0.002	0.09	0.20	-0.022	0.003	0.70	0.77	-0.104	0.003	0.11	0.14	0.016	0.004	0.78	0.78
SES group	0.068	0.003	0.18	0.41	0.161	0.005	<0.01	**<0.01**	0.276	0.005	<0.001	**<0.001**	0.105	0.006	0.02	0.05
GA at delivery	-0.001	0.001	0.98	0.98	-0.124	0.002	0.02	0.12	-0.156	0.002	0.01	0.06	-0.097	0.002	0.05	0.13
PMA at scan	0.534	0.001	<0.001	**<0.001**	-0.573	0.002	<0.001	**<0.001**	-0.315	0.002	<0.001	**<0.001**	-0.606	0.002	<0.001	**<0.001**
Sex	0.002	0.003	0.97	1.00	-0.118	0.005	0.01	0.06	-0.180	0.005	<0.01	<0.01	-0.089	0.006	0.05	0.22
IL-6:SES group	0.024	0.004	0.70	0.88	-0.021	0.006	0.71	0.80	-0.013	0.006	0.84	0.86	-0.025	0.007	0.66	0.75
**Uncinate Fasciculus**																
IL-6	-0.130	0.002	0.04	0.11	-0.098	0.003	0.11	0.26	-0.195	0.004	<0.01	**0.04**	-0.043	0.003	0.47	0.53
SES group	-0.053	0.003	0.29	0.52	0.109	0.004	0.03	**0.04**	0.083	0.005	0.14	0.18	0.103	0.005	0.03	0.05
GA at delivery	0.108	0.001	0.05	0.08	0.036	0.002	0.50	0.63	0.125	0.002	0.04	0.17	-0.014	0.002	0.79	0.79
PMA at scan	0.496	0.001	<0.001	**<0.001**	-0.567	0.001	<0.001	**<0.001**	-0.284	0.002	<0.001	**<0.001**	-0.615	0.002	<0.001	**<0.001**

Sex	-0.080	0.002	0.10	0.27	-0.056	0.004	0.25	0.53	-0.122	0.005	0.02	0.06	-0.020	0.004	0.67	0.88
IL-6:SES group	-0.002	0.003	0.98	0.98	-0.062	0.005	0.31	0.80	-0.071	0.006	0.30	0.86	-0.046	0.005	0.44	0.75
**Inferior Fronto-Occipital Fasciculus**																
IL-6	-0.019	0.002	0.74	0.85	-0.065	0.003	0.23	0.35	-0.128	0.003	0.05	0.10	-0.040	0.004	0.46	0.53
SES group	0.000	0.003	1.00	1.00	0.118	0.005	0.01	**0.02**	0.221	0.004	<0.001	**<0.001**	0.085	0.006	0.05	0.08
GA at delivery	0.139	0.001	0.01	0.05	-0.098	0.002	0.04	0.12	-0.005	0.001	0.92	0.92	-0.115	0.002	0.02	0.09
PMA at scan	0.594	0.001	<0.001	**<0.001**	-0.643	0.002	<0.001	**<0.001**	-0.459	0.001	<0.001	**<0.001**	-0.646	0.002	<0.001	**<0.001**
Sex	0.000	0.003	1.00	1.00	-0.101	0.005	0.02	0.06	-0.168	0.003	<0.01	**<0.01**	-0.070	0.006	0.10	0.30
IL-6:SES group	-0.026	0.004	0.64	0.88	0.037	0.005	0.50	0.80	0.040	0.004	0.53	0.86	0.033	0.007	0.54	0.75
**Anterior Limb of Internal Capsule**																
IL-6	0.003	0.002	0.96	0.96	-0.097	0.004	0.10	0.26	-0.142	0.003	0.03	0.10	-0.079	0.005	0.17	0.51
SES group	0.014	0.003	0.76	0.92	0.102	0.006	0.03	**0.04**	0.151	0.005	0.01	**0.01**	0.079	0.007	0.09	0.10
GA at delivery	0.120	0.001	0.02	0.05	-0.106	0.002	0.04	0.12	-0.047	0.002	0.42	0.75	-0.118	0.002	0.02	0.09
PMA at scan	0.596	0.001	<0.001	**<0.001**	-0.576	0.002	<0.001	**<0.001**	-0.437	0.002	<0.001	**<0.001**	-0.598	0.002	<0.001	**<0.001**
Sex	-0.029	0.003	0.51	0.92	-0.030	0.006	0.52	0.60	-0.049	0.005	0.34	0.34	-0.019	0.006	0.68	0.88
IL-6:SES group	0.016	0.003	0.78	0.88	0.022	0.007	0.70	0.80	0.047	0.006	0.47	0.86	0.014	0.008	0.81	0.81
**Inferior Cingulum Bundle**																
IL-6	-0.143	0.002	0.04	0.11	-0.017	0.002	0.77	0.77	-0.118	0.002	0.09	0.14	0.046	0.002	0.43	0.53
SES group	-0.049	0.002	0.38	0.57	0.189	0.003	<0.001	**<0.01**	0.159	0.004	<0.01	**0.01**	0.154	0.003	<0.01	**0.01**
GA at delivery	-0.123	0.001	0.04	0.08	0.050	0.001	0.34	0.61	-0.060	0.001	0.32	0.73	0.097	0.001	0.06	0.13
PMA at scan	0.425	0.001	<0.001	**<0.001**	-0.619	0.001	<0.001	**<0.001**	-0.327	0.001	<0.001	**<0.001**	-0.644	0.001	<0.001	**<0.001**
Sex	-0.003	0.002	0.96	1.00	-0.114	0.003	0.01	0.06	-0.103	0.003	0.06	0.10	-0.098	0.003	0.03	0.22
IL-6:SES group	0.046	0.003	0.49	0.88	-0.006	0.003	0.92	0.92	0.032	0.004	0.64	0.86	-0.047	0.003	0.42	0.75
**Fornix**																
IL-6	-0.085	0.001	0.22	0.39	-0.085	0.002	0.14	0.26	-0.133	0.003	0.04	0.10	-0.043	0.002	0.45	0.53
SES group	-0.118	0.002	0.04	0.16	0.102	0.003	0.03	**0.04**	0.055	0.004	0.30	0.33	0.121	0.003	0.01	**0.03**
GA at delivery	0.014	0.001	0.82	0.96	-0.027	0.001	0.60	0.63	-0.014	0.001	0.80	0.90	-0.028	0.001	0.57	0.79
PMA at scan	0.366	0.001	<0.001	**<0.001**	-0.629	0.001	<0.001	**<0.001**	-0.489	0.001	<0.001	**<0.001**	-0.639	0.001	<0.001	**<0.001**
Sex	-0.085	0.002	0.12	0.27	-0.028	0.003	0.53	0.60	-0.072	0.004	0.16	0.18	0.002	0.003	0.96	0.96
IL-6:SES group	0.128	0.002	0.06	0.55	0.025	0.003	0.66	0.80	0.106	0.005	0.10	0.44	-0.024	0.003	0.67	0.75
**IL-8**																
	**FA**				**MD**				**AD**				**RD**			
	**β**	**SE**	***p***	***q***	**β**	**SE**	***p***	***q***	**β**	**SE**	***p***	***q***	**β**	**SE**	***p***	***q***
**Corpus Callosum**																
IL-8	0.001	0.002	0.99	0.99	0.008	0.002	0.90	0.95	0.024	0.003	0.74	0.90	0.018	0.003	0.77	0.98
SES group	0.094	0.003	0.07	0.20	-0.012	0.003	0.82	0.82	0.151	0.004	0.01	**0.02**	-0.056	0.004	0.26	0.26
GA at delivery	0.038	0.001	0.50	0.64	0.051	0.001	0.35	0.52	0.095	0.001	0.13	0.40	0.024	0.002	0.65	0.94
PMA at scan	0.468	0.001	<0.001	**<0.001**	-0.576	0.001	<0.001	**<0.001**	-0.224	0.001	<0.001	**<0.001**	-0.567	0.002	<0.001	**<0.001**
Sex	-0.084	0.003	0.09	0.24	-0.014	0.003	0.77	0.77	-0.095	0.003	0.10	0.13	0.040	0.004	0.41	0.76
IL-8:SES group	-0.020	0.004	0.76	0.98	-0.037	0.004	0.56	0.94	-0.072	0.004	0.33	0.63	-0.026	0.005	0.67	0.87
**Superior Cingulum Bundle**																

IL-8	-0.058	0.002	0.41	0.77	0.059	0.003	0.36	0.95	0.015	0.004	0.83	0.90	0.074	0.004	0.25	0.98
SES group	-0.151	0.003	0.01	0.06	0.156	0.005	<0.01	**<0.01**	0.056	0.005	0.30	0.30	0.177	0.005	<0.01	**<0.01**
GA at delivery	0.127	0.001	0.03	0.08	-0.046	0.002	0.41	0.52	0.030	0.002	0.61	0.80	-0.076	0.002	0.17	0.31
PMA at scan	0.314	0.001	<0.001	**<0.001**	-0.504	0.002	<0.001	**<0.001**	-0.402	0.002	<0.001	**<0.001**	-0.479	0.002	<0.001	**<0.001**
Sex	-0.098	0.003	0.07	0.24	-0.043	0.004	0.39	0.55	-0.134	0.005	0.01	**0.03**	0.003	0.005	0.94	0.94
IL-8:SES group	0.080	0.003	0.25	0.75	-0.051	0.005	0.43	0.94	0.012	0.006	0.86	0.86	-0.075	0.006	0.24	0.87
**Corticospinal Tract**																
IL-8	0.028	0.002	0.66	0.77	0.021	0.003	0.70	0.95	0.046	0.003	0.43	0.90	0.009	0.004	0.87	0.98
SES group	0.036	0.003	0.47	0.70	0.111	0.004	0.01	**0.02**	0.220	0.004	<0.001	**<0.001**	0.066	0.005	0.13	0.15
GA at delivery	0.041	0.001	0.44	0.64	0.021	0.002	0.64	0.72	0.059	0.001	0.23	0.53	0.004	0.002	0.94	1.00
PMA at scan	0.538	0.001	<0.001	**<0.001**	-0.709	0.002	<0.001	**<0.001**	-0.642	0.001	<0.001	**<0.001**	-0.683	0.002	<0.001	**<0.001**
Sex	-0.009	0.003	0.85	0.92	-0.049	0.004	0.25	0.44	-0.084	0.003	0.06	0.09	-0.035	0.005	0.42	0.76
IL-8:SES group	-0.089	0.004	0.15	0.75	0.006	0.005	0.91	0.94	-0.076	0.004	0.19	0.63	0.033	0.006	0.55	0.87
**Optic Radiation**																
IL-8	0.027	0.002	0.67	0.77	0.043	0.004	0.46	0.95	0.047	0.004	0.48	0.90	0.024	0.004	0.68	0.98
SES group	0.073	0.003	0.14	0.33	0.176	0.005	<0.001	**<0.01**	0.301	0.005	<0.001	**<0.001**	0.115	0.006	0.01	**0.02**
GA at delivery	0.014	0.001	0.80	0.80	-0.106	0.002	0.04	0.31	-0.124	0.002	0.03	0.14	-0.088	0.002	0.08	0.17
PMA at scan	0.530	0.001	<0.001	**<0.001**	-0.568	0.002	<0.001	**<0.001**	-0.312	0.002	<0.001	**<0.001**	-0.602	0.002	<0.001	**<0.001**
Sex	0.005	0.003	0.92	0.92	-0.127	0.005	0.01	**0.04**	-0.190	0.005	<0.001	**<0.01**	-0.097	0.006	0.03	0.15
IL-8:SES group	-0.045	0.004	0.48	0.98	-0.021	0.006	0.73	0.94	-0.043	0.006	0.51	0.66	-0.002	0.007	0.97	0.97
**Uncinate Fasciculus**																
IL-8	0.059	0.002	0.35	0.77	0.004	0.003	0.95	0.95	0.044	0.004	0.54	0.90	-0.017	0.003	0.78	0.98
SES group	-0.039	0.003	0.44	0.70	0.139	0.004	0.01	**0.01**	0.125	0.005	0.03	**0.04**	0.123	0.004	0.01	**0.02**
GA at delivery	0.135	0.001	0.01	0.06	0.063	0.002	0.24	0.51	0.172	0.002	0.01	0.05	0.000	0.002	1.00	1.00
PMA at scan	0.493	0.001	<0.001	**<0.001**	-0.570	0.001	<0.001	**<0.001**	-0.290	0.002	<0.001	**<0.001**	-0.617	0.002	<0.001	**<0.001**
Sex	-0.079	0.002	0.11	0.24	-0.068	0.004	0.16	0.37	-0.135	0.005	0.02	**0.03**	-0.030	0.004	0.52	0.76
IL-8:SES group	0.002	0.003	0.98	0.98	-0.095	0.005	0.13	0.94	-0.106	0.006	0.14	0.63	-0.075	0.005	0.21	0.87
**Inferior Fronto-Occipital Fasciculus**																
IL-8	0.023	0.002	0.68	0.77	0.013	0.004	0.81	0.95	0.051	0.003	0.43	0.90	0.001	0.004	0.99	0.98
SES group	-0.002	0.003	0.96	0.96	0.131	0.005	<0.01	**0.01**	0.239	0.004	<0.001	**<0.001**	0.095	0.006	0.03	**0.04**
GA at delivery	0.141	0.001	<0.01	**0.04**	-0.082	0.002	0.08	0.31	0.023	0.001	0.69	0.80	-0.105	0.002	0.03	0.17
PMA at scan	0.595	0.001	<0.001	**<0.001**	-0.644	0.002	<0.001	**<0.001**	-0.461	0.001	<0.001	**<0.001**	-0.648	0.002	<0.001	**<0.001**
Sex	0.005	0.003	0.91	0.92	-0.109	0.005	0.01	**0.04**	-0.174	0.004	<0.01	**<0.01**	-0.077	0.006	0.07	0.21
IL-8:SES group	-0.005	0.004	0.94	0.98	-0.033	0.006	0.55	0.94	-0.061	0.004	0.35	0.63	-0.023	0.007	0.68	0.87
**Anterior Limb of Internal Capsule**																

IL-8	-0.036	0.002	0.53	0.77	0.034	0.004	0.57	0.95	-0.010	0.004	0.88	0.90	0.039	0.005	0.51	0.98
SES group	0.013	0.003	0.77	0.87	0.119	0.006	0.01	**0.02**	0.176	0.005	<0.01	**<0.01**	0.093	0.007	0.04	0.06
GA at delivery	0.114	0.001	0.02	0.06	-0.084	0.002	0.10	0.31	-0.021	0.002	0.71	0.80	-0.098	0.002	0.05	0.17
PMA at scan	0.594	0.001	<0.001	**<0.001**	-0.579	0.002	<0.001	**<0.001**	-0.443	0.002	<0.001	**<0.001**	-0.601	0.002	<0.001	**<0.001**
Sex	-0.029	0.002	0.51	0.92	-0.037	0.006	0.43	0.55	-0.060	0.005	0.25	0.25	-0.024	0.006	0.59	0.76
IL-8:SES group	0.014	0.003	0.80	0.98	-0.048	0.007	0.43	0.94	-0.044	0.006	0.52	0.66	-0.042	0.008	0.47	0.87
**Inferior Cingulum Bundle**																
IL-8	-0.070	0.002	0.32	0.77	-0.024	0.002	0.69	0.95	-0.075	0.003	0.29	0.90	0.009	0.002	0.87	0.98
SES group	-0.027	0.002	0.62	0.80	0.201	0.003	<0.001	**<0.001**	0.187	0.004	<0.01	**<0.01**	0.159	0.003	<0.01	**<0.01**
GA at delivery	-0.106	0.001	0.08	0.14	0.055	0.001	0.28	0.51	-0.042	0.001	0.49	0.80	0.096	0.001	0.06	0.17
PMA at scan	0.421	0.001	<0.001	**<0.001**	-0.619	0.001	<0.001	**<0.001**	-0.330	0.001	<0.001	**<0.001**	-0.644	0.001	<0.001	**<0.001**
Sex	-0.007	0.002	0.90	0.92	-0.120	0.003	0.01	**0.04**	-0.113	0.003	0.04	0.07	-0.103	0.003	0.03	0.15
IL-8:SES group	0.090	0.003	0.19	0.75	0.013	0.003	0.82	0.94	0.073	0.004	0.29	0.63	-0.026	0.003	0.66	0.87
**Fornix**																
IL-8	-0.036	0.001	0.61	0.77	0.024	0.002	0.68	0.95	-0.008	0.003	0.90	0.90	0.039	0.002	0.51	0.98
SES group	-0.107	0.002	0.05	0.20	0.113	0.003	0.02	**0.02**	0.072	0.004	0.17	0.19	0.127	0.003	0.01	**0.02**
GA at delivery	0.022	0.001	0.72	0.80	-0.011	0.001	0.83	0.83	0.005	0.001	0.93	0.93	-0.017	0.001	0.73	0.94
PMA at scan	0.353	0.001	<0.001	**<0.001**	-0.632	0.001	<0.001	**<0.001**	-0.492	0.001	<0.001	**<0.001**	-0.638	0.001	<0.001	**<0.001**
Sex	-0.092	0.002	0.09	0.24	-0.030	0.003	0.51	0.57	-0.079	0.004	0.12	0.14	0.004	0.003	0.92	0.94
IL-8:SES group	0.009	0.002	0.90	0.98	0.004	0.003	0.94	0.94	0.018	0.005	0.78	0.86	-0.004	0.003	0.95	0.97
**IL-10**																
	FA				MD				AD				RD			
	β	SE	*p*	*q*	β	SE	*p*	*q*	β	SE	*p*	*q*	β	SE	*p*	*q*
**Corpus Callosum**																
IL-10	-0.065	0.002	0.32	0.36	0.050	0.002	0.43	0.55	-0.075	0.003	0.32	0.57	0.067	0.003	0.28	0.51
SES group	0.101	0.003	0.05	0.18	-0.011	0.003	0.82	0.82	0.160	0.004	0.01	**0.01**	-0.063	0.004	0.20	0.20
GA at delivery	0.041	0.001	0.46	0.68	0.048	0.001	0.37	0.56	0.090	0.001	0.16	0.48	0.027	0.002	0.61	0.86
PMA at scan	0.475	0.001	<0.001	**<0.001**	-0.573	0.001	<0.001	**<0.001**	-0.210	0.001	<0.001	**<0.001**	-0.576	0.002	<0.001	**<0.001**
Sex	-0.091	0.003	0.07	0.32	-0.006	0.003	0.89	0.89	-0.092	0.003	0.11	0.14	0.042	0.004	0.38	0.73
IL-10:SES group	-0.021	0.004	0.74	0.92	0.054	0.004	0.40	0.78	0.109	0.004	0.15	0.79	0.032	0.005	0.61	0.81
**Superior Cingulum Bundle**																
IL-10	-0.113	0.002	0.11	0.18	0.084	0.003	0.20	0.45	0.034	0.004	0.63	0.71	0.103	0.004	0.12	0.27
SES group	-0.152	0.003	0.01	0.06	0.161	0.005	<0.01	**0.01**	0.061	0.005	0.27	0.27	0.181	0.005	<0.001	**<0.01**
GA at delivery	0.120	0.001	0.05	0.10	-0.043	0.002	0.44	0.57	0.030	0.002	0.61	0.81	-0.069	0.002	0.21	0.39
PMA at scan	0.319	0.001	<0.001	**<0.001**	-0.500	0.002	<0.001	**<0.001**	-0.394	0.002	<0.001	**<0.001**	-0.480	0.002	<0.001	**<0.001**
Sex	-0.096	0.003	0.08	0.32	-0.050	0.004	0.32	0.48	-0.139	0.005	0.01	**0.03**	-0.004	0.005	0.93	0.76
IL-10:SES group	0.118	0.003	0.10	0.87	-0.018	0.005	0.78	0.78	0.062	0.006	0.38	0.79	-0.058	0.006	0.38	0.81

**Corticospinal Tract**																
IL-10	-0.214	0.002	0.00	0.01	0.137	0.003	0.01	0.11	0.004	0.003	0.94	0.94	0.166	0.004	<0.01	**0.03**
SES group	0.035	0.003	0.46	0.63	0.116	0.004	0.01	**0.01**	0.225	0.004	<0.001	**<0.001**	0.069	0.005	0.11	0.12
GA at delivery	0.025	0.001	0.63	0.71	0.029	0.002	0.52	0.58	0.056	0.001	0.27	0.60	0.016	0.002	0.73	0.86
PMA at scan	0.532	0.001	<0.001	**<0.001**	-0.701	0.001	<0.001	**<0.001**	-0.635	0.001	<0.001	**<0.001**	-0.675	0.002	<0.001	**<0.001**
Sex	-0.008	0.003	0.86	0.99	-0.047	0.004	0.26	0.46	-0.081	0.003	0.08	0.11	-0.034	0.005	0.42	0.62
IL-10:SES group	0.006	0.003	0.92	0.92	0.017	0.005	0.75	0.78	0.033	0.004	0.58	0.79	0.014	0.006	0.80	0.87
**Optic Radiation**																
IL-10	-0.106	0.002	0.10	0.18	0.119	0.004	0.05	0.14	0.073	0.004	0.28	0.57	0.127	0.004	0.03	0.09
SES group	0.078	0.003	0.12	0.27	0.177	0.005	<0.001	**<0.01**	0.305	0.005	<0.001	**<0.001**	0.112	0.006	0.01	**0.03**
GA at delivery	0.007	0.001	0.89	0.89	-0.101	0.002	0.05	0.28	-0.118	0.002	0.04	0.17	-0.079	0.002	0.11	0.25
PMA at scan	0.531	0.001	<0.001	**<0.001**	-0.570	0.002	<0.001	**<0.001**	-0.313	0.002	<0.001	**<0.001**	-0.606	0.002	<0.001	**<0.001**
Sex	-0.001	0.003	0.98	0.99	-0.124	0.005	0.01	**0.03**	-0.191	0.005	<0.001	**<0.01**	-0.095	0.006	0.03	0.16
IL-10:SES group	0.017	0.004	0.79	0.92	-0.051	0.006	0.39	0.78	-0.055	0.006	0.41	0.79	-0.045	0.007	0.44	0.81
**Uncinate Fasciculus**																
IL-10	-0.035	0.002	0.59	0.59	-0.066	0.003	0.31	0.55	-0.103	0.004	0.15	0.55	-0.041	0.003	0.51	0.65
SES group	-0.041	0.003	0.42	0.63	0.142	0.004	<0.001	**0.01**	0.127	0.005	0.02	**0.03**	0.124	0.004	0.01	**0.02**
GA at delivery	0.126	0.001	0.02	0.07	0.066	0.002	0.22	0.40	0.168	0.002	0.01	0.05	0.008	0.002	0.88	0.88
PMA at scan	0.481	0.001	<0.001	**<0.001**	-0.567	0.001	<0.001	**<0.001**	-0.293	0.002	<0.001	**<0.001**	-0.615	0.002	<0.001	**<0.001**
Sex	-0.072	0.003	0.14	0.32	-0.078	0.004	0.11	0.26	-0.140	0.005	0.01	**0.03**	-0.042	0.004	0.37	0.62
IL-10:SES group	-0.044	0.003	0.49	0.92	-0.052	0.005	0.42	0.78	-0.084	0.006	0.24	0.79	-0.030	0.005	0.63	0.81
**Inferior Fronto-Occipital Fasciculus**																
IL-10	-0.089	0.002	0.12	0.18	0.030	0.004	0.59	0.66	-0.038	0.003	0.56	0.71	0.049	0.004	0.38	0.57
SES group	0.000	0.003	0.99	0.99	0.134	0.005	<0.01	**0.01**	0.247	0.004	<0.001	**<0.001**	0.095	0.006	0.03	**0.04**
GA at delivery	0.136	0.001	<0.01	**0.04**	-0.081	0.002	0.09	0.28	0.020	0.001	0.72	0.81	-0.101	0.002	0.03	0.15
PMA at scan	0.591	0.001	<0.001	**<0.001**	-0.638	0.002	<0.001	**<0.001**	-0.454	0.001	<0.001	**<0.001**	-0.643	0.002	<0.001	**<0.001**
Sex	0.000	0.003	0.99	0.99	-0.110	0.005	0.01	**0.03**	-0.182	0.004	<0.001	**<0.01**	-0.078	0.006	0.07	0.20
IL-10:SES group	-0.037	0.004	0.51	0.92	0.032	0.006	0.57	0.78	0.017	0.004	0.80	0.79	0.038	0.007	0.49	0.81
**Anterior Limb of Internal Capsule**																
IL-10	-0.098	0.002	0.09	0.18	-0.006	0.004	0.92	0.92	-0.091	0.004	0.18	0.55	0.019	0.005	0.75	0.75
SES group	0.012	0.003	0.79	0.89	0.122	0.006	0.01	**0.01**	0.177	0.005	<0.01	**<0.01**	0.095	0.007	0.04	0.05
GA at delivery	0.111	0.001	0.02	0.07	-0.087	0.002	0.09	0.28	-0.025	0.002	0.67	0.81	-0.099	0.003	0.05	0.15
PMA at scan	0.594	0.001	<0.001	**<0.001**	-0.576	0.002	<0.001	**<0.001**	-0.436	0.002	<0.001	**<0.001**	-0.599	0.002	<0.001	**<0.001**
Sex	-0.031	0.003	0.49	0.88	-0.039	0.006	0.41	0.53	-0.063	0.005	0.23	0.23	-0.026	0.006	0.57	0.73
IL-10:SES group	0.021	0.003	0.72	0.92	0.018	0.007	0.77	0.78	0.047	0.006	0.49	0.79	0.009	0.008	0.87	0.87
**Inferior Cingulum Bundle**																
IL-10	-0.130	0.002	0.07	0.18	0.133	0.002	0.03	0.13	0.051	0.003	0.47	0.71	0.158	0.002	0.01	**0.04**
SES group	-0.038	0.002	0.49	0.63	0.200	0.003	<0.001	**<0.001**	0.178	0.004	<0.01	**<0.01**	0.162	0.003	<0.01	**<0.01**
GA at delivery	-0.113	0.001	0.06	0.10	0.067	0.001	0.19	0.40	-0.034	0.001	0.57	0.81	0.109	0.001	0.03	0.15

PMA at scan	0.410	0.001	<0.001	**<0.001**	-0.613	0.001	<0.001	**<0.001**	-0.332	0.001	<0.001	**<0.001**	-0.636	0.001	<0.001	**<0.001**
Sex	0.001	0.002	0.99	0.99	-0.123	0.003	0.01	**0.03**	-0.110	0.003	0.04	0.08	-0.108	0.003	0.02	0.16
IL-10:SES group	-0.010	0.003	0.89	0.92	-0.026	0.003	0.67	0.78	-0.036	0.004	0.62	0.79	-0.031	0.003	0.60	0.81
**Fornix**																
IL-10	0.083	0.001	0.24	0.31	0.053	0.002	0.38	0.55	0.092	0.003	0.17	0.55	0.022	0.002	0.71	0.75
SES group	-0.105	0.002	0.06	0.18	0.112	0.003	0.02	**0.02**	0.073	0.004	0.16	0.18	0.122	0.003	0.01	**0.02**
GA at delivery	0.032	0.001	0.59	0.71	-0.010	0.001	0.84	0.84	0.011	0.001	0.85	0.85	-0.015	0.001	0.76	0.86
PMA at scan	0.359	0.001	<0.001	**<0.001**	-0.633	0.001	<0.001	**<0.001**	-0.489	0.001	<0.001	**<0.001**	-0.646	0.001	<0.001	**<0.001**
Sex	-0.085	0.002	0.12	0.32	-0.028	0.003	0.54	0.61	-0.072	0.004	0.16	0.18	0.000	0.003	0.99	0.99
IL-10:SES group	0.016	0.002	0.82	0.92	-0.023	0.003	0.70	0.78	0.020	0.005	0.77	0.80	-0.039	0.004	0.51	0.81

TNF-α

	FA				MD				AD				RD			
	**β**	**SE**	***p***	***q***	**β**	**SE**	***p***	***q***	**β**	**SE**	***p***	***q***	**β**	**SE**	***p***	***q***
**Corpus Callosum**																
TNF-α	0.043	0.002	0.50	0.91	-0.116	0.003	0.07	0.20	-0.124	0.003	0.09	0.17	-0.088	0.003	0.16	0.36
SES group	0.103	0.003	0.05	0.17	-0.008	0.003	0.87	0.87	0.166	0.003	<0.01	**0.01**	-0.056	0.004	0.25	0.25
GA at delivery	0.037	0.001	0.51	0.72	0.043	0.001	0.42	0.54	0.088	0.001	0.17	0.50	0.018	0.002	0.73	0.94
PMA at scan	0.466	0.001	<0.001	**<0.001**	-0.569	0.001	<0.001	**<0.001**	-0.220	0.001	<0.001	**<0.001**	-0.563	0.002	<0.001	**<0.001**
Sex	-0.088	0.003	0.08	0.20	-0.018	0.003	0.72	0.72	-0.107	0.003	0.06	0.08	0.040	0.004	0.41	0.73
TNF-α:SES group	-0.073	0.004	0.26	0.64	0.084	0.004	0.18	0.27	0.066	0.004	0.37	0.48	0.074	0.005	0.24	0.35
**Superior Cingulum Bundle**																
TNF-α	0.010	0.002	0.89	0.94	-0.104	0.003	0.10	0.23	-0.105	0.004	0.12	0.18	-0.082	0.004	0.21	0.37
SES group	-0.153	0.003	0.01	0.06	0.156	0.004	<0.01	**<0.01**	0.053	0.005	0.33	0.33	0.179	0.005	<0.01	**<0.01**
GA at delivery	0.134	0.001	0.03	0.07	-0.046	0.002	0.41	0.54	0.038	0.002	0.52	0.77	-0.079	0.002	0.15	0.27
PMA at scan	0.312	0.001	<0.001	**<0.001**	-0.495	0.002	<0.001	**<0.001**	-0.391	0.002	<0.001	**<0.001**	-0.472	0.002	<0.001	**<0.001**
Sex	-0.101	0.003	0.06	0.20	-0.047	0.004	0.34	0.47	-0.141	0.005	0.01	**0.02**	0.002	0.005	0.97	0.97
TNF-α:SES group	0.017	0.003	0.81	0.86	0.167	0.005	0.01	0.08	0.214	0.006	<0.01	**0.02**	0.116	0.006	0.07	0.34
**Corticospinal Tract**																
TNF-α	-0.005	0.002	0.93	0.94	-0.034	0.003	0.53	0.68	-0.072	0.003	0.22	0.28	-0.024	0.004	0.67	0.92
SES group	0.045	0.003	0.36	0.65	0.109	0.004	0.01	**0.01**	0.225	0.004	<0.001	**<0.001**	0.062	0.005	0.16	0.18
GA at delivery	0.031	0.001	0.56	0.72	0.022	0.002	0.63	0.71	0.051	0.001	0.30	0.68	0.007	0.002	0.87	0.98
PMA at scan	0.537	0.001	<0.001	**<0.001**	-0.706	0.002	<0.001	**<0.001**	-0.636	0.001	<0.001	**<0.001**	-0.681	0.002	<0.001	**<0.001**
Sex	-0.013	0.003	0.78	0.99	-0.048	0.004	0.25	0.46	-0.087	0.003	0.06	0.08	-0.033	0.005	0.45	0.73
TNF-α:SES group	-0.057	0.004	0.36	0.65	0.065	0.005	0.24	0.30	0.048	0.004	0.41	0.48	0.068	0.006	0.23	0.35
**Optic Radiation**																
TNF-α	-0.021	0.002	0.74	0.94	0.001	0.004	0.98	0.98	0.002	0.004	0.98	0.98	0.008	0.004	0.89	0.92
SES group	0.082	0.003	0.11	0.24	0.175	0.005	<0.01	**<0.01**	0.304	0.005	<0.001	**<0.001**	0.112	0.006	0.02	**0.03**
GA at delivery	0.008	0.001	0.89	0.89	-0.110	0.002	0.03	0.23	-0.130	0.002	0.02	0.10	-0.090	0.002	0.07	0.16
PMA at scan	0.530	0.001	<0.001	**<0.001**	-0.570	0.002	<0.001	**<0.001**	-0.315	0.002	<0.001	**<0.001**	-0.603	0.002	<0.001	**<0.001**

Sex	0.001	0.003	0.99	0.99	-0.130	0.005	<0.01	**0.03**	-0.196	0.005	<0.001	**<0.01**	-0.097	0.006	0.03	0.14
TNF-α:SES group	-0.017	0.004	0.79	0.86	0.017	0.006	0.77	0.77	-0.002	0.006	0.97	0.97	0.018	0.007	0.76	0.76
**Uncinate Fasciculus**																
TNF-α	-0.133	0.002	0.03	0.15	-0.026	0.003	0.68	0.77	-0.128	0.004	0.07	0.16	0.022	0.003	0.71	0.92
SES group	-0.025	0.003	0.61	0.91	0.147	0.004	<0.01	**0.01**	0.144	0.005	0.01	**0.01**	0.125	0.004	0.01	**0.02**
GA at delivery	0.116	0.001	0.03	0.07	0.056	0.002	0.30	0.54	0.150	0.002	0.01	0.10	0.001	0.002	0.99	0.99
PMA at scan	0.493	0.001	<0.001	**<0.001**	-0.564	0.002	<0.001	**<0.001**	-0.284	0.002	<0.001	**<0.001**	-0.613	0.002	<0.001	**<0.001**
Sex	-0.084	0.002	0.09	0.20	-0.066	0.004	0.18	0.41	-0.136	0.005	0.01	**0.03**	-0.027	0.004	0.57	0.73
TNF-α:SES group	0.020	0.003	0.75	0.86	-0.064	0.005	0.31	0.35	-0.056	0.006	0.43	0.48	-0.058	0.005	0.34	0.38
**Inferior Fronto-Occipital Fasciculus**																
TNF-α	0.050	0.002	0.38	0.85	-0.083	0.004	0.14	0.24	-0.065	0.003	0.32	0.36	-0.078	0.004	0.15	0.36
SES group	0.000	0.003	1.00	1.00	0.134	0.005	<0.01	**0.01**	0.245	0.004	<0.001	**<0.001**	0.097	0.006	0.03	**0.04**
GA at delivery	0.137	0.001	0.01	0.05	-0.084	0.002	0.08	0.23	0.017	0.001	0.77	0.86	-0.105	0.002	0.03	0.13
PMA at scan	0.588	0.001	<0.001	**<0.001**	-0.636	0.002	<0.001	**<0.001**	-0.457	0.001	<0.001	**<0.001**	-0.640	0.002	<0.001	**<0.001**
Sex	0.004	0.003	0.93	0.99	-0.112	0.005	0.01	**0.03**	-0.181	0.004	<0.001	**<0.01**	-0.079	0.006	0.06	0.19
TNF-α:SES group	-0.069	0.004	0.23	0.64	0.084	0.006	0.13	0.24	0.070	0.004	0.28	0.48	0.083	0.007	0.13	0.35
**Anterior Limb of Internal Capsule**																
TNF-α	0.059	0.002	0.31	0.85	-0.146	0.004	0.01	0.06	-0.166	0.004	0.01	0.04	-0.132	0.005	0.02	0.19
SES group	0.011	0.003	0.81	0.91	0.125	0.006	0.01	**0.01**	0.181	0.005	<0.01	**<0.01**	0.099	0.007	0.03	**0.04**
GA at delivery	0.118	0.001	0.02	0.07	-0.093	0.002	0.07	0.23	-0.027	0.002	0.64	0.82	-0.106	0.002	0.03	0.13
PMA at scan	0.590	0.001	<0.001	**<0.001**	-0.569	0.002	<0.001	**<0.001**	-0.430	0.002	<0.001	**<0.001**	-0.592	0.002	<0.001	**<0.001**
Sex	-0.026	0.003	0.56	0.99	-0.042	0.006	0.37	0.47	-0.064	0.005	0.22	0.22	-0.029	0.006	0.52	0.73
TNF-α:SES group	-0.061	0.003	0.29	0.64	0.111	0.007	0.06	0.14	0.120	0.006	0.07	0.17	0.103	0.008	0.08	0.34
**Inferior Cingulum Bundle**																
TNF-α	-0.171	0.002	0.01	0.12	-0.075	0.002	0.20	0.31	-0.196	0.003	<0.01	**0.04**	0.006	0.002	0.92	0.92
SES group	-0.019	0.002	0.73	0.91	0.199	0.003	<0.001	**<0.001**	0.190	0.003	<0.01	**<0.01**	0.154	0.003	<0.01	**0.01**
GA at delivery	-0.114	0.001	0.05	0.10	0.062	0.001	0.22	0.50	-0.040	0.001	0.50	0.77	0.103	0.001	0.04	0.13
PMA at scan	0.427	0.001	<0.001	**<0.001**	-0.609	0.001	<0.001	**<0.001**	-0.319	0.001	<0.001	**<0.001**	-0.639	0.001	<0.001	**<0.001**
Sex	-0.016	0.002	0.76	0.99	-0.124	0.003	0.01	**0.03**	-0.125	0.003	0.02	**0.04**	-0.103	0.003	0.03	0.14
TNF-α:SES group	0.081	0.003	0.24	0.64	0.133	0.003	0.03	0.12	0.197	0.004	0.00	**0.02**	0.062	0.004	0.30	0.38
**Fornix**																
TNF-α	-0.005	0.001	0.94	0.94	-0.148	0.002	0.01	0.06	-0.169	0.003	0.01	**0.04**	-0.118	0.002	0.04	0.19
SES group	-0.107	0.002	0.06	0.17	0.116	0.003	0.01	**0.01**	0.073	0.004	0.16	0.18	0.130	0.003	<0.01	**0.01**
GA at delivery	0.021	0.001	0.73	0.82	-0.018	0.001	0.72	0.72	0.000	0.001	1.00	1.00	-0.024	0.001	0.63	0.94
PMA at scan	0.352	0.001	<0.001	**<0.001**	-0.624	0.001	<0.001	**<0.001**	-0.482	0.001	<0.001	**<0.001**	-0.634	0.001	<0.001	**<0.001**
Sex	-0.102	0.002	0.06	0.20	-0.030	0.003	0.51	0.57	-0.084	0.004	0.10	0.11	0.007	0.003	0.87	0.97
TNF-α:SES group	0.012	0.002	0.86	0.86	0.110	0.004	0.06	0.14	0.154	0.005	0.02	0.06	0.077	0.004	0.19	0.35

β, standardized beta coefficient; SE, standard error; *q*, FDR-corrected p-value; IL, interleukin; TNF-α, tumor necrosis factor alpha; FA, fractional anisotropy; MD, mean diffusivity; AD, axial diffusivity; RD, radial diffusivity; GA, gestational age; PMA, infant postmenstrual age; SES, socioeconomic status.

Figures

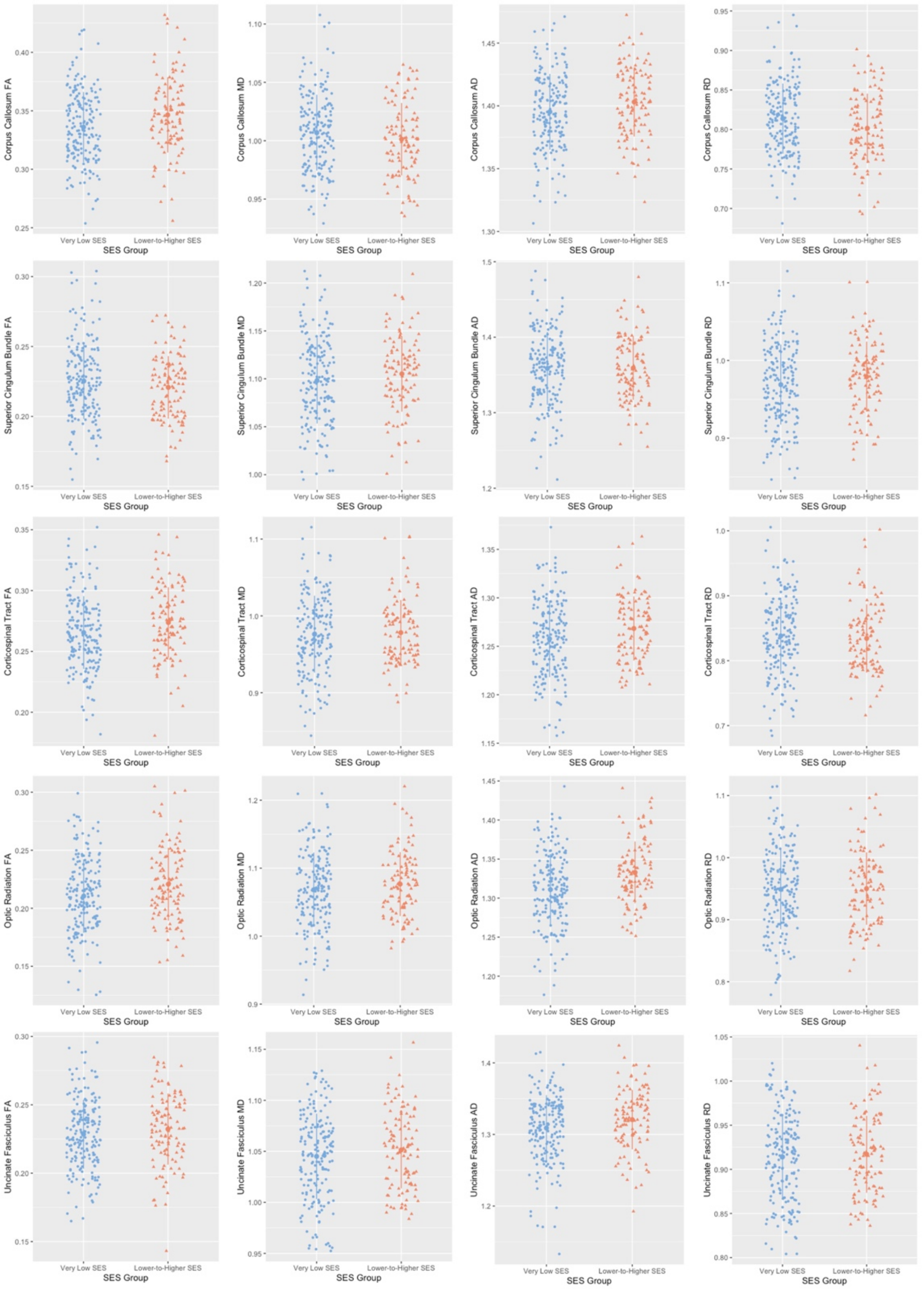
Corpus Callosum FA
Corpus Callosum MD
Corpus Callosum AD
Corpus Callosum RD
Superior Cingulum Bundle FA
Superior Cingulum Bundle MD
Superior Cingulum Bundle AD
Superior Cingulum Bundle RD
Corticospinal Tract FA
Corticospinal Tract MD
Corticospinal Tract AD
Corticospinal Tract RD
Optic Radiation FA
Optic Radiation MD
Optic Radiation AD
Optic Radiation RD
Uncinate Fasciculus FA
Uncinate Fasciculus MD
Uncinate Fasciculus AD
Uncinate Fasciculus RD
Very Low SES
Lower-to-Higher SES
SES Group

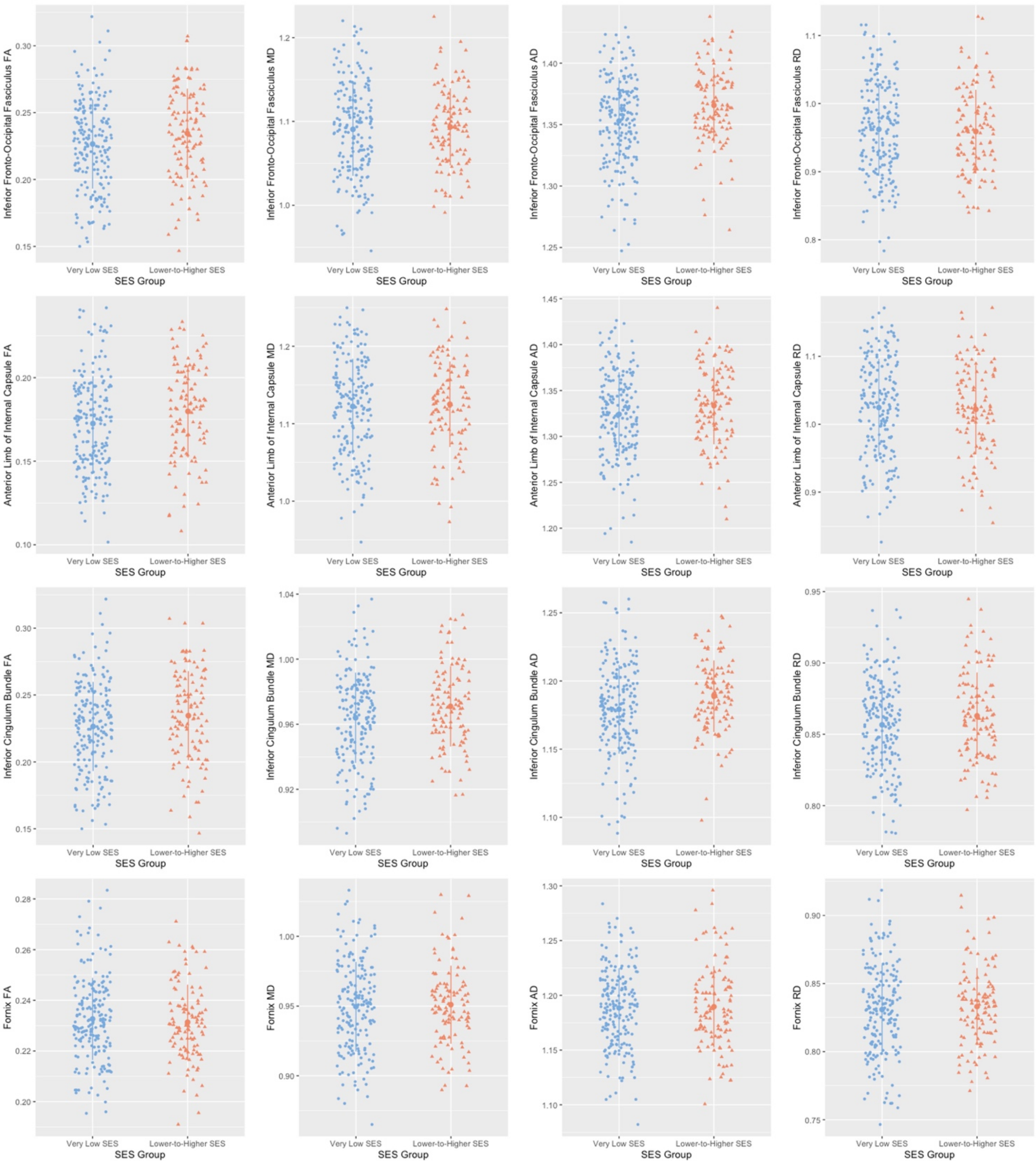
Inferior Fronto-Occipital Fasciculus FA
Inferior Fronto-Occipital Fasciculus MD
Inferior Fronto-Occipital Fasciculus AD
Inferior Fronto-Occipital Fasciculus RD
Anterior Limb of Internal Capsule FA
Anterior Limb of Internal Capsule MD
Anterior Limb of Internal Capsule AD
Anterior Limb of Internal Capsule RD
Inferior Cingulum Bundle FA
Inferior Cingulum Bundle MD
Inferior Cingulum Bundle AD
Inferior Cingulum Bundle RD
Fornix FA
Fornix MD
Fornix AD
Fornix RD
Very Low SES
Lower-to-Higher SES
SES Group

Figure S1. Dimensional scatterplots of the relationships between family SES group and neonatal white matter tract dMRI parameters. FA, fractional anisotropy; MD, mean diffusivity; AD, axial diffusivity; RD, radial diffusivity.

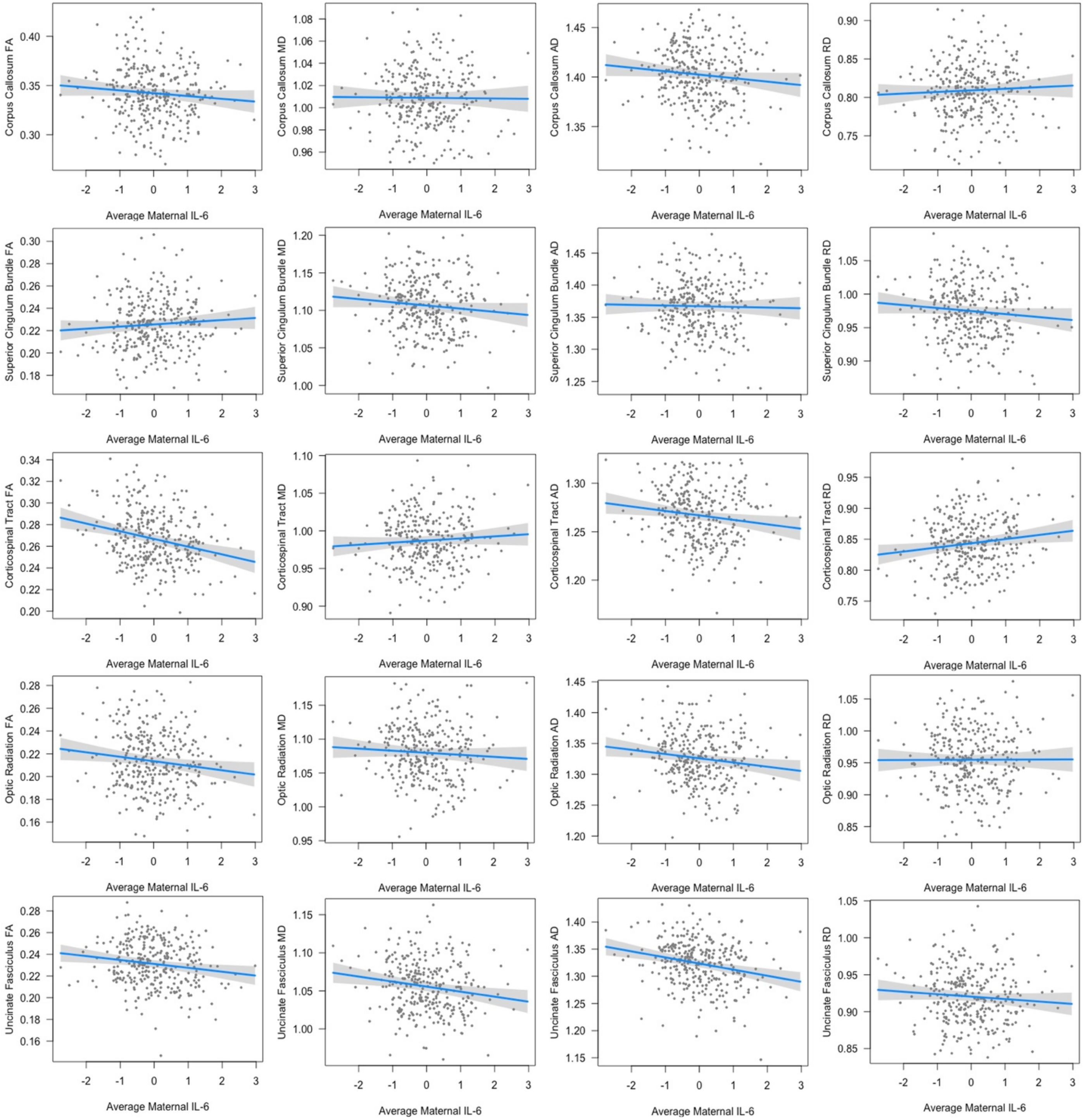
Corpus Callosum FA
Corpus Callosum MD
Corpus Callosum AD
Corpus Callosum RD
Superior Cingulum Bundle FA
Superior Cingulum Bundle MD
Superior Cingulum Bundle AD
Superior Cingulum Bundle RD
Corticospinal Tract FA
Corticospinal Tract MD
Corticospinal Tract AD
Corticospinal Tract RD
Optic Radiation FA
Optic Radiation MD
Optic Radiation AD
Optic Radiation RD
Uncinate Fasciculus FA
Uncinate Fasciculus MD
Uncinate Fasciculus AD
Uncinate Fasciculus RD
Average Maternal IL-6

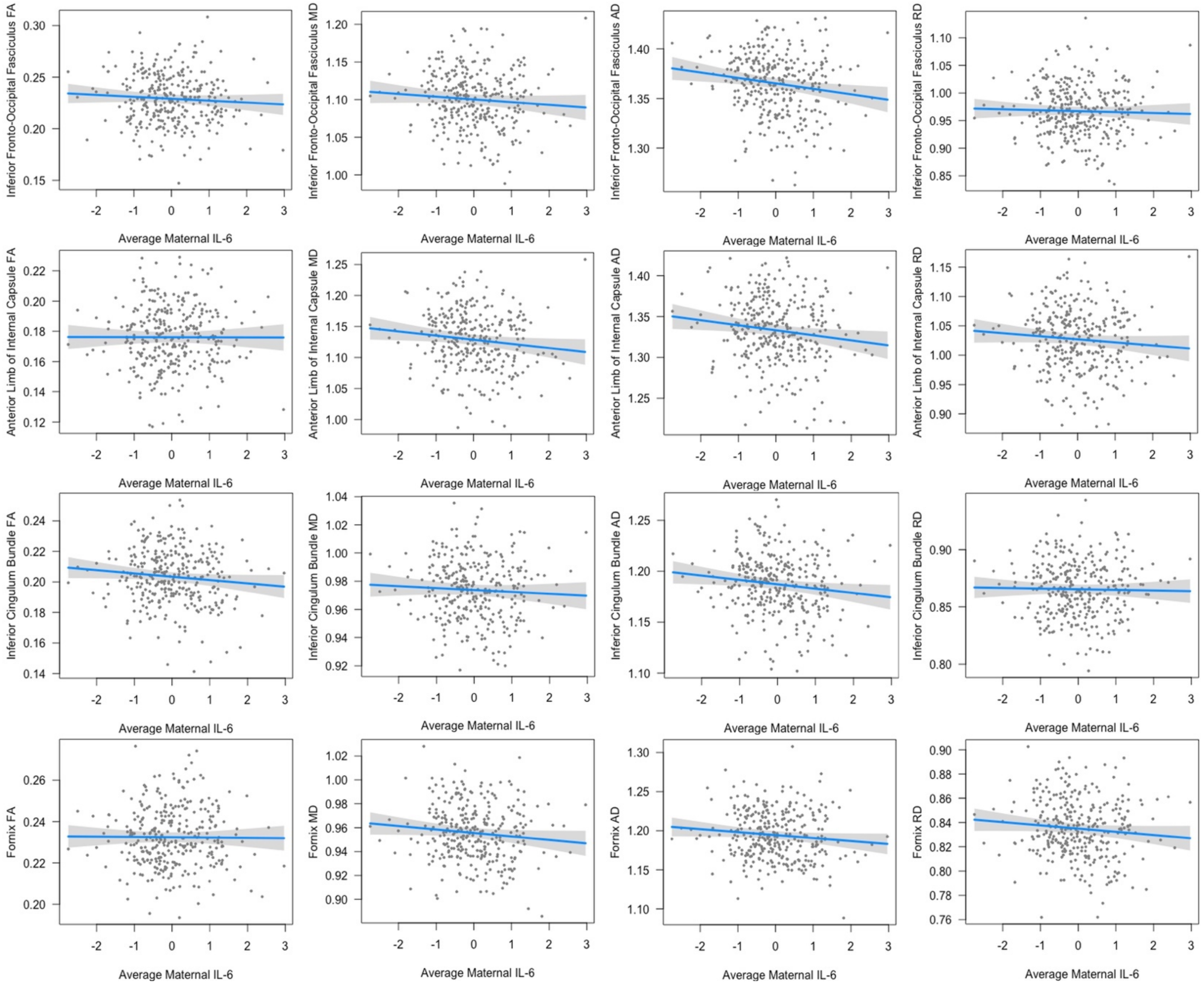

Figure S2. Multiple linear regression plots of the relationship between average maternal IL-6 levels during pregnancy and neonatal white matter tract dMRI parameters. Covariates in models are child sex, gestational age at delivery, infant postmenstrual age at scan, and NICU stay. FA, fractional anisotropy; MD, mean diffusivity; AD, axial diffusivity; RD, radial diffusivity; IL, interleukin.

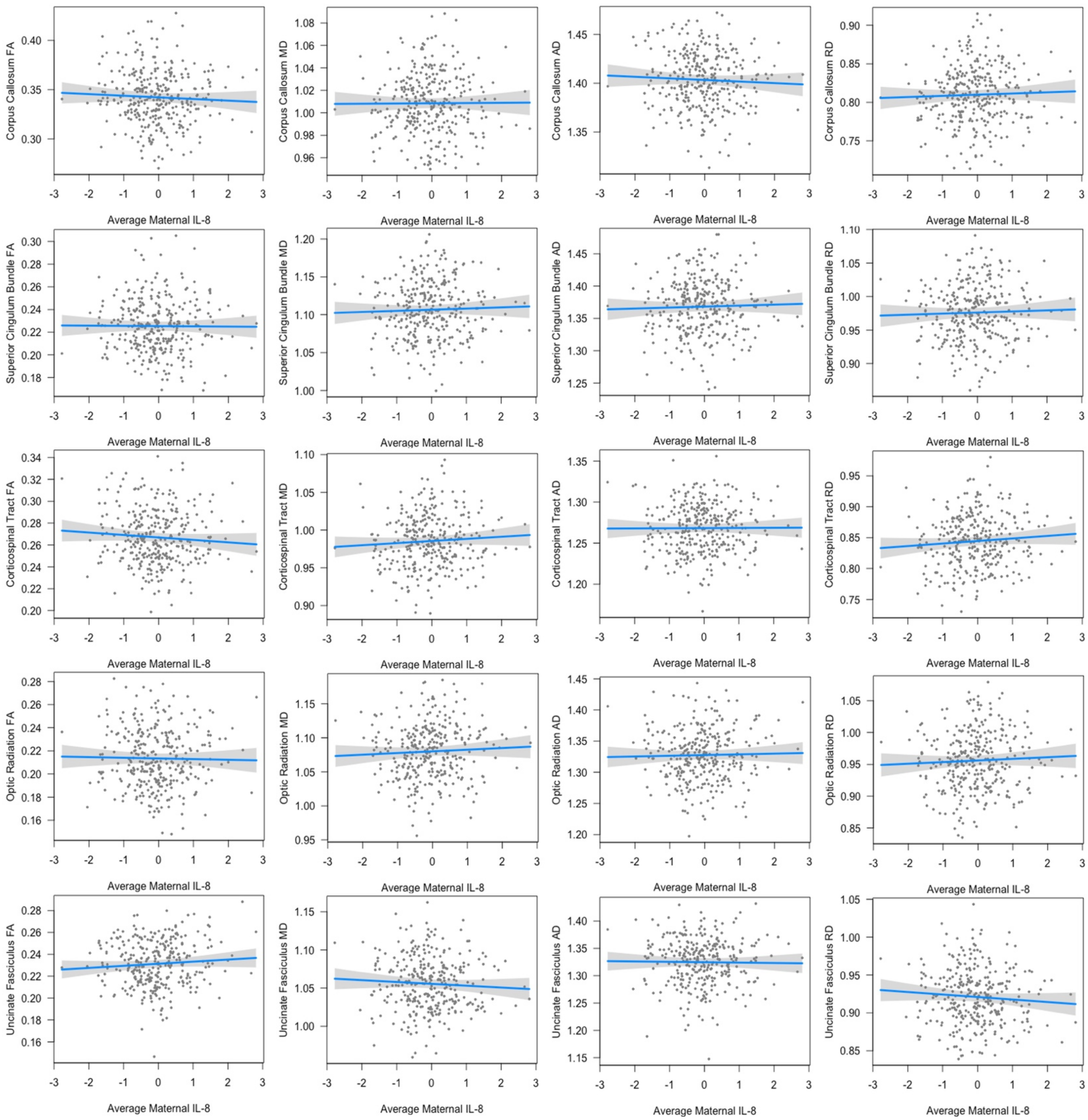

Corpus Callosum FA
Corpus Callosum MD
Corpus Callosum AD
Corpus Callosum RD
Superior Cingulum Bundle FA
Superior Cingulum Bundle MD
Superior Cingulum Bundle AD
Superior Cingulum Bundle RD
Corticospinal Tract FA
Corticospinal Tract MD
Corticospinal Tract AD
Corticospinal Tract RD
Optic Radiation FA
Optic Radiation MD
Optic Radiation AD
Optic Radiation RD
Uncinate Fasciculus FA
Uncinate Fasciculus MD
Uncinate Fasciculus AD
Uncinate Fasciculus RD
Average Maternal IL-8

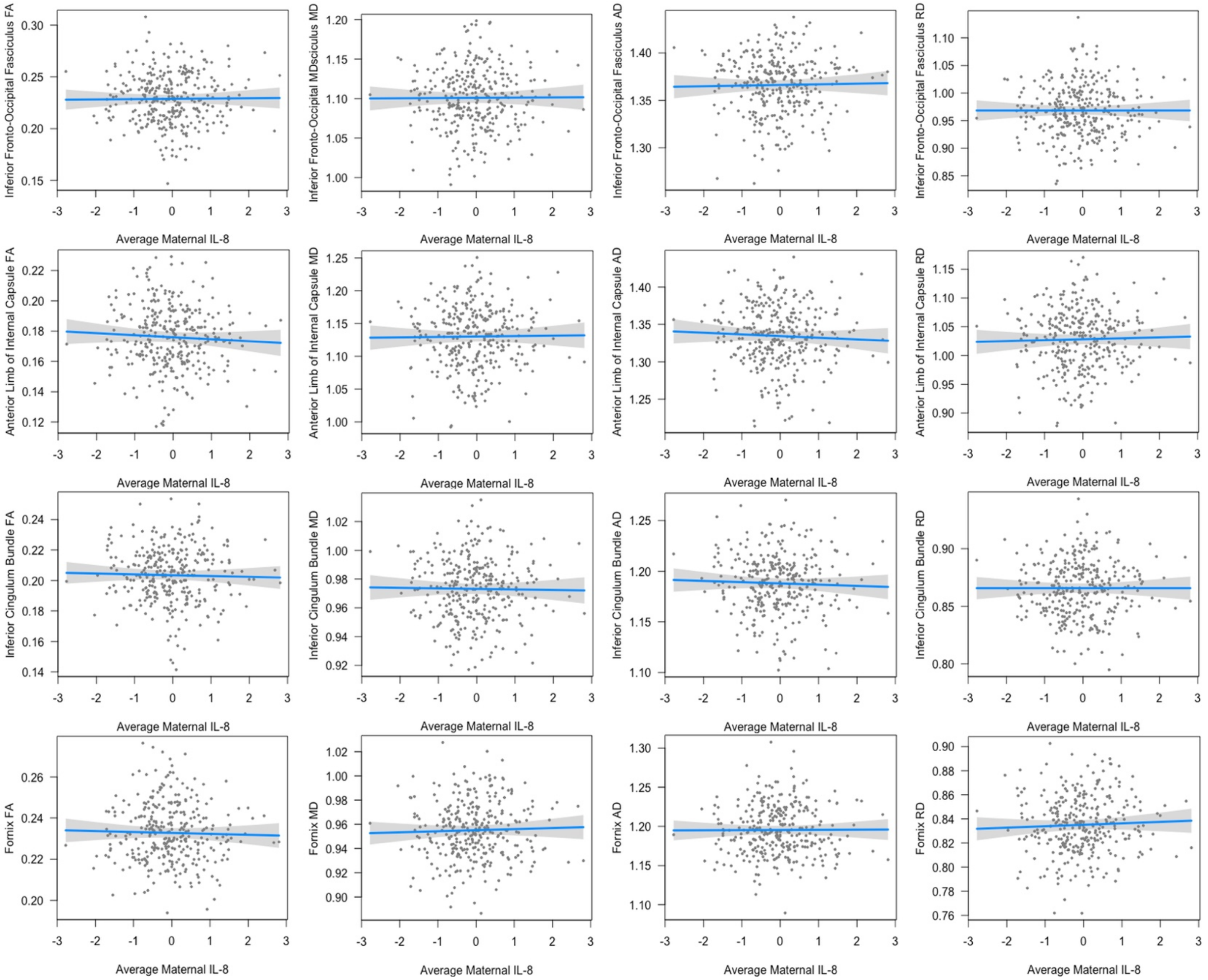

Figure S3. Multiple linear regression plots of the relationship between average maternal IL-8 levels during pregnancy and neonatal white matter tract dMRI parameters. Covariates in models are child sex, gestational age at delivery, infant postmenstrual age at scan, and NICU stay. FA, fractional anisotropy; MD, mean diffusivity; AD, axial diffusivity; RD, radial diffusivity; IL, interleukin.

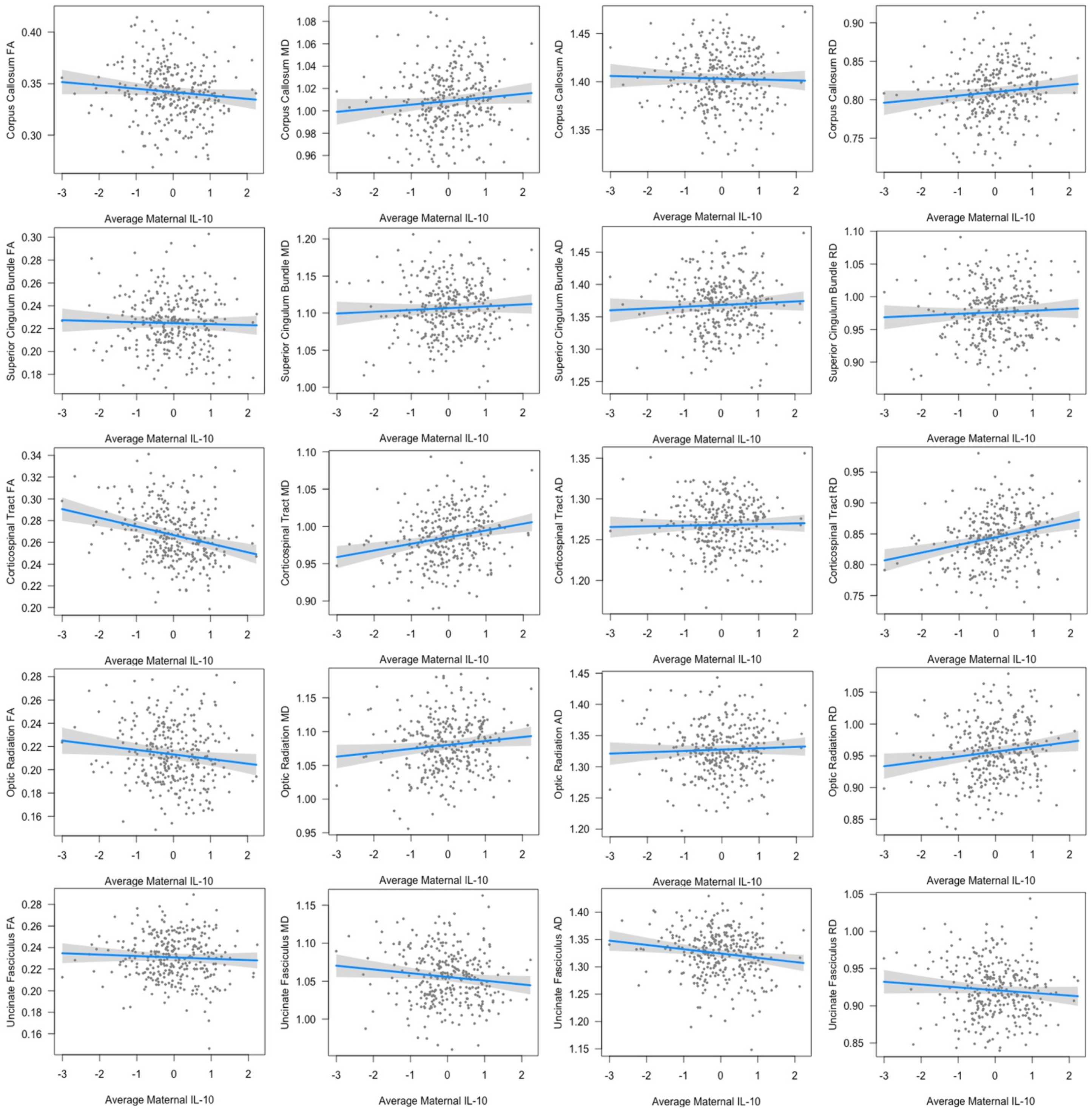
Corpus Callosum FA
Corpus Callosum MD
Corpus Callosum AD
Corpus Callosum RD
Superior Cingulum Bundle FA
Superior Cingulum Bundle MD
Superior Cingulum Bundle AD
Superior Cingulum Bundle RD
Corticospinal Tract FA
Corticospinal Tract MD
Corticospinal Tract AD
Corticospinal Tract RD
Optic Radiation FA
Optic Radiation MD
Optic Radiation AD
Optic Radiation RD
Uncinate Fasciculus FA
Uncinate Fasciculus MD
Uncinate Fasciculus AD
Uncinate Fasciculus RD
Average Maternal IL-10

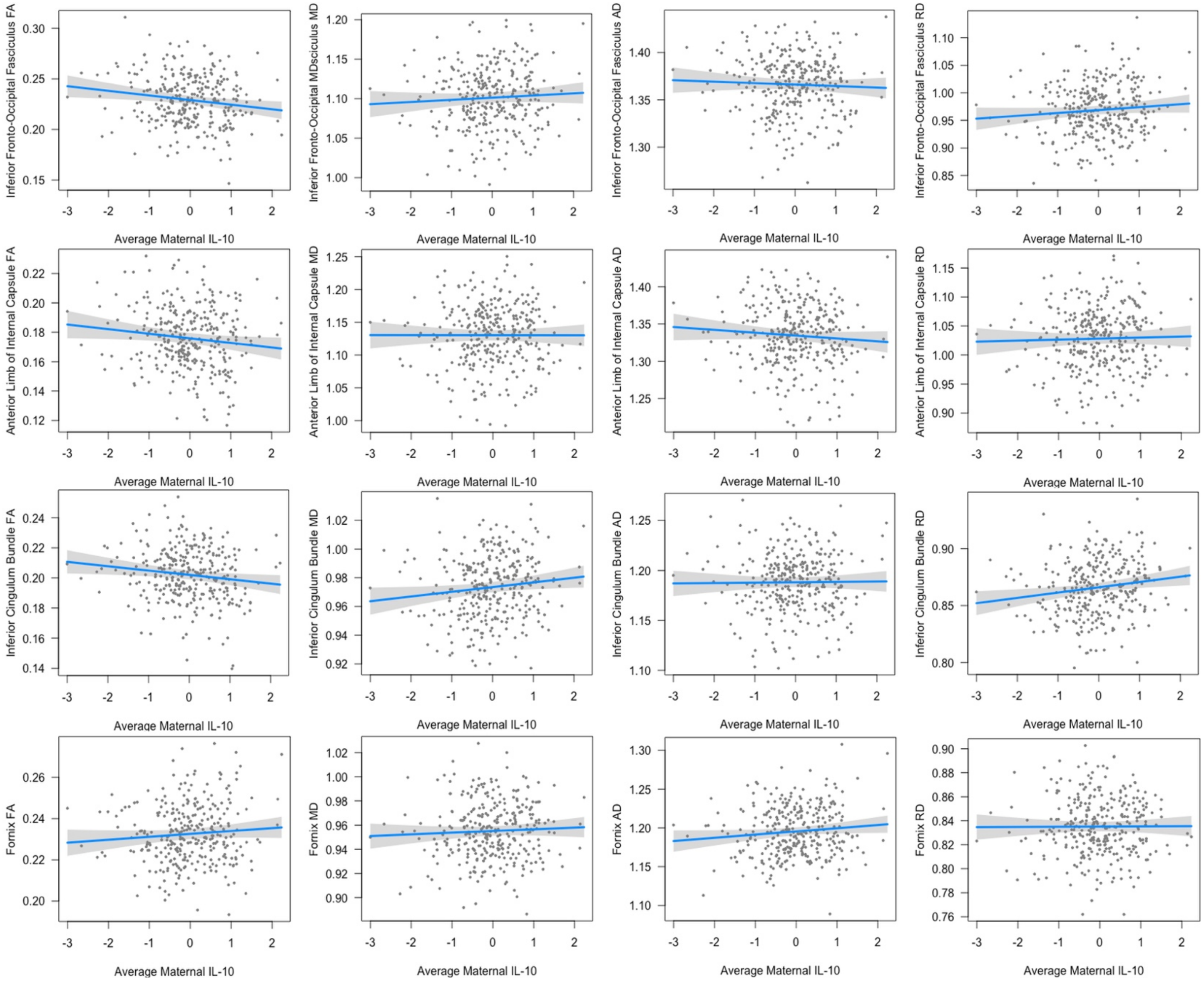

Figure S4. Multiple linear regression plots of the relationship between average maternal IL-10 levels during pregnancy and neonatal white matter tract dMRI parameters. Covariates in models are child sex, gestational age at delivery, infant postmenstrual age at scan, and NICU stay. FA, fractional anisotropy; MD, mean diffusivity; AD, axial diffusivity; RD, radial diffusivity; IL, interleukin.

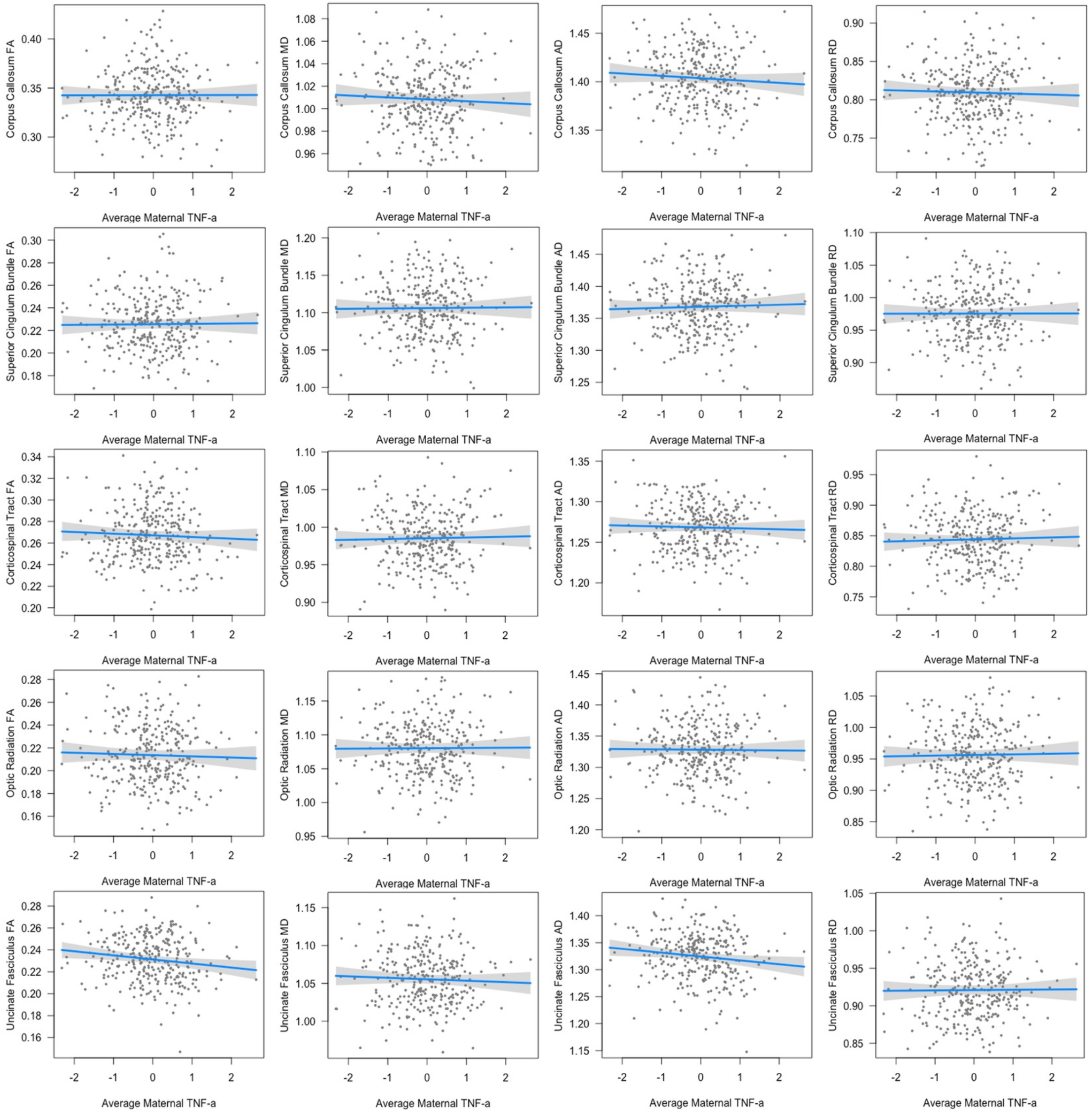
Corpus Callosum FA
Corpus Callosum MD
Corpus Callosum AD
Corpus Callosum RD
Superior Cingulum Bundle FA
Superior Cingulum Bundle MD
Superior Cingulum Bundle AD
Superior Cingulum Bundle RD
Corticospinal Tract FA
Corticospinal Tract MD
Corticospinal Tract AD
Corticospinal Tract RD
Optic Radiation FA
Optic Radiation MD
Optic Radiation AD
Optic Radiation RD
Uncinate Fasciculus FA
Uncinate Fasciculus MD
Uncinate Fasciculus AD
Uncinate Fasciculus RD
Average Maternal TNF-a

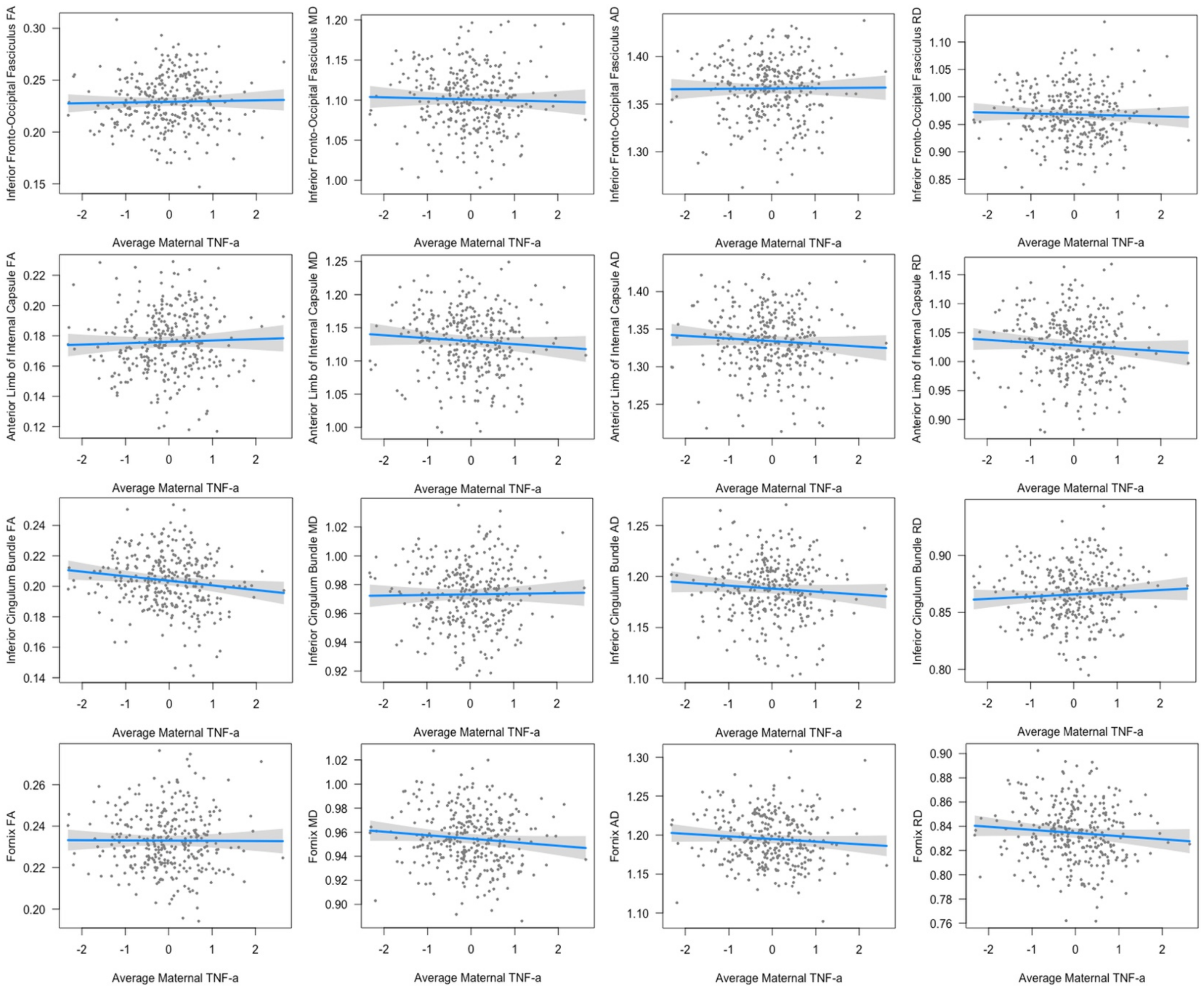

Figure S5. Multiple linear regression plots of the relationship between average maternal tumor necrosis factor alpha levels during pregnancy and neonatal white matter tract dMRI parameters. Covariates in models are child sex, gestational age at delivery, infant postmenstrual age at scan, and NICU stay. FA, fractional anisotropy; MD, mean diffusivity; AD, axial diffusivity; RD, radial diffusivity; TNF-a, tumor necrosis factor alpha.

Supplementary Methods

Covariates for the SES group and maternal cytokine analysis included maternal age and maternal pre-pregnancy body mass index (BMI) to account for their potentially confounding roles on cytokine levels during pregnancy. Aging[1] and increased BMI[2] are linked to heightened concentrations of circulating cytokines and proinflammatory markers. For SES group, neonatal dMRI, and maternal cytokine analyses, covariates included sex, NICU stay >7 days, gestational age (GA) at delivery, and infant postmenstrual age (PMA) at scan [3, 4]. While all infants in this sample were scanned within the first month of life, there was variability in PMA that may impact measures of white matter microstructure. Prior analyses from the same cohort used in the current study showed that infant PMA at scan accounted for 13-31% of the variance in MD, 18-32% of the variance in RD, 4-17% of the variance in AD, and 8-26% of the variance in FA across tracks and was therefore included as a covariate to account for expected age-related differences in infant white matter connectivity metrics.[5] Additionally, GA ranged from 28-41 weeks and was therefore included as a covariate. Our sample also included infants who were admitted to the NICU (>7 days). Given that NICU stressors have been associated with regional alterations in brain structure and function,[6] we controlled for NICU stay (no NICU stay = 0, NICU stay = 1) in all analyses. Finally, prior studies have reported sex differences in infant dMRI measures.[7] Therefore, models were adjusted for infant sex assigned at birth (male = 1, female = 2).

Psychosocial stress.

In each trimester, mothers completed the Edinburg Postnatal Depression Scale (EDPS)[8] and the Cohen Perceived Stress Scale (PSS)[9] to assess depression symptoms and perceived stress, respectively. At the neonatal MRI scan, mothers completed the Stress and Adversity Inventory for Adults (STRAIN)[10] and the Everyday Discrimination Survey[11] to assess stressful/traumatic life events (count and severity) and racial discrimination, respectively. These observed variables were analyzed using structural equation modeling, resulting in the latent factor "psychosocial stress". Observed psychosocial stress variables were all correlated (range 0.21-0.93, all $p < 0.01$). See Luby et al.[12] for details. Mothers in the very low SES group had higher Psychosocial Stress scores (m=0.18, SD=0.96) than mothers in the lower-to-higher SES group (m=-0.39, SD=0.76, p<0.01). There was a negative relationship between neonatal CBIF and maternal Psychosocial Stress (β=-0.13; q=0.02) controlling for child sex, NICU stay, GA at delivery, and infant PMA. There were no other associations between infant dMRI metrics and maternal Psychosocial Stress. Additionally, there were no associations between maternal Psychosocial stress scores and cytokine levels during pregnancy controlling for maternal age and pre-pregnancy BMI.

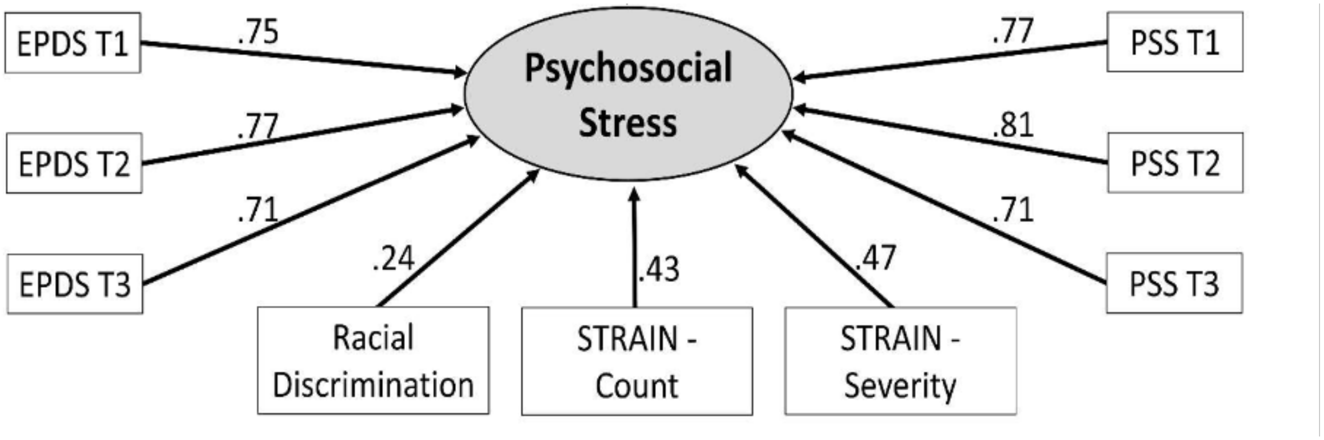

Figure S6. Structural equation model of psychosocial stress latent factor. Adapted from Luby et al.[12], Figure S1 illustrates the latent prenatal Psychosocial Stress factor and its observed components (n=399). Standard estimates between the latent and observed variables are shown. T1, trimester 1; T2, trimester 2; T3, trimester 3; EPDS, Edinburg Postnatal Depression Scale; STRAIN, Stress and Adversity Inventory for Adults; PSS, Cohen Perceived Stress Scale.

Supplementary Discussion

Early research into neonatal diffusion-based MR imaging revealed that water diffusion in the brain decreases while anisotropy rises with increasing age [13-15]. That is, as the brain matures, its water content declines, resulting in a drop in MD. Conversely, anisotropy values tend to increase with brain maturation due to increases in myelin content, resulting in age-dependent increases in FA [16]. However, FA values are more susceptible to noise than MD values – they are considerably affected by the number of diffusion-weighting directions [17-19], and this effect is greater for isotropic than anisotropic structures [20]. Consequently, FA values tend to be overestimated due to such noise [21].

Another caveat related to FA in neonates is its dependence on myelin content. In utero, the human fetal brain's major white matter tracts primarily develop over the second and third trimesters. During the first trimester, some commissural (e.g., corpus callosum) and projection (e.g., corticospinal tract) fibers are present, followed by limbic (e.g., cingulum) and association (fronto-occipital fasciculus) tracts that develop into the second and third trimesters [4, 22, 23]. While there is little myelin present during gestation, growth of myelin sheath around neuronal axons begins around mid-to-late gestation and is critical for short- and long-reaching communication in the brain [24]. Limbic and association fibers continue to myelinate into the first two postnatal years [25]. This developmental timing is important when considering FA in neonates – not all white matter tracts studied here are myelinated by the sampled age (i.e., >30 days). Therefore, we primarily focus on tract diffusivity in our discussion of findings while reminding the reader that our interpretation of FA-related results should be understood in the context of the caveats outlined here.

We found that family SES was related to white matter tract diffusivity (MD), but not anisotropy (FA), at birth. Neonates in the very low SES group displayed significantly lower MD in all tracts investigated except for the corpus callosum. The differential timing of white matter tract development in the human brain is characterized by two categories: "early developing" and "late developing" [26]. Early developing tracts include projections connecting sensorimotor regions, whereas later developing tracts project to and from association systems. In the current study, we found evidence of lower MD and AD in both early and later developing tracks, and lower RD primarily in later developing tracts in neonates born to very low SES families. Reflecting findings from Lean et al. [5], our results suggest that SES may alter white matter microstructure regardless of myelination timing as we found relations to both early and late developing tracts. Reductions in MD neonatally reflect brain maturation. Here, we found evidence for aberrant maturation of brain-wide white matter tracts in neonates born to families experiencing economic hardship. A recent Perspective by Tooley et al. [27] outlined how environmental stressors, such as those commonly experienced by individuals from low SES backgrounds, may impact the pace of brain development. Children living in poverty face higher instances of crime [28], toxin exposure (e.g., lead, mercury) [29], noise and air pollution [30], sleep disturbances [31], and nutrient deficiencies [32]. Exposure to such stressors *in utero* has the potential to disrupt synaptic plasticity and myelination, ultimately affecting the course of neurodevelopment in profound ways. Specifically, they posit exposure to chronic, negative events accelerates brain maturation and reduces plasticity [33]. Our findings point to altered maturation of white matter in association with low SES, such that axonal integrity (as indicated by AD) may be altered across tracts regardless of their maturational timing and myelination (as indicated by RD) may be impacted in tracts that continue to develop postnatally.

Maternal inflammatory biomarkers during pregnancy were also associated with SES and altered white matter microstructure. IL-6 was the only cytokine studied here associated with family SES. Mothers in the very low SES group had significantly higher average IL-6 concentrations than those in the lower-to-higher SES group. Additionally, pregnant mothers with higher average IL-6 concentration birthed offspring with significantly lower AD in both early and later developing white matter tracts, and lower FA in both the corticospinal tract and uncinate. IL-6, a cytokine characterized

by its pro-inflammatory function, plays a necessary role in fetal brain development [34]. However, chronically elevated levels may have deleterious effects on cell survival, synaptogenesis, and axonal growth [35]. In mice, a single maternal injection of IL-6 can yield offspring with significant behavioral abnormalities [36, 37], illustrating the fetal brain's malleability in response to its environment. Our finding of lower neonatal AD across most tracts in relation to elevated maternal IL-6 concentration supports the notion that heightened inflammatory cytokine exposure may alter the course of axonal maturation. In terms of tract FA, a study by Rasmussen et al. [38] similarly found that elevated maternal IL-6 was associated with lower uncinate FA in newborns. The uncinate plays a key role in communication between regions involved in emotion regulation and higher-order cognition, and its integrity is associated with socioemotional development. Though our results suggest a negative relationship between maternal IL-6 and neonatal uncinate FA, it is important to note that FA is relatively insensitive to isotropic processes. Since the uncinate is one of the last structures to fully myelinate, this is of particular concern [39, 40]. While we refrain from maturational rate inferences due to such concerns, our results in conjunction with those of Rasmussen et al. suggest that the uncinate in a developing fetus may be one tract that is sensitive to maternal inflammation during pregnancy.

In addition, we found that higher average maternal IL-10 concentration was associated with offspring exhibiting higher MD and RD in the corticospinal tract, higher RD in the inferior cingulum bundle, and lower FA in the corticospinal tract, inferior fronto-occipital fasciculus, and inferior cingulum bundle. IL-10, a key anti-inflammatory cytokine, has the ability to suppress inflammatory cytokine production. However, chronically elevated IL-10 concentrations have been implicated in behavioral abnormalities [41] and demyelination of white matter [42] in animal models. In patients with schizophrenia, higher circulating IL-10 levels have been linked with widespread increases in white matter MD and RD and decreases in FA [43]. While IL-10's role as a protective regulator has been highlighted in the literature, here we provide evidence for its role in neonatal white matter alterations. That is, elevated maternal IL-10 concentrations during gestation may have implications for maturational disturbances of major projection, limbic, and association fibers.

To interpret the pattern of results for SES and cytokine relations to white matter measures, we must also consider normal maturation and neurodevelopment. Myelination starts *in utero* and, for some prefrontal fiber tracts, is not complete until adolescence. As the brain matures, its water content declines, resulting in a drop in MD. Conversely, anisotropy values tend to increase with brain maturation due to increasing myelin content, resulting in age-dependent increases in FA [16]. At the PMA studied herein, some tracts are myelinated and others are not, which affects the interpretation of FA values. While MD reflects water displacements in all directions within a voxel, FA represents the spatial heterogeneity of those displacements. FA values in myelinated white matter thereby reflect the degree of myelination, with heavier or more mature myelination leading to higher FA values. For unmyelinated white matter tracts, FA is related to the packing of parallel fibers, with tighter packing leading to higher FA values. At the age at which infants were evaluated for this study (term equivalent PMA), the CST and, to a lesser extent, the OR are myelinated (i.e., earlier developing), and the remainder of the white matter tracts included in this study are not (i.e., later developing). Overall, higher RD values with lower FA suggest altered maturation and, in the case of the earlier developing tracts, reduced myelination [44-47].

References

1. Michaud M, Balardy L, Moulis G, Gaudin C, Peyrot C, Vellas B *et al.* Proinflammatory cytokines, aging, and age-related diseases. *J Am Med Dir Assoc* 2013; **14**(12)**:** 877-882.

2. Maguire RL, House JS, Lloyd DT, Skinner HG, Allen TK, Raffi AM *et al.* Associations between maternal obesity, gestational cytokine levels and child obesity in the NEST cohort. *Pediatr Obes* 2021; **16**(7)**:** e12763.

3. Back SA, Luo NL, Borenstein NS, Levine JM, Volpe JJ, Kinney HC. Late oligodendrocyte progenitors coincide with the developmental window of vulnerability for human perinatal white matter injury. *J Neurosci* 2001; **21**(4)**:** 1302-1312.

4. Dubois J, Dehaene-Lambertz G, Kulikova S, Poupon C, Hüppi PS, Hertz-Pannier L. The early development of brain white matter: a review of imaging studies in fetuses, newborns and infants. *Neuroscience* 2014; **276:** 48-71.

5. Lean RE, Smyser CD, Brady RG, Triplett RL, Kaplan S, Kenley JK *et al.* Exposure to Prenatal Social Disadvantage and Maternal Psychosocial Stress: Relationships to Neonatal White Matter Connectivity. *medRxiv* 2022.

6. Smith GC, Gutovich J, Smyser C, Pineda R, Newnham C, Tjoeng TH *et al.* Neonatal intensive care unit stress is associated with brain development in preterm infants. *Ann Neurol* 2011; **70**(4)**:** 541-549.

7. Dean DC, Planalp EM, Wooten W, Adluru N, Kecskemeti SR, Frye C *et al.* Mapping White Matter Microstructure in the One Month Human Brain. *Sci Rep* 2017; **7**(1)**:** 9759.

8. Cox JL, Holden JM, Sagovsky R. Detection of postnatal depression. Development of the 10-item Edinburgh Postnatal Depression Scale. *Br J Psychiatry* 1987; **150:** 782-786.

9. Cohen S, Kamarck T, Mermelstein R. A global measure of perceived stress. *J Health Soc Behav* 1983; **24**(4)**:** 385-396.

10. Slavich GM, Shields GS. Assessing Lifetime Stress Exposure Using the Stress and Adversity Inventory for Adults (Adult STRAIN): An Overview and Initial Validation. *Psychosom Med* 2018; **80**(1)**:** 17-27.

11. Williams DR, Yan Yu, Jackson JS, Anderson NB. Racial Differences in Physical and Mental Health: Socio-economic Status, Stress and Discrimination. *J Health Psychol* 1997; **2**(3)**:** 335-351.

12. Luby JL, Barch DM, Warner B, Rogers C, Smyser C, Triplett R *et al.* Modeling prenatal adversity/advantage: Effects on birth weight. *medRxiv* 2021.

13. Hüppi PS, Maier SE, Peled S, Zientara GP, Barnes PD, Jolesz FA *et al.* Microstructural development of human newborn cerebral white matter assessed in vivo by diffusion tensor magnetic resonance imaging. *Pediatr Res* 1998; **44**(4)**:** 584-590.

14. Neil JJ, Shiran SI, McKinstry RC, Schefft GL, Snyder AZ, Almli CR *et al.* Normal brain in human newborns: apparent diffusion coefficient and diffusion anisotropy measured by using diffusion tensor MR imaging. *Radiology* 1998; **209**(1)**:** 57-66.

15. Mukherjee P, Miller JH, Shimony JS, Conturo TE, Lee BC, Almli CR *et al.* Normal brain maturation during childhood: developmental trends characterized with diffusion-tensor MR imaging. *Radiology* 2001; **221**(2)**:** 349-358.

16. Beaulieu C, Fenrich FR, Allen PS. Multicomponent water proton transverse relaxation and T2-discriminated water diffusion in myelinated and nonmyelinated nerve. *Magn Reson Imaging* 1998; **16**(10)**:** 1201-1210.

17. Bastin ME, Armitage PA, Marshall I. A theoretical study of the effect of experimental noise on the measurement of anisotropy in diffusion imaging. *Magn Reson Imaging* 1998; **16**(7)**:** 773-785.

18. Anderson AW. Theoretical analysis of the effects of noise on diffusion tensor imaging. *Magn Reson Med* 2001; **46**(6)**:** 1174-1188.

19. Skare S, Li T, Nordell B, Ingvar M. Noise considerations in the determination of diffusion tensor anisotropy. *Magn Reson Imaging* 2000; **18**(6)**:** 659-669.

20. Giannelli M, Cosottini M, Michelassi MC, Lazzarotti G, Belmonte G, Bartolozzi C *et al.* Dependence of brain DTI maps of fractional anisotropy and mean diffusivity on the number of diffusion weighting directions. *J Appl Clin Med Phys* 2009; **11**(1)**:** 2927.

21. Pierpaoli C, Basser PJ. Toward a quantitative assessment of diffusion anisotropy. *Magn Reson Med* 1996; **36**(6)**:** 893-906.

22. Ouyang M, Dubois J, Yu Q, Mukherjee P, Huang H. Delineation of early brain development from fetuses to infants with diffusion MRI and beyond. *Neuroimage* 2019; **185:** 836-850.

23. Takahashi E, Folkerth RD, Galaburda AM, Grant PE. Emerging cerebral connectivity in the human fetal brain: an MR tractography study. *Cereb Cortex* 2012; **22**(2)**:** 455-464.

24. Fields RD. Neuroscience. Change in the brain's white matter. *Science* 2010; **330**(6005)**:** 768-769.

25. Geng X, Gouttard S, Sharma A, Gu H, Styner M, Lin W *et al.* Quantitative tract-based white matter development from birth to age 2 years. *Neuroimage* 2012; **61**(3)**:** 542-557.

26. Sydnor VJ, Larsen B, Bassett DS, Alexander-Bloch A, Fair DA, Liston C *et al.* Neurodevelopment of the association cortices: Patterns, mechanisms, and implications for psychopathology. *Neuron* 2021; **109**(18)**:** 2820-2846.

27. Tooley UA, Bassett DS, Mackey AP. Environmental influences on the pace of brain development. *Nat Rev Neurosci* 2021; **22**(6)**:** 372-384.

28. Brady RG, Rogers CE, Prochaska T, Kaplan S, Lean RE, Smyser TA *et al.* The Effects of Prenatal Exposure to Neighborhood Crime on Neonatal Functional Connectivity. *Biol Psychiatry* 2022; **92**(2)**:** 139-148.

29. Rauh VA, Landrigan PJ, Claudio L. Housing and health: intersection of poverty and environmental exposures. *Ann N Y Acad Sci* 2008; **1136:** 276-288.

30. Zhang M, Mueller NT, Wang H, Hong X, Appel LJ, Wang X. Maternal exposure to ambient particulate matter≤ 2.5 µm during pregnancy and the risk for high blood pressure in childhood. *Hypertension* 2018; **72**(1)**:** 194-201.

31. Hoyniak CP, Whalen D, Luby J, Barch D, Miller JP, Zhao P *et al.* Chronodisruption during Pregnancy Mediates the Relationship between Social Disadvantage and Reduced Brain Maturation in Neonates. *medRxiv* 2022.

32. Ernawati E, Kurniawati L, Umijati S. Correlation between Gestational Weight Gain in the Second and Third Trimester and Preeclampsia Risk: A Study From Indonesia. *Indian Journal of Forensic Medicine & Toxicology* 2021; **15**(4)**:** 1072-1078.

33. Bock J, Wainstock T, Braun K, Segal M. Stress In Utero: Prenatal Programming of Brain Plasticity and Cognition. *Biol Psychiatry* 2015; **78**(5)**:** 315-326.

34. Burns TM, Clough JA, Klein RM, Wood GW, Berman NE. Developmental regulation of cytokine expression in the mouse brain. *Growth Factors* 1993; **9**(4)**:** 253-258.

35. Deverman BE, Patterson PH. Cytokines and CNS development. *Neuron* 2009; **64**(1)**:** 61-78.

36. Smith SE, Li J, Garbett K, Mirnics K, Patterson PH. Maternal immune activation alters fetal brain development through interleukin-6. *J Neurosci* 2007; **27**(40)**:** 10695-10702.

37. Samuelsson A-M, Jennische E, Hansson H-A, Holmang A. Prenatal exposure to interleukin-6 results in inflammatory neurodegeneration in hippocampus with NMDA/GABAA dysregulation and impaired spatial learning. *American Journal of Physiology-Regulatory, Integrative and Comparative Physiology* 2006; **290**(5)**:** R1345-R1356.

38. Rasmussen JM, Graham AM, Entringer S, Gilmore JH, Styner M, Fair DA *et al.* Maternal Interleukin-6 concentration during pregnancy is associated with variation in frontolimbic white matter and cognitive development in early life. *Neuroimage* 2019; **185:** 825-835.

39. Miranda-Dominguez O, Mills BD, Grayson D, Woodall A, Grant KA, Kroenke CD *et al.* Bridging the gap between the human and macaque connectome: a quantitative comparison of global interspecies structure-function relationships and network topology. *J Neurosci* 2014; **34**(16)**:** 5552-5563.

40. Grayson DS, Fair DA. Development of large-scale functional networks from birth to adulthood: A guide to the neuroimaging literature. *Neuroimage* 2017; **160:** 15-31.

41. Meyer U, Murray PJ, Urwyler A, Yee BK, Schedlowski M, Feldon J. Adult behavioral and pharmacological dysfunctions following disruption of the fetal brain balance between pro-inflammatory and IL-10-mediated anti-inflammatory signaling. *Mol Psychiatry* 2008; **13**(2)**:** 208-221.

42. Puntambekar SS, Hinton DR, Yin X, Savarin C, Bergmann CC, Trapp BD *et al.* Interleukin-10 is a critical regulator of white matter lesion containment following viral induced demyelination. *Glia* 2015; **63**(11)**:** 2106-2120.

43. Fu G, Zhang W, Dai J, Liu J, Li F, Wu D *et al.* Increased Peripheral Interleukin 10 Relate to White Matter Integrity in Schizophrenia. *Front Neurosci* 2019; **13:** 52.

44. Buyanova IS, Arsalidou M. Cerebral White Matter Myelination and Relations to Age, Gender, and Cognition: A Selective Review. *Front Hum Neurosci* 2021; **15:** 662031.

45. Kinney HC, Brody BA, Kloman AS, Gilles FH. Sequence of central nervous system myelination in human infancy. II. Patterns of myelination in autopsied infants. *J Neuropathol Exp Neurol* 1988; **47**(3)**:** 217-234.

46. Brody BA, Kinney HC, Kloman AS, Gilles FH. Sequence of central nervous system myelination in human infancy. I. An autopsy study of myelination. *J Neuropathol Exp Neurol* 1987; **46**(3)**:** 283-301.

47. Yakovlev PI. The myelogenetic cycles of regional maturation of the brain. *Regional development of the brain in early life* 1967**:** 3-70.
